# Supplementary material for: Machine-Learning-Based Prediction of 1-Year Arrhythmia Recurrence after Ventricular Tachycardia Ablation in Patients with Structural Heart Disease
Source: Bioengineering (Basel). 2023 Dec 1;10(12):1386. doi: 10.3390/bioengineering10121386 (PMC10740977; doi:10.3390/bioengineering10121386)
Supplement: Supplementary file 1 [file bioengineering-10-01386-s001.zip › bioengineering-2668064-supplementary.pdf]

Machine Learning Based Prediction of 1-year Arrhythmia Recurrence after Ventricular Tachycardia Ablation in Patients with Structural Heart Disease

Supplement to *Materials and Methods 2.5* – Machine learning pipeline

Table of contents:

S1. Methods

1.a Supplementary Table S1a: Model hyperparameters for feature ranking

1.b Supplementary Table S1b: Model hyperparameters for feature group ranking

1.c Supplementary Table S1c: Final model selection using Bayesian grid search

S2. Results

Supplementary Table S2a: Feature selection overview

Supplementary Table S2b: Feature ranking results

Supplementary Table S2c: Feature group ranking results

Supplementary Table S2d: Final model selection results

S1. Methods

a. **Supplementary Table S1a** Model hyperparameters for feature ranking (5-fold cross-validation)

The following tables show the hyperparameter spaces utilized for the grid search during the feature ranking. For Multilayer Perceptron (MLP), Random Forest (RF) and Extreme Gradient Boosting (XGB) classifiers the respective hyperparameters are listed (Parameter column), along with the possible values assessed by the grid search (Search space column).

i. MLP

	Parameter	Search space
MLP	'NN__hidden_layer_sizes'	[(5,), (10,), (10, 5), (5, 5)]
	'NN__alpha'	[1, 0.01, 0.001]
	'NN__max_iter'	[200]
	'NN__activation'	['identity', 'tanh', 'relu']
	'NN__solver'	['adam', 'lbfgs']
	'NN__random_state'	

ii. RF

	Parameter	Search space
RF	'max_depth'	[2, 3]
	'max_features'	[2, 3]
	'min_samples_leaf'	[5, 10]
	'criterion'	['gini', 'log_loss']
	'n_estimators'	[500]
	'random_state'	

iii. XGB

	Parameter	Search space
XGB	'min_child_weight'	[5, 10]
	'gamma'	[0.5, 1, 1.5, 2]
	'subsample'	[0.6, 0.8]
	'colsample_bytree'	[0.6, 0.8]
	'max_depth'	[2, 3]
	'random_state'	

b. **Supplementary Table S1b**: Model hyperparameters for feature group ranking (5-fold cross-validation). The following tables show the hyperparameter spaces utilized for the grid search during the feature group ranking. For Multilayer Perceptron (MLP), Random Forest (RF) and Extreme Gradient Boosting (XGB) classifiers the respective hyperparameters are listed (Parameter column), along with the possible values assessed by the grid search (Search space column).

i. MLP

	Parameter	Search space
MLP	'NN__hidden_layer_sizes'	[(20,), (5, 4, 3), (5,), (10,), (10, 5), (5, 5)]
	'NN__alpha'	[10, 1, 0.01, 0.001, 0.0001]
	'NN__max_iter'	[200, 150]
	'NN__activation'	['identity', 'logistic', 'tanh', 'relu']
	'NN__solver'	['adam', 'lbfgs']
	'NN__random_state'	[0]

ii. RF

	Parameter	Search space
RF	'max_depth'	[2, 3, 5]
	'max_features'	[2, 3, 5]
	'min_samples_leaf'	[5, 10, 20]
	'criterion'	['gini', 'log_loss']
	'n_estimators'	[100, 500, 700]
	'random_state'	[0, 42]

iii. XGB

	Parameter	Search space
XGB	'min_child_weight'	[1, 5, 10]
	'gamma'	[0.5, 1, 1.5, 2, 5]
	'subsample'	[0.6, 0.8, 1.0]
	'colsample_bytree'	[0.6, 0.8, 1.0]
	'max_depth'	[3, 4, 5]
	'random_state'	[0, 42]

c. **Supplementary Table S1c:** Final model selection using Bayesian grid search, hyperparameter space. "Parameter" shows the name of parameter and "Search space" represents the options tested during grid search.

	Parameter	Search space
MLP	'NN__hidden_layer_sizes'	20, 15, 10, 25, 9, 8, 7, 6, 5, 4, 3, 22, 24, 27, 30, 35, 40, 50
	'NN__alpha'	Real numbers between 0.0001 and 10, 'log-uniform' distribution
	'NN__max_iter'	Integers between 50 and 500
	'NN__activation'	'identity', 'logistic', 'tanh', 'relu'
	'NN__solver'	'adam', 'lbfgs'
	'NN__random_state'	0

	Parameter	Search space
RF	'max_depth'	Integers between 1 and 5
	'max_features'	Integers between 1 and 9
	'min_samples_leaf'	Integers between 1 and 20, 'log-uniform' distribution
	'criterion'	'gini', 'log_loss'
	'RF__class_weight'	'balanced', 'balanced_subsample'
	'RF__random_state'	Integers between 0 and 100

	Parameter	Search space
XGB	'min_child_weight'	Real numbers between 0 and 50
	'gamma'	Real numbers between 0.1 and 5
	'subsample'	Real numbers between 0.5 and 0.7
	'colsample_bytree'	Real numbers between 0.5 and 1
	'max_depth'	Integers between 1 and 3
	'random_state'	0
	'alpha'	Real numbers between 5 and 30
	'Scale_pos_weight'	Real numbers between 0.5 and 2

S2. Results

d. Feature selection overview

Supplementary Table S2a: Feature selection overview: this table lists the 31 recorded features and shows which features were excluded at certain steps of the feature selection process, as well as the features selected for the two final classifiers.

Features manually excluded	Male
	Hypertension
	Diabetes
	COPD
	SCD
	CAD
	ICD
	CRT
	HF
	Amiodarone
	Beta blocker
	Clinical VT inducible
	Non-clinical VT inducible
	Major complications
Features excluded by machine learning based selection	All VTs eliminated
	Atrial fibrillation
	Clinical VT cycle length
	NYHA status
	E wave deceleration time (DT)
	TR III-IV
	Electrical Storm
	Age
Features used only in one-year model	Inducible VT morphologies
	LVESD
	MR
Features used in both models	HD instability
	ICD shock
Features used only in one-month model	Incessant VT
	LVEF
	TAPSE
	Clinical VT eliminated

Abbreviations: COPD: chronic obstructive pulmonary disease, SCD: sudden cardiac death, CAD: coronary artery disease, ICD: implantable cardioverter defibrillator, CRT: cardiac resynchronization therapy, HF: heart failure, VT: ventricular tachycardia, NYHA: New York Heart Association, TR: tricuspid regurgitation, LVESD: left ventricular end

systolic diameter, MR: mitral regurgitation, HD: hemodynamic, LVEF: left ventricular ejection fraction, TAPSE: tricuspid annular plane systolic excursion

e. **Supplementary Table S2b**: Feature ranking results for 100 iterations using each approach. Through each iteration, the hyperparameters yielding the highest AUC were selected, and the resulting model was used to determine the feature ranking.

Each row represents an iteration, where

- the "iteration number (random_state)" column shows the index of the given iteration out of the 100; this value matches the random state used for that iteration.
- the "Model" column shows the type of classifier and approach used:
  - MLP, PI: Multilayer Perceptron combined with permutation importance
  - RF, PI: Random Forest combined with permutation importance
  - XGB, PI: Extreme Gradient Boosting combined with permutation importance
  - XGB, RFE: Extreme Gradient Boosting combined with recursive feature elimination
  - AVG: the average ranks across the above four methods, for a corresponding iteration (e.g. AVG iteration 0 is derived from the average ranks across iteration 0 of MLP PI, RF PI, XGB PI and XGB RFE rankings),
- the "Parameters" column shows the selected best hyperparameters during the iteration, and
- in each consequent column gives the rank of the respective feature, as determined in that iteration.

i. 1-month VT-recurrence

Iteration number (random_state)	Model	Parameters	Age	Atrial fibrillation	NYHA	EF	LVESD	TAPSE	E wave deceleration time (DT)	MR	TR III-IV	ICD shock	HD instability	Incessant_VT	ES or multiple ICD therapies	Inducible VT morphologies	Clinical VT cycle length	Clinical VT eliminated
0	MLP, PI	{'NN__activation': 'identity', 'NN__alpha': 1, 'NN__hidden_layer_sizes': (5,), 'NN__max_iter': 200, 'NN__random_state': 0, 'NN__solver': 'adam'}	12	6	14	4	13	0	15	7	16	5	1	2	11	3	10	9
1	MLP, PI	{'NN__activation': 'identity', 'NN__alpha': 1, 'NN__hidden_layer_sizes': (5,), 'NN__max_iter': 200, 'NN__random_state': 1, 'NN__solver': 'adam'}	9	12	14	6	11	0	7	8	16	1	2	3	10	5	15	13
2	MLP, PI	{'NN__activation': 'identity', 'NN__alpha': 1, 'NN__hidden_layer_sizes': (5,), 'NN__max_iter': 200, 'NN__random_state': 2, 'NN__solver': 'adam'}	15	10	13	2	12	1	7	6	16	4	0	5	8	9	11	3
3	MLP, PI	{'NN__activation': 'identity', 'NN__alpha': 1, 'NN__hidden_layer_sizes': (5,), 'NN__max_iter': 200, 'NN__random_state': 3, 'NN__solver': 'adam'}	14	6	12	7	10	2	16	3	15	4	1	0	13	9	11	5
4	MLP, PI	{'NN__activation': 'identity', 'NN__alpha': 1, 'NN__hidden_layer_sizes': (5,), 'NN__max_iter': 200, 'NN__random_state': 4, 'NN__solver': 'adam'}	12	9	15	5	13	0	10	6	16	8	1	7	2	4	14	3
5	MLP, PI	{'NN__activation': 'identity', 'NN__alpha': 1, 'NN__hidden_layer_sizes': (5,), 'NN__max_iter': 200, 'NN__random_state': 5, 'NN__solver': 'adam'}	12	10	14	3	9	0	7	6	16	5	2	13	1	8	15	4

6	MLP, PI	{'NN__activation': 'identity', 'NN__alpha': 1, 'NN__hidden_layer_sizes': (5,), 'NN__max_iter': 200, 'NN__random_state': 6, 'NN__solver': 'adam'}	13	10	14	4	15	1	9	5	16	2	0	3	8	7	12	6
7	MLP, PI	{'NN__activation': 'identity', 'NN__alpha': 1, 'NN__hidden_layer_sizes': (5,), 'NN__max_iter': 200, 'NN__random_state': 7, 'NN__solver': 'adam'}	12	6	10	7	14	1	13	8	16	9	2	3	0	5	15	4
8	MLP, PI	{'NN__activation': 'identity', 'NN__alpha': 1, 'NN__hidden_layer_sizes': (5,), 'NN__max_iter': 200, 'NN__random_state': 8, 'NN__solver': 'adam'}	14	5	15	3	8	4	13	7	10	2	1	0	12	9	16	11
9	MLP, PI	{'NN__activation': 'identity', 'NN__alpha': 1, 'NN__hidden_layer_sizes': (5,), 'NN__max_iter': 200, 'NN__random_state': 9, 'NN__solver': 'adam'}	11	4	13	1	12	2	10	9	15	14	0	6	16	5	7	3
10	MLP, PI	{'NN__activation': 'identity', 'NN__alpha': 1, 'NN__hidden_layer_sizes': (5,), 'NN__max_iter': 200, 'NN__random_state': 10, 'NN__solver': 'adam'}	13	10	15	6	9	0	12	7	16	2	1	4	8	5	14	3
11	MLP, PI	{'NN__activation': 'identity', 'NN__alpha': 1, 'NN__hidden_layer_sizes': (5,), 'NN__max_iter': 200, 'NN__random_state': 11, 'NN__solver': 'adam'}	13	10	14	4	11	1	8	7	15	6	2	5	0	9	16	3
12	MLP, PI	{'NN__activation': 'identity', 'NN__alpha': 1, 'NN__hidden_layer_sizes': (5,), 'NN__max_iter': 200, 'NN__random_state': 12, 'NN__solver': 'adam'}	13	2	12	5	14	1	15	6	16	3	9	0	10	4	11	7
13	MLP, PI	{'NN__activation': 'identity', 'NN__alpha': 1, 'NN__hidden_layer_sizes': (5,), 'NN__max_iter': 200, 'NN__random_state': 13, 'NN__solver': 'adam'}	11	4	13	6	9	2	5	8	12	15	0	1	10	7	16	3
14	MLP, PI	{'NN__activation': 'identity', 'NN__alpha': 1, 'NN__hidden_layer_sizes': (5,), 'NN__max_iter': 200, 'NN__random_state': 14, 'NN__solver': 'adam'}	13	12	14	2	15	1	10	8	16	4	6	7	0	5	11	3
15	MLP, PI	{'NN__activation': 'identity', 'NN__alpha': 1, 'NN__hidden_layer_sizes': (5,), 'NN__max_iter': 200, 'NN__random_state': 15, 'NN__solver': 'adam'}	11	10	13	16	4	2	9	7	15	8	1	5	0	6	14	3

16	MLP, PI	{'NN__activation': 'identity', 'NN__alpha': 1, 'NN__hidden_layer_sizes': (5,), 'NN__max_iter': 200, 'NN__random_state': 16, 'NN__solver': 'adam'}	12	10	13	2	15	4	6	5	16	3	1	0	8	9	14	7
17	MLP, PI	{'NN__activation': 'identity', 'NN__alpha': 1, 'NN__hidden_layer_sizes': (5,), 'NN__max_iter': 200, 'NN__random_state': 17, 'NN__solver': 'adam'}	12	7	14	4	11	0	13	9	16	3	1	2	15	5	10	8
18	MLP, PI	{'NN__activation': 'identity', 'NN__alpha': 1, 'NN__hidden_layer_sizes': (5,), 'NN__max_iter': 200, 'NN__random_state': 18, 'NN__solver': 'adam'}	14	10	12	4	13	1	9	5	16	3	0	2	11	6	15	8
19	MLP, PI	{'NN__activation': 'identity', 'NN__alpha': 1, 'NN__hidden_layer_sizes': (5,), 'NN__max_iter': 200, 'NN__random_state': 19, 'NN__solver': 'adam'}	15	8	14	5	10	1	12	7	16	9	2	0	3	6	11	4
20	MLP, PI	{'NN__activation': 'identity', 'NN__alpha': 1, 'NN__hidden_layer_sizes': (5,), 'NN__max_iter': 200, 'NN__random_state': 20, 'NN__solver': 'adam'}	13	5	12	6	7	1	15	9	16	4	0	3	11	8	14	2
21	MLP, PI	{'NN__activation': 'identity', 'NN__alpha': 1, 'NN__hidden_layer_sizes': (5,), 'NN__max_iter': 200, 'NN__random_state': 21, 'NN__solver': 'adam'}	13	11	15	12	3	0	10	7	16	4	1	8	2	5	14	6
22	MLP, PI	{'NN__activation': 'identity', 'NN__alpha': 1, 'NN__hidden_layer_sizes': (5,), 'NN__max_iter': 200, 'NN__random_state': 22, 'NN__solver': 'adam'}	13	10	14	8	12	1	3	6	16	4	0	2	9	7	15	5
23	MLP, PI	{'NN__activation': 'identity', 'NN__alpha': 1, 'NN__hidden_layer_sizes': (5,), 'NN__max_iter': 200, 'NN__random_state': 23, 'NN__solver': 'adam'}	14	9	6	15	3	1	12	8	11	4	0	16	2	7	13	5
24	MLP, PI	{'NN__activation': 'identity', 'NN__alpha': 1, 'NN__hidden_layer_sizes': (5,), 'NN__max_iter': 200, 'NN__random_state': 24, 'NN__solver': 'adam'}	13	7	14	10	8	0	9	5	16	3	1	15	2	4	12	6
25	MLP, PI	{'NN__activation': 'identity', 'NN__alpha': 1, 'NN__hidden_layer_sizes': (5,), 'NN__max_iter': 200, 'NN__random_state': 25, 'NN__solver': 'adam'}	13	12	15	5	8	1	9	7	11	4	0	3	10	6	16	2

26	MLP, PI	{'NN__activation': 'identity', 'NN__alpha': 1, 'NN__hidden_layer_sizes': (5,), 'NN__max_iter': 200, 'NN__random_state': 26, 'NN__solver': 'adam'}	15	4	12	3	5	1	6	10	14	16	0	7	8	9	11	2
27	MLP, PI	{'NN__activation': 'identity', 'NN__alpha': 1, 'NN__hidden_layer_sizes': (5,), 'NN__max_iter': 200, 'NN__random_state': 27, 'NN__solver': 'adam'}	11	5	12	2	10	4	8	9	14	6	1	3	13	7	15	0
28	MLP, PI	{'NN__activation': 'identity', 'NN__alpha': 1, 'NN__hidden_layer_sizes': (5,), 'NN__max_iter': 200, 'NN__random_state': 28, 'NN__solver': 'adam'}	14	8	16	3	15	10	11	2	13	0	1	7	4	5	12	6
29	MLP, PI	{'NN__activation': 'identity', 'NN__alpha': 1, 'NN__hidden_layer_sizes': (5,), 'NN__max_iter': 200, 'NN__random_state': 29, 'NN__solver': 'adam'}	11	10	15	4	12	0	9	5	16	3	2	14	1	7	13	6
30	MLP, PI	{'NN__activation': 'identity', 'NN__alpha': 1, 'NN__hidden_layer_sizes': (5,), 'NN__max_iter': 200, 'NN__random_state': 30, 'NN__solver': 'adam'}	11	4	15	1	10	2	9	7	16	5	0	12	6	8	14	3
31	MLP, PI	{'NN__activation': 'identity', 'NN__alpha': 1, 'NN__hidden_layer_sizes': (5,), 'NN__max_iter': 200, 'NN__random_state': 31, 'NN__solver': 'adam'}	12	5	15	10	11	1	7	6	16	3	2	0	9	13	14	4
32	MLP, PI	{'NN__activation': 'identity', 'NN__alpha': 1, 'NN__hidden_layer_sizes': (5,), 'NN__max_iter': 200, 'NN__random_state': 32, 'NN__solver': 'adam'}	11	10	14	5	9	2	6	8	16	3	1	0	13	7	15	4
33	MLP, PI	{'NN__activation': 'identity', 'NN__alpha': 1, 'NN__hidden_layer_sizes': (5,), 'NN__max_iter': 200, 'NN__random_state': 33, 'NN__solver': 'adam'}	16	7	14	9	10	1	6	8	13	4	0	11	2	5	15	3
34	MLP, PI	{'NN__activation': 'identity', 'NN__alpha': 1, 'NN__hidden_layer_sizes': (5,), 'NN__max_iter': 200, 'NN__random_state': 34, 'NN__solver': 'adam'}	15	12	11	6	14	2	4	3	16	5	0	10	1	8	13	7
35	MLP, PI	{'NN__activation': 'identity', 'NN__alpha': 1, 'NN__hidden_layer_sizes': (5,), 'NN__max_iter': 200, 'NN__random_state': 35, 'NN__solver': 'adam'}	12	11	13	8	10	1	7	5	16	3	2	14	0	6	15	4

36	MLP, PI	{'NN__activation': 'identity', 'NN__alpha': 1, 'NN__hidden_layer_sizes': (5,), 'NN__max_iter': 200, 'NN__random_state': 36, 'NN__solver': 'adam'}	10	12	15	6	7	1	8	4	16	3	2	0	14	9	13	5
37	MLP, PI	{'NN__activation': 'identity', 'NN__alpha': 1, 'NN__hidden_layer_sizes': (5,), 'NN__max_iter': 200, 'NN__random_state': 37, 'NN__solver': 'adam'}	14	7	13	4	9	0	16	6	15	5	2	11	1	8	12	3
38	MLP, PI	{'NN__activation': 'identity', 'NN__alpha': 1, 'NN__hidden_layer_sizes': (5,), 'NN__max_iter': 200, 'NN__random_state': 38, 'NN__solver': 'adam'}	11	6	9	5	2	16	3	14	10	15	0	4	7	8	12	1
39	MLP, PI	{'NN__activation': 'identity', 'NN__alpha': 1, 'NN__hidden_layer_sizes': (5,), 'NN__max_iter': 200, 'NN__random_state': 39, 'NN__solver': 'adam'}	10	12	14	0	15	9	7	8	13	5	3	16	1	4	6	2
40	MLP, PI	{'NN__activation': 'identity', 'NN__alpha': 1, 'NN__hidden_layer_sizes': (5,), 'NN__max_iter': 200, 'NN__random_state': 40, 'NN__solver': 'adam'}	12	15	14	5	7	4	8	11	13	3	0	6	2	9	10	16
41	MLP, PI	{'NN__activation': 'identity', 'NN__alpha': 1, 'NN__hidden_layer_sizes': (5,), 'NN__max_iter': 200, 'NN__random_state': 41, 'NN__solver': 'adam'}	15	6	12	4	13	2	7	11	14	3	0	1	16	5	10	8
42	MLP, PI	{'NN__activation': 'identity', 'NN__alpha': 1, 'NN__hidden_layer_sizes': (5,), 'NN__max_iter': 200, 'NN__random_state': 42, 'NN__solver': 'adam'}	13	7	12	3	14	1	6	16	15	9	2	8	0	4	10	5
43	MLP, PI	{'NN__activation': 'identity', 'NN__alpha': 1, 'NN__hidden_layer_sizes': (5,), 'NN__max_iter': 200, 'NN__random_state': 43, 'NN__solver': 'adam'}	10	9	14	3	7	1	15	6	16	5	0	2	13	8	11	4
44	MLP, PI	{'NN__activation': 'identity', 'NN__alpha': 1, 'NN__hidden_layer_sizes': (5,), 'NN__max_iter': 200, 'NN__random_state': 44, 'NN__solver': 'adam'}	14	10	15	9	12	2	4	3	13	1	0	8	11	5	16	6
45	MLP, PI	{'NN__activation': 'identity', 'NN__alpha': 1, 'NN__hidden_layer_sizes': (5,), 'NN__max_iter': 200, 'NN__random_state': 45, 'NN__solver': 'adam'}	12	8	14	5	9	1	10	7	16	2	0	15	4	6	13	3

46	MLP, PI	{'NN__activation': 'identity', 'NN__alpha': 1, 'NN__hidden_layer_sizes': (5,), 'NN__max_iter': 200, 'NN__random_state': 46, 'NN__solver': 'adam'}	13	9	14	10	8	1	7	12	16	2	0	6	4	5	15	3
47	MLP, PI	{'NN__activation': 'identity', 'NN__alpha': 1, 'NN__hidden_layer_sizes': (5,), 'NN__max_iter': 200, 'NN__random_state': 47, 'NN__solver': 'adam'}	14	11	15	4	12	1	8	7	16	2	0	10	3	9	13	5
48	MLP, PI	{'NN__activation': 'identity', 'NN__alpha': 1, 'NN__hidden_layer_sizes': (5,), 'NN__max_iter': 200, 'NN__random_state': 48, 'NN__solver': 'adam'}	11	9	16	5	13	1	7	6	14	2	0	10	8	4	15	3
49	MLP, PI	{'NN__activation': 'identity', 'NN__alpha': 1, 'NN__hidden_layer_sizes': (5,), 'NN__max_iter': 200, 'NN__random_state': 49, 'NN__solver': 'adam'}	12	8	13	5	11	1	16	6	15	4	2	9	0	3	14	7
50	MLP, PI	{'NN__activation': 'identity', 'NN__alpha': 1, 'NN__hidden_layer_sizes': (5,), 'NN__max_iter': 200, 'NN__random_state': 50, 'NN__solver': 'adam'}	14	10	11	3	15	1	9	5	16	2	0	4	12	7	13	6
51	MLP, PI	{'NN__activation': 'identity', 'NN__alpha': 1, 'NN__hidden_layer_sizes': (5,), 'NN__max_iter': 200, 'NN__random_state': 51, 'NN__solver': 'adam'}	14	10	13	3	12	0	11	5	15	1	16	2	9	6	7	4
52	MLP, PI	{'NN__activation': 'identity', 'NN__alpha': 1, 'NN__hidden_layer_sizes': (5,), 'NN__max_iter': 200, 'NN__random_state': 52, 'NN__solver': 'adam'}	14	8	13	3	12	1	10	7	16	5	0	15	2	6	11	4
53	MLP, PI	{'NN__activation': 'identity', 'NN__alpha': 1, 'NN__hidden_layer_sizes': (5,), 'NN__max_iter': 200, 'NN__random_state': 53, 'NN__solver': 'adam'}	13	7	11	10	12	1	9	5	14	4	0	3	16	6	15	2
54	MLP, PI	{'NN__activation': 'identity', 'NN__alpha': 1, 'NN__hidden_layer_sizes': (5,), 'NN__max_iter': 200, 'NN__random_state': 54, 'NN__solver': 'adam'}	13	9	12	5	15	1	14	6	16	8	2	3	0	4	11	7
55	MLP, PI	{'NN__activation': 'identity', 'NN__alpha': 1, 'NN__hidden_layer_sizes': (5,), 'NN__max_iter': 200, 'NN__random_state': 55, 'NN__solver': 'adam'}	12	11	13	9	5	2	8	7	14	3	1	10	0	6	15	16

56	MLP, PI	{'NN__activation': 'identity', 'NN__alpha': 1, 'NN__hidden_layer_sizes': (5,), 'NN__max_iter': 200, 'NN__random_state': 56, 'NN__solver': 'adam'}	13	7	12	4	11	3	8	6	15	14	0	9	1	5	10	2
57	MLP, PI	{'NN__activation': 'identity', 'NN__alpha': 1, 'NN__hidden_layer_sizes': (5,), 'NN__max_iter': 200, 'NN__random_state': 57, 'NN__solver': 'adam'}	14	10	13	8	9	2	6	5	16	4	0	15	1	7	11	3
58	MLP, PI	{'NN__activation': 'identity', 'NN__alpha': 1, 'NN__hidden_layer_sizes': (5,), 'NN__max_iter': 200, 'NN__random_state': 58, 'NN__solver': 'adam'}	10	9	12	5	13	1	8	6	16	7	0	15	2	4	14	3
59	MLP, PI	{'NN__activation': 'identity', 'NN__alpha': 1, 'NN__hidden_layer_sizes': (5,), 'NN__max_iter': 200, 'NN__random_state': 59, 'NN__solver': 'adam'}	14	9	11	10	4	2	6	5	13	7	1	16	0	8	15	3
60	MLP, PI	{'NN__activation': 'identity', 'NN__alpha': 1, 'NN__hidden_layer_sizes': (5,), 'NN__max_iter': 200, 'NN__random_state': 60, 'NN__solver': 'adam'}	12	8	14	6	9	1	7	4	15	3	0	2	13	10	16	5
61	MLP, PI	{'NN__activation': 'identity', 'NN__alpha': 1, 'NN__hidden_layer_sizes': (5,), 'NN__max_iter': 200, 'NN__random_state': 61, 'NN__solver': 'adam'}	13	9	15	2	14	1	8	7	16	3	5	10	0	4	12	6
62	MLP, PI	{'NN__activation': 'identity', 'NN__alpha': 1, 'NN__hidden_layer_sizes': (5,), 'NN__max_iter': 200, 'NN__random_state': 62, 'NN__solver': 'adam'}	9	12	13	16	2	1	5	10	15	3	0	4	7	8	14	6
63	MLP, PI	{'NN__activation': 'identity', 'NN__alpha': 1, 'NN__hidden_layer_sizes': (5,), 'NN__max_iter': 200, 'NN__random_state': 63, 'NN__solver': 'adam'}	13	4	15	1	11	14	16	5	9	7	2	6	0	8	12	3
64	MLP, PI	{'NN__activation': 'identity', 'NN__alpha': 1, 'NN__hidden_layer_sizes': (5,), 'NN__max_iter': 200, 'NN__random_state': 64, 'NN__solver': 'adam'}	14	8	12	1	16	2	7	6	15	5	0	3	13	9	11	4
65	MLP, PI	{'NN__activation': 'identity', 'NN__alpha': 1, 'NN__hidden_layer_sizes': (5,), 'NN__max_iter': 200, 'NN__random_state': 65, 'NN__solver': 'adam'}	11	6	16	4	9	14	3	5	12	1	0	2	10	7	13	8

66	MLP, PI	{'NN__activation': 'identity', 'NN__alpha': 1, 'NN__hidden_layer_sizes': (5,), 'NN__max_iter': 200, 'NN__random_state': 66, 'NN__solver': 'adam'}	9	8	13	10	4	2	11	6	15	1	16	14	0	5	12	7
67	MLP, PI	{'NN__activation': 'identity', 'NN__alpha': 1, 'NN__hidden_layer_sizes': (5,), 'NN__max_iter': 200, 'NN__random_state': 67, 'NN__solver': 'adam'}	12	9	15	4	13	1	10	6	16	2	3	14	0	7	11	8
68	MLP, PI	{'NN__activation': 'identity', 'NN__alpha': 1, 'NN__hidden_layer_sizes': (5,), 'NN__max_iter': 200, 'NN__random_state': 68, 'NN__solver': 'adam'}	14	9	15	3	13	1	8	7	16	2	0	5	10	6	12	4
69	MLP, PI	{'NN__activation': 'identity', 'NN__alpha': 1, 'NN__hidden_layer_sizes': (5,), 'NN__max_iter': 200, 'NN__random_state': 69, 'NN__solver': 'adam'}	10	12	14	2	9	1	5	4	16	6	0	3	11	8	15	7
70	MLP, PI	{'NN__activation': 'identity', 'NN__alpha': 1, 'NN__hidden_layer_sizes': (5,), 'NN__max_iter': 200, 'NN__random_state': 70, 'NN__solver': 'adam'}	12	9	11	3	14	1	7	6	16	4	2	0	15	8	13	5
71	MLP, PI	{'NN__activation': 'identity', 'NN__alpha': 1, 'NN__hidden_layer_sizes': (5,), 'NN__max_iter': 200, 'NN__random_state': 71, 'NN__solver': 'adam'}	12	7	15	16	1	5	3	11	10	6	2	0	9	8	13	4
72	MLP, PI	{'NN__activation': 'identity', 'NN__alpha': 1, 'NN__hidden_layer_sizes': (5,), 'NN__max_iter': 200, 'NN__random_state': 72, 'NN__solver': 'adam'}	12	10	14	11	5	0	9	8	16	3	1	13	2	4	15	6
73	MLP, PI	{'NN__activation': 'identity', 'NN__alpha': 1, 'NN__hidden_layer_sizes': (5,), 'NN__max_iter': 200, 'NN__random_state': 73, 'NN__solver': 'adam'}	11	6	15	2	14	7	10	9	12	3	1	0	4	8	13	16
74	MLP, PI	{'NN__activation': 'identity', 'NN__alpha': 1, 'NN__hidden_layer_sizes': (5,), 'NN__max_iter': 200, 'NN__random_state': 74, 'NN__solver': 'adam'}	13	11	14	7	5	4	8	9	12	2	1	0	3	10	16	15
75	MLP, PI	{'NN__activation': 'identity', 'NN__alpha': 1, 'NN__hidden_layer_sizes': (5,), 'NN__max_iter': 200, 'NN__random_state': 75, 'NN__solver': 'adam'}	14	10	12	4	3	1	7	16	13	2	0	8	11	6	15	5

76	MLP, PI	{'NN__activation': 'identity', 'NN__alpha': 1, 'NN__hidden_layer_sizes': (5,), 'NN__max_iter': 200, 'NN__random_state': 76, 'NN__solver': 'adam'}	12	13	15	4	7	1	11	6	10	3	0	2	14	8	9	16
77	MLP, PI	{'NN__activation': 'identity', 'NN__alpha': 1, 'NN__hidden_layer_sizes': (5,), 'NN__max_iter': 200, 'NN__random_state': 77, 'NN__solver': 'adam'}	15	4	11	3	12	2	6	9	13	5	14	1	16	10	8	0
78	MLP, PI	{'NN__activation': 'identity', 'NN__alpha': 1, 'NN__hidden_layer_sizes': (5,), 'NN__max_iter': 200, 'NN__random_state': 78, 'NN__solver': 'adam'}	15	7	12	3	13	0	11	9	16	2	1	4	14	8	10	6
79	MLP, PI	{'NN__activation': 'identity', 'NN__alpha': 1, 'NN__hidden_layer_sizes': (5,), 'NN__max_iter': 200, 'NN__random_state': 79, 'NN__solver': 'adam'}	14	10	15	12	8	2	9	7	16	3	1	5	0	6	13	4
80	MLP, PI	{'NN__activation': 'identity', 'NN__alpha': 1, 'NN__hidden_layer_sizes': (5,), 'NN__max_iter': 200, 'NN__random_state': 80, 'NN__solver': 'adam'}	12	6	11	5	14	1	8	3	16	4	0	7	13	9	15	2
81	MLP, PI	{'NN__activation': 'identity', 'NN__alpha': 1, 'NN__hidden_layer_sizes': (5,), 'NN__max_iter': 200, 'NN__random_state': 81, 'NN__solver': 'adam'}	12	5	13	1	16	0	10	7	15	2	11	8	3	6	14	4
82	MLP, PI	{'NN__activation': 'identity', 'NN__alpha': 1, 'NN__hidden_layer_sizes': (5,), 'NN__max_iter': 200, 'NN__random_state': 82, 'NN__solver': 'adam'}	12	5	13	4	14	1	16	8	15	10	0	7	2	6	9	3
83	MLP, PI	{'NN__activation': 'identity', 'NN__alpha': 1, 'NN__hidden_layer_sizes': (5,), 'NN__max_iter': 200, 'NN__random_state': 83, 'NN__solver': 'adam'}	15	10	9	0	7	3	11	5	16	2	1	13	4	12	14	6
84	MLP, PI	{'NN__activation': 'identity', 'NN__alpha': 1, 'NN__hidden_layer_sizes': (5,), 'NN__max_iter': 200, 'NN__random_state': 84, 'NN__solver': 'adam'}	14	9	13	4	15	2	10	5	16	3	0	1	11	6	12	8
85	MLP, PI	{'NN__activation': 'identity', 'NN__alpha': 1, 'NN__hidden_layer_sizes': (5,), 'NN__max_iter': 200, 'NN__random_state': 85, 'NN__solver': 'adam'}	11	7	13	8	12	1	9	5	16	2	0	10	6	4	15	3

86	MLP, PI	{'NN__activation': 'identity', 'NN__alpha': 1, 'NN__hidden_layer_sizes': (5,), 'NN__max_iter': 200, 'NN__random_state': 86, 'NN__solver': 'adam'}	12	7	14	4	13	1	9	6	16	2	0	8	10	5	15	3
87	MLP, PI	{'NN__activation': 'identity', 'NN__alpha': 1, 'NN__hidden_layer_sizes': (5,), 'NN__max_iter': 200, 'NN__random_state': 87, 'NN__solver': 'adam'}	14	9	13	12	8	0	6	7	16	2	1	4	10	5	15	3
88	MLP, PI	{'NN__activation': 'identity', 'NN__alpha': 1, 'NN__hidden_layer_sizes': (5,), 'NN__max_iter': 200, 'NN__random_state': 88, 'NN__solver': 'adam'}	12	7	15	3	14	1	9	6	13	5	0	11	4	8	10	2
89	MLP, PI	{'NN__activation': 'identity', 'NN__alpha': 1, 'NN__hidden_layer_sizes': (5,), 'NN__max_iter': 200, 'NN__random_state': 89, 'NN__solver': 'adam'}	12	10	15	3	11	1	5	7	16	4	2	13	0	8	14	6
90	MLP, PI	{'NN__activation': 'identity', 'NN__alpha': 1, 'NN__hidden_layer_sizes': (5,), 'NN__max_iter': 200, 'NN__random_state': 90, 'NN__solver': 'adam'}	12	10	13	6	9	2	8	7	15	3	1	0	4	16	14	5
91	MLP, PI	{'NN__activation': 'identity', 'NN__alpha': 1, 'NN__hidden_layer_sizes': (5,), 'NN__max_iter': 200, 'NN__random_state': 91, 'NN__solver': 'adam'}	9	3	13	5	11	2	7	16	12	4	0	1	14	10	15	6
92	MLP, PI	{'NN__activation': 'identity', 'NN__alpha': 1, 'NN__hidden_layer_sizes': (5,), 'NN__max_iter': 200, 'NN__random_state': 92, 'NN__solver': 'adam'}	13	9	15	4	11	2	8	5	16	3	0	1	14	6	12	7
93	MLP, PI	{'NN__activation': 'identity', 'NN__alpha': 1, 'NN__hidden_layer_sizes': (5,), 'NN__max_iter': 200, 'NN__random_state': 93, 'NN__solver': 'adam'}	15	9	11	2	12	1	8	6	13	4	0	5	10	7	14	3
94	MLP, PI	{'NN__activation': 'identity', 'NN__alpha': 1, 'NN__hidden_layer_sizes': (5,), 'NN__max_iter': 200, 'NN__random_state': 94, 'NN__solver': 'adam'}	6	9	15	0	14	2	10	16	13	4	1	8	7	3	12	5
95	MLP, PI	{'NN__activation': 'identity', 'NN__alpha': 1, 'NN__hidden_layer_sizes': (5,), 'NN__max_iter': 200, 'NN__random_state': 95, 'NN__solver': 'adam'}	13	8	14	4	15	1	10	5	16	9	2	7	0	6	12	3

96	MLP, PI	{'NN__activation': 'identity', 'NN__alpha': 1, 'NN__hidden_layer_sizes': (5,), 'NN__max_iter': 200, 'NN__random_state': 96, 'NN__solver': 'adam'}	10	11	12	16	2	3	5	9	13	4	0	1	15	6	14	8
97	MLP, PI	{'NN__activation': 'identity', 'NN__alpha': 1, 'NN__hidden_layer_sizes': (5,), 'NN__max_iter': 200, 'NN__random_state': 97, 'NN__solver': 'adam'}	15	7	10	9	14	1	0	6	16	5	2	12	3	4	13	8
98	MLP, PI	{'NN__activation': 'identity', 'NN__alpha': 1, 'NN__hidden_layer_sizes': (5,), 'NN__max_iter': 200, 'NN__random_state': 98, 'NN__solver': 'adam'}	13	5	10	1	14	15	4	6	16	7	0	12	3	8	11	2
99	MLP, PI	{'NN__activation': 'identity', 'NN__alpha': 1, 'NN__hidden_layer_sizes': (5,), 'NN__max_iter': 200, 'NN__random_state': 99, 'NN__solver': 'adam'}	12	5	15	2	16	3	10	13	11	4	1	0	7	9	14	6
0	RF, PI	{'criterion': 'gini', 'max_depth': 2, 'max_features': 2, 'min_samples_leaf': 5, 'n_estimators': 500, 'n_jobs': -1, 'random_state': 0}	8	16	12	2	1	3	5	4	10	9	0	14	13	11	6	7
1	RF, PI	{'criterion': 'gini', 'max_depth': 2, 'max_features': 2, 'min_samples_leaf': 5, 'n_estimators': 500, 'n_jobs': -1, 'random_state': 1}	8	16	13	1	0	3	12	7	14	4	2	11	10	9	6	5
2	RF, PI	{'criterion': 'gini', 'max_depth': 2, 'max_features': 2, 'min_samples_leaf': 5, 'n_estimators': 500, 'n_jobs': -1, 'random_state': 2}	4	14	13	1	2	3	11	10	9	8	0	16	7	12	5	6
3	RF, PI	{'criterion': 'gini', 'max_depth': 2, 'max_features': 2, 'min_samples_leaf': 5, 'n_estimators': 500, 'n_jobs': -1, 'random_state': 3}	5	16	15	2	1	3	11	6	12	8	0	10	9	13	7	4
4	RF, PI	{'criterion': 'gini', 'max_depth': 2, 'max_features': 2, 'min_samples_leaf': 5, 'n_estimators': 500, 'n_jobs': -1, 'random_state': 4}	5	16	15	2	0	3	12	7	10	13	1	8	9	11	6	4
5	RF, PI	{'criterion': 'gini', 'max_depth': 2, 'max_features': 2, 'min_samples_leaf': 5, 'n_estimators': 500, 'n_jobs': -1, 'random_state': 5}	4	14	15	2	1	3	12	9	11	10	0	13	6	8	5	7

6	RF, PI	{'criterion': 'gini', 'max_depth': 2, 'max_features': 2, 'min_samples_leaf': 5, 'n_estimators': 500, 'n_jobs': -1, 'random_state': 6}	5	16	15	3	1	2	13	12	11	6	0	10	7	9	4	8
7	RF, PI	{'criterion': 'gini', 'max_depth': 2, 'max_features': 2, 'min_samples_leaf': 5, 'n_estimators': 500, 'n_jobs': -1, 'random_state': 7}	3	13	11	2	0	5	15	8	9	6	1	16	14	10	7	4
8	RF, PI	{'criterion': 'gini', 'max_depth': 2, 'max_features': 2, 'min_samples_leaf': 5, 'n_estimators': 500, 'n_jobs': -1, 'random_state': 8}	6	16	10	2	0	3	13	8	9	11	1	15	4	12	7	5
9	RF, PI	{'criterion': 'gini', 'max_depth': 2, 'max_features': 2, 'min_samples_leaf': 5, 'n_estimators': 500, 'n_jobs': -1, 'random_state': 9}	6	13	11	2	1	3	14	7	9	8	0	15	16	10	4	5
10	RF, PI	{'criterion': 'gini', 'max_depth': 2, 'max_features': 2, 'min_samples_leaf': 5, 'n_estimators': 500, 'n_jobs': -1, 'random_state': 10}	6	13	12	3	2	1	15	8	11	7	0	16	14	9	4	5
11	RF, PI	{'criterion': 'gini', 'max_depth': 2, 'max_features': 2, 'min_samples_leaf': 5, 'n_estimators': 500, 'n_jobs': -1, 'random_state': 11}	8	16	13	2	0	3	9	5	11	6	1	14	10	12	7	4
12	RF, PI	{'criterion': 'gini', 'max_depth': 2, 'max_features': 2, 'min_samples_leaf': 5, 'n_estimators': 500, 'n_jobs': -1, 'random_state': 12}	4	16	13	3	1	2	15	8	11	14	0	6	7	10	5	9
13	RF, PI	{'criterion': 'gini', 'max_depth': 2, 'max_features': 2, 'min_samples_leaf': 5, 'n_estimators': 500, 'n_jobs': -1, 'random_state': 13}	5	15	13	2	1	3	12	9	11	6	0	16	7	10	8	4
14	RF, PI	{'criterion': 'gini', 'max_depth': 2, 'max_features': 2, 'min_samples_leaf': 5, 'n_estimators': 500, 'n_jobs': -1, 'random_state': 14}	4	16	14	2	1	3	15	8	13	6	0	10	11	9	7	5
15	RF, PI	{'criterion': 'gini', 'max_depth': 2, 'max_features': 2, 'min_samples_leaf': 5, 'n_estimators': 500, 'n_jobs': -1, 'random_state': 15}	7	14	13	1	2	3	12	8	10	6	0	16	11	9	4	5

16	RF, PI	{'criterion': 'gini', 'max_depth': 2, 'max_features': 2, 'min_samples_leaf': 5, 'n_estimators': 500, 'n_jobs': -1, 'random_state': 16}	6	16	13	2	1	3	10	5	14	7	0	9	12	11	8	4
17	RF, PI	{'criterion': 'gini', 'max_depth': 2, 'max_features': 2, 'min_samples_leaf': 5, 'n_estimators': 500, 'n_jobs': -1, 'random_state': 17}	6	14	10	2	1	3	13	8	7	9	0	15	12	11	5	4
18	RF, PI	{'criterion': 'gini', 'max_depth': 2, 'max_features': 2, 'min_samples_leaf': 5, 'n_estimators': 500, 'n_jobs': -1, 'random_state': 18}	5	13	10	3	2	1	11	8	12	7	0	16	15	9	6	4
19	RF, PI	{'criterion': 'gini', 'max_depth': 2, 'max_features': 2, 'min_samples_leaf': 5, 'n_estimators': 500, 'n_jobs': -1, 'random_state': 19}	5	16	14	4	0	1	13	9	12	6	2	15	11	10	7	3
20	RF, PI	{'criterion': 'gini', 'max_depth': 2, 'max_features': 2, 'min_samples_leaf': 5, 'n_estimators': 500, 'n_jobs': -1, 'random_state': 20}	5	14	8	3	1	2	16	12	13	6	0	15	11	9	7	4
21	RF, PI	{'criterion': 'gini', 'max_depth': 2, 'max_features': 2, 'min_samples_leaf': 5, 'n_estimators': 500, 'n_jobs': -1, 'random_state': 21}	6	16	14	2	3	1	13	7	12	9	0	10	11	8	5	4
22	RF, PI	{'criterion': 'gini', 'max_depth': 2, 'max_features': 2, 'min_samples_leaf': 5, 'n_estimators': 500, 'n_jobs': -1, 'random_state': 22}	6	15	13	2	1	3	10	9	14	11	0	12	7	8	5	4
23	RF, PI	{'criterion': 'gini', 'max_depth': 2, 'max_features': 2, 'min_samples_leaf': 5, 'n_estimators': 500, 'n_jobs': -1, 'random_state': 23}	4	14	16	2	1	3	9	5	12	7	0	15	13	10	8	6
24	RF, PI	{'criterion': 'gini', 'max_depth': 2, 'max_features': 2, 'min_samples_leaf': 5, 'n_estimators': 500, 'n_jobs': -1, 'random_state': 24}	4	15	14	2	0	3	8	6	12	5	1	16	11	10	7	9
25	RF, PI	{'criterion': 'gini', 'max_depth': 2, 'max_features': 2, 'min_samples_leaf': 5, 'n_estimators': 500, 'n_jobs': -1, 'random_state': 25}	7	16	13	3	0	2	15	9	11	6	1	12	5	10	8	4

26	RF, PI	{'criterion': 'gini', 'max_depth': 2, 'max_features': 2, 'min_samples_leaf': 5, 'n_estimators': 500, 'n_jobs': -1, 'random_state': 26}	5	16	14	3	1	2	8	7	12	11	0	9	10	13	6	4
27	RF, PI	{'criterion': 'gini', 'max_depth': 2, 'max_features': 2, 'min_samples_leaf': 5, 'n_estimators': 500, 'n_jobs': -1, 'random_state': 27}	5	16	12	1	3	2	13	7	14	6	0	15	10	9	8	4
28	RF, PI	{'criterion': 'gini', 'max_depth': 2, 'max_features': 2, 'min_samples_leaf': 5, 'n_estimators': 500, 'n_jobs': -1, 'random_state': 28}	4	15	13	2	1	5	16	8	11	6	0	10	9	12	7	3
29	RF, PI	{'criterion': 'gini', 'max_depth': 2, 'max_features': 2, 'min_samples_leaf': 5, 'n_estimators': 500, 'n_jobs': -1, 'random_state': 29}	5	14	12	2	1	3	11	8	10	7	0	16	15	9	4	6
30	RF, PI	{'criterion': 'gini', 'max_depth': 2, 'max_features': 2, 'min_samples_leaf': 5, 'n_estimators': 500, 'n_jobs': -1, 'random_state': 30}	4	15	16	1	3	2	8	10	13	5	0	12	11	9	7	6
31	RF, PI	{'criterion': 'gini', 'max_depth': 2, 'max_features': 2, 'min_samples_leaf': 5, 'n_estimators': 500, 'n_jobs': -1, 'random_state': 31}	5	15	11	3	1	2	13	7	8	9	0	14	16	12	4	6
32	RF, PI	{'criterion': 'gini', 'max_depth': 2, 'max_features': 2, 'min_samples_leaf': 5, 'n_estimators': 500, 'n_jobs': -1, 'random_state': 32}	5	16	13	2	1	3	14	8	9	7	0	11	12	10	6	4
33	RF, PI	{'criterion': 'gini', 'max_depth': 2, 'max_features': 2, 'min_samples_leaf': 5, 'n_estimators': 500, 'n_jobs': -1, 'random_state': 33}	7	14	12	2	1	3	13	10	16	4	0	11	6	9	8	5
34	RF, PI	{'criterion': 'gini', 'max_depth': 2, 'max_features': 2, 'min_samples_leaf': 5, 'n_estimators': 500, 'n_jobs': -1, 'random_state': 34}	6	16	14	3	1	2	8	4	11	12	0	15	10	9	7	5
35	RF, PI	{'criterion': 'gini', 'max_depth': 2, 'max_features': 2, 'min_samples_leaf': 5, 'n_estimators': 500, 'n_jobs': -1, 'random_state': 35}	4	16	15	2	1	3	14	9	11	6	0	8	10	12	7	5

36	RF, PI	{'criterion': 'gini', 'max_depth': 2, 'max_features': 2, 'min_samples_leaf': 5, 'n_estimators': 500, 'n_jobs': -1, 'random_state': 36}	4	16	14	3	1	2	7	5	12	8	0	9	10	13	11	6
37	RF, PI	{'criterion': 'gini', 'max_depth': 2, 'max_features': 2, 'min_samples_leaf': 5, 'n_estimators': 500, 'n_jobs': -1, 'random_state': 37}	4	13	15	0	2	3	10	8	9	7	1	16	12	11	5	6
38	RF, PI	{'criterion': 'gini', 'max_depth': 2, 'max_features': 2, 'min_samples_leaf': 5, 'n_estimators': 500, 'n_jobs': -1, 'random_state': 38}	4	16	15	2	0	3	8	10	14	7	1	11	6	13	9	5
39	RF, PI	{'criterion': 'gini', 'max_depth': 2, 'max_features': 2, 'min_samples_leaf': 5, 'n_estimators': 500, 'n_jobs': -1, 'random_state': 39}	6	16	12	1	0	3	11	7	10	8	2	14	5	13	9	4
40	RF, PI	{'criterion': 'gini', 'max_depth': 2, 'max_features': 2, 'min_samples_leaf': 5, 'n_estimators': 500, 'n_jobs': -1, 'random_state': 40}	4	16	15	2	0	3	6	13	12	10	1	7	8	11	9	5
41	RF, PI	{'criterion': 'gini', 'max_depth': 2, 'max_features': 2, 'min_samples_leaf': 5, 'n_estimators': 500, 'n_jobs': -1, 'random_state': 41}	5	15	9	3	1	2	12	8	11	7	0	14	13	10	6	4
42	RF, PI	{'criterion': 'gini', 'max_depth': 2, 'max_features': 2, 'min_samples_leaf': 5, 'n_estimators': 500, 'n_jobs': -1, 'random_state': 42}	4	16	11	2	1	3	5	6	12	8	0	15	7	10	9	13
43	RF, PI	{'criterion': 'gini', 'max_depth': 2, 'max_features': 2, 'min_samples_leaf': 5, 'n_estimators': 500, 'n_jobs': -1, 'random_state': 43}	4	15	14	3	1	2	16	7	13	9	0	10	11	12	6	5
44	RF, PI	{'criterion': 'gini', 'max_depth': 2, 'max_features': 2, 'min_samples_leaf': 5, 'n_estimators': 500, 'n_jobs': -1, 'random_state': 44}	4	14	13	1	3	2	10	5	11	7	0	15	16	9	8	6
45	RF, PI	{'criterion': 'gini', 'max_depth': 2, 'max_features': 2, 'min_samples_leaf': 5, 'n_estimators': 500, 'n_jobs': -1, 'random_state': 45}	5	13	12	2	3	1	11	10	9	6	0	16	15	8	7	4

46	RF, PI	{'criterion': 'gini', 'max_depth': 2, 'max_features': 2, 'min_samples_leaf': 5, 'n_estimators': 500, 'n_jobs': -1, 'random_state': 46}	4	13	11	2	3	1	10	14	15	8	0	7	16	9	6	5
47	RF, PI	{'criterion': 'gini', 'max_depth': 2, 'max_features': 2, 'min_samples_leaf': 5, 'n_estimators': 500, 'n_jobs': -1, 'random_state': 47}	5	16	10	2	1	3	14	7	12	11	0	9	6	15	8	4
48	RF, PI	{'criterion': 'gini', 'max_depth': 2, 'max_features': 2, 'min_samples_leaf': 5, 'n_estimators': 500, 'n_jobs': -1, 'random_state': 48}	4	16	15	1	0	3	12	8	13	5	2	14	9	11	7	6
49	RF, PI	{'criterion': 'gini', 'max_depth': 2, 'max_features': 2, 'min_samples_leaf': 5, 'n_estimators': 500, 'n_jobs': -1, 'random_state': 49}	4	14	13	3	1	2	10	7	11	6	0	16	15	9	5	8
50	RF, PI	{'criterion': 'gini', 'max_depth': 2, 'max_features': 2, 'min_samples_leaf': 5, 'n_estimators': 500, 'n_jobs': -1, 'random_state': 50}	6	15	14	2	1	3	10	9	12	7	0	16	13	11	5	4
51	RF, PI	{'criterion': 'gini', 'max_depth': 2, 'max_features': 2, 'min_samples_leaf': 5, 'n_estimators': 500, 'n_jobs': -1, 'random_state': 51}	4	16	15	3	1	2	9	6	12	7	0	13	8	14	10	5
52	RF, PI	{'criterion': 'gini', 'max_depth': 2, 'max_features': 2, 'min_samples_leaf': 5, 'n_estimators': 500, 'n_jobs': -1, 'random_state': 52}	6	15	16	2	0	3	8	5	14	4	1	12	10	11	9	7
53	RF, PI	{'criterion': 'gini', 'max_depth': 2, 'max_features': 2, 'min_samples_leaf': 5, 'n_estimators': 500, 'n_jobs': -1, 'random_state': 53}	4	15	12	2	1	3	13	9	14	6	0	11	8	10	5	7
54	RF, PI	{'criterion': 'gini', 'max_depth': 2, 'max_features': 2, 'min_samples_leaf': 5, 'n_estimators': 500, 'n_jobs': -1, 'random_state': 54}	4	15	13	2	1	3	8	7	12	9	0	16	11	10	6	5
55	RF, PI	{'criterion': 'gini', 'max_depth': 2, 'max_features': 2, 'min_samples_leaf': 5, 'n_estimators': 500, 'n_jobs': -1, 'random_state': 55}	6	15	16	2	1	3	10	8	14	4	0	11	9	12	5	7

56	RF, PI	{'criterion': 'gini', 'max_depth': 2, 'max_features': 2, 'min_samples_leaf': 5, 'n_estimators': 500, 'n_jobs': -1, 'random_state': 56}	9	16	14	3	1	2	15	7	11	4	0	13	12	10	8	6
57	RF, PI	{'criterion': 'gini', 'max_depth': 2, 'max_features': 2, 'min_samples_leaf': 5, 'n_estimators': 500, 'n_jobs': -1, 'random_state': 57}	4	16	14	3	0	2	12	8	11	7	1	10	9	13	6	5
58	RF, PI	{'criterion': 'gini', 'max_depth': 2, 'max_features': 2, 'min_samples_leaf': 5, 'n_estimators': 500, 'n_jobs': -1, 'random_state': 58}	4	16	13	2	3	1	11	5	12	15	0	14	8	9	7	6
59	RF, PI	{'criterion': 'gini', 'max_depth': 2, 'max_features': 2, 'min_samples_leaf': 5, 'n_estimators': 500, 'n_jobs': -1, 'random_state': 59}	4	15	12	1	2	0	10	6	11	14	3	8	9	13	5	7
60	RF, PI	{'criterion': 'gini', 'max_depth': 2, 'max_features': 2, 'min_samples_leaf': 5, 'n_estimators': 500, 'n_jobs': -1, 'random_state': 60}	6	16	15	2	0	3	10	5	9	4	1	14	12	11	8	7
61	RF, PI	{'criterion': 'gini', 'max_depth': 2, 'max_features': 2, 'min_samples_leaf': 5, 'n_estimators': 500, 'n_jobs': -1, 'random_state': 61}	5	13	12	2	0	3	7	8	11	10	1	16	14	9	6	4
62	RF, PI	{'criterion': 'gini', 'max_depth': 2, 'max_features': 2, 'min_samples_leaf': 5, 'n_estimators': 500, 'n_jobs': -1, 'random_state': 62}	4	13	16	2	1	3	12	10	8	7	0	15	9	11	6	5
63	RF, PI	{'criterion': 'gini', 'max_depth': 2, 'max_features': 2, 'min_samples_leaf': 5, 'n_estimators': 500, 'n_jobs': -1, 'random_state': 63}	4	16	13	3	1	2	10	5	11	7	0	12	8	14	9	6
64	RF, PI	{'criterion': 'gini', 'max_depth': 2, 'max_features': 2, 'min_samples_leaf': 5, 'n_estimators': 500, 'n_jobs': -1, 'random_state': 64}	4	16	13	3	0	2	11	8	9	5	1	15	7	12	10	6
65	RF, PI	{'criterion': 'gini', 'max_depth': 2, 'max_features': 2, 'min_samples_leaf': 5, 'n_estimators': 500, 'n_jobs': -1, 'random_state': 65}	3	15	13	2	1	4	12	9	8	6	0	16	11	10	5	7

66	RF, PI	{'criterion': 'gini', 'max_depth': 2, 'max_features': 2, 'min_samples_leaf': 5, 'n_estimators': 500, 'n_jobs': -1, 'random_state': 66}	3	16	13	4	1	2	9	5	12	8	0	11	14	6	7	10
67	RF, PI	{'criterion': 'gini', 'max_depth': 2, 'max_features': 2, 'min_samples_leaf': 5, 'n_estimators': 500, 'n_jobs': -1, 'random_state': 67}	5	16	13	2	1	3	12	9	10	8	0	7	4	15	11	6
68	RF, PI	{'criterion': 'gini', 'max_depth': 2, 'max_features': 2, 'min_samples_leaf': 5, 'n_estimators': 500, 'n_jobs': -1, 'random_state': 68}	7	15	14	3	0	1	16	11	10	5	2	8	12	9	6	4
69	RF, PI	{'criterion': 'gini', 'max_depth': 2, 'max_features': 2, 'min_samples_leaf': 5, 'n_estimators': 500, 'n_jobs': -1, 'random_state': 69}	4	14	11	2	3	0	13	6	7	10	1	16	12	9	5	8
70	RF, PI	{'criterion': 'gini', 'max_depth': 2, 'max_features': 2, 'min_samples_leaf': 5, 'n_estimators': 500, 'n_jobs': -1, 'random_state': 70}	5	16	12	1	2	4	14	6	11	10	0	8	13	9	7	3
71	RF, PI	{'criterion': 'gini', 'max_depth': 2, 'max_features': 2, 'min_samples_leaf': 5, 'n_estimators': 500, 'n_jobs': -1, 'random_state': 71}	4	15	11	1	2	3	7	6	9	14	0	13	12	10	8	5
72	RF, PI	{'criterion': 'gini', 'max_depth': 2, 'max_features': 2, 'min_samples_leaf': 5, 'n_estimators': 500, 'n_jobs': -1, 'random_state': 72}	5	15	12	3	1	2	9	8	6	11	0	16	14	10	7	4
73	RF, PI	{'criterion': 'gini', 'max_depth': 2, 'max_features': 2, 'min_samples_leaf': 5, 'n_estimators': 500, 'n_jobs': -1, 'random_state': 73}	4	16	13	2	0	3	12	5	9	6	1	14	7	8	11	10
74	RF, PI	{'criterion': 'gini', 'max_depth': 2, 'max_features': 2, 'min_samples_leaf': 5, 'n_estimators': 500, 'n_jobs': -1, 'random_state': 74}	8	16	13	1	2	3	10	7	12	6	0	14	4	11	9	5
75	RF, PI	{'criterion': 'gini', 'max_depth': 2, 'max_features': 2, 'min_samples_leaf': 5, 'n_estimators': 500, 'n_jobs': -1, 'random_state': 75}	6	14	12	2	1	3	10	7	9	5	0	16	15	11	8	4

76	RF, PI	{'criterion': 'gini', 'max_depth': 2, 'max_features': 2, 'min_samples_leaf': 5, 'n_estimators': 500, 'n_jobs': -1, 'random_state': 76}	5	16	15	2	1	3	14	6	11	9	0	13	8	10	7	4
77	RF, PI	{'criterion': 'gini', 'max_depth': 2, 'max_features': 2, 'min_samples_leaf': 5, 'n_estimators': 500, 'n_jobs': -1, 'random_state': 77}	4	16	14	1	0	3	7	9	10	6	2	15	11	12	8	5
78	RF, PI	{'criterion': 'gini', 'max_depth': 2, 'max_features': 2, 'min_samples_leaf': 5, 'n_estimators': 500, 'n_jobs': -1, 'random_state': 78}	6	15	12	2	0	3	13	8	10	5	1	14	16	11	7	4
79	RF, PI	{'criterion': 'gini', 'max_depth': 2, 'max_features': 2, 'min_samples_leaf': 5, 'n_estimators': 500, 'n_jobs': -1, 'random_state': 79}	3	16	10	1	4	2	14	8	12	7	0	15	11	9	5	6
80	RF, PI	{'criterion': 'gini', 'max_depth': 2, 'max_features': 2, 'min_samples_leaf': 5, 'n_estimators': 500, 'n_jobs': -1, 'random_state': 80}	4	11	14	0	3	1	15	7	10	5	2	16	13	9	8	6
81	RF, PI	{'criterion': 'gini', 'max_depth': 2, 'max_features': 2, 'min_samples_leaf': 5, 'n_estimators': 500, 'n_jobs': -1, 'random_state': 81}	5	15	13	2	0	3	9	8	14	7	1	11	6	12	10	4
82	RF, PI	{'criterion': 'gini', 'max_depth': 2, 'max_features': 2, 'min_samples_leaf': 5, 'n_estimators': 500, 'n_jobs': -1, 'random_state': 82}	4	14	15	3	1	2	16	9	10	13	0	7	6	12	5	8
83	RF, PI	{'criterion': 'gini', 'max_depth': 2, 'max_features': 2, 'min_samples_leaf': 5, 'n_estimators': 500, 'n_jobs': -1, 'random_state': 83}	2	16	14	5	0	3	11	7	12	4	1	10	9	13	6	8
84	RF, PI	{'criterion': 'gini', 'max_depth': 2, 'max_features': 2, 'min_samples_leaf': 5, 'n_estimators': 500, 'n_jobs': -1, 'random_state': 84}	5	16	12	3	0	2	8	7	14	10	1	13	9	11	4	6
85	RF, PI	{'criterion': 'gini', 'max_depth': 2, 'max_features': 2, 'min_samples_leaf': 5, 'n_estimators': 500, 'n_jobs': -1, 'random_state': 85}	6	14	11	2	1	3	12	7	10	8	0	16	15	9	5	4

86	RF, PI	{'criterion': 'gini', 'max_depth': 2, 'max_features': 2, 'min_samples_leaf': 5, 'n_estimators': 500, 'n_jobs': -1, 'random_state': 86}	8	16	15	1	2	3	14	5	10	6	0	11	13	9	7	4
87	RF, PI	{'criterion': 'gini', 'max_depth': 2, 'max_features': 2, 'min_samples_leaf': 5, 'n_estimators': 500, 'n_jobs': -1, 'random_state': 87}	4	12	15	3	2	1	13	8	9	7	0	16	14	10	5	6
88	RF, PI	{'criterion': 'gini', 'max_depth': 2, 'max_features': 2, 'min_samples_leaf': 5, 'n_estimators': 500, 'n_jobs': -1, 'random_state': 88}	10	16	14	2	0	4	6	11	9	3	1	13	12	15	5	7
89	RF, PI	{'criterion': 'gini', 'max_depth': 2, 'max_features': 2, 'min_samples_leaf': 5, 'n_estimators': 500, 'n_jobs': -1, 'random_state': 89}	4	16	15	1	3	2	8	6	11	5	0	14	10	13	9	7
90	RF, PI	{'criterion': 'gini', 'max_depth': 2, 'max_features': 2, 'min_samples_leaf': 5, 'n_estimators': 500, 'n_jobs': -1, 'random_state': 90}	5	16	14	3	1	2	8	7	15	11	0	10	12	9	6	4
91	RF, PI	{'criterion': 'gini', 'max_depth': 2, 'max_features': 2, 'min_samples_leaf': 5, 'n_estimators': 500, 'n_jobs': -1, 'random_state': 91}	5	14	15	2	3	1	6	8	16	9	0	11	12	13	4	7
92	RF, PI	{'criterion': 'gini', 'max_depth': 2, 'max_features': 2, 'min_samples_leaf': 5, 'n_estimators': 500, 'n_jobs': -1, 'random_state': 92}	3	16	15	4	0	2	10	12	14	5	1	8	6	11	9	7
93	RF, PI	{'criterion': 'gini', 'max_depth': 2, 'max_features': 2, 'min_samples_leaf': 5, 'n_estimators': 500, 'n_jobs': -1, 'random_state': 93}	4	13	14	0	3	2	9	10	12	7	1	15	11	8	6	5
94	RF, PI	{'criterion': 'gini', 'max_depth': 2, 'max_features': 2, 'min_samples_leaf': 5, 'n_estimators': 500, 'n_jobs': -1, 'random_state': 94}	6	16	9	0	3	2	14	7	13	12	1	15	10	8	5	4
95	RF, PI	{'criterion': 'gini', 'max_depth': 2, 'max_features': 2, 'min_samples_leaf': 5, 'n_estimators': 500, 'n_jobs': -1, 'random_state': 95}	4	11	15	3	1	2	7	5	14	9	0	10	12	13	6	8

96	RF, PI	{'criterion': 'gini', 'max_depth': 2, 'max_features': 2, 'min_samples_leaf': 5, 'n_estimators': 500, 'n_jobs': -1, 'random_state': 96}	4	16	15	2	1	3	14	7	11	6	0	10	12	9	8	5
97	RF, PI	{'criterion': 'gini', 'max_depth': 2, 'max_features': 2, 'min_samples_leaf': 5, 'n_estimators': 500, 'n_jobs': -1, 'random_state': 97}	4	16	13	3	2	1	8	9	14	15	0	10	6	11	5	7
98	RF, PI	{'criterion': 'gini', 'max_depth': 2, 'max_features': 2, 'min_samples_leaf': 5, 'n_estimators': 500, 'n_jobs': -1, 'random_state': 98}	8	16	14	2	1	3	13	7	10	4	0	15	12	9	5	6
99	RF, PI	{'criterion': 'gini', 'max_depth': 2, 'max_features': 2, 'min_samples_leaf': 5, 'n_estimators': 500, 'n_jobs': -1, 'random_state': 99}	4	16	15	3	1	5	14	7	13	10	0	11	8	9	6	2
0	XGB, PI	{'colsample_bytree': 0.6, 'gamma': 0.5, 'max_depth': 2, 'min_child_weight': 5, 'random_state': 0, 'subsample': 0.6}	0	16	6	2	5	3	1	9	14	8	7	11	10	12	4	13
1	XGB, PI	{'colsample_bytree': 0.6, 'gamma': 0.5, 'max_depth': 2, 'min_child_weight': 5, 'random_state': 1, 'subsample': 0.6}	0	14	3	5	4	6	2	12	13	7	10	15	8	11	1	9
2	XGB, PI	{'colsample_bytree': 0.6, 'gamma': 0.5, 'max_depth': 2, 'min_child_weight': 5, 'random_state': 2, 'subsample': 0.6}	0	12	8	1	6	3	4	11	16	7	9	15	5	13	2	10
3	XGB, PI	{'colsample_bytree': 0.6, 'gamma': 0.5, 'max_depth': 2, 'min_child_weight': 5, 'random_state': 3, 'subsample': 0.6}	0	15	9	1	8	2	5	7	16	6	4	12	11	13	3	10
4	XGB, PI	{'colsample_bytree': 0.6, 'gamma': 0.5, 'max_depth': 2, 'min_child_weight': 5, 'random_state': 4, 'subsample': 0.6}	0	14	7	2	3	4	6	13	16	9	8	10	5	15	1	11
5	XGB, PI	{'colsample_bytree': 0.6, 'gamma': 0.5, 'max_depth': 2, 'min_child_weight': 5, 'random_state': 5, 'subsample': 0.6}	1	12	9	0	4	3	5	10	14	8	7	13	6	11	2	16
6	XGB, PI	{'colsample_bytree': 0.6, 'gamma': 0.5, 'max_depth': 2, 'min_child_weight': 5, 'random_state': 6, 'subsample': 0.6}	0	14	6	1	3	5	7	8	16	9	2	12	10	13	4	11
7	XGB, PI	{'colsample_bytree': 0.6, 'gamma': 0.5, 'max_depth': 2, 'min_child_weight': 5, 'random_state': 7, 'subsample': 0.6}	0	13	6	1	4	5	7	10	15	9	11	16	3	14	2	8
8	XGB, PI	{'colsample_bytree': 0.6, 'gamma': 0.5, 'max_depth': 2, 'min_child_weight': 5, 'random_state': 8, 'subsample': 0.6}	1	15	7	0	3	6	2	11	13	12	5	10	8	9	4	14

9	XGB, PI	{'colsample_bytree': 0.6, 'gamma': 0.5, 'max_depth': 2, 'min_child_weight': 5, 'random_state': 9, 'subsample': 0.6}	1	15	9	0	8	7	4	10	14	5	3	11	6	13	2	16
10	XGB, PI	{'colsample_bytree': 0.6, 'gamma': 0.5, 'max_depth': 2, 'min_child_weight': 5, 'random_state': 10, 'subsample': 0.6}	0	14	8	1	5	4	3	9	11	10	6	13	7	12	2	16
11	XGB, PI	{'colsample_bytree': 0.6, 'gamma': 0.5, 'max_depth': 2, 'min_child_weight': 5, 'random_state': 11, 'subsample': 0.6}	1	13	10	0	6	5	4	8	14	9	3	15	7	11	2	12
12	XGB, PI	{'colsample_bytree': 0.6, 'gamma': 0.5, 'max_depth': 2, 'min_child_weight': 5, 'random_state': 12, 'subsample': 0.6}	0	11	9	1	5	3	4	7	15	8	12	6	10	13	2	14
13	XGB, PI	{'colsample_bytree': 0.6, 'gamma': 0.5, 'max_depth': 2, 'min_child_weight': 5, 'random_state': 13, 'subsample': 0.6}	2	11	8	1	3	4	5	10	16	9	6	13	7	12	0	14
14	XGB, PI	{'colsample_bytree': 0.6, 'gamma': 0.5, 'max_depth': 2, 'min_child_weight': 5, 'random_state': 14, 'subsample': 0.6}	0	15	5	1	7	8	3	11	14	9	4	16	6	10	2	13
15	XGB, PI	{'colsample_bytree': 0.6, 'gamma': 0.5, 'max_depth': 2, 'min_child_weight': 5, 'random_state': 15, 'subsample': 0.6}	1	12	4	0	5	7	6	10	14	8	9	15	3	11	2	16
16	XGB, PI	{'colsample_bytree': 0.6, 'gamma': 0.5, 'max_depth': 2, 'min_child_weight': 5, 'random_state': 16, 'subsample': 0.6}	0	14	4	1	6	5	3	12	13	9	7	11	10	15	2	8
17	XGB, PI	{'colsample_bytree': 0.6, 'gamma': 0.5, 'max_depth': 2, 'min_child_weight': 5, 'random_state': 17, 'subsample': 0.6}	0	16	6	2	3	4	7	9	14	10	5	8	13	12	1	11
18	XGB, PI	{'colsample_bytree': 0.6, 'gamma': 0.5, 'max_depth': 2, 'min_child_weight': 5, 'random_state': 18, 'subsample': 0.6}	0	14	9	2	4	6	3	8	15	5	10	12	7	13	1	11
19	XGB, PI	{'colsample_bytree': 0.6, 'gamma': 0.5, 'max_depth': 2, 'min_child_weight': 5, 'random_state': 19, 'subsample': 0.6}	0	11	7	1	6	5	3	12	13	9	4	8	14	15	2	10
20	XGB, PI	{'colsample_bytree': 0.6, 'gamma': 0.5, 'max_depth': 2, 'min_child_weight': 5, 'random_state': 20, 'subsample': 0.6}	0	11	9	2	5	3	4	10	13	8	7	14	6	16	1	15
21	XGB, PI	{'colsample_bytree': 0.6, 'gamma': 0.5, 'max_depth': 2, 'min_child_weight': 5, 'random_state': 21, 'subsample': 0.6}	3	14	6	2	8	0	4	10	15	7	5	13	9	12	1	11
22	XGB, PI	{'colsample_bytree': 0.6, 'gamma': 0.5, 'max_depth': 2, 'min_child_weight': 5, 'random_state': 22, 'subsample': 0.6}	1	13	6	2	5	8	4	9	14	16	7	11	3	15	0	10
23	XGB, PI	{'colsample_bytree': 0.6, 'gamma': 0.5, 'max_depth': 2, 'min_child_weight': 5, 'random_state': 23, 'subsample': 0.6}	0	16	6	1	4	3	7	10	12	8	5	11	9	14	2	15

24	XGB, PI	{'colsample_bytree': 0.6, 'gamma': 0.5, 'max_depth': 2, 'min_child_weight': 5, 'random_state': 24, 'subsample': 0.6}	0	11	4	1	5	3	6	9	15	7	8	12	10	14	2	13
25	XGB, PI	{'colsample_bytree': 0.6, 'gamma': 0.5, 'max_depth': 2, 'min_child_weight': 5, 'random_state': 25, 'subsample': 0.6}	1	10	12	0	4	6	5	9	11	7	8	13	2	16	3	14
26	XGB, PI	{'colsample_bytree': 0.6, 'gamma': 0.5, 'max_depth': 2, 'min_child_weight': 5, 'random_state': 26, 'subsample': 0.6}	0	16	9	1	3	5	4	15	14	7	8	12	6	10	2	11
27	XGB, PI	{'colsample_bytree': 0.6, 'gamma': 0.5, 'max_depth': 2, 'min_child_weight': 5, 'random_state': 27, 'subsample': 0.6}	0	11	7	1	5	3	4	9	14	6	8	12	13	16	2	10
28	XGB, PI	{'colsample_bytree': 0.6, 'gamma': 0.5, 'max_depth': 2, 'min_child_weight': 5, 'random_state': 28, 'subsample': 0.6}	3	14	9	1	2	6	5	10	15	8	4	12	7	13	0	11
29	XGB, PI	{'colsample_bytree': 0.6, 'gamma': 0.5, 'max_depth': 2, 'min_child_weight': 5, 'random_state': 29, 'subsample': 0.6}	0	14	6	3	4	7	2	8	13	10	5	11	9	12	1	16
30	XGB, PI	{'colsample_bytree': 0.6, 'gamma': 0.5, 'max_depth': 2, 'min_child_weight': 5, 'random_state': 30, 'subsample': 0.6}	1	13	6	0	4	5	3	9	15	10	7	8	14	11	2	12
31	XGB, PI	{'colsample_bytree': 0.6, 'gamma': 0.5, 'max_depth': 2, 'min_child_weight': 5, 'random_state': 31, 'subsample': 0.6}	0	13	4	3	6	5	2	9	14	10	7	16	8	11	1	12
32	XGB, PI	{'colsample_bytree': 0.6, 'gamma': 0.5, 'max_depth': 2, 'min_child_weight': 5, 'random_state': 32, 'subsample': 0.6}	0	14	8	1	3	5	2	11	13	9	6	16	7	10	4	12
33	XGB, PI	{'colsample_bytree': 0.6, 'gamma': 0.5, 'max_depth': 2, 'min_child_weight': 5, 'random_state': 33, 'subsample': 0.6}	0	13	7	1	5	4	2	10	14	11	6	12	8	15	3	9
34	XGB, PI	{'colsample_bytree': 0.6, 'gamma': 0.5, 'max_depth': 2, 'min_child_weight': 5, 'random_state': 34, 'subsample': 0.6}	1	14	6	5	4	3	7	12	16	10	0	15	8	11	2	13
35	XGB, PI	{'colsample_bytree': 0.6, 'gamma': 0.5, 'max_depth': 2, 'min_child_weight': 5, 'random_state': 35, 'subsample': 0.6}	0	16	6	2	3	5	4	9	13	10	8	11	7	14	1	12
36	XGB, PI	{'colsample_bytree': 0.6, 'gamma': 0.5, 'max_depth': 2, 'min_child_weight': 5, 'random_state': 36, 'subsample': 0.6}	1	14	9	0	4	5	3	8	16	10	6	11	7	13	2	12
37	XGB, PI	{'colsample_bytree': 0.6, 'gamma': 0.5, 'max_depth': 2, 'min_child_weight': 5, 'random_state': 37, 'subsample': 0.6}	1	12	7	0	4	3	6	9	16	10	2	11	8	14	5	13
38	XGB, PI	{'colsample_bytree': 0.6, 'gamma': 0.5, 'max_depth': 2, 'min_child_weight': 5, 'random_state': 38, 'subsample': 0.6}	0	14	7	1	5	4	3	11	16	9	8	10	6	15	2	12

39	XGB, PI	{'colsample_bytree': 0.6, 'gamma': 0.5, 'max_depth': 2, 'min_child_weight': 5, 'random_state': 39, 'subsample': 0.6}	1	11	7	0	6	4	3	10	14	8	5	12	9	13	2	15
40	XGB, PI	{'colsample_bytree': 0.6, 'gamma': 0.5, 'max_depth': 2, 'min_child_weight': 5, 'random_state': 40, 'subsample': 0.6}	0	12	8	2	4	3	5	11	14	6	10	9	7	16	1	13
41	XGB, PI	{'colsample_bytree': 0.6, 'gamma': 0.5, 'max_depth': 2, 'min_child_weight': 5, 'random_state': 41, 'subsample': 0.6}	2	15	6	1	7	4	3	10	16	8	5	11	9	14	0	12
42	XGB, PI	{'colsample_bytree': 0.6, 'gamma': 0.5, 'max_depth': 2, 'min_child_weight': 5, 'random_state': 42, 'subsample': 0.6}	0	12	3	1	8	4	6	10	15	9	7	14	5	11	2	13
43	XGB, PI	{'colsample_bytree': 0.6, 'gamma': 0.5, 'max_depth': 2, 'min_child_weight': 5, 'random_state': 43, 'subsample': 0.6}	0	13	7	2	4	6	5	11	15	8	9	16	3	10	1	14
44	XGB, PI	{'colsample_bytree': 0.6, 'gamma': 0.5, 'max_depth': 2, 'min_child_weight': 5, 'random_state': 44, 'subsample': 0.6}	1	16	6	0	5	7	3	12	14	9	4	10	11	8	2	13
45	XGB, PI	{'colsample_bytree': 0.6, 'gamma': 0.5, 'max_depth': 2, 'min_child_weight': 5, 'random_state': 45, 'subsample': 0.6}	5	13	9	0	4	2	8	7	16	10	6	12	3	11	1	15
46	XGB, PI	{'colsample_bytree': 0.6, 'gamma': 0.5, 'max_depth': 2, 'min_child_weight': 5, 'random_state': 46, 'subsample': 0.6}	2	13	7	1	6	5	3	9	15	11	4	10	8	16	0	14
47	XGB, PI	{'colsample_bytree': 0.6, 'gamma': 0.5, 'max_depth': 2, 'min_child_weight': 5, 'random_state': 47, 'subsample': 0.6}	2	13	6	0	3	7	4	10	15	11	8	14	5	12	1	9
48	XGB, PI	{'colsample_bytree': 0.6, 'gamma': 0.5, 'max_depth': 2, 'min_child_weight': 5, 'random_state': 48, 'subsample': 0.6}	2	15	6	0	8	3	4	10	16	9	7	14	5	12	1	11
49	XGB, PI	{'colsample_bytree': 0.6, 'gamma': 0.5, 'max_depth': 2, 'min_child_weight': 5, 'random_state': 49, 'subsample': 0.6}	1	10	11	0	5	3	4	8	14	7	6	15	9	13	2	12
50	XGB, PI	{'colsample_bytree': 0.6, 'gamma': 0.5, 'max_depth': 2, 'min_child_weight': 5, 'random_state': 50, 'subsample': 0.6}	1	14	7	4	9	5	0	10	11	8	6	15	2	12	3	13
51	XGB, PI	{'colsample_bytree': 0.6, 'gamma': 0.5, 'max_depth': 2, 'min_child_weight': 5, 'random_state': 51, 'subsample': 0.6}	1	14	9	0	4	8	5	6	13	11	3	12	10	16	2	7
52	XGB, PI	{'colsample_bytree': 0.6, 'gamma': 0.5, 'max_depth': 2, 'min_child_weight': 5, 'random_state': 52, 'subsample': 0.6}	0	15	7	2	5	3	6	10	13	9	1	14	8	11	4	12
53	XGB, PI	{'colsample_bytree': 0.6, 'gamma': 0.5, 'max_depth': 2, 'min_child_weight': 5, 'random_state': 53, 'subsample': 0.6}	1	13	8	0	7	4	5	11	16	9	3	12	6	10	2	15

54	XGB, PI	{'colsample_bytree': 0.6, 'gamma': 0.5, 'max_depth': 2, 'min_child_weight': 5, 'random_state': 54, 'subsample': 0.6}	1	13	8	0	4	5	3	7	15	10	6	16	9	11	2	14
55	XGB, PI	{'colsample_bytree': 0.6, 'gamma': 0.5, 'max_depth': 2, 'min_child_weight': 5, 'random_state': 55, 'subsample': 0.6}	2	13	7	5	1	4	3	9	12	10	6	11	8	14	0	16
56	XGB, PI	{'colsample_bytree': 0.6, 'gamma': 0.5, 'max_depth': 2, 'min_child_weight': 5, 'random_state': 56, 'subsample': 0.6}	5	16	9	1	3	4	6	10	12	7	2	11	8	13	0	15
57	XGB, PI	{'colsample_bytree': 0.6, 'gamma': 0.5, 'max_depth': 2, 'min_child_weight': 5, 'random_state': 57, 'subsample': 0.6}	0	12	9	3	4	5	6	10	16	8	7	11	1	14	2	13
58	XGB, PI	{'colsample_bytree': 0.6, 'gamma': 0.5, 'max_depth': 2, 'min_child_weight': 5, 'random_state': 58, 'subsample': 0.6}	0	13	10	1	4	3	5	9	14	7	6	15	8	11	2	16
59	XGB, PI	{'colsample_bytree': 0.6, 'gamma': 0.5, 'max_depth': 2, 'min_child_weight': 5, 'random_state': 59, 'subsample': 0.6}	0	13	9	2	5	1	4	6	14	10	7	11	8	12	3	15
60	XGB, PI	{'colsample_bytree': 0.6, 'gamma': 0.5, 'max_depth': 2, 'min_child_weight': 5, 'random_state': 60, 'subsample': 0.6}	1	11	7	3	4	6	2	9	16	5	10	13	8	14	0	12
61	XGB, PI	{'colsample_bytree': 0.6, 'gamma': 0.5, 'max_depth': 2, 'min_child_weight': 5, 'random_state': 61, 'subsample': 0.6}	0	13	3	1	4	6	5	11	14	9	7	15	8	16	2	10
62	XGB, PI	{'colsample_bytree': 0.6, 'gamma': 0.5, 'max_depth': 2, 'min_child_weight': 5, 'random_state': 62, 'subsample': 0.6}	0	14	7	1	5	4	3	13	15	8	6	10	9	11	2	12
63	XGB, PI	{'colsample_bytree': 0.6, 'gamma': 0.5, 'max_depth': 2, 'min_child_weight': 5, 'random_state': 63, 'subsample': 0.6}	1	14	8	0	7	4	3	6	16	9	5	11	10	12	2	15
64	XGB, PI	{'colsample_bytree': 0.6, 'gamma': 0.5, 'max_depth': 2, 'min_child_weight': 5, 'random_state': 64, 'subsample': 0.6}	1	13	8	0	5	4	6	11	12	7	9	15	3	14	2	10
65	XGB, PI	{'colsample_bytree': 0.6, 'gamma': 0.5, 'max_depth': 2, 'min_child_weight': 5, 'random_state': 65, 'subsample': 0.6}	0	13	8	1	4	6	7	11	15	9	2	10	5	16	3	14
66	XGB, PI	{'colsample_bytree': 0.6, 'gamma': 0.5, 'max_depth': 2, 'min_child_weight': 5, 'random_state': 66, 'subsample': 0.6}	0	11	6	1	4	7	2	8	15	10	3	9	13	14	5	12
67	XGB, PI	{'colsample_bytree': 0.6, 'gamma': 0.5, 'max_depth': 2, 'min_child_weight': 5, 'random_state': 67, 'subsample': 0.6}	0	12	8	2	6	3	5	11	15	7	9	13	4	16	1	14
68	XGB, PI	{'colsample_bytree': 0.6, 'gamma': 0.5, 'max_depth': 2, 'min_child_weight': 5, 'random_state': 68, 'subsample': 0.6}	1	12	7	0	5	6	4	10	14	9	3	8	11	15	2	16

69	XGB, PI	{'colsample_bytree': 0.6, 'gamma': 0.5, 'max_depth': 2, 'min_child_weight': 5, 'random_state': 69, 'subsample': 0.6}	0	12	6	1	5	4	2	8	15	9	10	16	7	13	3	11
70	XGB, PI	{'colsample_bytree': 0.6, 'gamma': 0.5, 'max_depth': 2, 'min_child_weight': 5, 'random_state': 70, 'subsample': 0.6}	1	16	7	3	6	2	4	12	11	8	5	9	10	13	0	14
71	XGB, PI	{'colsample_bytree': 0.6, 'gamma': 0.5, 'max_depth': 2, 'min_child_weight': 5, 'random_state': 71, 'subsample': 0.6}	1	13	8	0	4	5	7	9	16	10	3	11	6	14	2	12
72	XGB, PI	{'colsample_bytree': 0.6, 'gamma': 0.5, 'max_depth': 2, 'min_child_weight': 5, 'random_state': 72, 'subsample': 0.6}	0	13	5	1	6	2	4	8	16	9	7	14	10	12	3	11
73	XGB, PI	{'colsample_bytree': 0.6, 'gamma': 0.5, 'max_depth': 2, 'min_child_weight': 5, 'random_state': 73, 'subsample': 0.6}	1	14	8	6	3	5	2	9	11	10	4	12	7	16	0	15
74	XGB, PI	{'colsample_bytree': 0.6, 'gamma': 0.5, 'max_depth': 2, 'min_child_weight': 5, 'random_state': 74, 'subsample': 0.6}	0	12	7	2	1	6	4	11	10	9	5	13	8	14	3	16
75	XGB, PI	{'colsample_bytree': 0.6, 'gamma': 0.5, 'max_depth': 2, 'min_child_weight': 5, 'random_state': 75, 'subsample': 0.6}	1	15	5	0	4	6	8	10	16	9	3	14	7	11	2	12
76	XGB, PI	{'colsample_bytree': 0.6, 'gamma': 0.5, 'max_depth': 2, 'min_child_weight': 5, 'random_state': 76, 'subsample': 0.6}	0	12	9	1	2	4	6	10	14	8	7	16	3	11	5	13
77	XGB, PI	{'colsample_bytree': 0.6, 'gamma': 0.5, 'max_depth': 2, 'min_child_weight': 5, 'random_state': 77, 'subsample': 0.6}	3	16	8	2	6	4	5	10	14	9	1	15	7	12	0	11
78	XGB, PI	{'colsample_bytree': 0.6, 'gamma': 0.5, 'max_depth': 2, 'min_child_weight': 5, 'random_state': 78, 'subsample': 0.6}	0	15	5	2	9	4	6	10	16	7	3	14	8	11	1	12
79	XGB, PI	{'colsample_bytree': 0.6, 'gamma': 0.5, 'max_depth': 2, 'min_child_weight': 5, 'random_state': 79, 'subsample': 0.6}	1	15	6	2	4	5	3	10	13	9	8	16	7	14	0	11
80	XGB, PI	{'colsample_bytree': 0.6, 'gamma': 0.5, 'max_depth': 2, 'min_child_weight': 5, 'random_state': 80, 'subsample': 0.6}	0	14	7	2	3	5	6	10	15	8	4	13	9	12	1	11
81	XGB, PI	{'colsample_bytree': 0.6, 'gamma': 0.5, 'max_depth': 2, 'min_child_weight': 5, 'random_state': 81, 'subsample': 0.6}	0	14	9	1	5	2	4	8	16	3	7	10	12	13	6	11
82	XGB, PI	{'colsample_bytree': 0.6, 'gamma': 0.5, 'max_depth': 2, 'min_child_weight': 5, 'random_state': 82, 'subsample': 0.6}	1	14	10	2	3	4	6	9	13	12	7	8	5	16	0	11
83	XGB, PI	{'colsample_bytree': 0.6, 'gamma': 0.5, 'max_depth': 2, 'min_child_weight': 5, 'random_state': 83, 'subsample': 0.6}	0	16	5	1	8	4	3	10	12	6	11	9	7	14	2	15

84	XGB, PI	{'colsample_bytree': 0.6, 'gamma': 0.5, 'max_depth': 2, 'min_child_weight': 5, 'random_state': 84, 'subsample': 0.6}	0	16	8	1	6	3	4	10	14	7	5	9	11	12	2	13
85	XGB, PI	{'colsample_bytree': 0.6, 'gamma': 0.5, 'max_depth': 2, 'min_child_weight': 5, 'random_state': 85, 'subsample': 0.6}	2	10	8	1	3	4	5	12	13	7	6	9	11	15	0	16
86	XGB, PI	{'colsample_bytree': 0.6, 'gamma': 0.5, 'max_depth': 2, 'min_child_weight': 5, 'random_state': 86, 'subsample': 0.6}	0	15	6	1	7	4	5	9	14	12	3	13	8	11	2	10
87	XGB, PI	{'colsample_bytree': 0.6, 'gamma': 0.5, 'max_depth': 2, 'min_child_weight': 5, 'random_state': 87, 'subsample': 0.6}	0	15	6	2	9	5	4	8	16	7	3	12	11	13	1	10
88	XGB, PI	{'colsample_bytree': 0.6, 'gamma': 0.5, 'max_depth': 2, 'min_child_weight': 5, 'random_state': 88, 'subsample': 0.6}	0	16	8	1	5	3	4	11	15	9	7	12	6	14	2	13
89	XGB, PI	{'colsample_bytree': 0.6, 'gamma': 0.5, 'max_depth': 2, 'min_child_weight': 5, 'random_state': 89, 'subsample': 0.6}	0	15	6	1	5	7	4	10	13	8	9	14	3	12	2	11
90	XGB, PI	{'colsample_bytree': 0.6, 'gamma': 0.5, 'max_depth': 2, 'min_child_weight': 5, 'random_state': 90, 'subsample': 0.6}	0	14	6	1	3	9	5	8	16	10	4	7	11	13	2	12
91	XGB, PI	{'colsample_bytree': 0.6, 'gamma': 0.5, 'max_depth': 2, 'min_child_weight': 5, 'random_state': 91, 'subsample': 0.6}	0	16	8	1	5	3	4	9	14	12	6	11	7	13	2	10
92	XGB, PI	{'colsample_bytree': 0.6, 'gamma': 0.5, 'max_depth': 2, 'min_child_weight': 5, 'random_state': 92, 'subsample': 0.6}	1	15	2	5	7	4	3	10	16	9	6	12	8	11	0	14
93	XGB, PI	{'colsample_bytree': 0.6, 'gamma': 0.5, 'max_depth': 2, 'min_child_weight': 5, 'random_state': 93, 'subsample': 0.6}	1	12	7	0	5	6	4	9	16	8	3	11	10	14	2	13
94	XGB, PI	{'colsample_bytree': 0.6, 'gamma': 0.5, 'max_depth': 2, 'min_child_weight': 5, 'random_state': 94, 'subsample': 0.6}	0	16	4	1	2	5	3	8	15	12	6	10	9	11	7	13
95	XGB, PI	{'colsample_bytree': 0.6, 'gamma': 0.5, 'max_depth': 2, 'min_child_weight': 5, 'random_state': 95, 'subsample': 0.6}	1	9	11	0	4	6	5	8	16	7	3	13	10	12	2	15
96	XGB, PI	{'colsample_bytree': 0.6, 'gamma': 0.5, 'max_depth': 2, 'min_child_weight': 5, 'random_state': 96, 'subsample': 0.6}	0	10	7	3	5	6	4	15	14	8	2	12	9	16	1	11
97	XGB, PI	{'colsample_bytree': 0.6, 'gamma': 0.5, 'max_depth': 2, 'min_child_weight': 5, 'random_state': 97, 'subsample': 0.6}	1	15	8	0	5	6	2	9	14	7	4	12	10	11	3	16
98	XGB, PI	{'colsample_bytree': 0.6, 'gamma': 0.5, 'max_depth': 2, 'min_child_weight': 5, 'random_state': 98, 'subsample': 0.6}	1	12	9	0	5	4	3	8	13	10	6	7	14	16	2	11

99	XGB, PI	{'colsample_bytree': 0.6, 'gamma': 0.5, 'max_depth': 2, 'min_child_weight': 5, 'random_state': 99, 'subsample': 0.6}	0	12	10	2	4	3	6	8	16	9	7	11	5	13	1	15
0	XGB, RFE	{'colsample_bytree': 0.6, 'gamma': 0.5, 'max_depth': 2, 'min_child_weight': 5, 'random_state': 0, 'subsample': 0.6}	4	17	11	12	10	8	2	14	15	9	1	13	6	16	3	7
1	XGB, RFE	{'colsample_bytree': 0.6, 'gamma': 0.5, 'max_depth': 2, 'min_child_weight': 5, 'random_state': 1, 'subsample': 0.6}	1	15	13	4	9	16	12	10	14	3	8	11	5	17	6	2
2	XGB, RFE	{'colsample_bytree': 0.6, 'gamma': 0.5, 'max_depth': 2, 'min_child_weight': 5, 'random_state': 2, 'subsample': 0.6}	12	13	6	8	9	4	7	5	17	11	2	16	10	15	3	1
3	XGB, RFE	{'colsample_bytree': 0.6, 'gamma': 0.5, 'max_depth': 2, 'min_child_weight': 5, 'random_state': 3, 'subsample': 0.6}	3	17	8	4	12	7	10	5	6	14	13	9	11	15	1	2
4	XGB, RFE	{'colsample_bytree': 0.6, 'gamma': 0.5, 'max_depth': 2, 'min_child_weight': 5, 'random_state': 4, 'subsample': 0.6}	1	14	6	5	12	3	11	10	17	15	8	7	13	16	9	4
5	XGB, RFE	{'colsample_bytree': 0.6, 'gamma': 0.5, 'max_depth': 2, 'min_child_weight': 5, 'random_state': 5, 'subsample': 0.6}	4	12	15	10	14	2	6	9	13	1	5	11	7	17	8	16
6	XGB, RFE	{'colsample_bytree': 0.6, 'gamma': 0.5, 'max_depth': 2, 'min_child_weight': 5, 'random_state': 6, 'subsample': 0.6}	7	13	16	8	3	6	11	10	4	1	5	14	9	15	12	2
7	XGB, RFE	{'colsample_bytree': 0.6, 'gamma': 0.5, 'max_depth': 2, 'min_child_weight': 5, 'random_state': 7, 'subsample': 0.6}	3	8	14	2	15	6	9	5	16	10	7	17	4	11	12	1
8	XGB, RFE	{'colsample_bytree': 0.6, 'gamma': 0.5, 'max_depth': 2, 'min_child_weight': 5, 'random_state': 8, 'subsample': 0.6}	4	14	15	6	5	7	9	17	12	10	1	2	8	13	16	3
9	XGB, RFE	{'colsample_bytree': 0.6, 'gamma': 0.5, 'max_depth': 2, 'min_child_weight': 5, 'random_state': 9, 'subsample': 0.6}	7	17	11	12	5	13	10	6	8	15	2	1	14	16	4	3
10	XGB, RFE	{'colsample_bytree': 0.6, 'gamma': 0.5, 'max_depth': 2, 'min_child_weight': 5, 'random_state': 10, 'subsample': 0.6}	8	16	11	1	12	7	4	6	13	15	3	14	9	17	5	2
11	XGB, RFE	{'colsample_bytree': 0.6, 'gamma': 0.5, 'max_depth': 2, 'min_child_weight': 5, 'random_state': 11, 'subsample': 0.6}	2	9	14	5	8	12	11	4	16	17	1	7	13	10	6	3
12	XGB, RFE	{'colsample_bytree': 0.6, 'gamma': 0.5, 'max_depth': 2, 'min_child_weight': 5, 'random_state': 12, 'subsample': 0.6}	1	17	7	3	4	9	8	15	14	12	11	10	5	13	6	2
13	XGB, RFE	{'colsample_bytree': 0.6, 'gamma': 0.5, 'max_depth': 2, 'min_child_weight': 5, 'random_state': 13, 'subsample': 0.6}	11	16	13	4	3	8	5	9	17	12	2	1	6	14	7	15

14	XGB, RFE	{'colsample_bytree': 0.6, 'gamma': 0.5, 'max_depth': 2, 'min_child_weight': 5, 'random_state': 14, 'subsample': 0.6}	9	14	12	4	2	5	11	10	13	17	6	16	1	15	8	3
15	XGB, RFE	{'colsample_bytree': 0.6, 'gamma': 0.5, 'max_depth': 2, 'min_child_weight': 5, 'random_state': 15, 'subsample': 0.6}	7	10	15	6	9	3	13	8	11	12	4	14	2	16	1	17
16	XGB, RFE	{'colsample_bytree': 0.6, 'gamma': 0.5, 'max_depth': 2, 'min_child_weight': 5, 'random_state': 16, 'subsample': 0.6}	4	16	5	3	9	10	13	6	15	17	8	7	1	14	12	2
17	XGB, RFE	{'colsample_bytree': 0.6, 'gamma': 0.5, 'max_depth': 2, 'min_child_weight': 5, 'random_state': 17, 'subsample': 0.6}	1	17	15	10	2	3	7	6	13	14	4	16	8	12	5	11
18	XGB, RFE	{'colsample_bytree': 0.6, 'gamma': 0.5, 'max_depth': 2, 'min_child_weight': 5, 'random_state': 18, 'subsample': 0.6}	1	16	12	6	5	4	2	14	15	10	8	13	11	17	9	3
19	XGB, RFE	{'colsample_bytree': 0.6, 'gamma': 0.5, 'max_depth': 2, 'min_child_weight': 5, 'random_state': 19, 'subsample': 0.6}	15	17	14	5	3	6	4	9	10	8	2	13	7	12	11	1
20	XGB, RFE	{'colsample_bytree': 0.6, 'gamma': 0.5, 'max_depth': 2, 'min_child_weight': 5, 'random_state': 20, 'subsample': 0.6}	3	14	8	6	4	12	10	7	16	11	2	15	9	17	5	1
21	XGB, RFE	{'colsample_bytree': 0.6, 'gamma': 0.5, 'max_depth': 2, 'min_child_weight': 5, 'random_state': 21, 'subsample': 0.6}	4	12	15	2	7	10	6	8	16	11	1	5	9	14	13	3
22	XGB, RFE	{'colsample_bytree': 0.6, 'gamma': 0.5, 'max_depth': 2, 'min_child_weight': 5, 'random_state': 22, 'subsample': 0.6}	1	13	15	3	4	5	11	16	14	12	7	9	8	17	10	2
23	XGB, RFE	{'colsample_bytree': 0.6, 'gamma': 0.5, 'max_depth': 2, 'min_child_weight': 5, 'random_state': 23, 'subsample': 0.6}	1	16	13	8	5	11	9	6	15	12	4	3	7	17	10	2
24	XGB, RFE	{'colsample_bytree': 0.6, 'gamma': 0.5, 'max_depth': 2, 'min_child_weight': 5, 'random_state': 24, 'subsample': 0.6}	7	15	14	5	8	1	10	11	17	3	2	12	4	13	9	16
25	XGB, RFE	{'colsample_bytree': 0.6, 'gamma': 0.5, 'max_depth': 2, 'min_child_weight': 5, 'random_state': 25, 'subsample': 0.6}	1	15	11	10	2	8	12	7	16	6	9	14	5	17	4	3
26	XGB, RFE	{'colsample_bytree': 0.6, 'gamma': 0.5, 'max_depth': 2, 'min_child_weight': 5, 'random_state': 26, 'subsample': 0.6}	1	17	9	3	5	10	4	13	15	8	6	14	7	11	12	2
27	XGB, RFE	{'colsample_bytree': 0.6, 'gamma': 0.5, 'max_depth': 2, 'min_child_weight': 5, 'random_state': 27, 'subsample': 0.6}	4	12	17	2	15	5	10	9	16	11	6	14	3	13	7	1
28	XGB, RFE	{'colsample_bytree': 0.6, 'gamma': 0.5, 'max_depth': 2, 'min_child_weight': 5, 'random_state': 28, 'subsample': 0.6}	3	17	16	2	7	10	4	15	13	1	8	11	6	14	9	5

29	XGB, RFE	{'colsample_bytree': 0.6, 'gamma': 0.5, 'max_depth': 2, 'min_child_weight': 5, 'random_state': 29, 'subsample': 0.6}	1	14	9	2	4	16	6	10	17	8	12	13	5	15	7	3
30	XGB, RFE	{'colsample_bytree': 0.6, 'gamma': 0.5, 'max_depth': 2, 'min_child_weight': 5, 'random_state': 30, 'subsample': 0.6}	3	14	12	10	7	9	6	8	11	5	1	2	15	16	4	13
31	XGB, RFE	{'colsample_bytree': 0.6, 'gamma': 0.5, 'max_depth': 2, 'min_child_weight': 5, 'random_state': 31, 'subsample': 0.6}	10	15	7	9	8	13	1	3	12	16	4	17	5	11	6	2
32	XGB, RFE	{'colsample_bytree': 0.6, 'gamma': 0.5, 'max_depth': 2, 'min_child_weight': 5, 'random_state': 32, 'subsample': 0.6}	3	17	15	5	1	8	10	13	14	9	4	7	6	12	11	2
33	XGB, RFE	{'colsample_bytree': 0.6, 'gamma': 0.5, 'max_depth': 2, 'min_child_weight': 5, 'random_state': 33, 'subsample': 0.6}	6	15	13	3	7	12	1	9	10	5	4	11	8	17	14	2
34	XGB, RFE	{'colsample_bytree': 0.6, 'gamma': 0.5, 'max_depth': 2, 'min_child_weight': 5, 'random_state': 34, 'subsample': 0.6}	12	14	6	7	2	15	5	13	17	10	1	8	16	11	3	4
35	XGB, RFE	{'colsample_bytree': 0.6, 'gamma': 0.5, 'max_depth': 2, 'min_child_weight': 5, 'random_state': 35, 'subsample': 0.6}	11	15	17	4	2	7	6	10	16	9	1	5	13	12	14	3
36	XGB, RFE	{'colsample_bytree': 0.6, 'gamma': 0.5, 'max_depth': 2, 'min_child_weight': 5, 'random_state': 36, 'subsample': 0.6}	8	15	13	5	7	10	2	14	17	6	1	16	4	12	3	11
37	XGB, RFE	{'colsample_bytree': 0.6, 'gamma': 0.5, 'max_depth': 2, 'min_child_weight': 5, 'random_state': 37, 'subsample': 0.6}	9	16	13	4	10	5	7	3	17	11	1	15	6	14	12	2
38	XGB, RFE	{'colsample_bytree': 0.6, 'gamma': 0.5, 'max_depth': 2, 'min_child_weight': 5, 'random_state': 38, 'subsample': 0.6}	2	14	15	5	1	9	6	13	16	4	8	11	3	17	10	12
39	XGB, RFE	{'colsample_bytree': 0.6, 'gamma': 0.5, 'max_depth': 2, 'min_child_weight': 5, 'random_state': 39, 'subsample': 0.6}	5	16	12	2	11	7	13	14	15	10	3	9	6	17	4	1
40	XGB, RFE	{'colsample_bytree': 0.6, 'gamma': 0.5, 'max_depth': 2, 'min_child_weight': 5, 'random_state': 40, 'subsample': 0.6}	2	16	5	13	3	10	6	14	15	7	1	4	11	17	9	12
41	XGB, RFE	{'colsample_bytree': 0.6, 'gamma': 0.5, 'max_depth': 2, 'min_child_weight': 5, 'random_state': 41, 'subsample': 0.6}	3	13	10	7	4	12	2	11	15	6	1	5	14	17	9	16
42	XGB, RFE	{'colsample_bytree': 0.6, 'gamma': 0.5, 'max_depth': 2, 'min_child_weight': 5, 'random_state': 42, 'subsample': 0.6}	13	16	15	1	12	5	11	4	14	10	6	3	8	17	7	2
43	XGB, RFE	{'colsample_bytree': 0.6, 'gamma': 0.5, 'max_depth': 2, 'min_child_weight': 5, 'random_state': 43, 'subsample': 0.6}	3	17	13	12	5	10	7	9	4	14	1	16	6	15	8	2

44	XGB, RFE	{'colsample_bytree': 0.6, 'gamma': 0.5, 'max_depth': 2, 'min_child_weight': 5, 'random_state': 44, 'subsample': 0.6}	5	17	10	6	1	13	9	3	16	2	7	4	15	12	11	14
45	XGB, RFE	{'colsample_bytree': 0.6, 'gamma': 0.5, 'max_depth': 2, 'min_child_weight': 5, 'random_state': 45, 'subsample': 0.6}	4	2	14	5	8	10	7	13	16	15	1	17	6	11	9	12
46	XGB, RFE	{'colsample_bytree': 0.6, 'gamma': 0.5, 'max_depth': 2, 'min_child_weight': 5, 'random_state': 46, 'subsample': 0.6}	3	12	17	6	8	5	14	15	16	7	1	9	13	11	4	2
47	XGB, RFE	{'colsample_bytree': 0.6, 'gamma': 0.5, 'max_depth': 2, 'min_child_weight': 5, 'random_state': 47, 'subsample': 0.6}	7	16	11	4	5	10	2	13	17	9	6	1	15	14	8	3
48	XGB, RFE	{'colsample_bytree': 0.6, 'gamma': 0.5, 'max_depth': 2, 'min_child_weight': 5, 'random_state': 48, 'subsample': 0.6}	2	17	12	3	9	14	13	5	8	10	6	11	15	16	7	4
49	XGB, RFE	{'colsample_bytree': 0.6, 'gamma': 0.5, 'max_depth': 2, 'min_child_weight': 5, 'random_state': 49, 'subsample': 0.6}	6	13	16	5	11	7	1	9	14	4	8	12	2	15	10	3
50	XGB, RFE	{'colsample_bytree': 0.6, 'gamma': 0.5, 'max_depth': 2, 'min_child_weight': 5, 'random_state': 50, 'subsample': 0.6}	1	11	5	4	7	12	2	6	16	13	9	17	10	14	15	8
51	XGB, RFE	{'colsample_bytree': 0.6, 'gamma': 0.5, 'max_depth': 2, 'min_child_weight': 5, 'random_state': 51, 'subsample': 0.6}	2	17	10	3	12	4	14	9	5	13	8	16	6	15	7	1
52	XGB, RFE	{'colsample_bytree': 0.6, 'gamma': 0.5, 'max_depth': 2, 'min_child_weight': 5, 'random_state': 52, 'subsample': 0.6}	11	6	13	2	5	8	4	7	12	17	1	14	9	15	10	3
53	XGB, RFE	{'colsample_bytree': 0.6, 'gamma': 0.5, 'max_depth': 2, 'min_child_weight': 5, 'random_state': 53, 'subsample': 0.6}	4	17	11	7	3	15	8	10	16	9	2	12	5	13	6	1
54	XGB, RFE	{'colsample_bytree': 0.6, 'gamma': 0.5, 'max_depth': 2, 'min_child_weight': 5, 'random_state': 54, 'subsample': 0.6}	10	11	13	6	1	3	15	14	17	2	5	9	8	16	4	12
55	XGB, RFE	{'colsample_bytree': 0.6, 'gamma': 0.5, 'max_depth': 2, 'min_child_weight': 5, 'random_state': 55, 'subsample': 0.6}	1	15	10	3	12	9	7	13	14	6	2	8	11	17	5	4
56	XGB, RFE	{'colsample_bytree': 0.6, 'gamma': 0.5, 'max_depth': 2, 'min_child_weight': 5, 'random_state': 56, 'subsample': 0.6}	13	14	8	4	7	11	9	5	15	12	1	16	6	17	3	2
57	XGB, RFE	{'colsample_bytree': 0.6, 'gamma': 0.5, 'max_depth': 2, 'min_child_weight': 5, 'random_state': 57, 'subsample': 0.6}	14	17	13	4	3	5	2	11	15	7	12	6	1	16	10	9
58	XGB, RFE	{'colsample_bytree': 0.6, 'gamma': 0.5, 'max_depth': 2, 'min_child_weight': 5, 'random_state': 58, 'subsample': 0.6}	8	16	13	4	14	3	2	10	12	11	9	6	17	15	7	1

59	XGB, RFE	{'colsample_bytree': 0.6, 'gamma': 0.5, 'max_depth': 2, 'min_child_weight': 5, 'random_state': 59, 'subsample': 0.6}	3	12	11	13	4	6	1	10	14	7	2	9	16	15	8	5
60	XGB, RFE	{'colsample_bytree': 0.6, 'gamma': 0.5, 'max_depth': 2, 'min_child_weight': 5, 'random_state': 60, 'subsample': 0.6}	1	14	9	8	12	7	10	13	15	4	5	16	6	17	3	2
61	XGB, RFE	{'colsample_bytree': 0.6, 'gamma': 0.5, 'max_depth': 2, 'min_child_weight': 5, 'random_state': 61, 'subsample': 0.6}	15	14	16	8	6	12	7	11	5	1	4	13	10	17	2	3
62	XGB, RFE	{'colsample_bytree': 0.6, 'gamma': 0.5, 'max_depth': 2, 'min_child_weight': 5, 'random_state': 62, 'subsample': 0.6}	2	17	12	3	9	10	5	13	15	14	1	16	8	6	7	4
63	XGB, RFE	{'colsample_bytree': 0.6, 'gamma': 0.5, 'max_depth': 2, 'min_child_weight': 5, 'random_state': 63, 'subsample': 0.6}	15	16	7	6	12	5	11	8	17	9	4	10	1	14	3	2
64	XGB, RFE	{'colsample_bytree': 0.6, 'gamma': 0.5, 'max_depth': 2, 'min_child_weight': 5, 'random_state': 64, 'subsample': 0.6}	1	16	10	14	4	5	7	8	12	6	2	9	17	11	15	3
65	XGB, RFE	{'colsample_bytree': 0.6, 'gamma': 0.5, 'max_depth': 2, 'min_child_weight': 5, 'random_state': 65, 'subsample': 0.6}	1	14	10	4	6	7	5	12	15	8	11	9	3	17	16	2
66	XGB, RFE	{'colsample_bytree': 0.6, 'gamma': 0.5, 'max_depth': 2, 'min_child_weight': 5, 'random_state': 66, 'subsample': 0.6}	1	17	15	4	7	13	5	9	11	12	2	10	14	16	8	3
67	XGB, RFE	{'colsample_bytree': 0.6, 'gamma': 0.5, 'max_depth': 2, 'min_child_weight': 5, 'random_state': 67, 'subsample': 0.6}	1	17	15	7	9	3	13	11	10	14	8	5	6	12	16	4
68	XGB, RFE	{'colsample_bytree': 0.6, 'gamma': 0.5, 'max_depth': 2, 'min_child_weight': 5, 'random_state': 68, 'subsample': 0.6}	10	8	11	4	3	14	7	16	17	9	6	5	13	15	2	1
69	XGB, RFE	{'colsample_bytree': 0.6, 'gamma': 0.5, 'max_depth': 2, 'min_child_weight': 5, 'random_state': 69, 'subsample': 0.6}	11	16	17	7	5	8	13	9	6	10	4	12	1	14	2	3
70	XGB, RFE	{'colsample_bytree': 0.6, 'gamma': 0.5, 'max_depth': 2, 'min_child_weight': 5, 'random_state': 70, 'subsample': 0.6}	6	17	7	4	5	11	9	12	15	3	8	10	13	16	2	1
71	XGB, RFE	{'colsample_bytree': 0.6, 'gamma': 0.5, 'max_depth': 2, 'min_child_weight': 5, 'random_state': 71, 'subsample': 0.6}	8	12	9	6	3	15	4	13	14	11	2	7	10	17	5	1
72	XGB, RFE	{'colsample_bytree': 0.6, 'gamma': 0.5, 'max_depth': 2, 'min_child_weight': 5, 'random_state': 72, 'subsample': 0.6}	15	11	12	3	7	5	10	4	17	6	2	13	1	14	8	16
73	XGB, RFE	{'colsample_bytree': 0.6, 'gamma': 0.5, 'max_depth': 2, 'min_child_weight': 5, 'random_state': 73, 'subsample': 0.6}	12	16	11	2	8	3	14	9	7	5	4	6	15	17	1	13

74	XGB, RFE	{'colsample_bytree': 0.6, 'gamma': 0.5, 'max_depth': 2, 'min_child_weight': 5, 'random_state': 74, 'subsample': 0.6}	5	13	7	3	10	11	2	12	6	9	1	14	4	16	15	17
75	XGB, RFE	{'colsample_bytree': 0.6, 'gamma': 0.5, 'max_depth': 2, 'min_child_weight': 5, 'random_state': 75, 'subsample': 0.6}	8	15	13	4	3	9	1	7	17	6	5	16	11	14	10	2
76	XGB, RFE	{'colsample_bytree': 0.6, 'gamma': 0.5, 'max_depth': 2, 'min_child_weight': 5, 'random_state': 76, 'subsample': 0.6}	9	14	15	2	12	5	1	11	16	10	3	6	4	17	13	7
77	XGB, RFE	{'colsample_bytree': 0.6, 'gamma': 0.5, 'max_depth': 2, 'min_child_weight': 5, 'random_state': 77, 'subsample': 0.6}	4	15	7	3	8	6	2	14	13	10	1	9	11	17	12	16
78	XGB, RFE	{'colsample_bytree': 0.6, 'gamma': 0.5, 'max_depth': 2, 'min_child_weight': 5, 'random_state': 78, 'subsample': 0.6}	5	17	12	4	11	13	1	8	16	7	3	10	14	15	6	2
79	XGB, RFE	{'colsample_bytree': 0.6, 'gamma': 0.5, 'max_depth': 2, 'min_child_weight': 5, 'random_state': 79, 'subsample': 0.6}	6	17	15	7	9	11	2	10	16	5	1	3	8	14	13	4
80	XGB, RFE	{'colsample_bytree': 0.6, 'gamma': 0.5, 'max_depth': 2, 'min_child_weight': 5, 'random_state': 80, 'subsample': 0.6}	7	14	11	5	6	8	9	10	17	13	2	12	4	16	1	3
81	XGB, RFE	{'colsample_bytree': 0.6, 'gamma': 0.5, 'max_depth': 2, 'min_child_weight': 5, 'random_state': 81, 'subsample': 0.6}	11	16	7	10	6	4	5	3	15	8	2	13	9	14	17	1
82	XGB, RFE	{'colsample_bytree': 0.6, 'gamma': 0.5, 'max_depth': 2, 'min_child_weight': 5, 'random_state': 82, 'subsample': 0.6}	6	16	13	2	10	3	11	5	14	12	8	9	4	17	7	1
83	XGB, RFE	{'colsample_bytree': 0.6, 'gamma': 0.5, 'max_depth': 2, 'min_child_weight': 5, 'random_state': 83, 'subsample': 0.6}	11	17	7	6	8	9	10	13	16	2	5	1	12	14	3	15
84	XGB, RFE	{'colsample_bytree': 0.6, 'gamma': 0.5, 'max_depth': 2, 'min_child_weight': 5, 'random_state': 84, 'subsample': 0.6}	11	14	8	7	4	2	5	13	16	6	3	9	12	17	10	1
85	XGB, RFE	{'colsample_bytree': 0.6, 'gamma': 0.5, 'max_depth': 2, 'min_child_weight': 5, 'random_state': 85, 'subsample': 0.6}	8	12	6	4	10	9	5	13	16	11	2	14	7	15	1	17
86	XGB, RFE	{'colsample_bytree': 0.6, 'gamma': 0.5, 'max_depth': 2, 'min_child_weight': 5, 'random_state': 86, 'subsample': 0.6}	4	13	11	8	10	6	17	5	12	16	2	7	9	15	14	3
87	XGB, RFE	{'colsample_bytree': 0.6, 'gamma': 0.5, 'max_depth': 2, 'min_child_weight': 5, 'random_state': 87, 'subsample': 0.6}	11	14	12	7	2	3	4	6	17	10	5	15	9	16	8	1
88	XGB, RFE	{'colsample_bytree': 0.6, 'gamma': 0.5, 'max_depth': 2, 'min_child_weight': 5, 'random_state': 88, 'subsample': 0.6}	3	12	17	5	7	9	10	15	14	8	2	11	1	13	6	4

89	XGB, RFE	{'colsample_bytree': 0.6, 'gamma': 0.5, 'max_depth': 2, 'min_child_weight': 5, 'random_state': 89, 'subsample': 0.6}	9	17	16	11	1	3	5	15	10	8	4	7	2	14	12	13
90	XGB, RFE	{'colsample_bytree': 0.6, 'gamma': 0.5, 'max_depth': 2, 'min_child_weight': 5, 'random_state': 90, 'subsample': 0.6}	11	12	16	7	6	14	2	13	17	3	1	10	9	15	5	8
91	XGB, RFE	{'colsample_bytree': 0.6, 'gamma': 0.5, 'max_depth': 2, 'min_child_weight': 5, 'random_state': 91, 'subsample': 0.6}	7	16	9	4	11	10	3	14	13	8	1	15	6	17	5	2
92	XGB, RFE	{'colsample_bytree': 0.6, 'gamma': 0.5, 'max_depth': 2, 'min_child_weight': 5, 'random_state': 92, 'subsample': 0.6}	9	10	11	4	12	2	7	5	17	15	1	13	6	14	8	3
93	XGB, RFE	{'colsample_bytree': 0.6, 'gamma': 0.5, 'max_depth': 2, 'min_child_weight': 5, 'random_state': 93, 'subsample': 0.6}	13	14	15	5	7	3	10	2	16	9	1	11	4	17	12	6
94	XGB, RFE	{'colsample_bytree': 0.6, 'gamma': 0.5, 'max_depth': 2, 'min_child_weight': 5, 'random_state': 94, 'subsample': 0.6}	1	17	4	3	9	8	6	7	16	14	5	13	10	11	12	2
95	XGB, RFE	{'colsample_bytree': 0.6, 'gamma': 0.5, 'max_depth': 2, 'min_child_weight': 5, 'random_state': 95, 'subsample': 0.6}	5	14	6	1	4	13	12	3	17	11	9	7	15	16	8	2
96	XGB, RFE	{'colsample_bytree': 0.6, 'gamma': 0.5, 'max_depth': 2, 'min_child_weight': 5, 'random_state': 96, 'subsample': 0.6}	4	17	10	2	7	6	11	5	16	3	1	15	9	13	8	12
97	XGB, RFE	{'colsample_bytree': 0.6, 'gamma': 0.5, 'max_depth': 2, 'min_child_weight': 5, 'random_state': 97, 'subsample': 0.6}	5	13	6	3	4	12	2	14	15	10	1	16	9	8	7	17
98	XGB, RFE	{'colsample_bytree': 0.6, 'gamma': 0.5, 'max_depth': 2, 'min_child_weight': 5, 'random_state': 98, 'subsample': 0.6}	4	10	15	3	6	9	8	11	14	12	16	5	17	13	2	1
99	XGB, RFE	{'colsample_bytree': 0.6, 'gamma': 0.5, 'max_depth': 2, 'min_child_weight': 5, 'random_state': 99, 'subsample': 0.6}	2	16	5	3	4	10	7	9	17	6	1	12	8	11	14	15
0	AVG		5	15	14	2	6	1	4	8	16	7	0	10	11	12	3	9
1	AVG		2	15	14	1	4	5	8	10	16	0	3	11	9	12	6	7
2	AVG		9	12	11	2	5	0	6	10	16	7	1	14	8	13	4	3
3	AVG		5	16	11	0	7	1	10	3	13	9	2	8	12	14	6	4
4	AVG		2	15	12	1	5	0	11	9	16	13	3	8	6	14	7	4
5	AVG		4	13	15	2	6	0	8	9	16	5	1	14	3	11	7	10
6	AVG		5	15	14	2	4	1	11	9	13	3	0	10	8	12	7	6
7	AVG		3	10	12	0	7	1	13	6	16	8	4	15	5	11	9	2
8	AVG		4	16	14	1	2	3	9	12	13	8	0	5	6	11	10	7

9	AVG		3	15	12	1	5	4	9	7	14	11	0	8	16	13	2	6
10	AVG		5	16	13	1	6	2	8	7	15	9	0	14	10	11	3	4
11	AVG		4	13	14	1	6	2	9	5	15	10	0	11	7	12	8	3
12	AVG		2	14	12	0	4	1	13	9	16	10	7	3	8	11	5	6
13	AVG		5	13	14	1	2	3	4	9	16	11	0	7	6	12	8	10
14	AVG		6	16	13	0	5	2	11	9	15	8	1	14	3	10	7	4
15	AVG		6	14	13	5	3	1	9	7	16	8	0	15	2	11	4	10
16	AVG		3	15	10	0	7	4	9	6	16	12	1	5	8	13	11	2
17	AVG		4	16	12	3	2	0	9	6	15	8	1	11	14	10	5	7
18	AVG		3	15	10	1	4	0	5	9	16	6	2	11	13	14	8	7
19	AVG		8	15	13	2	4	1	6	11	14	7	0	10	9	12	5	3
20	AVG		4	11	8	1	2	3	13	10	16	7	0	14	9	15	6	5
21	AVG		5	14	13	2	3	1	9	8	16	6	0	11	7	12	10	4
22	AVG		3	15	14	1	5	2	7	10	16	11	0	9	6	13	8	4
23	AVG		3	16	11	4	1	2	10	6	15	7	0	12	8	14	9	5
24	AVG		5	14	13	2	4	0	9	8	16	3	1	15	6	10	7	11
25	AVG		4	15	14	2	0	1	10	9	13	7	3	11	5	12	8	6
26	AVG		5	14	12	0	1	3	6	13	15	9	2	10	7	11	8	4
27	AVG		4	11	14	0	7	1	9	8	16	5	3	12	10	13	6	2
28	AVG		3	15	16	0	4	8	10	9	14	2	1	11	6	12	7	5
29	AVG		1	14	11	0	3	5	6	9	16	7	2	15	8	12	4	10
30	AVG		3	12	14	1	4	2	6	8	15	5	0	10	13	11	7	9
31	AVG		8	15	9	5	7	1	2	6	16	10	0	14	11	12	4	3
32	AVG		4	15	13	1	2	3	7	12	14	6	0	8	10	11	9	5
33	AVG		8	14	12	1	5	3	4	9	15	6	0	11	7	13	10	2
34	AVG		8	15	10	1	2	3	4	7	16	11	0	14	9	12	5	6
35	AVG		5	16	14	1	2	3	8	9	15	6	0	11	7	12	10	4
36	AVG		5	15	14	1	3	2	4	8	16	6	0	11	10	12	7	9
37	AVG		7	14	13	1	4	2	10	5	16	8	0	15	6	11	9	3
38	AVG		2	14	12	1	0	7	4	13	16	9	3	10	5	15	8	6
39	AVG		4	16	11	0	8	6	9	10	15	7	1	14	3	12	2	5

40	AVG		2	16	11	4	1	3	5	13	15	7	0	6	8	14	9	12
41	AVG		5	14	9	1	7	2	3	10	16	4	0	8	15	12	6	11
42	AVG		6	15	12	0	8	1	5	9	16	10	2	11	3	13	4	7
43	AVG		1	16	14	4	2	3	11	8	15	9	0	12	7	13	6	5
44	AVG		5	16	13	1	3	6	7	4	15	2	0	10	14	8	9	11
45	AVG		4	9	14	1	3	2	10	12	15	7	0	16	5	11	6	8
46	AVG		3	13	14	2	6	1	9	15	16	7	0	8	11	10	5	4
47	AVG		5	15	12	0	2	3	6	11	16	9	1	10	7	14	8	4
48	AVG		2	16	13	0	8	3	10	6	15	5	1	14	11	12	7	4
49	AVG		4	12	14	0	6	1	9	7	15	3	2	13	5	11	10	8
50	AVG		4	14	12	0	8	2	3	6	16	5	1	15	11	13	10	7
51	AVG		3	16	14	0	7	1	10	5	12	8	6	11	9	15	4	2
52	AVG		8	12	13	1	3	2	5	6	16	10	0	15	7	11	9	4
53	AVG		2	15	13	1	3	4	8	9	16	7	0	11	10	12	6	5
54	AVG		5	15	14	1	3	0	10	8	16	7	2	13	6	11	4	9
55	AVG		4	15	12	2	3	1	7	9	16	5	0	10	8	14	6	11
56	AVG		10	15	11	1	4	2	9	7	16	8	0	14	6	12	3	5
57	AVG		9	15	12	3	2	1	5	10	16	6	4	11	0	14	7	8
58	AVG		3	15	13	1	8	0	4	7	16	12	2	14	9	11	6	5
59	AVG		3	14	11	5	2	0	4	6	15	10	1	12	9	13	8	7
60	AVG		4	13	11	3	5	2	8	9	16	0	1	12	10	15	7	6
61	AVG		9	15	13	0	6	3	7	10	12	5	1	16	8	11	2	4
62	AVG		1	16	13	4	2	3	5	12	15	8	0	11	9	10	7	6
63	AVG		9	14	12	0	7	4	11	3	16	8	1	10	2	13	6	5
64	AVG		3	15	12	2	6	1	7	8	14	5	0	11	10	13	9	4
65	AVG		2	13	12	0	3	7	5	9	14	4	1	10	6	15	11	8
66	AVG		0	15	14	2	1	4	5	6	16	7	3	13	11	12	8	9
67	AVG		3	16	14	2	5	0	12	9	15	6	4	10	1	13	11	8
68	AVG		8	10	14	0	2	3	9	11	16	6	1	7	13	12	4	5
69	AVG		4	15	14	0	3	1	9	6	12	10	2	13	8	11	5	7
70	AVG		5	16	11	0	7	2	9	10	14	6	1	8	13	12	3	4

71	AVG		5	13	12	4	1	6	2	10	14	11	0	8	9	15	7	3
72	AVG		7	14	12	2	3	0	8	5	15	6	1	16	4	11	9	10
73	AVG		6	15	13	1	5	2	10	7	11	3	0	8	9	14	4	16
74	AVG		6	15	10	1	2	4	5	8	9	7	0	11	3	14	12	16
75	AVG		7	14	10	1	2	3	6	9	16	4	0	15	12	11	8	5
76	AVG		4	16	15	0	3	2	7	8	14	6	1	10	5	13	9	12
77	AVG		4	16	10	0	5	1	3	12	14	7	2	11	13	15	6	8
78	AVG		6	15	11	1	8	2	7	9	16	3	0	12	14	13	4	5
79	AVG		3	16	13	2	5	1	8	10	15	4	0	11	7	12	9	6
80	AVG		4	12	11	1	6	2	9	7	16	8	0	14	10	13	5	3
81	AVG		7	14	10	1	6	0	8	5	16	3	4	11	9	12	13	2
82	AVG		5	11	13	1	7	0	12	8	15	10	2	9	3	14	4	6
83	AVG		6	16	9	0	4	3	10	11	15	1	2	8	7	14	5	13
84	AVG		8	15	11	2	3	0	5	10	16	4	1	9	12	13	6	7
85	AVG		5	12	9	1	4	2	7	8	16	6	0	15	10	13	3	11
86	AVG		4	15	14	1	6	2	13	5	16	7	0	9	11	10	8	3
87	AVG		7	15	12	4	3	0	6	9	16	5	1	13	10	11	8	2
88	AVG		5	14	16	1	7	2	9	10	15	6	0	11	4	12	3	8
89	AVG		6	16	15	3	4	0	5	10	14	7	2	13	1	12	8	9
90	AVG		8	14	13	1	2	5	3	10	16	6	0	7	11	15	4	9
91	AVG		4	14	12	1	7	2	3	13	16	8	0	9	10	15	6	5
92	AVG		3	14	13	2	6	1	4	8	16	9	0	10	11	12	5	7
93	AVG		8	14	13	1	3	2	7	4	16	6	0	11	10	12	9	5
94	AVG		1	16	6	0	5	3	7	11	15	12	2	13	10	8	9	4
95	AVG		4	12	13	0	5	3	8	2	16	9	1	10	11	14	7	6
96	AVG		2	15	11	5	1	3	7	8	16	4	0	10	13	12	6	9
97	AVG		4	15	9	2	5	3	1	11	16	10	0	14	7	8	6	13
98	AVG		4	12	15	0	5	7	6	8	16	9	3	10	13	14	1	2
99	AVG		2	15	13	1	4	3	9	10	16	6	0	7	5	12	8	11

ii. 1-year VT recurrence

Iteration number (random_state)	Model	Parameters	Age	Atrial fibrillation	NYHA	EF	LVESD	TAPSE	E wave deceleration time (DT)	MR	TR III-IV	ICD shock	HD instability	Incessant_VT	ES or multiple ICD therapies	Inducible VT morphologies	Clinical VT cycle length	Clinical VT eliminated
0	MLP, PI	{'NN__activation': 'identity', 'NN__alpha': 1, 'NN__hidden_layer_sizes': (5,), 'NN__max_iter': 200, 'NN__random_state': 0, 'NN__solver': 'adam'}	15	7	6	11	4	5	10	2	13	3	9	1	12	0	16	14
1	MLP, PI	{'NN__activation': 'identity', 'NN__alpha': 1, 'NN__hidden_layer_sizes': (5,), 'NN__max_iter': 200, 'NN__random_state': 1, 'NN__solver': 'adam'}	14	10	8	12	3	7	6	5	9	2	4	1	11	0	16	15
2	MLP, PI	{'NN__activation': 'identity', 'NN__alpha': 1, 'NN__hidden_layer_sizes': (5,), 'NN__max_iter': 200, 'NN__random_state': 2, 'NN__solver': 'adam'}	14	10	8	13	2	5	6	7	11	3	4	1	9	0	15	16
3	MLP, PI	{'NN__activation': 'identity', 'NN__alpha': 1, 'NN__hidden_layer_sizes': (5,), 'NN__max_iter': 200, 'NN__random_state': 3, 'NN__solver': 'adam'}	10	9	8	12	6	5	16	2	11	3	4	1	7	0	13	15
4	MLP, PI	{'NN__activation': 'identity', 'NN__alpha': 1, 'NN__hidden_layer_sizes': (5,), 'NN__max_iter': 200, 'NN__random_state': 4, 'NN__solver': 'adam'}	12	10	7	15	8	5	1	3	16	4	2	6	14	0	13	11

5	MLP, PI	{'NN__activation': 'identity', 'NN__alpha': 1, 'NN__hidden_layer_sizes': (5,), 'NN__max_iter': 200, 'NN__random_state': 5, 'NN__solver': 'adam'}	11	14	16	9	4	5	6	2	15	3	1	10	7	0	12	13
6	MLP, PI	{'NN__activation': 'identity', 'NN__alpha': 1, 'NN__hidden_layer_sizes': (5,), 'NN__max_iter': 200, 'NN__random_state': 6, 'NN__solver': 'adam'}	14	12	8	13	6	7	4	2	9	1	3	5	16	0	15	11
7	MLP, PI	{'NN__activation': 'identity', 'NN__alpha': 1, 'NN__hidden_layer_sizes': (5,), 'NN__max_iter': 200, 'NN__random_state': 7, 'NN__solver': 'adam'}	12	13	9	15	4	5	7	3	8	10	2	11	1	0	14	16
8	MLP, PI	{'NN__activation': 'identity', 'NN__alpha': 1, 'NN__hidden_layer_sizes': (5,), 'NN__max_iter': 200, 'NN__random_state': 8, 'NN__solver': 'adam'}	13	8	6	16	3	9	10	7	14	2	4	0	5	1	15	11
9	MLP, PI	{'NN__activation': 'identity', 'NN__alpha': 1, 'NN__hidden_layer_sizes': (5,), 'NN__max_iter': 200, 'NN__random_state': 9, 'NN__solver': 'adam'}	14	9	11	8	7	6	3	5	15	16	2	1	4	0	13	12
10	MLP, PI	{'NN__activation': 'identity', 'NN__alpha': 1, 'NN__hidden_layer_sizes': (5,), 'NN__max_iter': 200, 'NN__random_state': 10, 'NN__solver': 'adam'}	16	10	7	13	2	5	6	8	12	4	3	1	9	0	14	15
11	MLP, PI	{'NN__activation': 'identity', 'NN__alpha': 1, 'NN__hidden_layer_sizes': (5,), 'NN__max_iter': 200, 'NN__random_state': 11, 'NN__solver': 'adam'}	15	13	8	11	4	5	7	2	12	6	3	10	1	0	14	16
12	MLP, PI	{'NN__activation': 'identity', 'NN__alpha': 1, 'NN__hidden_layer_sizes': (5,), 'NN__max_iter': 200, 'NN__random_state': 12, 'NN__solver': 'adam'}	5	7	4	16	12	6	15	1	9	2	11	3	8	0	13	14
13	MLP, PI	{'NN__activation': 'identity', 'NN__alpha': 1, 'NN__hidden_layer_sizes': (5,), 'NN__max_iter': 200, 'NN__random_state': 13, 'NN__solver': 'adam'}	10	11	9	13	4	6	5	2	14	12	3	0	7	1	16	15
14	MLP, PI	{'NN__activation': 'identity', 'NN__alpha': 1, 'NN__hidden_layer_sizes': (5,), 'NN__max_iter': 200, 'NN__random_state': 14, 'NN__solver': 'adam'}	16	14	7	8	6	4	5	3	11	2	9	13	1	0	12	10

15	MLP, PI	{'NN__activation': 'identity', 'NN__alpha': 1, 'NN__hidden_layer_sizes': (5,), 'NN__max_iter': 200, 'NN__random_state': 15, 'NN__solver': 'adam'}	10	13	7	12	5	6	8	3	11	2	4	9	1	0	16	14
16	MLP, PI	{'NN__activation': 'identity', 'NN__alpha': 1, 'NN__hidden_layer_sizes': (5,), 'NN__max_iter': 200, 'NN__random_state': 16, 'NN__solver': 'adam'}	13	8	7	9	11	10	3	4	15	6	5	0	2	1	16	14
17	MLP, PI	{'NN__activation': 'identity', 'NN__alpha': 1, 'NN__hidden_layer_sizes': (5,), 'NN__max_iter': 200, 'NN__random_state': 17, 'NN__solver': 'adam'}	12	10	5	11	3	7	8	4	14	2	6	1	9	0	15	13
18	MLP, PI	{'NN__activation': 'identity', 'NN__alpha': 1, 'NN__hidden_layer_sizes': (5,), 'NN__max_iter': 200, 'NN__random_state': 18, 'NN__solver': 'adam'}	13	12	7	9	5	10	8	3	15	2	4	1	6	0	16	11
19	MLP, PI	{'NN__activation': 'identity', 'NN__alpha': 1, 'NN__hidden_layer_sizes': (5,), 'NN__max_iter': 200, 'NN__random_state': 19, 'NN__solver': 'adam'}	16	12	5	14	3	7	11	4	9	6	8	0	2	1	15	13
20	MLP, PI	{'NN__activation': 'identity', 'NN__alpha': 1, 'NN__hidden_layer_sizes': (5,), 'NN__max_iter': 200, 'NN__random_state': 20, 'NN__solver': 'adam'}	10	7	9	16	5	4	13	2	11	8	3	1	6	0	14	15
21	MLP, PI	{'NN__activation': 'identity', 'NN__alpha': 1, 'NN__hidden_layer_sizes': (5,), 'NN__max_iter': 200, 'NN__random_state': 21, 'NN__solver': 'adam'}	14	9	8	11	1	5	6	7	12	2	3	4	15	0	16	13
22	MLP, PI	{'NN__activation': 'identity', 'NN__alpha': 1, 'NN__hidden_layer_sizes': (5,), 'NN__max_iter': 200, 'NN__random_state': 22, 'NN__solver': 'adam'}	13	15	7	12	2	8	3	5	16	4	6	1	9	0	14	11
23	MLP, PI	{'NN__activation': 'identity', 'NN__alpha': 1, 'NN__hidden_layer_sizes': (5,), 'NN__max_iter': 200, 'NN__random_state': 23, 'NN__solver': 'adam'}	9	12	16	10	0	4	11	5	14	2	3	8	6	1	15	13
24	MLP, PI	{'NN__activation': 'identity', 'NN__alpha': 1, 'NN__hidden_layer_sizes': (5,), 'NN__max_iter': 200, 'NN__random_state': 24, 'NN__solver': 'adam'}	14	11	8	15	1	7	6	5	9	2	3	4	16	0	13	12

25	MLP, PI	{'NN__activation': 'identity', 'NN__alpha': 1, 'NN__hidden_layer_sizes': (5,), 'NN__max_iter': 200, 'NN__random_state': 25, 'NN__solver': 'adam'}	16	12	9	13	2	8	7	4	15	10	3	0	6	1	14	11
26	MLP, PI	{'NN__activation': 'identity', 'NN__alpha': 1, 'NN__hidden_layer_sizes': (5,), 'NN__max_iter': 200, 'NN__random_state': 26, 'NN__solver': 'adam'}	11	8	9	10	1	7	5	4	14	16	0	3	15	2	13	12
27	MLP, PI	{'NN__activation': 'identity', 'NN__alpha': 1, 'NN__hidden_layer_sizes': (5,), 'NN__max_iter': 200, 'NN__random_state': 27, 'NN__solver': 'adam'}	12	9	8	2	16	11	4	5	15	7	6	0	3	1	14	13
28	MLP, PI	{'NN__activation': 'identity', 'NN__alpha': 1, 'NN__hidden_layer_sizes': (5,), 'NN__max_iter': 200, 'NN__random_state': 28, 'NN__solver': 'adam'}	10	6	4	7	5	9	15	1	16	2	8	3	13	0	12	14
29	MLP, PI	{'NN__activation': 'identity', 'NN__alpha': 1, 'NN__hidden_layer_sizes': (5,), 'NN__max_iter': 200, 'NN__random_state': 29, 'NN__solver': 'adam'}	13	9	8	7	10	3	6	1	12	2	5	16	4	0	14	15
30	MLP, PI	{'NN__activation': 'identity', 'NN__alpha': 1, 'NN__hidden_layer_sizes': (5,), 'NN__max_iter': 200, 'NN__random_state': 30, 'NN__solver': 'adam'}	10	6	9	2	16	8	11	1	12	4	5	3	7	0	15	14
31	MLP, PI	{'NN__activation': 'identity', 'NN__alpha': 1, 'NN__hidden_layer_sizes': (5,), 'NN__max_iter': 200, 'NN__random_state': 31, 'NN__solver': 'adam'}	13	10	7	14	2	4	8	6	11	3	5	0	9	1	15	16
32	MLP, PI	{'NN__activation': 'identity', 'NN__alpha': 1, 'NN__hidden_layer_sizes': (5,), 'NN__max_iter': 200, 'NN__random_state': 32, 'NN__solver': 'adam'}	13	12	8	15	7	9	5	3	14	6	4	0	2	1	11	16
33	MLP, PI	{'NN__activation': 'identity', 'NN__alpha': 1, 'NN__hidden_layer_sizes': (5,), 'NN__max_iter': 200, 'NN__random_state': 33, 'NN__solver': 'adam'}	11	12	7	16	1	8	6	3	10	2	5	4	13	0	14	15
34	MLP, PI	{'NN__activation': 'identity', 'NN__alpha': 1, 'NN__hidden_layer_sizes': (5,), 'NN__max_iter': 200, 'NN__random_state': 34, 'NN__solver': 'adam'}	10	15	9	13	6	7	5	1	8	2	3	16	4	0	11	14

35	MLP, PI	{'NN__activation': 'identity', 'NN__alpha': 1, 'NN__hidden_layer_sizes': (5,), 'NN__max_iter': 200, 'NN__random_state': 35, 'NN__solver': 'adam'}	11	8	4	16	2	6	5	1	13	3	7	12	10	0	15	14
36	MLP, PI	{'NN__activation': 'identity', 'NN__alpha': 1, 'NN__hidden_layer_sizes': (5,), 'NN__max_iter': 200, 'NN__random_state': 36, 'NN__solver': 'adam'}	11	14	9	13	2	8	7	6	15	3	5	0	4	1	16	12
37	MLP, PI	{'NN__activation': 'identity', 'NN__alpha': 1, 'NN__hidden_layer_sizes': (5,), 'NN__max_iter': 200, 'NN__random_state': 37, 'NN__solver': 'adam'}	15	8	7	10	2	5	13	1	9	3	4	12	6	0	11	14
38	MLP, PI	{'NN__activation': 'identity', 'NN__alpha': 1, 'NN__hidden_layer_sizes': (5,), 'NN__max_iter': 200, 'NN__random_state': 38, 'NN__solver': 'adam'}	10	7	4	9	1	16	2	14	13	15	3	5	8	0	12	11
39	MLP, PI	{'NN__activation': 'identity', 'NN__alpha': 1, 'NN__hidden_layer_sizes': (5,), 'NN__max_iter': 200, 'NN__random_state': 39, 'NN__solver': 'adam'}	14	9	5	2	12	10	3	4	11	1	7	16	6	0	15	13
40	MLP, PI	{'NN__activation': 'identity', 'NN__alpha': 1, 'NN__hidden_layer_sizes': (5,), 'NN__max_iter': 200, 'NN__random_state': 40, 'NN__solver': 'adam'}	16	10	12	15	3	14	4	6	11	0	2	5	13	1	8	7
41	MLP, PI	{'NN__activation': 'identity', 'NN__alpha': 1, 'NN__hidden_layer_sizes': (5,), 'NN__max_iter': 200, 'NN__random_state': 41, 'NN__solver': 'adam'}	15	10	6	16	5	11	7	4	13	3	2	1	12	0	9	8
42	MLP, PI	{'NN__activation': 'identity', 'NN__alpha': 1, 'NN__hidden_layer_sizes': (5,), 'NN__max_iter': 200, 'NN__random_state': 42, 'NN__solver': 'adam'}	8	16	5	6	11	2	3	12	15	9	4	7	1	0	13	14
43	MLP, PI	{'NN__activation': 'identity', 'NN__alpha': 1, 'NN__hidden_layer_sizes': (5,), 'NN__max_iter': 200, 'NN__random_state': 43, 'NN__solver': 'adam'}	13	11	10	12	5	9	7	3	15	6	2	0	4	1	14	16
44	MLP, PI	{'NN__activation': 'identity', 'NN__alpha': 1, 'NN__hidden_layer_sizes': (5,), 'NN__max_iter': 200, 'NN__random_state': 44, 'NN__solver': 'adam'}	10	8	7	15	2	12	6	3	14	1	4	5	16	0	13	11

45	MLP, PI	{'NN__activation': 'identity', 'NN__alpha': 1, 'NN__hidden_layer_sizes': (5,), 'NN__max_iter': 200, 'NN__random_state': 45, 'NN__solver': 'adam'}	13	11	8	14	2	7	4	1	10	6	3	5	16	0	15	12
46	MLP, PI	{'NN__activation': 'identity', 'NN__alpha': 1, 'NN__hidden_layer_sizes': (5,), 'NN__max_iter': 200, 'NN__random_state': 46, 'NN__solver': 'adam'}	13	16	8	14	0	6	5	7	10	4	3	2	12	1	15	11
47	MLP, PI	{'NN__activation': 'identity', 'NN__alpha': 1, 'NN__hidden_layer_sizes': (5,), 'NN__max_iter': 200, 'NN__random_state': 47, 'NN__solver': 'adam'}	14	9	6	15	1	8	4	5	16	2	3	7	12	0	13	10
48	MLP, PI	{'NN__activation': 'identity', 'NN__alpha': 1, 'NN__hidden_layer_sizes': (5,), 'NN__max_iter': 200, 'NN__random_state': 48, 'NN__solver': 'adam'}	9	16	3	13	4	8	7	6	12	1	5	2	15	0	14	11
49	MLP, PI	{'NN__activation': 'identity', 'NN__alpha': 1, 'NN__hidden_layer_sizes': (5,), 'NN__max_iter': 200, 'NN__random_state': 49, 'NN__solver': 'adam'}	11	8	7	14	2	5	10	1	9	4	6	16	3	0	13	15
50	MLP, PI	{'NN__activation': 'identity', 'NN__alpha': 1, 'NN__hidden_layer_sizes': (5,), 'NN__max_iter': 200, 'NN__random_state': 50, 'NN__solver': 'adam'}	12	10	8	9	7	5	6	2	15	3	4	1	11	0	14	16
51	MLP, PI	{'NN__activation': 'identity', 'NN__alpha': 1, 'NN__hidden_layer_sizes': (5,), 'NN__max_iter': 200, 'NN__random_state': 51, 'NN__solver': 'adam'}	13	10	7	9	4	5	12	3	8	1	16	2	15	0	6	14
52	MLP, PI	{'NN__activation': 'identity', 'NN__alpha': 1, 'NN__hidden_layer_sizes': (5,), 'NN__max_iter': 200, 'NN__random_state': 52, 'NN__solver': 'adam'}	16	9	5	12	1	6	7	2	14	4	3	10	8	0	13	15
53	MLP, PI	{'NN__activation': 'identity', 'NN__alpha': 1, 'NN__hidden_layer_sizes': (5,), 'NN__max_iter': 200, 'NN__random_state': 53, 'NN__solver': 'adam'}	9	7	6	16	11	2	14	1	12	5	4	3	10	0	15	13
54	MLP, PI	{'NN__activation': 'identity', 'NN__alpha': 1, 'NN__hidden_layer_sizes': (5,), 'NN__max_iter': 200, 'NN__random_state': 54, 'NN__solver': 'adam'}	10	9	7	12	6	3	15	2	8	4	5	16	1	0	14	13

55	MLP, PI	{'NN__activation': 'identity', 'NN__alpha': 1, 'NN__hidden_layer_sizes': (5,), 'NN__max_iter': 200, 'NN__random_state': 55, 'NN__solver': 'adam'}	12	13	6	14	1	7	4	5	8	3	2	11	9	0	15	10
56	MLP, PI	{'NN__activation': 'identity', 'NN__alpha': 1, 'NN__hidden_layer_sizes': (5,), 'NN__max_iter': 200, 'NN__random_state': 56, 'NN__solver': 'adam'}	10	9	5	12	3	6	1	4	13	11	2	14	8	0	16	15
57	MLP, PI	{'NN__activation': 'identity', 'NN__alpha': 1, 'NN__hidden_layer_sizes': (5,), 'NN__max_iter': 200, 'NN__random_state': 57, 'NN__solver': 'adam'}	9	15	11	12	1	6	7	5	10	2	3	14	4	0	13	16
58	MLP, PI	{'NN__activation': 'identity', 'NN__alpha': 1, 'NN__hidden_layer_sizes': (5,), 'NN__max_iter': 200, 'NN__random_state': 58, 'NN__solver': 'adam'}	12	13	8	11	7	5	4	1	9	3	2	16	6	0	14	15
59	MLP, PI	{'NN__activation': 'identity', 'NN__alpha': 1, 'NN__hidden_layer_sizes': (5,), 'NN__max_iter': 200, 'NN__random_state': 59, 'NN__solver': 'adam'}	11	10	8	16	2	6	3	4	13	5	1	12	9	0	15	14
60	MLP, PI	{'NN__activation': 'identity', 'NN__alpha': 1, 'NN__hidden_layer_sizes': (5,), 'NN__max_iter': 200, 'NN__random_state': 60, 'NN__solver': 'adam'}	14	15	7	16	3	8	9	4	10	5	2	1	6	0	11	12
61	MLP, PI	{'NN__activation': 'identity', 'NN__alpha': 1, 'NN__hidden_layer_sizes': (5,), 'NN__max_iter': 200, 'NN__random_state': 61, 'NN__solver': 'adam'}	9	16	5	15	7	8	4	3	10	2	6	14	1	0	12	13
62	MLP, PI	{'NN__activation': 'identity', 'NN__alpha': 1, 'NN__hidden_layer_sizes': (5,), 'NN__max_iter': 200, 'NN__random_state': 62, 'NN__solver': 'adam'}	13	12	6	8	0	9	7	5	10	3	4	2	15	1	16	11
63	MLP, PI	{'NN__activation': 'identity', 'NN__alpha': 1, 'NN__hidden_layer_sizes': (5,), 'NN__max_iter': 200, 'NN__random_state': 63, 'NN__solver': 'adam'}	10	7	11	5	3	15	14	2	12	6	4	8	0	1	13	16
64	MLP, PI	{'NN__activation': 'identity', 'NN__alpha': 1, 'NN__hidden_layer_sizes': (5,), 'NN__max_iter': 200, 'NN__random_state': 64, 'NN__solver': 'adam'}	13	10	8	7	12	9	6	2	16	3	4	0	5	1	15	11

65	MLP, PI	{'NN__activation': 'identity', 'NN__alpha': 1, 'NN__hidden_layer_sizes': (5,), 'NN__max_iter': 200, 'NN__random_state': 65, 'NN__solver': 'adam'}	14	12	7	13	5	16	6	4	15	3	2	1	8	0	10	11
66	MLP, PI	{'NN__activation': 'identity', 'NN__alpha': 1, 'NN__hidden_layer_sizes': (5,), 'NN__max_iter': 200, 'NN__random_state': 66, 'NN__solver': 'adam'}	9	12	7	10	2	3	6	5	8	1	16	11	4	0	15	13
67	MLP, PI	{'NN__activation': 'identity', 'NN__alpha': 1, 'NN__hidden_layer_sizes': (5,), 'NN__max_iter': 200, 'NN__random_state': 67, 'NN__solver': 'adam'}	13	10	12	5	6	2	9	4	11	3	7	8	1	0	16	14
68	MLP, PI	{'NN__activation': 'identity', 'NN__alpha': 1, 'NN__hidden_layer_sizes': (5,), 'NN__max_iter': 200, 'NN__random_state': 68, 'NN__solver': 'adam'}	16	13	10	11	4	5	6	2	9	3	1	8	12	0	14	15
69	MLP, PI	{'NN__activation': 'identity', 'NN__alpha': 1, 'NN__hidden_layer_sizes': (5,), 'NN__max_iter': 200, 'NN__random_state': 69, 'NN__solver': 'adam'}	15	12	9	13	4	7	6	3	11	2	5	1	8	0	16	14
70	MLP, PI	{'NN__activation': 'identity', 'NN__alpha': 1, 'NN__hidden_layer_sizes': (5,), 'NN__max_iter': 200, 'NN__random_state': 70, 'NN__solver': 'adam'}	10	8	12	11	14	4	7	2	13	3	6	1	5	0	15	16
71	MLP, PI	{'NN__activation': 'identity', 'NN__alpha': 1, 'NN__hidden_layer_sizes': (5,), 'NN__max_iter': 200, 'NN__random_state': 71, 'NN__solver': 'adam'}	15	11	16	6	1	8	9	7	12	5	3	0	4	2	14	13
72	MLP, PI	{'NN__activation': 'identity', 'NN__alpha': 1, 'NN__hidden_layer_sizes': (5,), 'NN__max_iter': 200, 'NN__random_state': 72, 'NN__solver': 'adam'}	13	10	8	16	2	7	5	4	11	1	3	6	9	0	12	15
73	MLP, PI	{'NN__activation': 'identity', 'NN__alpha': 1, 'NN__hidden_layer_sizes': (5,), 'NN__max_iter': 200, 'NN__random_state': 73, 'NN__solver': 'adam'}	14	10	6	8	9	12	7	4	16	2	5	0	3	1	15	11
74	MLP, PI	{'NN__activation': 'identity', 'NN__alpha': 1, 'NN__hidden_layer_sizes': (5,), 'NN__max_iter': 200, 'NN__random_state': 74, 'NN__solver': 'adam'}	15	11	7	16	3	9	6	8	14	4	5	0	2	1	12	10

75	MLP, PI	{'NN__activation': 'identity', 'NN__alpha': 1, 'NN__hidden_layer_sizes': (5,), 'NN__max_iter': 200, 'NN__random_state': 75, 'NN__solver': 'adam'}	15	10	6	8	1	7	5	16	14	3	4	2	13	0	12	11
76	MLP, PI	{'NN__activation': 'identity', 'NN__alpha': 1, 'NN__hidden_layer_sizes': (5,), 'NN__max_iter': 200, 'NN__random_state': 76, 'NN__solver': 'adam'}	13	15	9	16	2	7	6	5	14	4	1	3	10	0	12	8
77	MLP, PI	{'NN__activation': 'identity', 'NN__alpha': 1, 'NN__hidden_layer_sizes': (5,), 'NN__max_iter': 200, 'NN__random_state': 77, 'NN__solver': 'adam'}	13	11	7	14	6	8	2	3	12	4	16	1	10	0	15	9
78	MLP, PI	{'NN__activation': 'identity', 'NN__alpha': 1, 'NN__hidden_layer_sizes': (5,), 'NN__max_iter': 200, 'NN__random_state': 78, 'NN__solver': 'adam'}	7	11	13	6	9	4	8	3	16	1	5	2	15	0	14	12
79	MLP, PI	{'NN__activation': 'identity', 'NN__alpha': 1, 'NN__hidden_layer_sizes': (5,), 'NN__max_iter': 200, 'NN__random_state': 79, 'NN__solver': 'adam'}	13	15	8	14	0	4	6	7	12	5	3	16	2	1	10	11
80	MLP, PI	{'NN__activation': 'identity', 'NN__alpha': 1, 'NN__hidden_layer_sizes': (5,), 'NN__max_iter': 200, 'NN__random_state': 80, 'NN__solver': 'adam'}	6	14	7	11	8	5	4	1	12	3	2	9	16	0	15	13
81	MLP, PI	{'NN__activation': 'identity', 'NN__alpha': 1, 'NN__hidden_layer_sizes': (5,), 'NN__max_iter': 200, 'NN__random_state': 81, 'NN__solver': 'adam'}	9	6	5	7	11	4	8	2	10	1	12	3	16	0	15	14
82	MLP, PI	{'NN__activation': 'identity', 'NN__alpha': 1, 'NN__hidden_layer_sizes': (5,), 'NN__max_iter': 200, 'NN__random_state': 82, 'NN__solver': 'adam'}	12	9	5	15	3	8	2	4	11	16	1	7	10	0	13	14
83	MLP, PI	{'NN__activation': 'identity', 'NN__alpha': 1, 'NN__hidden_layer_sizes': (5,), 'NN__max_iter': 200, 'NN__random_state': 83, 'NN__solver': 'adam'}	8	9	12	2	14	6	5	0	13	1	4	7	15	3	16	11
84	MLP, PI	{'NN__activation': 'identity', 'NN__alpha': 1, 'NN__hidden_layer_sizes': (5,), 'NN__max_iter': 200, 'NN__random_state': 84, 'NN__solver': 'adam'}	15	11	7	10	9	5	6	4	16	3	2	1	8	0	14	12

85	MLP, PI	{'NN__activation': 'identity', 'NN__alpha': 1, 'NN__hidden_layer_sizes': (5,), 'NN__max_iter': 200, 'NN__random_state': 85, 'NN__solver': 'adam'}	13	11	6	14	2	4	8	1	10	5	3	9	12	0	15	16
86	MLP, PI	{'NN__activation': 'identity', 'NN__alpha': 1, 'NN__hidden_layer_sizes': (5,), 'NN__max_iter': 200, 'NN__random_state': 86, 'NN__solver': 'adam'}	8	10	9	16	4	7	6	2	12	1	3	5	14	0	15	13
87	MLP, PI	{'NN__activation': 'identity', 'NN__alpha': 1, 'NN__hidden_layer_sizes': (5,), 'NN__max_iter': 200, 'NN__random_state': 87, 'NN__solver': 'adam'}	13	12	7	16	2	6	8	5	9	3	4	1	10	0	15	14
88	MLP, PI	{'NN__activation': 'identity', 'NN__alpha': 1, 'NN__hidden_layer_sizes': (5,), 'NN__max_iter': 200, 'NN__random_state': 88, 'NN__solver': 'adam'}	10	14	13	8	7	5	6	1	12	3	2	9	11	0	16	15
89	MLP, PI	{'NN__activation': 'identity', 'NN__alpha': 1, 'NN__hidden_layer_sizes': (5,), 'NN__max_iter': 200, 'NN__random_state': 89, 'NN__solver': 'adam'}	10	9	13	11	3	7	4	5	14	2	6	16	1	0	15	12
90	MLP, PI	{'NN__activation': 'identity', 'NN__alpha': 1, 'NN__hidden_layer_sizes': (5,), 'NN__max_iter': 200, 'NN__random_state': 90, 'NN__solver': 'adam'}	13	14	12	16	3	11	7	4	10	5	1	0	2	6	15	8
91	MLP, PI	{'NN__activation': 'identity', 'NN__alpha': 1, 'NN__hidden_layer_sizes': (5,), 'NN__max_iter': 200, 'NN__random_state': 91, 'NN__solver': 'adam'}	13	12	7	11	3	6	4	16	14	5	2	1	8	0	15	10
92	MLP, PI	{'NN__activation': 'identity', 'NN__alpha': 1, 'NN__hidden_layer_sizes': (5,), 'NN__max_iter': 200, 'NN__random_state': 92, 'NN__solver': 'adam'}	15	10	8	14	4	9	7	6	13	5	2	0	3	1	16	12
93	MLP, PI	{'NN__activation': 'identity', 'NN__alpha': 1, 'NN__hidden_layer_sizes': (5,), 'NN__max_iter': 200, 'NN__random_state': 93, 'NN__solver': 'adam'}	14	9	8	5	13	7	6	3	15	4	2	1	12	0	16	11
94	MLP, PI	{'NN__activation': 'identity', 'NN__alpha': 1, 'NN__hidden_layer_sizes': (5,), 'NN__max_iter': 200, 'NN__random_state': 94, 'NN__solver': 'adam'}	15	7	9	2	4	8	3	10	14	5	6	1	12	0	16	11

95	MLP, PI	{'NN__activation': 'identity', 'NN__alpha': 1, 'NN__hidden_layer_sizes': (5,), 'NN__max_iter': 200, 'NN__random_state': 95, 'NN__solver': 'adam'}	8	11	14	15	7	5	10	1	12	4	9	13	3	0	16	6
96	MLP, PI	{'NN__activation': 'identity', 'NN__alpha': 1, 'NN__hidden_layer_sizes': (5,), 'NN__max_iter': 200, 'NN__random_state': 96, 'NN__solver': 'adam'}	13	11	8	9	0	5	7	6	14	4	3	2	16	1	10	15
97	MLP, PI	{'NN__activation': 'identity', 'NN__alpha': 1, 'NN__hidden_layer_sizes': (5,), 'NN__max_iter': 200, 'NN__random_state': 97, 'NN__solver': 'adam'}	13	9	3	16	4	8	5	1	11	2	6	7	12	0	15	10
98	MLP, PI	{'NN__activation': 'identity', 'NN__alpha': 1, 'NN__hidden_layer_sizes': (5,), 'NN__max_iter': 200, 'NN__random_state': 98, 'NN__solver': 'adam'}	14	7	4	9	6	16	2	1	15	5	3	8	12	0	13	11
99	MLP, PI	{'NN__activation': 'identity', 'NN__alpha': 1, 'NN__hidden_layer_sizes': (5,), 'NN__max_iter': 200, 'NN__random_state': 99, 'NN__solver': 'adam'}	16	10	5	6	9	11	7	8	13	4	2	0	3	1	12	15
0	RF, PI	{'criterion': 'gini', 'max_depth': 2, 'max_features': 2, 'min_samples_leaf': 5, 'n_estimators': 500, 'n_jobs': -1, 'random_state': 0}	8	12	10	4	0	9	5	2	13	6	7	11	16	1	3	15
1	RF, PI	{'criterion': 'gini', 'max_depth': 2, 'max_features': 2, 'min_samples_leaf': 5, 'n_estimators': 500, 'n_jobs': -1, 'random_state': 1}	4	14	9	8	0	10	7	3	12	2	5	11	15	1	6	16
2	RF, PI	{'criterion': 'gini', 'max_depth': 2, 'max_features': 2, 'min_samples_leaf': 5, 'n_estimators': 500, 'n_jobs': -1, 'random_state': 2}	8	14	10	3	0	7	9	6	11	5	2	15	16	1	4	13
3	RF, PI	{'criterion': 'gini', 'max_depth': 2, 'max_features': 2, 'min_samples_leaf': 5, 'n_estimators': 500, 'n_jobs': -1, 'random_state': 3}	5	12	10	8	0	9	6	2	13	4	3	14	16	1	7	15
4	RF, PI	{'criterion': 'gini', 'max_depth': 2, 'max_features': 2, 'min_samples_leaf': 5, 'n_estimators': 500, 'n_jobs': -1, 'random_state': 4}	7	14	11	9	1	8	3	2	13	5	6	12	16	0	4	15

5	RF, PI	{'criterion': 'gini', 'max_depth': 2, 'max_features': 2, 'min_samples_leaf': 5, 'n_estimators': 500, 'n_jobs': -1, 'random_state': 5}	4	15	10	6	1	9	8	5	11	2	3	14	16	0	7	13
6	RF, PI	{'criterion': 'gini', 'max_depth': 2, 'max_features': 2, 'min_samples_leaf': 5, 'n_estimators': 500, 'n_jobs': -1, 'random_state': 6}	6	14	11	8	0	9	5	3	13	2	7	10	16	1	4	15
7	RF, PI	{'criterion': 'gini', 'max_depth': 2, 'max_features': 2, 'min_samples_leaf': 5, 'n_estimators': 500, 'n_jobs': -1, 'random_state': 7}	3	14	10	7	0	9	8	2	13	4	5	11	16	1	6	15
8	RF, PI	{'criterion': 'gini', 'max_depth': 2, 'max_features': 2, 'min_samples_leaf': 5, 'n_estimators': 500, 'n_jobs': -1, 'random_state': 8}	7	16	10	9	0	8	3	4	14	2	5	11	13	1	6	15
9	RF, PI	{'criterion': 'gini', 'max_depth': 2, 'max_features': 2, 'min_samples_leaf': 5, 'n_estimators': 500, 'n_jobs': -1, 'random_state': 9}	4	14	11	10	1	7	8	2	12	6	5	15	16	0	3	13
10	RF, PI	{'criterion': 'gini', 'max_depth': 2, 'max_features': 2, 'min_samples_leaf': 5, 'n_estimators': 500, 'n_jobs': -1, 'random_state': 10}	9	14	10	8	1	7	5	3	13	4	2	12	16	0	6	15
11	RF, PI	{'criterion': 'gini', 'max_depth': 2, 'max_features': 2, 'min_samples_leaf': 5, 'n_estimators': 500, 'n_jobs': -1, 'random_state': 11}	7	14	13	6	0	8	9	2	12	4	3	10	16	1	5	15
12	RF, PI	{'criterion': 'gini', 'max_depth': 2, 'max_features': 2, 'min_samples_leaf': 5, 'n_estimators': 500, 'n_jobs': -1, 'random_state': 12}	3	14	10	7	1	9	8	5	15	4	2	12	16	0	6	13
13	RF, PI	{'criterion': 'gini', 'max_depth': 2, 'max_features': 2, 'min_samples_leaf': 5, 'n_estimators': 500, 'n_jobs': -1, 'random_state': 13}	6	14	11	4	1	7	8	2	13	5	9	10	16	0	3	15
14	RF, PI	{'criterion': 'gini', 'max_depth': 2, 'max_features': 2, 'min_samples_leaf': 5, 'n_estimators': 500, 'n_jobs': -1, 'random_state': 14}	9	15	10	6	1	7	4	2	14	3	5	11	16	0	8	13

15	RF, PI	{'criterion': 'gini', 'max_depth': 2, 'max_features': 2, 'min_samples_leaf': 5, 'n_estimators': 500, 'n_jobs': -1, 'random_state': 15}	6	15	11	9	1	8	7	3	12	4	2	10	14	0	5	16
16	RF, PI	{'criterion': 'gini', 'max_depth': 2, 'max_features': 2, 'min_samples_leaf': 5, 'n_estimators': 500, 'n_jobs': -1, 'random_state': 16}	6	14	10	9	0	8	2	4	11	3	5	15	16	1	7	13
17	RF, PI	{'criterion': 'gini', 'max_depth': 2, 'max_features': 2, 'min_samples_leaf': 5, 'n_estimators': 500, 'n_jobs': -1, 'random_state': 17}	7	14	10	5	0	9	8	2	13	4	6	11	16	1	3	15
18	RF, PI	{'criterion': 'gini', 'max_depth': 2, 'max_features': 2, 'min_samples_leaf': 5, 'n_estimators': 500, 'n_jobs': -1, 'random_state': 18}	8	15	10	2	1	9	5	3	13	7	4	12	16	0	6	14
19	RF, PI	{'criterion': 'gini', 'max_depth': 2, 'max_features': 2, 'min_samples_leaf': 5, 'n_estimators': 500, 'n_jobs': -1, 'random_state': 19}	6	14	10	5	0	8	9	2	13	3	4	11	16	1	7	15
20	RF, PI	{'criterion': 'gini', 'max_depth': 2, 'max_features': 2, 'min_samples_leaf': 5, 'n_estimators': 500, 'n_jobs': -1, 'random_state': 20}	4	13	10	6	1	9	8	2	14	3	5	11	16	0	7	15
21	RF, PI	{'criterion': 'gini', 'max_depth': 2, 'max_features': 2, 'min_samples_leaf': 5, 'n_estimators': 500, 'n_jobs': -1, 'random_state': 21}	9	13	11	5	1	6	8	7	15	2	3	10	16	0	4	14
22	RF, PI	{'criterion': 'gini', 'max_depth': 2, 'max_features': 2, 'min_samples_leaf': 5, 'n_estimators': 500, 'n_jobs': -1, 'random_state': 22}	4	15	10	9	0	6	3	8	13	2	5	11	16	1	7	14
23	RF, PI	{'criterion': 'gini', 'max_depth': 2, 'max_features': 2, 'min_samples_leaf': 5, 'n_estimators': 500, 'n_jobs': -1, 'random_state': 23}	5	15	11	7	0	12	6	3	13	4	2	10	16	1	8	14
24	RF, PI	{'criterion': 'gini', 'max_depth': 2, 'max_features': 2, 'min_samples_leaf': 5, 'n_estimators': 500, 'n_jobs': -1, 'random_state': 24}	5	14	10	8	1	9	4	3	13	2	6	12	16	0	7	15

25	RF, PI	{'criterion': 'gini', 'max_depth': 2, 'max_features': 2, 'min_samples_leaf': 5, 'n_estimators': 500, 'n_jobs': -1, 'random_state': 25}	5	14	10	9	0	7	2	3	13	6	8	12	16	1	4	15
26	RF, PI	{'criterion': 'gini', 'max_depth': 2, 'max_features': 2, 'min_samples_leaf': 5, 'n_estimators': 500, 'n_jobs': -1, 'random_state': 26}	6	15	11	7	0	10	5	3	13	8	2	9	16	1	4	14
27	RF, PI	{'criterion': 'gini', 'max_depth': 2, 'max_features': 2, 'min_samples_leaf': 5, 'n_estimators': 500, 'n_jobs': -1, 'random_state': 27}	4	15	10	9	1	8	6	5	12	2	3	14	16	0	7	13
28	RF, PI	{'criterion': 'gini', 'max_depth': 2, 'max_features': 2, 'min_samples_leaf': 5, 'n_estimators': 500, 'n_jobs': -1, 'random_state': 28}	6	15	10	3	1	9	8	2	13	5	4	11	16	0	7	14
29	RF, PI	{'criterion': 'gini', 'max_depth': 2, 'max_features': 2, 'min_samples_leaf': 5, 'n_estimators': 500, 'n_jobs': -1, 'random_state': 29}	5	14	12	8	1	6	7	2	13	3	4	10	16	0	9	15
30	RF, PI	{'criterion': 'gini', 'max_depth': 2, 'max_features': 2, 'min_samples_leaf': 5, 'n_estimators': 500, 'n_jobs': -1, 'random_state': 30}	7	15	13	4	1	9	8	2	11	3	5	12	16	0	6	14
31	RF, PI	{'criterion': 'gini', 'max_depth': 2, 'max_features': 2, 'min_samples_leaf': 5, 'n_estimators': 500, 'n_jobs': -1, 'random_state': 31}	7	14	10	4	0	8	5	6	16	3	2	11	15	1	9	13
32	RF, PI	{'criterion': 'gini', 'max_depth': 2, 'max_features': 2, 'min_samples_leaf': 5, 'n_estimators': 500, 'n_jobs': -1, 'random_state': 32}	4	15	10	5	1	9	6	3	13	8	2	11	16	0	7	14
33	RF, PI	{'criterion': 'gini', 'max_depth': 2, 'max_features': 2, 'min_samples_leaf': 5, 'n_estimators': 500, 'n_jobs': -1, 'random_state': 33}	6	13	10	3	1	9	8	4	12	2	5	15	16	0	7	14
34	RF, PI	{'criterion': 'gini', 'max_depth': 2, 'max_features': 2, 'min_samples_leaf': 5, 'n_estimators': 500, 'n_jobs': -1, 'random_state': 34}	7	14	12	5	0	9	4	3	13	2	6	10	16	1	8	15

35	RF, PI	{'criterion': 'gini', 'max_depth': 2, 'max_features': 2, 'min_samples_leaf': 5, 'n_estimators': 500, 'n_jobs': -1, 'random_state': 35}	2	13	7	9	0	10	6	3	14	4	8	11	16	1	5	15
36	RF, PI	{'criterion': 'gini', 'max_depth': 2, 'max_features': 2, 'min_samples_leaf': 5, 'n_estimators': 500, 'n_jobs': -1, 'random_state': 36}	6	16	11	8	0	9	5	2	13	3	4	10	15	1	7	14
37	RF, PI	{'criterion': 'gini', 'max_depth': 2, 'max_features': 2, 'min_samples_leaf': 5, 'n_estimators': 500, 'n_jobs': -1, 'random_state': 37}	2	15	10	7	0	9	6	3	13	4	8	11	16	1	5	14
38	RF, PI	{'criterion': 'gini', 'max_depth': 2, 'max_features': 2, 'min_samples_leaf': 5, 'n_estimators': 500, 'n_jobs': -1, 'random_state': 38}	4	14	12	7	0	9	3	2	13	5	6	10	16	1	8	15
39	RF, PI	{'criterion': 'gini', 'max_depth': 2, 'max_features': 2, 'min_samples_leaf': 5, 'n_estimators': 500, 'n_jobs': -1, 'random_state': 39}	5	15	8	10	0	9	6	3	13	2	4	11	16	1	7	14
40	RF, PI	{'criterion': 'gini', 'max_depth': 2, 'max_features': 2, 'min_samples_leaf': 5, 'n_estimators': 500, 'n_jobs': -1, 'random_state': 40}	5	14	8	10	1	9	4	7	13	2	3	11	15	0	6	16
41	RF, PI	{'criterion': 'gini', 'max_depth': 2, 'max_features': 2, 'min_samples_leaf': 5, 'n_estimators': 500, 'n_jobs': -1, 'random_state': 41}	3	14	9	10	0	8	7	2	13	4	6	11	16	1	5	15
42	RF, PI	{'criterion': 'gini', 'max_depth': 2, 'max_features': 2, 'min_samples_leaf': 5, 'n_estimators': 500, 'n_jobs': -1, 'random_state': 42}	4	15	10	8	1	7	9	2	12	3	6	14	16	0	5	13
43	RF, PI	{'criterion': 'gini', 'max_depth': 2, 'max_features': 2, 'min_samples_leaf': 5, 'n_estimators': 500, 'n_jobs': -1, 'random_state': 43}	7	15	10	8	0	9	5	2	12	4	3	11	16	1	6	14
44	RF, PI	{'criterion': 'gini', 'max_depth': 2, 'max_features': 2, 'min_samples_leaf': 5, 'n_estimators': 500, 'n_jobs': -1, 'random_state': 44}	5	14	11	4	1	9	8	2	13	3	6	12	16	0	7	15

45	RF, PI	{'criterion': 'gini', 'max_depth': 2, 'max_features': 2, 'min_samples_leaf': 5, 'n_estimators': 500, 'n_jobs': -1, 'random_state': 45}	8	15	10	2	1	9	4	3	11	6	5	12	16	0	7	14
46	RF, PI	{'criterion': 'gini', 'max_depth': 2, 'max_features': 2, 'min_samples_leaf': 5, 'n_estimators': 500, 'n_jobs': -1, 'random_state': 46}	2	16	9	7	1	11	3	4	13	6	5	10	14	0	8	15
47	RF, PI	{'criterion': 'gini', 'max_depth': 2, 'max_features': 2, 'min_samples_leaf': 5, 'n_estimators': 500, 'n_jobs': -1, 'random_state': 47}	5	13	10	8	0	9	3	2	12	7	4	14	16	1	6	15
48	RF, PI	{'criterion': 'gini', 'max_depth': 2, 'max_features': 2, 'min_samples_leaf': 5, 'n_estimators': 500, 'n_jobs': -1, 'random_state': 48}	5	16	10	8	1	9	6	3	14	2	7	11	15	0	4	13
49	RF, PI	{'criterion': 'gini', 'max_depth': 2, 'max_features': 2, 'min_samples_leaf': 5, 'n_estimators': 500, 'n_jobs': -1, 'random_state': 49}	3	14	9	8	0	11	5	2	13	7	4	10	16	1	6	15
50	RF, PI	{'criterion': 'gini', 'max_depth': 2, 'max_features': 2, 'min_samples_leaf': 5, 'n_estimators': 500, 'n_jobs': -1, 'random_state': 50}	7	15	12	2	1	8	5	3	13	6	4	10	16	0	9	14
51	RF, PI	{'criterion': 'gini', 'max_depth': 2, 'max_features': 2, 'min_samples_leaf': 5, 'n_estimators': 500, 'n_jobs': -1, 'random_state': 51}	5	15	9	6	1	10	8	2	13	3	4	11	16	0	7	14
52	RF, PI	{'criterion': 'gini', 'max_depth': 2, 'max_features': 2, 'min_samples_leaf': 5, 'n_estimators': 500, 'n_jobs': -1, 'random_state': 52}	6	13	9	3	0	10	8	2	14	4	5	12	16	1	7	15
53	RF, PI	{'criterion': 'gini', 'max_depth': 2, 'max_features': 2, 'min_samples_leaf': 5, 'n_estimators': 500, 'n_jobs': -1, 'random_state': 53}	4	15	13	9	0	6	8	7	11	2	5	10	16	1	3	14
54	RF, PI	{'criterion': 'gini', 'max_depth': 2, 'max_features': 2, 'min_samples_leaf': 5, 'n_estimators': 500, 'n_jobs': -1, 'random_state': 54}	4	15	11	6	0	8	9	5	14	3	2	10	13	1	7	16

55	RF, PI	{'criterion': 'gini', 'max_depth': 2, 'max_features': 2, 'min_samples_leaf': 5, 'n_estimators': 500, 'n_jobs': -1, 'random_state': 55}	7	14	11	8	1	9	4	3	13	2	5	12	16	0	6	15
56	RF, PI	{'criterion': 'gini', 'max_depth': 2, 'max_features': 2, 'min_samples_leaf': 5, 'n_estimators': 500, 'n_jobs': -1, 'random_state': 56}	5	13	10	9	1	8	3	2	14	4	6	12	16	0	7	15
57	RF, PI	{'criterion': 'gini', 'max_depth': 2, 'max_features': 2, 'min_samples_leaf': 5, 'n_estimators': 500, 'n_jobs': -1, 'random_state': 57}	5	15	11	8	1	9	7	4	14	2	3	10	16	0	6	13
58	RF, PI	{'criterion': 'gini', 'max_depth': 2, 'max_features': 2, 'min_samples_leaf': 5, 'n_estimators': 500, 'n_jobs': -1, 'random_state': 58}	3	15	9	8	0	6	10	2	12	4	7	11	16	1	5	14
59	RF, PI	{'criterion': 'gini', 'max_depth': 2, 'max_features': 2, 'min_samples_leaf': 5, 'n_estimators': 500, 'n_jobs': -1, 'random_state': 59}	4	12	10	5	1	9	8	2	13	3	6	15	16	0	7	14
60	RF, PI	{'criterion': 'gini', 'max_depth': 2, 'max_features': 2, 'min_samples_leaf': 5, 'n_estimators': 500, 'n_jobs': -1, 'random_state': 60}	6	14	10	8	0	9	4	3	11	5	2	13	16	1	7	15
61	RF, PI	{'criterion': 'gini', 'max_depth': 2, 'max_features': 2, 'min_samples_leaf': 5, 'n_estimators': 500, 'n_jobs': -1, 'random_state': 61}	3	14	8	12	1	10	7	2	13	6	4	11	16	0	5	15
62	RF, PI	{'criterion': 'gini', 'max_depth': 2, 'max_features': 2, 'min_samples_leaf': 5, 'n_estimators': 500, 'n_jobs': -1, 'random_state': 62}	4	13	9	8	1	10	7	2	14	3	5	12	16	0	6	15
63	RF, PI	{'criterion': 'gini', 'max_depth': 2, 'max_features': 2, 'min_samples_leaf': 5, 'n_estimators': 500, 'n_jobs': -1, 'random_state': 63}	3	14	11	8	0	7	6	4	13	2	5	10	16	1	9	15
64	RF, PI	{'criterion': 'gini', 'max_depth': 2, 'max_features': 2, 'min_samples_leaf': 5, 'n_estimators': 500, 'n_jobs': -1, 'random_state': 64}	8	15	9	7	0	10	5	2	14	3	4	11	13	1	6	16

65	RF, PI	{'criterion': 'gini', 'max_depth': 2, 'max_features': 2, 'min_samples_leaf': 5, 'n_estimators': 500, 'n_jobs': -1, 'random_state': 65}	4	15	10	8	1	9	7	2	12	5	3	11	16	0	6	14
66	RF, PI	{'criterion': 'gini', 'max_depth': 2, 'max_features': 2, 'min_samples_leaf': 5, 'n_estimators': 500, 'n_jobs': -1, 'random_state': 66}	4	14	10	3	0	8	9	5	13	2	6	12	16	1	7	15
67	RF, PI	{'criterion': 'gini', 'max_depth': 2, 'max_features': 2, 'min_samples_leaf': 5, 'n_estimators': 500, 'n_jobs': -1, 'random_state': 67}	4	14	10	5	0	9	6	2	12	3	7	15	16	1	8	13
68	RF, PI	{'criterion': 'gini', 'max_depth': 2, 'max_features': 2, 'min_samples_leaf': 5, 'n_estimators': 500, 'n_jobs': -1, 'random_state': 68}	6	15	12	8	1	9	4	2	14	3	7	10	16	0	5	13
69	RF, PI	{'criterion': 'gini', 'max_depth': 2, 'max_features': 2, 'min_samples_leaf': 5, 'n_estimators': 500, 'n_jobs': -1, 'random_state': 69}	6	15	9	4	1	10	8	2	14	3	7	12	16	0	5	13
70	RF, PI	{'criterion': 'gini', 'max_depth': 2, 'max_features': 2, 'min_samples_leaf': 5, 'n_estimators': 500, 'n_jobs': -1, 'random_state': 70}	5	15	10	8	1	9	2	3	13	4	6	11	16	0	7	14
71	RF, PI	{'criterion': 'gini', 'max_depth': 2, 'max_features': 2, 'min_samples_leaf': 5, 'n_estimators': 500, 'n_jobs': -1, 'random_state': 71}	4	14	9	12	1	6	8	3	13	7	2	10	16	0	5	15
72	RF, PI	{'criterion': 'gini', 'max_depth': 2, 'max_features': 2, 'min_samples_leaf': 5, 'n_estimators': 500, 'n_jobs': -1, 'random_state': 72}	9	14	12	8	1	7	5	3	13	4	2	10	16	0	6	15
73	RF, PI	{'criterion': 'gini', 'max_depth': 2, 'max_features': 2, 'min_samples_leaf': 5, 'n_estimators': 500, 'n_jobs': -1, 'random_state': 73}	6	14	10	5	0	9	8	2	12	3	4	13	16	1	7	15
74	RF, PI	{'criterion': 'gini', 'max_depth': 2, 'max_features': 2, 'min_samples_leaf': 5, 'n_estimators': 500, 'n_jobs': -1, 'random_state': 74}	4	15	10	2	1	9	7	5	13	3	8	14	16	0	6	12

75	RF, PI	{'criterion': 'gini', 'max_depth': 2, 'max_features': 2, 'min_samples_leaf': 5, 'n_estimators': 500, 'n_jobs': -1, 'random_state': 75}	7	13	9	2	1	10	6	4	12	3	5	11	16	0	8	15
76	RF, PI	{'criterion': 'gini', 'max_depth': 2, 'max_features': 2, 'min_samples_leaf': 5, 'n_estimators': 500, 'n_jobs': -1, 'random_state': 76}	2	13	10	8	1	9	7	3	12	5	4	15	16	0	6	14
77	RF, PI	{'criterion': 'gini', 'max_depth': 2, 'max_features': 2, 'min_samples_leaf': 5, 'n_estimators': 500, 'n_jobs': -1, 'random_state': 77}	4	13	12	7	1	9	5	3	14	2	8	10	16	0	6	15
78	RF, PI	{'criterion': 'gini', 'max_depth': 2, 'max_features': 2, 'min_samples_leaf': 5, 'n_estimators': 500, 'n_jobs': -1, 'random_state': 78}	5	15	11	4	0	9	6	3	13	2	7	10	16	1	8	14
79	RF, PI	{'criterion': 'gini', 'max_depth': 2, 'max_features': 2, 'min_samples_leaf': 5, 'n_estimators': 500, 'n_jobs': -1, 'random_state': 79}	7	16	11	5	0	9	8	2	13	3	6	10	15	1	4	14
80	RF, PI	{'criterion': 'gini', 'max_depth': 2, 'max_features': 2, 'min_samples_leaf': 5, 'n_estimators': 500, 'n_jobs': -1, 'random_state': 80}	5	15	12	6	1	9	4	3	11	2	7	14	16	0	8	13
81	RF, PI	{'criterion': 'gini', 'max_depth': 2, 'max_features': 2, 'min_samples_leaf': 5, 'n_estimators': 500, 'n_jobs': -1, 'random_state': 81}	4	15	9	3	0	10	8	6	13	7	2	11	16	1	5	14
82	RF, PI	{'criterion': 'gini', 'max_depth': 2, 'max_features': 2, 'min_samples_leaf': 5, 'n_estimators': 500, 'n_jobs': -1, 'random_state': 82}	3	14	10	8	1	9	5	2	13	4	7	12	16	0	6	15
83	RF, PI	{'criterion': 'gini', 'max_depth': 2, 'max_features': 2, 'min_samples_leaf': 5, 'n_estimators': 500, 'n_jobs': -1, 'random_state': 83}	4	15	11	8	0	9	6	2	13	3	5	10	16	1	7	14
84	RF, PI	{'criterion': 'gini', 'max_depth': 2, 'max_features': 2, 'min_samples_leaf': 5, 'n_estimators': 500, 'n_jobs': -1, 'random_state': 84}	6	14	9	10	0	7	8	2	13	5	4	11	16	1	3	15

85	RF, PI	{'criterion': 'gini', 'max_depth': 2, 'max_features': 2, 'min_samples_leaf': 5, 'n_estimators': 500, 'n_jobs': -1, 'random_state': 85}	4	14	10	9	1	8	7	3	13	5	6	11	16	0	2	15
86	RF, PI	{'criterion': 'gini', 'max_depth': 2, 'max_features': 2, 'min_samples_leaf': 5, 'n_estimators': 500, 'n_jobs': -1, 'random_state': 86}	5	14	9	7	1	10	8	2	13	3	6	12	16	0	4	15
87	RF, PI	{'criterion': 'gini', 'max_depth': 2, 'max_features': 2, 'min_samples_leaf': 5, 'n_estimators': 500, 'n_jobs': -1, 'random_state': 87}	6	13	9	8	0	10	7	2	14	4	3	12	16	1	5	15
88	RF, PI	{'criterion': 'gini', 'max_depth': 2, 'max_features': 2, 'min_samples_leaf': 5, 'n_estimators': 500, 'n_jobs': -1, 'random_state': 88}	7	14	10	4	0	11	6	2	13	3	8	9	16	1	5	15
89	RF, PI	{'criterion': 'gini', 'max_depth': 2, 'max_features': 2, 'min_samples_leaf': 5, 'n_estimators': 500, 'n_jobs': -1, 'random_state': 89}	5	16	12	8	0	10	6	3	13	2	4	9	15	1	7	14
90	RF, PI	{'criterion': 'gini', 'max_depth': 2, 'max_features': 2, 'min_samples_leaf': 5, 'n_estimators': 500, 'n_jobs': -1, 'random_state': 90}	6	15	12	7	1	10	8	2	14	4	5	9	16	0	3	13
91	RF, PI	{'criterion': 'gini', 'max_depth': 2, 'max_features': 2, 'min_samples_leaf': 5, 'n_estimators': 500, 'n_jobs': -1, 'random_state': 91}	4	15	10	8	1	9	6	3	12	2	7	13	16	0	5	14
92	RF, PI	{'criterion': 'gini', 'max_depth': 2, 'max_features': 2, 'min_samples_leaf': 5, 'n_estimators': 500, 'n_jobs': -1, 'random_state': 92}	6	13	11	3	0	9	2	4	15	7	5	10	16	1	8	14
93	RF, PI	{'criterion': 'gini', 'max_depth': 2, 'max_features': 2, 'min_samples_leaf': 5, 'n_estimators': 500, 'n_jobs': -1, 'random_state': 93}	6	15	10	4	1	7	8	3	13	5	2	11	16	0	9	14
94	RF, PI	{'criterion': 'gini', 'max_depth': 2, 'max_features': 2, 'min_samples_leaf': 5, 'n_estimators': 500, 'n_jobs': -1, 'random_state': 94}	4	13	10	2	0	6	7	3	14	8	9	11	16	1	5	15

95	RF, PI	{'criterion': 'gini', 'max_depth': 2, 'max_features': 2, 'min_samples_leaf': 5, 'n_estimators': 500, 'n_jobs': -1, 'random_state': 95}	6	14	11	8	0	10	7	2	13	3	4	9	16	1	5	15
96	RF, PI	{'criterion': 'gini', 'max_depth': 2, 'max_features': 2, 'min_samples_leaf': 5, 'n_estimators': 500, 'n_jobs': -1, 'random_state': 96}	7	16	9	4	0	10	5	2	12	3	6	11	14	1	8	15
97	RF, PI	{'criterion': 'gini', 'max_depth': 2, 'max_features': 2, 'min_samples_leaf': 5, 'n_estimators': 500, 'n_jobs': -1, 'random_state': 97}	4	14	10	5	0	9	8	2	13	3	6	12	16	1	7	15
98	RF, PI	{'criterion': 'gini', 'max_depth': 2, 'max_features': 2, 'min_samples_leaf': 5, 'n_estimators': 500, 'n_jobs': -1, 'random_state': 98}	8	15	11	4	0	9	5	2	13	7	3	10	16	1	6	14
99	RF, PI	{'criterion': 'gini', 'max_depth': 2, 'max_features': 2, 'min_samples_leaf': 5, 'n_estimators': 500, 'n_jobs': -1, 'random_state': 99}	9	14	11	3	1	8	6	2	13	5	4	10	16	0	7	15
0	XGB, PI	{'colsample_bytree': 0.6, 'gamma': 0.5, 'max_depth': 2, 'min_child_weight': 5, 'random_state': 0, 'subsample': 0.6}	1	13	10	2	0	5	3	4	12	9	8	11	15	7	6	16
1	XGB, PI	{'colsample_bytree': 0.6, 'gamma': 0.5, 'max_depth': 2, 'min_child_weight': 5, 'random_state': 1, 'subsample': 0.6}	1	16	7	3	0	6	2	4	13	9	10	12	14	5	8	15
2	XGB, PI	{'colsample_bytree': 0.6, 'gamma': 0.5, 'max_depth': 2, 'min_child_weight': 5, 'random_state': 2, 'subsample': 0.6}	1	15	11	2	0	3	4	6	12	7	10	9	14	8	5	16
3	XGB, PI	{'colsample_bytree': 0.6, 'gamma': 0.5, 'max_depth': 2, 'min_child_weight': 5, 'random_state': 3, 'subsample': 0.6}	2	16	7	1	0	6	3	8	12	11	9	10	14	5	4	15
4	XGB, PI	{'colsample_bytree': 0.6, 'gamma': 0.5, 'max_depth': 2, 'min_child_weight': 5, 'random_state': 4, 'subsample': 0.6}	0	13	10	1	3	2	4	5	14	8	12	6	16	9	7	15
5	XGB, PI	{'colsample_bytree': 0.6, 'gamma': 0.5, 'max_depth': 2, 'min_child_weight': 5, 'random_state': 5, 'subsample': 0.6}	2	16	8	1	0	3	4	5	13	10	12	7	11	9	6	15
6	XGB, PI	{'colsample_bytree': 0.6, 'gamma': 0.5, 'max_depth': 2, 'min_child_weight': 5, 'random_state': 6, 'subsample': 0.6}	5	12	10	4	0	2	1	3	16	8	9	11	13	7	6	14
7	XGB, PI	{'colsample_bytree': 0.6, 'gamma': 0.5, 'max_depth': 2, 'min_child_weight': 5, 'random_state': 7, 'subsample': 0.6}	0	15	7	2	1	5	3	4	16	8	10	11	12	9	6	13

8	XGB, PI	{'colsample_bytree': 0.6, 'gamma': 0.5, 'max_depth': 2, 'min_child_weight': 5, 'random_state': 8, 'subsample': 0.6}	1	15	9	2	0	4	3	8	12	11	10	7	13	6	5	16
9	XGB, PI	{'colsample_bytree': 0.6, 'gamma': 0.5, 'max_depth': 2, 'min_child_weight': 5, 'random_state': 9, 'subsample': 0.6}	1	13	6	2	0	3	4	8	10	9	11	15	12	5	7	16
10	XGB, PI	{'colsample_bytree': 0.6, 'gamma': 0.5, 'max_depth': 2, 'min_child_weight': 5, 'random_state': 10, 'subsample': 0.6}	1	12	7	2	0	5	3	4	13	10	8	11	16	9	6	14
11	XGB, PI	{'colsample_bytree': 0.6, 'gamma': 0.5, 'max_depth': 2, 'min_child_weight': 5, 'random_state': 11, 'subsample': 0.6}	1	15	5	3	0	6	8	4	12	10	9	13	14	7	2	16
12	XGB, PI	{'colsample_bytree': 0.6, 'gamma': 0.5, 'max_depth': 2, 'min_child_weight': 5, 'random_state': 12, 'subsample': 0.6}	1	15	6	2	0	4	3	7	12	9	10	13	16	8	5	14
13	XGB, PI	{'colsample_bytree': 0.6, 'gamma': 0.5, 'max_depth': 2, 'min_child_weight': 5, 'random_state': 13, 'subsample': 0.6}	1	14	12	2	0	8	3	7	16	6	10	9	13	5	4	15
14	XGB, PI	{'colsample_bytree': 0.6, 'gamma': 0.5, 'max_depth': 2, 'min_child_weight': 5, 'random_state': 14, 'subsample': 0.6}	1	16	7	5	0	2	4	3	13	9	10	11	12	8	6	15
15	XGB, PI	{'colsample_bytree': 0.6, 'gamma': 0.5, 'max_depth': 2, 'min_child_weight': 5, 'random_state': 15, 'subsample': 0.6}	1	12	11	0	2	7	5	4	14	8	9	10	15	6	3	16
16	XGB, PI	{'colsample_bytree': 0.6, 'gamma': 0.5, 'max_depth': 2, 'min_child_weight': 5, 'random_state': 16, 'subsample': 0.6}	3	13	10	1	0	5	2	4	15	11	9	6	12	7	8	14
17	XGB, PI	{'colsample_bytree': 0.6, 'gamma': 0.5, 'max_depth': 2, 'min_child_weight': 5, 'random_state': 17, 'subsample': 0.6}	1	12	8	4	0	2	5	6	15	7	9	10	13	11	3	16
18	XGB, PI	{'colsample_bytree': 0.6, 'gamma': 0.5, 'max_depth': 2, 'min_child_weight': 5, 'random_state': 18, 'subsample': 0.6}	1	15	10	3	0	4	2	6	12	11	7	8	14	9	5	16
19	XGB, PI	{'colsample_bytree': 0.6, 'gamma': 0.5, 'max_depth': 2, 'min_child_weight': 5, 'random_state': 19, 'subsample': 0.6}	2	11	7	0	1	5	4	6	15	9	12	10	14	8	3	16
20	XGB, PI	{'colsample_bytree': 0.6, 'gamma': 0.5, 'max_depth': 2, 'min_child_weight': 5, 'random_state': 20, 'subsample': 0.6}	1	15	8	3	0	7	2	10	13	5	9	12	11	6	4	16
21	XGB, PI	{'colsample_bytree': 0.6, 'gamma': 0.5, 'max_depth': 2, 'min_child_weight': 5, 'random_state': 21, 'subsample': 0.6}	1	15	8	3	0	10	2	7	12	4	9	11	16	5	6	14
22	XGB, PI	{'colsample_bytree': 0.6, 'gamma': 0.5, 'max_depth': 2, 'min_child_weight': 5, 'random_state': 22, 'subsample': 0.6}	1	15	11	4	0	5	3	6	14	9	10	8	12	7	2	16

23	XGB, PI	{'colsample_bytree': 0.6, 'gamma': 0.5, 'max_depth': 2, 'min_child_weight': 5, 'random_state': 23, 'subsample': 0.6}	1	14	8	4	0	5	3	7	15	10	9	11	12	2	6	16
24	XGB, PI	{'colsample_bytree': 0.6, 'gamma': 0.5, 'max_depth': 2, 'min_child_weight': 5, 'random_state': 24, 'subsample': 0.6}	0	14	10	3	1	7	2	5	13	6	9	15	11	8	4	16
25	XGB, PI	{'colsample_bytree': 0.6, 'gamma': 0.5, 'max_depth': 2, 'min_child_weight': 5, 'random_state': 25, 'subsample': 0.6}	3	14	10	1	0	4	2	7	12	8	9	11	16	6	5	15
26	XGB, PI	{'colsample_bytree': 0.6, 'gamma': 0.5, 'max_depth': 2, 'min_child_weight': 5, 'random_state': 26, 'subsample': 0.6}	0	12	11	3	1	5	2	4	13	7	10	8	16	9	6	15
27	XGB, PI	{'colsample_bytree': 0.6, 'gamma': 0.5, 'max_depth': 2, 'min_child_weight': 5, 'random_state': 27, 'subsample': 0.6}	0	15	11	2	1	5	4	8	12	10	9	7	13	3	6	16
28	XGB, PI	{'colsample_bytree': 0.6, 'gamma': 0.5, 'max_depth': 2, 'min_child_weight': 5, 'random_state': 28, 'subsample': 0.6}	2	16	9	4	0	1	5	6	13	7	11	12	10	8	3	14
29	XGB, PI	{'colsample_bytree': 0.6, 'gamma': 0.5, 'max_depth': 2, 'min_child_weight': 5, 'random_state': 29, 'subsample': 0.6}	2	16	10	3	0	5	1	9	12	8	11	4	13	6	7	15
30	XGB, PI	{'colsample_bytree': 0.6, 'gamma': 0.5, 'max_depth': 2, 'min_child_weight': 5, 'random_state': 30, 'subsample': 0.6}	1	15	8	3	2	4	5	7	12	11	10	9	14	6	0	16
31	XGB, PI	{'colsample_bytree': 0.6, 'gamma': 0.5, 'max_depth': 2, 'min_child_weight': 5, 'random_state': 31, 'subsample': 0.6}	0	14	6	3	1	2	4	5	12	8	10	11	13	9	7	16
32	XGB, PI	{'colsample_bytree': 0.6, 'gamma': 0.5, 'max_depth': 2, 'min_child_weight': 5, 'random_state': 32, 'subsample': 0.6}	1	14	8	2	0	5	4	6	13	11	9	7	15	10	3	16
33	XGB, PI	{'colsample_bytree': 0.6, 'gamma': 0.5, 'max_depth': 2, 'min_child_weight': 5, 'random_state': 33, 'subsample': 0.6}	1	15	9	2	0	3	5	4	14	8	10	11	13	7	6	16
34	XGB, PI	{'colsample_bytree': 0.6, 'gamma': 0.5, 'max_depth': 2, 'min_child_weight': 5, 'random_state': 34, 'subsample': 0.6}	1	13	5	3	0	2	6	7	12	9	11	10	15	8	4	16
35	XGB, PI	{'colsample_bytree': 0.6, 'gamma': 0.5, 'max_depth': 2, 'min_child_weight': 5, 'random_state': 35, 'subsample': 0.6}	2	15	11	1	0	7	3	4	13	6	10	9	12	8	5	16
36	XGB, PI	{'colsample_bytree': 0.6, 'gamma': 0.5, 'max_depth': 2, 'min_child_weight': 5, 'random_state': 36, 'subsample': 0.6}	1	13	7	5	0	3	2	6	12	9	10	11	14	8	4	16
37	XGB, PI	{'colsample_bytree': 0.6, 'gamma': 0.5, 'max_depth': 2, 'min_child_weight': 5, 'random_state': 37, 'subsample': 0.6}	1	14	10	2	0	3	6	5	12	9	8	11	16	7	4	15

38	XGB, PI	{'colsample_bytree': 0.6, 'gamma': 0.5, 'max_depth': 2, 'min_child_weight': 5, 'random_state': 38, 'subsample': 0.6}	1	16	3	7	0	6	2	4	12	9	10	11	14	8	5	15
39	XGB, PI	{'colsample_bytree': 0.6, 'gamma': 0.5, 'max_depth': 2, 'min_child_weight': 5, 'random_state': 39, 'subsample': 0.6}	2	15	9	3	0	8	7	1	12	6	11	10	13	4	5	16
40	XGB, PI	{'colsample_bytree': 0.6, 'gamma': 0.5, 'max_depth': 2, 'min_child_weight': 5, 'random_state': 40, 'subsample': 0.6}	5	14	8	2	0	3	4	6	13	10	9	11	12	7	1	16
41	XGB, PI	{'colsample_bytree': 0.6, 'gamma': 0.5, 'max_depth': 2, 'min_child_weight': 5, 'random_state': 41, 'subsample': 0.6}	1	15	11	2	0	5	6	3	13	9	10	8	14	7	4	16
42	XGB, PI	{'colsample_bytree': 0.6, 'gamma': 0.5, 'max_depth': 2, 'min_child_weight': 5, 'random_state': 42, 'subsample': 0.6}	3	16	8	1	0	6	4	7	11	10	9	14	13	5	2	15
43	XGB, PI	{'colsample_bytree': 0.6, 'gamma': 0.5, 'max_depth': 2, 'min_child_weight': 5, 'random_state': 43, 'subsample': 0.6}	1	14	8	2	0	4	3	5	13	9	11	10	16	6	7	12
44	XGB, PI	{'colsample_bytree': 0.6, 'gamma': 0.5, 'max_depth': 2, 'min_child_weight': 5, 'random_state': 44, 'subsample': 0.6}	1	15	9	3	0	5	2	4	14	7	10	11	12	6	8	16
45	XGB, PI	{'colsample_bytree': 0.6, 'gamma': 0.5, 'max_depth': 2, 'min_child_weight': 5, 'random_state': 45, 'subsample': 0.6}	2	15	9	1	0	3	5	7	14	8	10	13	11	6	4	16
46	XGB, PI	{'colsample_bytree': 0.6, 'gamma': 0.5, 'max_depth': 2, 'min_child_weight': 5, 'random_state': 46, 'subsample': 0.6}	0	14	9	3	1	5	2	4	13	6	11	10	12	7	8	16
47	XGB, PI	{'colsample_bytree': 0.6, 'gamma': 0.5, 'max_depth': 2, 'min_child_weight': 5, 'random_state': 47, 'subsample': 0.6}	2	14	10	4	0	1	3	6	12	7	11	9	16	8	5	15
48	XGB, PI	{'colsample_bytree': 0.6, 'gamma': 0.5, 'max_depth': 2, 'min_child_weight': 5, 'random_state': 48, 'subsample': 0.6}	0	14	9	3	1	5	7	4	16	10	12	2	11	8	6	15
49	XGB, PI	{'colsample_bytree': 0.6, 'gamma': 0.5, 'max_depth': 2, 'min_child_weight': 5, 'random_state': 49, 'subsample': 0.6}	0	14	6	3	1	7	2	5	13	9	10	11	15	8	4	16
50	XGB, PI	{'colsample_bytree': 0.6, 'gamma': 0.5, 'max_depth': 2, 'min_child_weight': 5, 'random_state': 50, 'subsample': 0.6}	0	16	11	3	1	2	6	4	13	7	8	10	12	9	5	15
51	XGB, PI	{'colsample_bytree': 0.6, 'gamma': 0.5, 'max_depth': 2, 'min_child_weight': 5, 'random_state': 51, 'subsample': 0.6}	1	15	7	3	0	2	5	6	12	9	8	11	13	10	4	16
52	XGB, PI	{'colsample_bytree': 0.6, 'gamma': 0.5, 'max_depth': 2, 'min_child_weight': 5, 'random_state': 52, 'subsample': 0.6}	1	13	9	3	0	2	4	6	14	8	12	10	11	7	5	16

53	XGB, PI	{'colsample_bytree': 0.6, 'gamma': 0.5, 'max_depth': 2, 'min_child_weight': 5, 'random_state': 53, 'subsample': 0.6}	1	15	7	2	0	3	6	5	14	9	10	11	12	8	4	16
54	XGB, PI	{'colsample_bytree': 0.6, 'gamma': 0.5, 'max_depth': 2, 'min_child_weight': 5, 'random_state': 54, 'subsample': 0.6}	2	14	10	1	0	8	4	5	12	3	9	11	16	7	6	15
55	XGB, PI	{'colsample_bytree': 0.6, 'gamma': 0.5, 'max_depth': 2, 'min_child_weight': 5, 'random_state': 55, 'subsample': 0.6}	2	14	11	1	0	3	4	6	12	8	10	9	16	7	5	15
56	XGB, PI	{'colsample_bytree': 0.6, 'gamma': 0.5, 'max_depth': 2, 'min_child_weight': 5, 'random_state': 56, 'subsample': 0.6}	1	14	8	2	0	4	3	5	15	9	10	12	13	6	7	16
57	XGB, PI	{'colsample_bytree': 0.6, 'gamma': 0.5, 'max_depth': 2, 'min_child_weight': 5, 'random_state': 57, 'subsample': 0.6}	1	12	10	3	0	4	2	6	11	7	9	13	15	8	5	16
58	XGB, PI	{'colsample_bytree': 0.6, 'gamma': 0.5, 'max_depth': 2, 'min_child_weight': 5, 'random_state': 58, 'subsample': 0.6}	1	14	9	3	0	5	4	6	13	7	8	10	12	11	2	16
59	XGB, PI	{'colsample_bytree': 0.6, 'gamma': 0.5, 'max_depth': 2, 'min_child_weight': 5, 'random_state': 59, 'subsample': 0.6}	2	16	9	1	0	5	3	4	12	10	8	11	15	7	6	14
60	XGB, PI	{'colsample_bytree': 0.6, 'gamma': 0.5, 'max_depth': 2, 'min_child_weight': 5, 'random_state': 60, 'subsample': 0.6}	1	15	8	2	0	6	3	4	12	9	11	10	13	7	5	16
61	XGB, PI	{'colsample_bytree': 0.6, 'gamma': 0.5, 'max_depth': 2, 'min_child_weight': 5, 'random_state': 61, 'subsample': 0.6}	2	12	8	1	0	4	3	6	15	7	10	11	13	9	5	16
62	XGB, PI	{'colsample_bytree': 0.6, 'gamma': 0.5, 'max_depth': 2, 'min_child_weight': 5, 'random_state': 62, 'subsample': 0.6}	1	15	2	4	0	3	5	8	13	11	9	10	16	7	6	14
63	XGB, PI	{'colsample_bytree': 0.6, 'gamma': 0.5, 'max_depth': 2, 'min_child_weight': 5, 'random_state': 63, 'subsample': 0.6}	1	16	6	4	0	2	3	8	13	11	9	10	14	7	5	15
64	XGB, PI	{'colsample_bytree': 0.6, 'gamma': 0.5, 'max_depth': 2, 'min_child_weight': 5, 'random_state': 64, 'subsample': 0.6}	1	13	7	2	0	4	3	6	15	10	11	8	14	9	5	16
65	XGB, PI	{'colsample_bytree': 0.6, 'gamma': 0.5, 'max_depth': 2, 'min_child_weight': 5, 'random_state': 65, 'subsample': 0.6}	0	11	8	2	1	5	3	9	12	6	10	14	16	7	4	15
66	XGB, PI	{'colsample_bytree': 0.6, 'gamma': 0.5, 'max_depth': 2, 'min_child_weight': 5, 'random_state': 66, 'subsample': 0.6}	4	13	8	3	0	2	1	6	12	7	10	11	16	9	5	14
67	XGB, PI	{'colsample_bytree': 0.6, 'gamma': 0.5, 'max_depth': 2, 'min_child_weight': 5, 'random_state': 67, 'subsample': 0.6}	0	13	9	5	1	4	3	6	11	8	7	12	15	10	2	16

68	XGB, PI	{'colsample_bytree': 0.6, 'gamma': 0.5, 'max_depth': 2, 'min_child_weight': 5, 'random_state': 68, 'subsample': 0.6}	2	16	6	1	0	5	3	4	14	11	7	9	12	10	8	15
69	XGB, PI	{'colsample_bytree': 0.6, 'gamma': 0.5, 'max_depth': 2, 'min_child_weight': 5, 'random_state': 69, 'subsample': 0.6}	1	14	7	2	0	4	6	3	15	9	10	13	12	8	5	16
70	XGB, PI	{'colsample_bytree': 0.6, 'gamma': 0.5, 'max_depth': 2, 'min_child_weight': 5, 'random_state': 70, 'subsample': 0.6}	1	12	5	2	0	6	4	3	15	9	10	11	14	8	7	16
71	XGB, PI	{'colsample_bytree': 0.6, 'gamma': 0.5, 'max_depth': 2, 'min_child_weight': 5, 'random_state': 71, 'subsample': 0.6}	0	13	4	2	1	6	5	7	14	9	11	10	12	8	3	16
72	XGB, PI	{'colsample_bytree': 0.6, 'gamma': 0.5, 'max_depth': 2, 'min_child_weight': 5, 'random_state': 72, 'subsample': 0.6}	1	15	7	3	0	4	2	5	11	10	6	12	13	8	9	16
73	XGB, PI	{'colsample_bytree': 0.6, 'gamma': 0.5, 'max_depth': 2, 'min_child_weight': 5, 'random_state': 73, 'subsample': 0.6}	3	15	9	1	0	2	5	6	12	11	7	8	13	10	4	16
74	XGB, PI	{'colsample_bytree': 0.6, 'gamma': 0.5, 'max_depth': 2, 'min_child_weight': 5, 'random_state': 74, 'subsample': 0.6}	1	14	7	4	0	6	2	5	12	10	9	11	15	8	3	16
75	XGB, PI	{'colsample_bytree': 0.6, 'gamma': 0.5, 'max_depth': 2, 'min_child_weight': 5, 'random_state': 75, 'subsample': 0.6}	1	14	7	2	0	4	5	9	13	3	10	11	15	8	6	16
76	XGB, PI	{'colsample_bytree': 0.6, 'gamma': 0.5, 'max_depth': 2, 'min_child_weight': 5, 'random_state': 76, 'subsample': 0.6}	1	13	8	4	0	2	3	5	12	6	10	11	16	9	7	15
77	XGB, PI	{'colsample_bytree': 0.6, 'gamma': 0.5, 'max_depth': 2, 'min_child_weight': 5, 'random_state': 77, 'subsample': 0.6}	1	16	9	3	0	2	6	4	12	7	8	11	14	10	5	15
78	XGB, PI	{'colsample_bytree': 0.6, 'gamma': 0.5, 'max_depth': 2, 'min_child_weight': 5, 'random_state': 78, 'subsample': 0.6}	2	13	9	1	0	5	3	6	12	8	11	10	15	7	4	16
79	XGB, PI	{'colsample_bytree': 0.6, 'gamma': 0.5, 'max_depth': 2, 'min_child_weight': 5, 'random_state': 79, 'subsample': 0.6}	1	16	9	4	0	6	2	7	13	8	10	11	14	3	5	15
80	XGB, PI	{'colsample_bytree': 0.6, 'gamma': 0.5, 'max_depth': 2, 'min_child_weight': 5, 'random_state': 80, 'subsample': 0.6}	4	13	10	1	0	5	2	3	12	8	11	7	15	6	9	14
81	XGB, PI	{'colsample_bytree': 0.6, 'gamma': 0.5, 'max_depth': 2, 'min_child_weight': 5, 'random_state': 81, 'subsample': 0.6}	1	14	10	6	0	2	5	4	11	8	13	7	15	9	3	16
82	XGB, PI	{'colsample_bytree': 0.6, 'gamma': 0.5, 'max_depth': 2, 'min_child_weight': 5, 'random_state': 82, 'subsample': 0.6}	2	15	9	3	0	4	1	7	13	11	10	6	14	5	8	16

83	XGB, PI	{'colsample_bytree': 0.6, 'gamma': 0.5, 'max_depth': 2, 'min_child_weight': 5, 'random_state': 83, 'subsample': 0.6}	4	14	9	1	0	3	2	8	13	12	7	6	10	11	5	16
84	XGB, PI	{'colsample_bytree': 0.6, 'gamma': 0.5, 'max_depth': 2, 'min_child_weight': 5, 'random_state': 84, 'subsample': 0.6}	1	13	10	4	0	3	6	2	12	8	11	9	15	7	5	16
85	XGB, PI	{'colsample_bytree': 0.6, 'gamma': 0.5, 'max_depth': 2, 'min_child_weight': 5, 'random_state': 85, 'subsample': 0.6}	2	14	9	3	0	1	4	5	13	8	10	11	15	7	6	16
86	XGB, PI	{'colsample_bytree': 0.6, 'gamma': 0.5, 'max_depth': 2, 'min_child_weight': 5, 'random_state': 86, 'subsample': 0.6}	2	14	7	1	0	5	4	3	11	9	10	12	15	8	6	16
87	XGB, PI	{'colsample_bytree': 0.6, 'gamma': 0.5, 'max_depth': 2, 'min_child_weight': 5, 'random_state': 87, 'subsample': 0.6}	1	15	8	4	0	7	2	5	13	9	10	11	16	6	3	14
88	XGB, PI	{'colsample_bytree': 0.6, 'gamma': 0.5, 'max_depth': 2, 'min_child_weight': 5, 'random_state': 88, 'subsample': 0.6}	1	16	11	2	0	3	4	5	12	9	7	10	14	6	8	15
89	XGB, PI	{'colsample_bytree': 0.6, 'gamma': 0.5, 'max_depth': 2, 'min_child_weight': 5, 'random_state': 89, 'subsample': 0.6}	1	14	11	2	0	4	3	5	13	7	8	9	12	10	6	16
90	XGB, PI	{'colsample_bytree': 0.6, 'gamma': 0.5, 'max_depth': 2, 'min_child_weight': 5, 'random_state': 90, 'subsample': 0.6}	1	14	7	2	0	6	4	5	11	8	10	13	15	9	3	16
91	XGB, PI	{'colsample_bytree': 0.6, 'gamma': 0.5, 'max_depth': 2, 'min_child_weight': 5, 'random_state': 91, 'subsample': 0.6}	0	16	9	3	1	2	4	7	14	8	12	11	13	6	5	15
92	XGB, PI	{'colsample_bytree': 0.6, 'gamma': 0.5, 'max_depth': 2, 'min_child_weight': 5, 'random_state': 92, 'subsample': 0.6}	1	16	12	2	0	7	3	4	14	6	9	10	11	8	5	15
93	XGB, PI	{'colsample_bytree': 0.6, 'gamma': 0.5, 'max_depth': 2, 'min_child_weight': 5, 'random_state': 93, 'subsample': 0.6}	1	13	7	2	0	3	5	4	14	9	10	11	12	8	6	16
94	XGB, PI	{'colsample_bytree': 0.6, 'gamma': 0.5, 'max_depth': 2, 'min_child_weight': 5, 'random_state': 94, 'subsample': 0.6}	1	13	9	2	0	4	3	7	12	10	6	11	14	5	8	16
95	XGB, PI	{'colsample_bytree': 0.6, 'gamma': 0.5, 'max_depth': 2, 'min_child_weight': 5, 'random_state': 95, 'subsample': 0.6}	1	11	9	6	0	2	5	4	10	7	12	16	15	8	3	14
96	XGB, PI	{'colsample_bytree': 0.6, 'gamma': 0.5, 'max_depth': 2, 'min_child_weight': 5, 'random_state': 96, 'subsample': 0.6}	4	13	7	1	0	3	5	9	11	12	10	8	14	6	2	16
97	XGB, PI	{'colsample_bytree': 0.6, 'gamma': 0.5, 'max_depth': 2, 'min_child_weight': 5, 'random_state': 97, 'subsample': 0.6}	2	15	7	1	0	6	5	3	13	8	9	14	11	10	4	16

98	XGB, PI	{'colsample_bytree': 0.6, 'gamma': 0.5, 'max_depth': 2, 'min_child_weight': 5, 'random_state': 98, 'subsample': 0.6}	2	15	9	1	0	4	6	5	13	8	11	10	16	7	3	14
99	XGB, PI	{'colsample_bytree': 0.6, 'gamma': 0.5, 'max_depth': 2, 'min_child_weight': 5, 'random_state': 99, 'subsample': 0.6}	1	12	4	2	0	7	5	3	15	10	9	11	14	8	6	16
0	XGB, RFE	{'colsample_bytree': 0.6, 'gamma': 0.5, 'max_depth': 2, 'min_child_weight': 5, 'random_state': 0, 'subsample': 0.6}	8	16	9	7	6	4	2	3	14	12	5	10	13	1	11	15
1	XGB, RFE	{'colsample_bytree': 0.6, 'gamma': 0.5, 'max_depth': 2, 'min_child_weight': 5, 'random_state': 1, 'subsample': 0.6}	11	14	7	2	6	12	3	9	5	13	1	15	8	4	10	17
2	XGB, RFE	{'colsample_bytree': 0.6, 'gamma': 0.5, 'max_depth': 2, 'min_child_weight': 5, 'random_state': 2, 'subsample': 0.6}	7	14	13	8	1	10	4	3	12	11	6	5	15	2	9	17
3	XGB, RFE	{'colsample_bytree': 0.6, 'gamma': 0.5, 'max_depth': 2, 'min_child_weight': 5, 'random_state': 3, 'subsample': 0.6}	10	7	13	8	5	14	1	2	17	6	3	12	9	4	11	15
4	XGB, RFE	{'colsample_bytree': 0.6, 'gamma': 0.5, 'max_depth': 2, 'min_child_weight': 5, 'random_state': 4, 'subsample': 0.6}	8	13	16	1	3	4	6	2	15	9	11	14	5	7	10	17
5	XGB, RFE	{'colsample_bytree': 0.6, 'gamma': 0.5, 'max_depth': 2, 'min_child_weight': 5, 'random_state': 5, 'subsample': 0.6}	2	16	6	1	9	4	8	7	13	15	14	12	10	3	5	17
6	XGB, RFE	{'colsample_bytree': 0.6, 'gamma': 0.5, 'max_depth': 2, 'min_child_weight': 5, 'random_state': 6, 'subsample': 0.6}	7	12	11	2	1	4	10	6	15	13	8	14	9	3	5	16
7	XGB, RFE	{'colsample_bytree': 0.6, 'gamma': 0.5, 'max_depth': 2, 'min_child_weight': 5, 'random_state': 7, 'subsample': 0.6}	9	14	4	12	1	6	10	2	13	5	7	8	15	3	17	16
8	XGB, RFE	{'colsample_bytree': 0.6, 'gamma': 0.5, 'max_depth': 2, 'min_child_weight': 5, 'random_state': 8, 'subsample': 0.6}	2	13	6	9	5	7	1	8	12	11	4	16	14	3	10	17
9	XGB, RFE	{'colsample_bytree': 0.6, 'gamma': 0.5, 'max_depth': 2, 'min_child_weight': 5, 'random_state': 9, 'subsample': 0.6}	7	14	12	2	1	15	8	3	10	4	5	11	9	6	13	17
10	XGB, RFE	{'colsample_bytree': 0.6, 'gamma': 0.5, 'max_depth': 2, 'min_child_weight': 5, 'random_state': 10, 'subsample': 0.6}	5	11	10	15	4	12	7	3	13	6	14	9	17	2	1	16
11	XGB, RFE	{'colsample_bytree': 0.6, 'gamma': 0.5, 'max_depth': 2, 'min_child_weight': 5, 'random_state': 11, 'subsample': 0.6}	1	14	6	8	3	12	11	9	17	7	5	4	13	10	2	16
12	XGB, RFE	{'colsample_bytree': 0.6, 'gamma': 0.5, 'max_depth': 2, 'min_child_weight': 5, 'random_state': 12, 'subsample': 0.6}	7	17	12	4	1	15	3	14	13	8	5	11	10	2	6	16

13	XGB, RFE	{'colsample_bytree': 0.6, 'gamma': 0.5, 'max_depth': 2, 'min_child_weight': 5, 'random_state': 13, 'subsample': 0.6}	1	16	13	5	9	7	3	4	11	8	6	10	17	2	12	15
14	XGB, RFE	{'colsample_bytree': 0.6, 'gamma': 0.5, 'max_depth': 2, 'min_child_weight': 5, 'random_state': 14, 'subsample': 0.6}	3	14	9	4	1	8	10	16	15	13	2	6	11	5	7	17
15	XGB, RFE	{'colsample_bytree': 0.6, 'gamma': 0.5, 'max_depth': 2, 'min_child_weight': 5, 'random_state': 15, 'subsample': 0.6}	1	8	11	2	4	10	7	3	15	12	9	14	16	5	6	17
16	XGB, RFE	{'colsample_bytree': 0.6, 'gamma': 0.5, 'max_depth': 2, 'min_child_weight': 5, 'random_state': 16, 'subsample': 0.6}	15	17	11	2	1	6	10	9	12	13	7	16	8	3	5	14
17	XGB, RFE	{'colsample_bytree': 0.6, 'gamma': 0.5, 'max_depth': 2, 'min_child_weight': 5, 'random_state': 17, 'subsample': 0.6}	2	11	13	3	9	12	4	8	16	7	14	6	10	1	5	17
18	XGB, RFE	{'colsample_bytree': 0.6, 'gamma': 0.5, 'max_depth': 2, 'min_child_weight': 5, 'random_state': 18, 'subsample': 0.6}	9	17	15	1	4	11	5	8	12	6	7	3	13	2	10	16
19	XGB, RFE	{'colsample_bytree': 0.6, 'gamma': 0.5, 'max_depth': 2, 'min_child_weight': 5, 'random_state': 19, 'subsample': 0.6}	5	15	12	4	1	10	8	7	16	13	6	2	11	3	9	17
20	XGB, RFE	{'colsample_bytree': 0.6, 'gamma': 0.5, 'max_depth': 2, 'min_child_weight': 5, 'random_state': 20, 'subsample': 0.6}	1	15	5	9	2	7	14	8	11	6	12	4	13	3	10	17
21	XGB, RFE	{'colsample_bytree': 0.6, 'gamma': 0.5, 'max_depth': 2, 'min_child_weight': 5, 'random_state': 21, 'subsample': 0.6}	5	17	6	4	3	10	13	7	12	8	11	2	16	1	9	15
22	XGB, RFE	{'colsample_bytree': 0.6, 'gamma': 0.5, 'max_depth': 2, 'min_child_weight': 5, 'random_state': 22, 'subsample': 0.6}	10	12	15	5	2	9	1	7	13	11	6	4	14	3	8	17
23	XGB, RFE	{'colsample_bytree': 0.6, 'gamma': 0.5, 'max_depth': 2, 'min_child_weight': 5, 'random_state': 23, 'subsample': 0.6}	1	15	12	6	2	7	5	4	16	9	11	10	14	3	13	17
24	XGB, RFE	{'colsample_bytree': 0.6, 'gamma': 0.5, 'max_depth': 2, 'min_child_weight': 5, 'random_state': 24, 'subsample': 0.6}	1	14	12	2	4	8	7	5	10	6	13	15	11	3	9	17
25	XGB, RFE	{'colsample_bytree': 0.6, 'gamma': 0.5, 'max_depth': 2, 'min_child_weight': 5, 'random_state': 25, 'subsample': 0.6}	6	15	5	2	1	13	12	9	16	4	11	10	17	3	7	14
26	XGB, RFE	{'colsample_bytree': 0.6, 'gamma': 0.5, 'max_depth': 2, 'min_child_weight': 5, 'random_state': 26, 'subsample': 0.6}	1	16	11	8	7	9	2	6	14	4	10	5	17	3	13	15
27	XGB, RFE	{'colsample_bytree': 0.6, 'gamma': 0.5, 'max_depth': 2, 'min_child_weight': 5, 'random_state': 27, 'subsample': 0.6}	1	16	15	11	5	4	10	2	12	9	6	8	14	3	7	17

28	XGB, RFE	{'colsample_bytree': 0.6, 'gamma': 0.5, 'max_depth': 2, 'min_child_weight': 5, 'random_state': 28, 'subsample': 0.6}	1	16	5	10	2	12	9	6	13	3	8	7	15	4	11	17
29	XGB, RFE	{'colsample_bytree': 0.6, 'gamma': 0.5, 'max_depth': 2, 'min_child_weight': 5, 'random_state': 29, 'subsample': 0.6}	10	12	9	8	2	3	5	11	13	7	4	15	17	1	6	16
30	XGB, RFE	{'colsample_bytree': 0.6, 'gamma': 0.5, 'max_depth': 2, 'min_child_weight': 5, 'random_state': 30, 'subsample': 0.6}	9	14	13	6	2	3	1	7	15	5	12	10	11	4	8	17
31	XGB, RFE	{'colsample_bytree': 0.6, 'gamma': 0.5, 'max_depth': 2, 'min_child_weight': 5, 'random_state': 31, 'subsample': 0.6}	1	15	14	6	5	9	8	2	11	7	12	16	10	3	13	17
32	XGB, RFE	{'colsample_bytree': 0.6, 'gamma': 0.5, 'max_depth': 2, 'min_child_weight': 5, 'random_state': 32, 'subsample': 0.6}	9	14	5	1	2	15	10	3	12	4	6	7	16	8	11	17
33	XGB, RFE	{'colsample_bytree': 0.6, 'gamma': 0.5, 'max_depth': 2, 'min_child_weight': 5, 'random_state': 33, 'subsample': 0.6}	5	16	14	3	1	10	9	2	12	11	4	15	13	7	6	17
34	XGB, RFE	{'colsample_bytree': 0.6, 'gamma': 0.5, 'max_depth': 2, 'min_child_weight': 5, 'random_state': 34, 'subsample': 0.6}	2	14	12	3	4	8	7	10	16	11	5	9	15	1	13	17
35	XGB, RFE	{'colsample_bytree': 0.6, 'gamma': 0.5, 'max_depth': 2, 'min_child_weight': 5, 'random_state': 35, 'subsample': 0.6}	12	15	14	5	2	11	6	3	8	10	4	9	16	1	7	17
36	XGB, RFE	{'colsample_bytree': 0.6, 'gamma': 0.5, 'max_depth': 2, 'min_child_weight': 5, 'random_state': 36, 'subsample': 0.6}	2	17	13	8	1	10	7	5	4	6	9	11	15	3	12	16
37	XGB, RFE	{'colsample_bytree': 0.6, 'gamma': 0.5, 'max_depth': 2, 'min_child_weight': 5, 'random_state': 37, 'subsample': 0.6}	4	17	8	1	12	3	5	9	11	6	7	15	14	2	10	16
38	XGB, RFE	{'colsample_bytree': 0.6, 'gamma': 0.5, 'max_depth': 2, 'min_child_weight': 5, 'random_state': 38, 'subsample': 0.6}	1	16	3	8	7	10	5	6	9	13	14	4	15	2	11	17
39	XGB, RFE	{'colsample_bytree': 0.6, 'gamma': 0.5, 'max_depth': 2, 'min_child_weight': 5, 'random_state': 39, 'subsample': 0.6}	9	13	10	2	1	14	8	3	12	7	15	4	16	5	6	17
40	XGB, RFE	{'colsample_bytree': 0.6, 'gamma': 0.5, 'max_depth': 2, 'min_child_weight': 5, 'random_state': 40, 'subsample': 0.6}	8	16	13	2	3	4	11	6	9	12	1	5	15	7	10	17
41	XGB, RFE	{'colsample_bytree': 0.6, 'gamma': 0.5, 'max_depth': 2, 'min_child_weight': 5, 'random_state': 41, 'subsample': 0.6}	12	17	3	9	1	10	7	6	16	8	4	11	14	2	5	13
42	XGB, RFE	{'colsample_bytree': 0.6, 'gamma': 0.5, 'max_depth': 2, 'min_child_weight': 5, 'random_state': 42, 'subsample': 0.6}	9	13	16	1	3	11	8	7	15	4	6	5	14	2	10	17

43	XGB, RFE	{'colsample_bytree': 0.6, 'gamma': 0.5, 'max_depth': 2, 'min_child_weight': 5, 'random_state': 43, 'subsample': 0.6}	7	15	11	9	3	12	1	5	13	8	10	4	14	2	6	16
44	XGB, RFE	{'colsample_bytree': 0.6, 'gamma': 0.5, 'max_depth': 2, 'min_child_weight': 5, 'random_state': 44, 'subsample': 0.6}	5	16	10	3	2	6	9	4	14	7	12	8	11	1	15	17
45	XGB, RFE	{'colsample_bytree': 0.6, 'gamma': 0.5, 'max_depth': 2, 'min_child_weight': 5, 'random_state': 45, 'subsample': 0.6}	7	16	3	6	2	5	8	4	15	11	9	17	14	1	13	10
46	XGB, RFE	{'colsample_bytree': 0.6, 'gamma': 0.5, 'max_depth': 2, 'min_child_weight': 5, 'random_state': 46, 'subsample': 0.6}	4	16	15	1	8	12	10	3	7	6	14	5	11	2	13	17
47	XGB, RFE	{'colsample_bytree': 0.6, 'gamma': 0.5, 'max_depth': 2, 'min_child_weight': 5, 'random_state': 47, 'subsample': 0.6}	1	17	14	5	10	8	4	7	12	11	6	2	13	3	9	16
48	XGB, RFE	{'colsample_bytree': 0.6, 'gamma': 0.5, 'max_depth': 2, 'min_child_weight': 5, 'random_state': 48, 'subsample': 0.6}	6	14	12	8	2	3	9	5	16	7	10	4	13	1	11	15
49	XGB, RFE	{'colsample_bytree': 0.6, 'gamma': 0.5, 'max_depth': 2, 'min_child_weight': 5, 'random_state': 49, 'subsample': 0.6}	4	16	6	9	7	12	1	3	10	13	8	5	14	2	11	17
50	XGB, RFE	{'colsample_bytree': 0.6, 'gamma': 0.5, 'max_depth': 2, 'min_child_weight': 5, 'random_state': 50, 'subsample': 0.6}	10	17	8	4	1	3	5	7	15	6	12	9	11	2	13	14
51	XGB, RFE	{'colsample_bytree': 0.6, 'gamma': 0.5, 'max_depth': 2, 'min_child_weight': 5, 'random_state': 51, 'subsample': 0.6}	1	16	10	3	2	8	7	5	11	9	4	14	13	6	12	17
52	XGB, RFE	{'colsample_bytree': 0.6, 'gamma': 0.5, 'max_depth': 2, 'min_child_weight': 5, 'random_state': 52, 'subsample': 0.6}	10	12	7	8	2	9	1	3	15	6	11	4	14	5	13	17
53	XGB, RFE	{'colsample_bytree': 0.6, 'gamma': 0.5, 'max_depth': 2, 'min_child_weight': 5, 'random_state': 53, 'subsample': 0.6}	5	17	3	11	2	14	10	7	12	6	8	4	13	1	9	16
54	XGB, RFE	{'colsample_bytree': 0.6, 'gamma': 0.5, 'max_depth': 2, 'min_child_weight': 5, 'random_state': 54, 'subsample': 0.6}	1	14	12	9	2	8	11	6	3	7	15	5	16	4	10	17
55	XGB, RFE	{'colsample_bytree': 0.6, 'gamma': 0.5, 'max_depth': 2, 'min_child_weight': 5, 'random_state': 55, 'subsample': 0.6}	1	16	9	2	5	10	8	7	12	4	14	6	15	3	11	17
56	XGB, RFE	{'colsample_bytree': 0.6, 'gamma': 0.5, 'max_depth': 2, 'min_child_weight': 5, 'random_state': 56, 'subsample': 0.6}	2	15	11	8	1	5	7	9	14	10	4	13	16	3	6	17
57	XGB, RFE	{'colsample_bytree': 0.6, 'gamma': 0.5, 'max_depth': 2, 'min_child_weight': 5, 'random_state': 57, 'subsample': 0.6}	1	10	11	9	2	6	5	7	16	13	3	8	15	4	14	17

58	XGB, RFE	{'colsample_bytree': 0.6, 'gamma': 0.5, 'max_depth': 2, 'min_child_weight': 5, 'random_state': 58, 'subsample': 0.6}	10	16	13	5	1	7	2	12	15	9	4	6	14	3	11	17
59	XGB, RFE	{'colsample_bytree': 0.6, 'gamma': 0.5, 'max_depth': 2, 'min_child_weight': 5, 'random_state': 59, 'subsample': 0.6}	5	17	10	4	1	12	9	3	13	7	8	11	14	2	6	16
60	XGB, RFE	{'colsample_bytree': 0.6, 'gamma': 0.5, 'max_depth': 2, 'min_child_weight': 5, 'random_state': 60, 'subsample': 0.6}	5	9	11	4	1	12	8	3	14	15	2	7	16	6	13	17
61	XGB, RFE	{'colsample_bytree': 0.6, 'gamma': 0.5, 'max_depth': 2, 'min_child_weight': 5, 'random_state': 61, 'subsample': 0.6}	8	14	15	1	2	6	4	5	12	7	11	9	13	3	10	17
62	XGB, RFE	{'colsample_bytree': 0.6, 'gamma': 0.5, 'max_depth': 2, 'min_child_weight': 5, 'random_state': 62, 'subsample': 0.6}	13	17	10	1	4	8	3	6	14	5	12	9	15	2	7	16
63	XGB, RFE	{'colsample_bytree': 0.6, 'gamma': 0.5, 'max_depth': 2, 'min_child_weight': 5, 'random_state': 63, 'subsample': 0.6}	1	17	10	9	3	6	7	11	8	12	4	14	13	2	5	16
64	XGB, RFE	{'colsample_bytree': 0.6, 'gamma': 0.5, 'max_depth': 2, 'min_child_weight': 5, 'random_state': 64, 'subsample': 0.6}	12	16	11	1	3	13	2	5	14	7	8	6	15	4	10	17
65	XGB, RFE	{'colsample_bytree': 0.6, 'gamma': 0.5, 'max_depth': 2, 'min_child_weight': 5, 'random_state': 65, 'subsample': 0.6}	3	13	16	5	4	10	1	7	15	8	6	14	11	2	12	17
66	XGB, RFE	{'colsample_bytree': 0.6, 'gamma': 0.5, 'max_depth': 2, 'min_child_weight': 5, 'random_state': 66, 'subsample': 0.6}	5	15	12	1	4	9	13	3	7	6	8	11	17	2	10	16
67	XGB, RFE	{'colsample_bytree': 0.6, 'gamma': 0.5, 'max_depth': 2, 'min_child_weight': 5, 'random_state': 67, 'subsample': 0.6}	1	13	5	9	2	12	3	11	14	6	8	7	17	10	4	16
68	XGB, RFE	{'colsample_bytree': 0.6, 'gamma': 0.5, 'max_depth': 2, 'min_child_weight': 5, 'random_state': 68, 'subsample': 0.6}	10	17	11	1	7	2	4	5	12	6	13	14	15	3	9	16
69	XGB, RFE	{'colsample_bytree': 0.6, 'gamma': 0.5, 'max_depth': 2, 'min_child_weight': 5, 'random_state': 69, 'subsample': 0.6}	7	15	13	5	1	2	6	4	14	11	8	12	17	3	9	16
70	XGB, RFE	{'colsample_bytree': 0.6, 'gamma': 0.5, 'max_depth': 2, 'min_child_weight': 5, 'random_state': 70, 'subsample': 0.6}	1	14	6	4	7	9	12	2	17	11	5	8	13	3	10	16
71	XGB, RFE	{'colsample_bytree': 0.6, 'gamma': 0.5, 'max_depth': 2, 'min_child_weight': 5, 'random_state': 71, 'subsample': 0.6}	13	11	3	4	1	7	10	12	14	8	5	9	16	2	6	17
72	XGB, RFE	{'colsample_bytree': 0.6, 'gamma': 0.5, 'max_depth': 2, 'min_child_weight': 5, 'random_state': 72, 'subsample': 0.6}	11	14	10	9	4	7	1	3	12	6	2	13	16	5	8	17

73	XGB, RFE	{'colsample_bytree': 0.6, 'gamma': 0.5, 'max_depth': 2, 'min_child_weight': 5, 'random_state': 73, 'subsample': 0.6}	2	13	14	5	7	10	8	6	11	3	12	1	15	4	9	17
74	XGB, RFE	{'colsample_bytree': 0.6, 'gamma': 0.5, 'max_depth': 2, 'min_child_weight': 5, 'random_state': 74, 'subsample': 0.6}	7	15	10	3	5	8	11	4	13	12	16	1	14	2	9	17
75	XGB, RFE	{'colsample_bytree': 0.6, 'gamma': 0.5, 'max_depth': 2, 'min_child_weight': 5, 'random_state': 75, 'subsample': 0.6}	8	16	14	10	1	4	5	3	15	12	6	9	13	2	7	17
76	XGB, RFE	{'colsample_bytree': 0.6, 'gamma': 0.5, 'max_depth': 2, 'min_child_weight': 5, 'random_state': 76, 'subsample': 0.6}	2	15	3	7	10	11	9	4	13	6	5	1	17	8	12	16
77	XGB, RFE	{'colsample_bytree': 0.6, 'gamma': 0.5, 'max_depth': 2, 'min_child_weight': 5, 'random_state': 77, 'subsample': 0.6}	8	16	11	6	1	7	15	4	13	5	3	12	14	2	9	17
78	XGB, RFE	{'colsample_bytree': 0.6, 'gamma': 0.5, 'max_depth': 2, 'min_child_weight': 5, 'random_state': 78, 'subsample': 0.6}	1	14	13	3	2	9	10	7	16	5	12	6	15	4	11	17
79	XGB, RFE	{'colsample_bytree': 0.6, 'gamma': 0.5, 'max_depth': 2, 'min_child_weight': 5, 'random_state': 79, 'subsample': 0.6}	1	17	12	13	4	5	11	10	9	8	3	7	15	2	6	16
80	XGB, RFE	{'colsample_bytree': 0.6, 'gamma': 0.5, 'max_depth': 2, 'min_child_weight': 5, 'random_state': 80, 'subsample': 0.6}	1	16	10	7	6	9	8	5	17	2	15	4	13	3	14	12
81	XGB, RFE	{'colsample_bytree': 0.6, 'gamma': 0.5, 'max_depth': 2, 'min_child_weight': 5, 'random_state': 81, 'subsample': 0.6}	7	12	11	9	2	8	3	4	16	6	14	13	17	1	5	10
82	XGB, RFE	{'colsample_bytree': 0.6, 'gamma': 0.5, 'max_depth': 2, 'min_child_weight': 5, 'random_state': 82, 'subsample': 0.6}	6	15	13	11	2	7	5	10	12	8	9	1	16	4	3	17
83	XGB, RFE	{'colsample_bytree': 0.6, 'gamma': 0.5, 'max_depth': 2, 'min_child_weight': 5, 'random_state': 83, 'subsample': 0.6}	1	16	7	11	3	9	10	5	12	14	4	6	13	2	8	17
84	XGB, RFE	{'colsample_bytree': 0.6, 'gamma': 0.5, 'max_depth': 2, 'min_child_weight': 5, 'random_state': 84, 'subsample': 0.6}	9	14	11	1	10	3	6	5	13	8	12	4	15	2	7	17
85	XGB, RFE	{'colsample_bytree': 0.6, 'gamma': 0.5, 'max_depth': 2, 'min_child_weight': 5, 'random_state': 85, 'subsample': 0.6}	2	14	15	10	1	9	11	4	7	6	13	16	8	3	5	17
86	XGB, RFE	{'colsample_bytree': 0.6, 'gamma': 0.5, 'max_depth': 2, 'min_child_weight': 5, 'random_state': 86, 'subsample': 0.6}	1	15	6	9	2	13	4	7	14	5	10	12	16	3	8	17
87	XGB, RFE	{'colsample_bytree': 0.6, 'gamma': 0.5, 'max_depth': 2, 'min_child_weight': 5, 'random_state': 87, 'subsample': 0.6}	8	14	9	10	1	4	13	6	15	5	12	7	17	2	3	16

88	XGB, RFE	{'colsample_bytree': 0.6, 'gamma': 0.5, 'max_depth': 2, 'min_child_weight': 5, 'random_state': 88, 'subsample': 0.6}	6	16	8	9	4	5	7	3	12	14	11	1	15	2	10	17
89	XGB, RFE	{'colsample_bytree': 0.6, 'gamma': 0.5, 'max_depth': 2, 'min_child_weight': 5, 'random_state': 89, 'subsample': 0.6}	1	15	11	5	2	12	13	7	16	4	9	6	8	3	10	17
90	XGB, RFE	{'colsample_bytree': 0.6, 'gamma': 0.5, 'max_depth': 2, 'min_child_weight': 5, 'random_state': 90, 'subsample': 0.6}	5	12	13	1	3	7	8	4	6	9	16	11	15	2	10	17
91	XGB, RFE	{'colsample_bytree': 0.6, 'gamma': 0.5, 'max_depth': 2, 'min_child_weight': 5, 'random_state': 91, 'subsample': 0.6}	1	17	12	2	7	4	10	13	16	9	15	5	14	3	6	11
92	XGB, RFE	{'colsample_bytree': 0.6, 'gamma': 0.5, 'max_depth': 2, 'min_child_weight': 5, 'random_state': 92, 'subsample': 0.6}	3	16	8	6	2	9	11	5	10	12	13	1	15	4	7	17
93	XGB, RFE	{'colsample_bytree': 0.6, 'gamma': 0.5, 'max_depth': 2, 'min_child_weight': 5, 'random_state': 93, 'subsample': 0.6}	1	14	12	8	9	2	10	3	5	4	15	13	11	6	7	17
94	XGB, RFE	{'colsample_bytree': 0.6, 'gamma': 0.5, 'max_depth': 2, 'min_child_weight': 5, 'random_state': 94, 'subsample': 0.6}	10	16	11	4	3	9	1	8	15	6	5	7	14	2	13	17
95	XGB, RFE	{'colsample_bytree': 0.6, 'gamma': 0.5, 'max_depth': 2, 'min_child_weight': 5, 'random_state': 95, 'subsample': 0.6}	8	14	13	2	5	6	10	3	12	4	9	11	15	1	7	17
96	XGB, RFE	{'colsample_bytree': 0.6, 'gamma': 0.5, 'max_depth': 2, 'min_child_weight': 5, 'random_state': 96, 'subsample': 0.6}	9	16	12	6	2	8	1	4	11	10	5	7	15	3	13	17
97	XGB, RFE	{'colsample_bytree': 0.6, 'gamma': 0.5, 'max_depth': 2, 'min_child_weight': 5, 'random_state': 97, 'subsample': 0.6}	5	12	7	4	1	9	8	3	14	13	6	15	11	2	10	17
98	XGB, RFE	{'colsample_bytree': 0.6, 'gamma': 0.5, 'max_depth': 2, 'min_child_weight': 5, 'random_state': 98, 'subsample': 0.6}	10	17	9	2	1	5	12	3	15	4	8	7	13	6	11	16
99	XGB, RFE	{'colsample_bytree': 0.6, 'gamma': 0.5, 'max_depth': 2, 'min_child_weight': 5, 'random_state': 99, 'subsample': 0.6}	1	11	4	13	2	12	7	5	14	8	6	10	16	3	9	17
0	AVG		8	12	10	5	1	4	3	2	13	7	6	9	15	0	11	16
1	AVG		7	15	8	5	0	9	2	4	11	6	3	10	13	1	12	16
2	AVG		8	13	11	6	0	5	4	2	12	7	3	9	15	1	10	16
3	AVG		6	12	11	7	1	8	5	2	14	4	3	10	13	0	9	16
4	AVG		7	13	12	5	2	4	1	0	16	6	8	10	14	3	9	15
5	AVG		3	16	10	2	1	5	6	4	14	8	9	11	12	0	7	15
6	AVG		9	12	10	6	0	4	3	2	13	5	7	11	14	1	8	16

7	AVG		3	15	8	9	0	5	7	1	14	6	4	10	13	2	11	16
8	AVG		3	14	8	11	0	7	2	6	13	5	4	9	12	1	10	16
9	AVG		6	15	10	3	0	7	4	2	13	8	5	12	11	1	9	16
10	AVG		8	13	10	11	0	7	3	2	14	4	6	9	15	1	5	16
11	AVG		5	15	9	7	0	8	10	1	14	6	3	11	12	2	4	16
12	AVG		2	15	9	6	1	10	7	4	13	3	5	11	14	0	8	16
13	AVG		3	15	12	5	1	6	4	2	14	9	7	8	13	0	10	16
14	AVG		8	16	10	3	0	2	4	5	13	7	6	12	11	1	9	15
15	AVG		3	13	10	4	1	9	7	2	14	6	5	11	12	0	8	16
16	AVG		9	14	11	3	0	6	2	4	15	7	5	10	12	1	8	16
17	AVG		4	12	11	5	0	9	6	2	15	3	10	8	13	1	7	16
18	AVG		8	16	11	2	0	9	3	4	13	7	5	6	12	1	10	15
19	AVG		5	14	11	3	0	6	9	2	15	8	7	4	12	1	10	16
20	AVG		2	14	8	9	0	5	11	3	13	4	7	6	12	1	10	16
21	AVG		7	14	10	3	0	9	8	6	13	2	4	5	16	1	11	15
22	AVG		7	15	11	9	0	8	1	4	14	5	6	3	13	2	10	16
23	AVG		2	14	12	7	0	8	5	3	15	6	4	10	13	1	11	16
24	AVG		5	14	10	6	0	7	4	3	11	2	8	12	15	1	9	16
25	AVG		6	14	11	4	0	9	2	3	16	5	8	10	15	1	7	13
26	AVG		4	13	11	7	0	8	1	3	14	9	5	6	16	2	10	15
27	AVG		1	15	11	4	3	7	5	2	14	8	6	9	12	0	10	16
28	AVG		4	13	6	5	0	7	11	2	15	3	8	10	14	1	9	16
29	AVG		8	15	10	7	1	2	3	5	12	4	6	11	13	0	9	16
30	AVG		7	14	11	1	3	5	6	2	13	4	9	10	12	0	8	16
31	AVG		3	15	9	7	0	5	6	2	14	4	8	10	13	1	12	16
32	AVG		7	15	9	4	0	11	5	1	14	8	3	6	13	2	10	16
33	AVG		3	15	11	5	0	8	7	1	13	4	6	12	14	2	9	16
34	AVG		2	15	10	5	0	8	4	3	13	6	7	12	14	1	9	16
35	AVG		5	14	10	7	0	9	3	2	12	4	6	11	15	1	8	16
36	AVG		3	16	11	9	0	7	4	2	12	5	6	8	13	1	10	15
37	AVG		5	14	10	3	1	4	9	2	11	6	7	12	13	0	8	16

38	AVG		3	15	4	7	0	10	2	5	13	12	8	6	14	1	9	16
39	AVG		6	15	7	4	2	10	5	1	13	3	9	11	14	0	8	16
40	AVG		10	14	11	7	0	8	3	6	12	4	2	9	15	1	5	16
41	AVG		8	15	7	11	0	10	6	2	14	5	3	9	16	1	4	12
42	AVG		3	16	10	2	1	6	4	8	14	7	5	11	12	0	9	15
43	AVG		7	15	11	8	0	10	3	2	13	6	5	4	12	1	9	16
44	AVG		4	13	10	5	0	7	6	2	15	3	8	9	14	1	11	16
45	AVG		7	16	8	4	0	5	3	2	13	9	6	12	15	1	10	14
46	AVG		3	16	10	6	1	9	4	2	11	5	8	7	14	0	12	15
47	AVG		4	14	11	8	0	6	2	3	13	7	5	9	16	1	10	15
48	AVG		4	16	9	8	0	6	7	2	15	5	10	3	14	1	11	13
49	AVG		3	15	5	9	0	10	4	1	12	7	6	11	13	2	8	16
50	AVG		8	15	10	3	0	4	5	2	14	6	7	9	12	1	11	16
51	AVG		3	14	10	4	0	6	8	1	12	5	9	11	15	2	7	16
52	AVG		9	12	7	5	0	6	3	1	15	4	8	10	13	2	11	16
53	AVG		2	15	8	10	1	5	11	3	13	4	6	7	14	0	9	16
54	AVG		2	15	11	6	0	5	10	4	9	3	7	12	13	1	8	16
55	AVG		5	16	10	6	0	7	3	4	12	2	8	11	14	1	9	15
56	AVG		3	12	8	7	0	6	2	4	15	9	5	13	14	1	10	16
57	AVG		2	15	10	8	0	7	4	5	14	6	3	11	13	1	9	16
58	AVG		7	15	10	8	0	5	2	3	14	6	4	11	13	1	9	16
59	AVG		3	15	10	7	0	8	4	2	13	6	5	12	14	1	9	16
60	AVG		5	15	11	6	0	9	4	1	12	8	3	7	14	2	10	16
61	AVG		4	15	10	7	0	6	3	2	13	5	8	12	11	1	9	16
62	AVG		9	15	6	2	0	7	4	3	13	5	8	10	16	1	11	14
63	AVG		2	15	10	5	0	6	7	4	13	8	3	11	12	1	9	16
64	AVG		8	14	9	4	0	11	3	1	15	5	7	6	13	2	10	16
65	AVG		3	13	11	7	1	9	2	5	15	6	4	10	14	0	8	16
66	AVG		5	14	9	3	0	6	7	4	10	2	11	12	13	1	8	16
67	AVG		1	14	10	6	0	7	3	5	12	2	8	11	13	4	9	16
68	AVG		8	16	11	4	0	5	3	2	13	6	7	12	14	1	9	15

69	AVG		7	15	10	4	0	3	6	2	14	5	8	11	13	1	9	16
70	AVG		2	14	10	4	3	8	5	0	15	6	7	9	12	1	11	16
71	AVG		9	13	10	3	0	4	11	6	15	7	2	8	12	1	5	16
72	AVG		7	13	10	9	0	6	1	4	12	5	3	11	14	2	8	16
73	AVG		6	14	11	3	0	9	7	2	13	4	8	5	12	1	10	16
74	AVG		6	16	10	3	0	9	4	2	14	7	11	5	13	1	8	15
75	AVG		7	13	11	4	0	5	2	8	14	3	6	10	15	1	9	16
76	AVG		3	15	8	10	0	7	6	1	13	5	4	9	16	2	11	14
77	AVG		4	15	12	7	0	5	6	2	13	3	10	8	14	1	9	16
78	AVG		3	13	12	2	0	6	7	5	14	4	9	8	16	1	10	15
79	AVG		2	16	10	9	0	4	8	7	13	5	3	11	12	1	6	15
80	AVG		4	15	10	6	2	7	5	1	14	3	9	8	16	0	11	13
81	AVG		3	12	10	7	1	5	6	2	13	4	11	9	16	0	8	15
82	AVG		3	14	9	10	0	7	2	4	13	11	6	5	15	1	8	16
83	AVG		1	14	11	5	2	7	6	0	12	9	4	8	15	3	10	16
84	AVG		10	12	11	5	3	2	7	1	13	4	9	6	14	0	8	16
85	AVG		3	15	10	9	0	4	7	2	11	5	8	13	14	1	6	16
86	AVG		3	14	7	9	0	10	5	2	13	4	6	11	16	1	8	15
87	AVG		6	14	10	11	0	5	8	2	13	3	7	9	16	1	4	15
88	AVG		5	15	12	3	1	6	4	2	13	9	7	8	14	0	10	16
89	AVG		3	14	12	5	0	8	6	4	15	2	7	11	9	1	10	16
90	AVG		3	16	12	4	0	10	6	1	11	5	8	9	14	2	7	15
91	AVG		2	16	11	4	1	3	5	12	15	6	9	7	14	0	8	13
92	AVG		5	15	11	6	0	9	4	2	14	8	7	3	12	1	10	16
93	AVG		4	13	10	2	6	3	7	0	12	5	8	9	14	1	11	16
94	AVG		8	12	10	2	0	5	3	6	14	7	4	9	15	1	11	16
95	AVG		4	15	11	7	2	5	8	1	12	3	9	13	14	0	6	16
96	AVG		9	14	11	3	0	6	2	4	12	8	5	7	15	1	10	16
97	AVG		3	12	7	4	0	9	5	1	14	6	8	11	13	2	10	16
98	AVG		9	13	8	3	0	10	5	1	15	4	6	11	16	2	7	14
99	AVG		7	12	4	5	0	11	6	2	15	8	3	9	13	1	10	16

f. **Supplementary Table S2c**: : Feature group ranking results: top ranking feature groups.
"Selected features" shows the features in the group, "Feature selection method" shows the ranking method from which the feature group was derived. "AUC score" represents the assessment metric for group ranking, and is equal to the average AUC of the top performing 10% of the models (search grid presented in point 3.a) trained on the group.
E.g. the top row in the "One-month VT recurrence" table represents the feature group with the highest AUC score, derived from the top 6 features of the feature ranking determined using permutation importance and a multilayer perceptron model.

i. One-month VT recurrence endpoint

**Selected features**	**Feature selection method**	**Number of features**	**AUC score**
EF, TAPSE, ICD shock, HD instability, Incessant VT, Clinical VT eliminated	MLP, PI	6	0.707
EF, TAPSE, ICD shock, HD instability, Clinical VT eliminated	MLP, PI	5	0.704
EF, TAPSE, ICD shock, HD instability, Incessant VT, Electrical storm, Clinical VT eliminated	MLP, PI	7	0.702
EF, LVESD, TAPSE, HD instability	RF, PI	4	0.698
EF, LVESD, TAPSE, HD instability	SUM	4	0.698
TAPSE, ICD shock, HD instability, Clinical VT eliminated	MLP, PI	4	0.691
Age, EF, LVESD, TAPSE, HD instability, Clinical VT eliminated	RF, PI	6	0.682
Age, EF, LVESD, TAPSE, HD instability, Clinical VT eliminated	SUM	6	0.682
Age, EF, LVESD, TAPSE, HD instability	SUM	5	0.680
Age, EF, LVESD, TAPSE, HD instability	RF, PI	5	0.680
Age, EF, LVESD, TAPSE, HD instability, Clinical VT cycle length, Clinical VT eliminated	SUM	7	0.670
Age, EF, LVESD, TAPSE, HD instability, Clinical VT cycle length, Clinical VT eliminated	RF, PI	7	0.670
Age, EF, LVESD, HD instability, Clinical VT eliminated	XGB, RFE	5	0.661
Age, EF, HD instability, Clinical VT eliminated	XGB, RFE	4	0.659

Age, EF, LVESD, TAPSE, E wave deceleration time (DT), HD instability, Clinical VT cycle length	XGB, PI	7	0.649
Age, EF, LVESD, E wave deceleration time (DT), HD instability, Clinical VT eliminated	XGB, RFE	6	0.648
Age, EF, LVESD, E wave deceleration time (DT), HD instability, Clinical VT cycle length, Clinical VT eliminated	XGB, RFE	7	0.633
Age, EF, TAPSE, E wave deceleration time (DT), Clinical VT cycle length	XGB, PI	5	0.598
Age, EF, LVESD, TAPSE, E wave deceleration time (DT), Clinical VT cycle length	XGB, PI	6	0.597
Age, EF, E wave deceleration time (DT), Clinical VT cycle length	XGB, PI	4	0.568

ii. 1-year VT recurrence endpoint

**Selected features**	**Feature selection method**	**Number of features**	**AUC score**
LVESD, MR, ICD shock, HD instability, Inducible VT morphologies	RF, PI	5	0.677
LVESD, MR, ICD shock, HD instability, Inducible VT morphologies	MLP, PI	5	0.677
LVESD, E wave deceleration time (DT), MR, ICD shock, HD instability, Incessant_VT, Inducible VT morphologies	MLP, PI	7	0.675
Age, LVESD, MR, ICD shock, HD instability, Inducible VT morphologies, Clinical VT cycle length	RF, PI	7	0.675
LVESD, MR, ICD shock, HD instability, Incessant_VT, Inducible VT morphologies	MLP, PI	6	0.675
Age, LVESD, MR, ICD shock, HD instability, Inducible VT morphologies	RF, PI	6	0.669
Age, EF, LVESD, E wave deceleration time (DT), MR, ICD shock, Inducible VT morphologies	SUM	7	0.664

Age, EF, LVESD, E wave deceleration time (DT), MR, ICD shock, Inducible VT morphologies	XGB, RFE	7	0.664
LVESD, E wave deceleration time (DT), MR, Inducible VT morphologies	SUM	4	0.662
LVESD, MR, ICD shock, Inducible VT morphologies	RF, PI	4	0.659
Age, LVESD, E wave deceleration time (DT), MR, ICD shock, Inducible VT morphologies	SUM	6	0.655
MR, ICD shock, HD instability, Inducible VT morphologies	MLP, PI	4	0.655
Age, EF, LVESD, E wave deceleration time (DT), MR, Inducible VT morphologies	XGB, RFE	6	0.644
Age, EF, LVESD, MR, Inducible VT morphologies	XGB, RFE	5	0.639
Age, LVESD, E wave deceleration time (DT), MR, Inducible VT morphologies	SUM	5	0.635
Age, EF, LVESD, Inducible VT morphologies	XGB, RFE	4	0.631
Age, EF, LVESD, TAPSE, E wave deceleration time (DT), MR, Clinical VT cycle length	XGB, PI	7	0.622
Age, EF, LVESD, TAPSE, E wave deceleration time (DT)	XGB, PI	5	0.617
Age, EF, LVESD, TAPSE, E wave deceleration time (DT), Clinical VT cycle length	XGB, PI	6	0.615
Age, EF, LVESD, E wave deceleration time (DT)	XGB, PI	4	0.612

g. **Supplementary Table S2d**: Final model selection results. Top 30 models of each grid search (each combination of model type, pre-processing and oversampling), as ranked by AUC, are presented for each endpoint. Rows are ordered so that the table starts with the model with the highest mean AUC on the test populations. "Model" shows the type of model, "Preprocessing" shows the prepro-cessing method used, "Parameters" shows the hyperparameters of the model during the iteration. "Mean AUC (test)" represents the average of AUCs as assessed in 10 cross-validations. "Mean AUC (train)" shows the same for the train populations.

i. 1-month VT recurrence

**Model**	**Preprocessing**	**Oversampling**	**Parameters**	**Mean AUC (test)**	**Mean AUC (train)**
RF	Not used	Not used	RF__class_weight : None; RF__criterion : log_loss; RF__max_depth : 1; RF__max_features : 1; RF__min_samples_leaf : 2; RF__n_estimators : 300; RF__random_state : 0	0.730	0.758
NN	SS	SMOTE	NN__activation : logistic; NN__alpha : 0.002245921354228774; NN__hidden_layer_sizes : 3; NN__max_iter : 161; NN__random_state : 0; NN__solver : adam	0.729	0.727
NN	SS	SMOTE	NN__activation : logistic; NN__alpha : 0.002853339802465133; NN__hidden_layer_sizes : 3; NN__max_iter : 158; NN__random_state : 0; NN__solver : adam	0.729	0.727
NN	SS	SMOTE	NN__activation : logistic; NN__alpha : 0.0001; NN__hidden_layer_sizes : 3; NN__max_iter : 148; NN__random_state : 0; NN__solver : adam	0.728	0.726
RF	SS	SMOTE	RF__class_weight : None; RF__criterion : log_loss; RF__max_depth : 1; RF__max_features : 1;	0.728	0.738

			RF__min_samples_leaf : 20; RF__n_estimators : 419; RF__random_state : 0		
RF	Not used	Not used	RF__class_weight : None; RF__criterion : log_loss; RF__max_depth : 1; RF__max_features : 1; RF__min_samples_leaf : 1; RF__n_estimators : 392; RF__random_state : 0	0.727	0.756
NN	SS	SMOTE	NN__activation : logistic; NN__alpha : 0.0001; NN__hidden_layer_sizes : 3; NN__max_iter : 174; NN__random_state : 0; NN__solver : adam	0.727	0.729
RF	SS	SMOTE	RF__class_weight : None; RF__criterion : log_loss; RF__max_depth : 1; RF__max_features : 1; RF__min_samples_leaf : 20; RF__n_estimators : 300; RF__random_state : 0	0.727	0.737
RF	SS	SMOTE	RF__class_weight : balanced; RF__criterion : log_loss; RF__max_depth : 1; RF__max_features : 1; RF__min_samples_leaf : 20; RF__n_estimators : 421; RF__random_state : 0	0.726	0.738
RF	SS	SMOTE	RF__class_weight : None; RF__criterion : log_loss; RF__max_depth : 1; RF__max_features : 1; RF__min_samples_leaf : 20; RF__n_estimators : 447; RF__random_state : 0	0.726	0.739
NN	SS	ROS	NN__activation : logistic; NN__alpha : 10.0; NN__hidden_layer_sizes : 3; NN__max_iter : 406; NN__random_state : 0; NN__solver : adam	0.726	0.728
RF	SS	SMOTE	RF__class_weight : balanced; RF__criterion : log_loss; RF__max_depth : 1; RF__max_features : 1; RF__min_samples_leaf : 20; RF__n_estimators : 323; RF__random_state : 0	0.725	0.738
RF	SS	SMOTE	RF__class_weight : balanced_subsample; RF__criterion : log_loss; RF__max_depth : 1;	0.725	0.737

			RF__max_features : 1; RF__min_samples_leaf : 20; RF__n_estimators : 364; RF__random_state : 0		
RF	SS	SMOTE	RF__class_weight : None; RF__criterion : log_loss; RF__max_depth : 1; RF__max_features : 1; RF__min_samples_leaf : 6; RF__n_estimators : 300; RF__random_state : 0	0.725	0.738
RF	SS	SMOTE	RF__class_weight : balanced; RF__criterion : log_loss; RF__max_depth : 1; RF__max_features : 1; RF__min_samples_leaf : 6; RF__n_estimators : 431; RF__random_state : 0	0.724	0.739
NN	SS	SMOTE	NN__activation : logistic; NN__alpha : 0.005073082593486951; NN__hidden_layer_sizes : 3; NN__max_iter : 201; NN__random_state : 0; NN__solver : adam	0.724	0.731
RF	Not used	Not used	RF__class_weight : balanced_subsample; RF__criterion : log_loss; RF__max_depth : 1; RF__max_features : 1; RF__min_samples_leaf : 2; RF__n_estimators : 655; RF__random_state : 43	0.723	0.754
RF	Not used	Not used	RF__class_weight : None; RF__criterion : log_loss; RF__max_depth : 1; RF__max_features : 1; RF__min_samples_leaf : 1; RF__n_estimators : 300; RF__random_state : 30	0.722	0.758
RF	Not used	Not used	RF__class_weight : None; RF__criterion : log_loss; RF__max_depth : 1; RF__max_features : 1; RF__min_samples_leaf : 7; RF__n_estimators : 1000; RF__random_state : 0	0.721	0.757
RF	Not used	Not used	RF__class_weight : None; RF__criterion : log_loss; RF__max_depth : 1; RF__max_features : 1; RF__min_samples_leaf : 1; RF__n_estimators : 1000; RF__random_state : 0	0.721	0.758

RF	Not used	Not used	RF__class_weight : balanced; RF__criterion : log_loss; RF__max_depth : 1; RF__max_features : 1; RF__min_samples_leaf : 8; RF__n_estimators : 564; RF__random_state : 0	0.720	0.754
NN	MM	ROS	NN__activation : identity; NN__alpha : 0.001254961325650091; NN__hidden_layer_sizes : 6; NN__max_iter : 500; NN__random_state : 0; NN__solver : adam	0.720	0.731
RF	Not used	Not used	RF__class_weight : balanced; RF__criterion : log_loss; RF__max_depth : 1; RF__max_features : 1; RF__min_samples_leaf : 1; RF__n_estimators : 300; RF__random_state : 35	0.720	0.756
RF	Not used	Not used	RF__class_weight : balanced; RF__criterion : log_loss; RF__max_depth : 1; RF__max_features : 1; RF__min_samples_leaf : 7; RF__n_estimators : 300; RF__random_state : 0	0.719	0.754
RF	Not used	Not used	RF__class_weight : None; RF__criterion : log_loss; RF__max_depth : 1; RF__max_features : 1; RF__min_samples_leaf : 12; RF__n_estimators : 300; RF__random_state : 0	0.719	0.749
RF	SS	ADASYN	RF__class_weight : balanced; RF__criterion : log_loss; RF__max_depth : 1; RF__max_features : 1; RF__min_samples_leaf : 2; RF__n_estimators : 1000; RF__random_state : 0	0.719	0.736
RF	SS	ADASYN	RF__class_weight : None; RF__criterion : log_loss; RF__max_depth : 1; RF__max_features : 1; RF__min_samples_leaf : 3; RF__n_estimators : 1000; RF__random_state : 0	0.719	0.736
RF	Not used	Not used	RF__class_weight : None; RF__criterion : log_loss; RF__max_depth : 1; RF__max_features : 1;	0.718	0.759

			RF__min_samples_leaf : 4; RF__n_estimators : 1000; RF__random_state : 0		
NN	SS	ROS	NN__activation : identity; NN__alpha : 10.0; NN__hidden_layer_sizes : 3; NN__max_iter : 207; NN__random_state : 0; NN__solver : adam	0.718	0.732
NN	SS	ROS	NN__activation : identity; NN__alpha : 10.0; NN__hidden_layer_sizes : 3; NN__max_iter : 197; NN__random_state : 0; NN__solver : adam	0.718	0.732
RF	SS	ADASYN	RF__class_weight : balanced; RF__criterion : log_loss; RF__max_depth : 1; RF__max_features : 1; RF__min_samples_leaf : 1; RF__n_estimators : 300; RF__random_state : 0	0.718	0.735
RF	MM	ROS	RF__class_weight : None; RF__criterion : log_loss; RF__max_depth : 1; RF__max_features : 1; RF__min_samples_leaf : 20; RF__n_estimators : 548; RF__random_state : 0	0.718	0.752
NN	SS	ROS	NN__activation : identity; NN__alpha : 10.0; NN__hidden_layer_sizes : 3; NN__max_iter : 206; NN__random_state : 0; NN__solver : adam	0.718	0.732
NN	SS	ROS	NN__activation : identity; NN__alpha : 10.0; NN__hidden_layer_sizes : 3; NN__max_iter : 205; NN__random_state : 0; NN__solver : adam	0.717	0.732
NN	SS	ROS	NN__activation : identity; NN__alpha : 10.0; NN__hidden_layer_sizes : 3; NN__max_iter : 189; NN__random_state : 0; NN__solver : adam	0.717	0.731
NN	SS	SMOTE	NN__activation : logistic; NN__alpha : 0.0001; NN__hidden_layer_sizes : 3; NN__max_iter : 245; NN__random_state : 0; NN__solver : adam	0.717	0.733
RF	Not used	Not used	RF__class_weight : balanced; RF__criterion : log_loss; RF__max_depth : 1; RF__max_features : 1;	0.717	0.756

			RF__min_samples_leaf : 6; RF__n_estimators : 1000; RF__random_state : 0		
NN	MM	ROS	NN__activation : logistic; NN__alpha : 0.006324534487428441; NN__hidden_layer_sizes : 6; NN__max_iter : 410; NN__random_state : 0; NN__solver : adam	0.717	0.726
RF	MM	ROS	RF__class_weight : balanced; RF__criterion : log_loss; RF__max_depth : 1; RF__max_features : 1; RF__min_samples_leaf : 20; RF__n_estimators : 1000; RF__random_state : 0	0.716	0.753
RF	SS	ROS	RF__class_weight : None; RF__criterion : log_loss; RF__max_depth : 1; RF__max_features : 1; RF__min_samples_leaf : 20; RF__n_estimators : 1000; RF__random_state : 0	0.716	0.753
NN	SS	ADASYN	NN__activation : logistic; NN__alpha : 0.0001; NN__hidden_layer_sizes : 50; NN__max_iter : 50; NN__random_state : 0; NN__solver : adam	0.716	0.732
NN	MM	ROS	NN__activation : logistic; NN__alpha : 0.001413672972795645; NN__hidden_layer_sizes : 4; NN__max_iter : 500; NN__random_state : 0; NN__solver : adam	0.716	0.725
NN	SS	SMOTE	NN__activation : logistic; NN__alpha : 10.0; NN__hidden_layer_sizes : 3; NN__max_iter : 182; NN__random_state : 0; NN__solver : adam	0.716	0.726
RF	MM	ROS	RF__class_weight : balanced_subsample; RF__criterion : log_loss; RF__max_depth : 1; RF__max_features : 1; RF__min_samples_leaf : 20; RF__n_estimators : 674; RF__random_state : 50	0.716	0.753
RF	SS	ADASYN	RF__class_weight : balanced; RF__criterion : log_loss; RF__max_depth : 1; RF__max_features : 1;	0.715	0.740

			RF__min_samples_leaf : 1; RF__n_estimators : 505; RF__random_state : 19		
RF	SS	SMOTE	RF__class_weight : None; RF__criterion : log_loss; RF__max_depth : 1; RF__max_features : 1; RF__min_samples_leaf : 20; RF__n_estimators : 1000; RF__random_state : 0	0.715	0.739
NN	MM	ROS	NN__activation : logistic; NN__alpha : 0.0001; NN__hidden_layer_sizes : 10; NN__max_iter : 500; NN__random_state : 0; NN__solver : adam	0.715	0.726
NN	MM	ROS	NN__activation : identity; NN__alpha : 0.00014302786666323548; NN__hidden_layer_sizes : 35; NN__max_iter : 50; NN__random_state : 0; NN__solver : adam	0.715	0.728
RF	MM	ROS	RF__class_weight : None; RF__criterion : log_loss; RF__max_depth : 1; RF__max_features : 1; RF__min_samples_leaf : 20; RF__n_estimators : 300; RF__random_state : 0	0.715	0.754
RF	SS	ROS	RF__class_weight : None; RF__criterion : log_loss; RF__max_depth : 1; RF__max_features : 1; RF__min_samples_leaf : 20; RF__n_estimators : 300; RF__random_state : 0	0.715	0.754
NN	SS	ROS	NN__activation : logistic; NN__alpha : 0.0009395218022160033; NN__hidden_layer_sizes : 3; NN__max_iter : 462; NN__random_state : 0; NN__solver : adam	0.714	0.736
NN	MM	ROS	NN__activation : tanh; NN__alpha : 0.004469278831114033; NN__hidden_layer_sizes : 8; NN__max_iter : 445; NN__random_state : 0; NN__solver : adam	0.714	0.732
RF	Not used	Not used	RF__class_weight : balanced_subsample; RF__criterion : log_loss; RF__max_depth : 1;	0.714	0.757

			RF__max_features : 1; RF__min_samples_leaf : 6; RF__n_estimators : 889; RF__random_state : 99		
RF	SS	SMOTE	RF__class_weight : None; RF__criterion : log_loss; RF__max_depth : 1; RF__max_features : 1; RF__min_samples_leaf : 1; RF__n_estimators : 1000; RF__random_state : 83	0.714	0.741
NN	MM	ROS	NN__activation : logistic; NN__alpha : 0.0001000006070296437; NN__hidden_layer_sizes : 40; NN__max_iter : 68; NN__random_state : 0; NN__solver : adam	0.714	0.722
RF	MM	SMOTE	RF__class_weight : None; RF__criterion : log_loss; RF__max_depth : 1; RF__max_features : 1; RF__min_samples_leaf : 20; RF__n_estimators : 357; RF__random_state : 0	0.714	0.739
RF	Not used	Not used	RF__class_weight : None; RF__criterion : gini; RF__max_depth : 1; RF__max_features : 1; RF__min_samples_leaf : 8; RF__n_estimators : 1000; RF__random_state : 0	0.714	0.755
NN	SS	ROS	NN__activation : relu; NN__alpha : 10.0; NN__hidden_layer_sizes : 3; NN__max_iter : 202; NN__random_state : 0; NN__solver : adam	0.713	0.729
RF	SS	SMOTE	RF__class_weight : balanced; RF__criterion : log_loss; RF__max_depth : 1; RF__max_features : 1; RF__min_samples_leaf : 1; RF__n_estimators : 1000; RF__random_state : 0	0.713	0.739
RF	MM	SMOTE	RF__class_weight : balanced; RF__criterion : log_loss; RF__max_depth : 1; RF__max_features : 1; RF__min_samples_leaf : 1; RF__n_estimators : 350; RF__random_state : 0	0.713	0.739
RF	MM	ROS	RF__class_weight : None; RF__criterion : log_loss; RF__max_depth : 1; RF__max_features : 1;	0.713	0.751

			RF__min_samples_leaf : 1; RF__n_estimators : 594; RF__random_state : 0		
RF	MM	ROS	RF__class_weight : balanced; RF__criterion : log_loss; RF__max_depth : 1; RF__max_features : 1; RF__min_samples_leaf : 1; RF__n_estimators : 1000; RF__random_state : 0	0.713	0.751
RF	MM	SMOTE	RF__class_weight : None; RF__criterion : log_loss; RF__max_depth : 1; RF__max_features : 1; RF__min_samples_leaf : 20; RF__n_estimators : 359; RF__random_state : 0	0.712	0.738
NN	Not used	Not used	NN__activation : tanh; NN__alpha : 0.27998320381921143; NN__hidden_layer_sizes : 9; NN__max_iter : 400; NN__random_state : 0; NN__solver : adam	0.712	0.755
RF	MM	SMOTE	RF__class_weight : balanced; RF__criterion : log_loss; RF__max_depth : 1; RF__max_features : 1; RF__min_samples_leaf : 1; RF__n_estimators : 354; RF__random_state : 0	0.712	0.739
RF	MM	SMOTE	RF__class_weight : balanced; RF__criterion : log_loss; RF__max_depth : 1; RF__max_features : 1; RF__min_samples_leaf : 1; RF__n_estimators : 348; RF__random_state : 0	0.712	0.739
RF	SS	ROS	RF__class_weight : None; RF__criterion : log_loss; RF__max_depth : 1; RF__max_features : 1; RF__min_samples_leaf : 5; RF__n_estimators : 1000; RF__random_state : 0	0.712	0.751
NN	SS	ROS	NN__activation : logistic; NN__alpha : 0.46415318133211225; NN__hidden_layer_sizes : 3; NN__max_iter : 500; NN__random_state : 0; NN__solver : adam	0.712	0.735

RF	Not used	Not used	RF__class_weight : None; RF__criterion : gini; RF__max_depth : 1; RF__max_features : 1; RF__min_samples_leaf : 20; RF__n_estimators : 300; RF__random_state : 0	0.712	0.747
RF	MM	SMOTE	RF__class_weight : None; RF__criterion : log_loss; RF__max_depth : 1; RF__max_features : 1; RF__min_samples_leaf : 20; RF__n_estimators : 363; RF__random_state : 0	0.712	0.738
RF	MM	SMOTE	RF__class_weight : balanced; RF__criterion : log_loss; RF__max_depth : 1; RF__max_features : 1; RF__min_samples_leaf : 1; RF__n_estimators : 355; RF__random_state : 0	0.712	0.739
RF	Not used	Not used	RF__class_weight : balanced; RF__criterion : gini; RF__max_depth : 1; RF__max_features : 1; RF__min_samples_leaf : 9; RF__n_estimators : 387; RF__random_state : 0	0.712	0.755
NN	MM	ROS	NN__activation : logistic; NN__alpha : 0.00018986178592672774; NN__hidden_layer_sizes : 40; NN__max_iter : 192; NN__random_state : 0; NN__solver : adam	0.712	0.726
RF	SS	ADASYN	RF__class_weight : balanced; RF__criterion : log_loss; RF__max_depth : 1; RF__max_features : 1; RF__min_samples_leaf : 20; RF__n_estimators : 1000; RF__random_state : 0	0.712	0.735
RF	MM	SMOTE	RF__class_weight : balanced; RF__criterion : log_loss; RF__max_depth : 1; RF__max_features : 1; RF__min_samples_leaf : 1; RF__n_estimators : 344; RF__random_state : 0	0.711	0.739
RF	MM	ROS	RF__class_weight : balanced; RF__criterion : log_loss; RF__max_depth : 1; RF__max_features : 1;	0.711	0.753

			RF__min_samples_leaf : 1; RF__n_estimators : 1000; RF__random_state : 63		
RF	MM	SMOTE	RF__class_weight : balanced; RF__criterion : log_loss; RF__max_depth : 1; RF__max_features : 1; RF__min_samples_leaf : 20; RF__n_estimators : 358; RF__random_state : 0	0.711	0.739
RF	MM	SMOTE	RF__class_weight : None; RF__criterion : log_loss; RF__max_depth : 1; RF__max_features : 1; RF__min_samples_leaf : 20; RF__n_estimators : 365; RF__random_state : 0	0.711	0.738
RF	SS	SMOTE	RF__class_weight : None; RF__criterion : gini; RF__max_depth : 1; RF__max_features : 1; RF__min_samples_leaf : 20; RF__n_estimators : 300; RF__random_state : 0	0.711	0.735
RF	MM	SMOTE	RF__class_weight : balanced; RF__criterion : log_loss; RF__max_depth : 1; RF__max_features : 1; RF__min_samples_leaf : 1; RF__n_estimators : 360; RF__random_state : 0	0.711	0.739
NN	MM	ADASYN	NN__activation : logistic; NN__alpha : 0.029741017653186517; NN__hidden_layer_sizes : 50; NN__max_iter : 50; NN__random_state : 0; NN__solver : adam	0.711	0.720
NN	MM	SMOTE	NN__activation : identity; NN__alpha : 0.5732787316498061; NN__hidden_layer_sizes : 30; NN__max_iter : 50; NN__random_state : 0; NN__solver : adam	0.711	0.725
NN	Not used	Not used	NN__activation : identity; NN__alpha : 0.00012096723075925161; NN__hidden_layer_sizes : 50; NN__max_iter : 51; NN__random_state : 0; NN__solver : lbfgs	0.711	0.735

RF	MM	ROS	RF__class_weight : balanced_subsample; RF__criterion : log_loss; RF__max_depth : 1; RF__max_features : 1; RF__min_samples_leaf : 10; RF__n_estimators : 1000; RF__random_state : 30	0.711	0.755
RF	MM	SMOTE	RF__class_weight : None; RF__criterion : log_loss; RF__max_depth : 1; RF__max_features : 1; RF__min_samples_leaf : 20; RF__n_estimators : 300; RF__random_state : 0	0.711	0.738
RF	MM	SMOTE	RF__class_weight : None; RF__criterion : log_loss; RF__max_depth : 1; RF__max_features : 1; RF__min_samples_leaf : 20; RF__n_estimators : 368; RF__random_state : 0	0.711	0.738
RF	MM	SMOTE	RF__class_weight : balanced; RF__criterion : log_loss; RF__max_depth : 1; RF__max_features : 1; RF__min_samples_leaf : 1; RF__n_estimators : 356; RF__random_state : 0	0.711	0.739
NN	MM	ROS	NN__activation : tanh; NN__alpha : 0.005403123829374884; NN__hidden_layer_sizes : 8; NN__max_iter : 202; NN__random_state : 0; NN__solver : adam	0.711	0.723
NN	Not used	Not used	NN__activation : tanh; NN__alpha : 0.0001; NN__hidden_layer_sizes : 30; NN__max_iter : 291; NN__random_state : 0; NN__solver : adam	0.711	0.758
RF	Not used	Not used	RF__class_weight : balanced_subsample; RF__criterion : log_loss; RF__max_depth : 1; RF__max_features : 1; RF__min_samples_leaf : 4; RF__n_estimators : 300; RF__random_state : 1	0.711	0.755
RF	MM	ROS	RF__class_weight : balanced; RF__criterion : log_loss; RF__max_depth : 1; RF__max_features : 1; RF__min_samples_leaf : 1; RF__n_estimators : 300; RF__random_state : 0	0.710	0.752

RF	MM	ROS	RF__class_weight : balanced_subsample; RF__criterion : log_loss; RF__max_depth : 1; RF__max_features : 1; RF__min_samples_leaf : 2; RF__n_estimators : 1000; RF__random_state : 32	0.710	0.754
NN	Not used	Not used	NN__activation : identity; NN__alpha : 0.02322431597682465; NN__hidden_layer_sizes : 9; NN__max_iter : 148; NN__random_state : 0; NN__solver : lbfgs	0.710	0.735
NN	MM	ROS	NN__activation : tanh; NN__alpha : 0.0001; NN__hidden_layer_sizes : 30; NN__max_iter : 50; NN__random_state : 0; NN__solver : adam	0.710	0.722
NN	SS	ROS	NN__activation : identity; NN__alpha : 10.0; NN__hidden_layer_sizes : 15; NN__max_iter : 202; NN__random_state : 0; NN__solver : adam	0.710	0.733
NN	Not used	Not used	NN__activation : tanh; NN__alpha : 0.0001; NN__hidden_layer_sizes : 30; NN__max_iter : 500; NN__random_state : 0; NN__solver : adam	0.710	0.769
RF	MM	ROS	RF__class_weight : balanced; RF__criterion : log_loss; RF__max_depth : 1; RF__max_features : 1; RF__min_samples_leaf : 8; RF__n_estimators : 601; RF__random_state : 59	0.710	0.754
NN	MM	ROS	NN__activation : logistic; NN__alpha : 0.007090111341938081; NN__hidden_layer_sizes : 15; NN__max_iter : 500; NN__random_state : 0; NN__solver : adam	0.710	0.723
NN	Not used	Not used	NN__activation : identity; NN__alpha : 0.0001; NN__hidden_layer_sizes : 24; NN__max_iter : 68; NN__random_state : 0; NN__solver : lbfgs	0.710	0.735
RF	Not used	Not used	RF__class_weight : None; RF__criterion : log_loss; RF__max_depth : 2; RF__max_features : 1;	0.710	0.774

			RF__min_samples_leaf : 2; RF__n_estimators : 573; RF__random_state : 3		
RF	MM	ROS	RF__class_weight : balanced; RF__criterion : log_loss; RF__max_depth : 2; RF__max_features : 1; RF__min_samples_leaf : 3; RF__n_estimators : 300; RF__random_state : 0	0.710	0.769
NN	MM	ROS	NN__activation : logistic; NN__alpha : 0.0001; NN__hidden_layer_sizes : 30; NN__max_iter : 500; NN__random_state : 0; NN__solver : adam	0.710	0.728
RF	SS	SMOTE	RF__class_weight : balanced_subsample; RF__criterion : log_loss; RF__max_depth : 1; RF__max_features : 1; RF__min_samples_leaf : 20; RF__n_estimators : 953; RF__random_state : 65	0.710	0.738
NN	SS	ROS	NN__activation : identity; NN__alpha : 2.248094973114098; NN__hidden_layer_sizes : 3; NN__max_iter : 196; NN__random_state : 0; NN__solver : adam	0.710	0.732
RF	MM	SMOTE	RF__class_weight : balanced; RF__criterion : log_loss; RF__max_depth : 1; RF__max_features : 1; RF__min_samples_leaf : 1; RF__n_estimators : 300; RF__random_state : 0	0.710	0.739
NN	SS	SMOTE	NN__activation : identity; NN__alpha : 0.0011726495223515266; NN__hidden_layer_sizes : 3; NN__max_iter : 141; NN__random_state : 0; NN__solver : adam	0.710	0.724
RF	SS	ROS	RF__class_weight : balanced; RF__criterion : log_loss; RF__max_depth : 1; RF__max_features : 1; RF__min_samples_leaf : 1; RF__n_estimators : 612; RF__random_state : 2	0.710	0.755

NN	SS	ROS	NN__activation : identity; NN__alpha : 10.0; NN__hidden_layer_sizes : 3; NN__max_iter : 279; NN__random_state : 0; NN__solver : adam	0.710	0.733
NN	Not used	Not used	NN__activation : identity; NN__alpha : 0.0001; NN__hidden_layer_sizes : 40; NN__max_iter : 270; NN__random_state : 0; NN__solver : lbfgs	0.710	0.735
NN	Not used	Not used	NN__activation : identity; NN__alpha : 0.04103700584134806; NN__hidden_layer_sizes : 4; NN__max_iter : 500; NN__random_state : 0; NN__solver : lbfgs	0.710	0.735
NN	SS	SMOTE	NN__activation : identity; NN__alpha : 10.0; NN__hidden_layer_sizes : 3; NN__max_iter : 232; NN__random_state : 0; NN__solver : adam	0.710	0.732
RF	MM	ROS	RF__class_weight : balanced; RF__criterion : log_loss; RF__max_depth : 2; RF__max_features : 1; RF__min_samples_leaf : 1; RF__n_estimators : 888; RF__random_state : 43	0.710	0.770
RF	SS	SMOTE	RF__class_weight : balanced_subsample; RF__criterion : log_loss; RF__max_depth : 1; RF__max_features : 1; RF__min_samples_leaf : 20; RF__n_estimators : 1000; RF__random_state : 75	0.710	0.739
RF	MM	SMOTE	RF__class_weight : balanced; RF__criterion : log_loss; RF__max_depth : 1; RF__max_features : 1; RF__min_samples_leaf : 20; RF__n_estimators : 364; RF__random_state : 0	0.709	0.738
NN	Not used	Not used	NN__activation : tanh; NN__alpha : 0.25314925275071964; NN__hidden_layer_sizes : 9; NN__max_iter : 374; NN__random_state : 0; NN__solver : adam	0.709	0.754
NN	MM	SMOTE	NN__activation : identity; NN__alpha : 0.2698616513598057; NN__hidden_layer_sizes : 30;	0.709	0.725

			NN__max_iter : 50; NN__random_state : 0; NN__solver : adam		
NN	Not used	Not used	NN__activation : identity; NN__alpha : 0.0005010365707035275; NN__hidden_layer_sizes : 35; NN__max_iter : 124; NN__random_state : 0; NN__solver : lbfgs	0.709	0.735
NN	Not used	Not used	NN__activation : identity; NN__alpha : 0.0003253151456288895; NN__hidden_layer_sizes : 10; NN__max_iter : 53; NN__random_state : 0; NN__solver : lbfgs	0.709	0.734
NN	MM	ROS	NN__activation : logistic; NN__alpha : 0.43513970791520495; NN__hidden_layer_sizes : 40; NN__max_iter : 192; NN__random_state : 0; NN__solver : adam	0.709	0.723
NN	MM	ROS	NN__activation : identity; NN__alpha : 1.2278635248432284; NN__hidden_layer_sizes : 27; NN__max_iter : 165; NN__random_state : 0; NN__solver : adam	0.709	0.729
NN	SS	SMOTE	NN__activation : identity; NN__alpha : 0.00043653486872989914; NN__hidden_layer_sizes : 3; NN__max_iter : 140; NN__random_state : 0; NN__solver : adam	0.709	0.724
NN	Not used	Not used	NN__activation : identity; NN__alpha : 0.0001; NN__hidden_layer_sizes : 8; NN__max_iter : 500; NN__random_state : 0; NN__solver : lbfgs	0.709	0.735
NN	SS	ROS	NN__activation : identity; NN__alpha : 10.0; NN__hidden_layer_sizes : 3; NN__max_iter : 435; NN__random_state : 0; NN__solver : adam	0.709	0.733
NN	MM	SMOTE	NN__activation : logistic; NN__alpha : 0.7392993308744839; NN__hidden_layer_sizes : 50;	0.709	0.717

			NN__max_iter : 50; NN__random_state : 0; NN__solver : adam		
NN	MM	ROS	NN__activation : tanh; NN__alpha : 2.6096146808538574; NN__hidden_layer_sizes : 8; NN__max_iter : 478; NN__random_state : 0; NN__solver : adam	0.709	0.727
NN	Not used	Not used	NN__activation : identity; NN__alpha : 0.0001; NN__hidden_layer_sizes : 40; NN__max_iter : 50; NN__random_state : 0; NN__solver : lbfgs	0.709	0.735
NN	Not used	Not used	NN__activation : identity; NN__alpha : 0.00011286464023056555; NN__hidden_layer_sizes : 50; NN__max_iter : 500; NN__random_state : 0; NN__solver : lbfgs	0.709	0.735
RF	SS	ROS	RF__class_weight : balanced; RF__criterion : gini; RF__max_depth : 1; RF__max_features : 1; RF__min_samples_leaf : 20; RF__n_estimators : 300; RF__random_state : 0	0.708	0.754
NN	SS	ROS	NN__activation : relu; NN__alpha : 10.0; NN__hidden_layer_sizes : 3; NN__max_iter : 467; NN__random_state : 0; NN__solver : adam	0.708	0.732
NN	Not used	Not used	NN__activation : identity; NN__alpha : 0.01953721425874314; NN__hidden_layer_sizes : 4; NN__max_iter : 465; NN__random_state : 0; NN__solver : lbfgs	0.708	0.735
NN	MM	ROS	NN__activation : logistic; NN__alpha : 0.0746220651193357; NN__hidden_layer_sizes : 20; NN__max_iter : 183; NN__random_state : 0; NN__solver : adam	0.708	0.720
NN	MM	SMOTE	NN__activation : tanh; NN__alpha : 6.603270381036594; NN__hidden_layer_sizes : 3;	0.708	0.718

			NN__max_iter : 489; NN__random_state : 0; NN__solver : adam		
NN	Not used	Not used	NN__activation : tanh; NN__alpha : 0.014240291413468184; NN__hidden_layer_sizes : 9; NN__max_iter : 447; NN__random_state : 0; NN__solver : adam	0.708	0.756
XGB	Not used	Not used	XGB__alpha : 7.9949791346095544; XGB__colsample_bytree : 1.0; XGB__gamma : 0.1; XGB__max_depth : 3; XGB__min_child_weight : 0.0; XGB__random_state : 0; XGB__scale_pos_weight : 2.0; XGB__subsample : 0.5	0.708	0.816
NN	MM	ROS	NN__activation : identity; NN__alpha : 0.0001; NN__hidden_layer_sizes : 8; NN__max_iter : 500; NN__random_state : 0; NN__solver : adam	0.708	0.728
XGB	Not used	Not used	XGB__alpha : 7.216063149618247; XGB__colsample_bytree : 1.0; XGB__gamma : 0.1; XGB__max_depth : 3; XGB__min_child_weight : 0.0; XGB__random_state : 0; XGB__scale_pos_weight : 1.531918949951429; XGB__subsample : 0.5	0.708	0.802
NN	MM	SMOTE	NN__activation : identity; NN__alpha : 0.8460012669587524; NN__hidden_layer_sizes : 35; NN__max_iter : 50; NN__random_state : 0; NN__solver : adam	0.708	0.724
NN	Not used	Not used	NN__activation : tanh; NN__alpha : 4.1023173071789145; NN__hidden_layer_sizes : 3; NN__max_iter : 325; NN__random_state : 0; NN__solver : adam	0.708	0.731
NN	MM	ROS	NN__activation : identity; NN__alpha : 10.0; NN__hidden_layer_sizes : 8; NN__max_iter : 500; NN__random_state : 0; NN__solver : adam	0.708	0.716

NN	MM	ADASYN	NN__activation : identity; NN__alpha : 0.8903812307853785; NN__hidden_layer_sizes : 7; NN__max_iter : 500; NN__random_state : 0; NN__solver : adam	0.708	0.730
RF	MM	ROS	RF__class_weight : None; RF__criterion : log_loss; RF__max_depth : 1; RF__max_features : 1; RF__min_samples_leaf : 20; RF__n_estimators : 419; RF__random_state : 34	0.708	0.754
NN	SS	SMOTE	NN__activation : identity; NN__alpha : 0.0017196598039130406; NN__hidden_layer_sizes : 3; NN__max_iter : 139; NN__random_state : 0; NN__solver : adam	0.708	0.724
NN	MM	SMOTE	NN__activation : relu; NN__alpha : 10.0; NN__hidden_layer_sizes : 50; NN__max_iter : 302; NN__random_state : 0; NN__solver : adam	0.708	0.716
NN	SS	SMOTE	NN__activation : identity; NN__alpha : 0.0001; NN__hidden_layer_sizes : 3; NN__max_iter : 135; NN__random_state : 0; NN__solver : adam	0.708	0.723
NN	MM	SMOTE	NN__activation : tanh; NN__alpha : 10.0; NN__hidden_layer_sizes : 50; NN__max_iter : 124; NN__random_state : 0; NN__solver : adam	0.708	0.718
NN	Not used	Not used	NN__activation : identity; NN__alpha : 0.03792262732844632; NN__hidden_layer_sizes : 4; NN__max_iter : 443; NN__random_state : 0; NN__solver : lbfgs	0.708	0.735
NN	SS	ROS	NN__activation : identity; NN__alpha : 0.0001; NN__hidden_layer_sizes : 3; NN__max_iter : 437; NN__random_state : 0; NN__solver : adam	0.708	0.733
NN	SS	SMOTE	NN__activation : identity; NN__alpha : 10.0; NN__hidden_layer_sizes : 3; NN__max_iter : 242; NN__random_state : 0; NN__solver : adam	0.708	0.732

NN	MM	SMOTE	NN__activation : identity; NN__alpha : 4.446755998289332; NN__hidden_layer_sizes : 50; NN__max_iter : 50; NN__random_state : 0; NN__solver : adam	0.708	0.715
NN	SS	ROS	NN__activation : identity; NN__alpha : 10.0; NN__hidden_layer_sizes : 4; NN__max_iter : 500; NN__random_state : 0; NN__solver : adam	0.708	0.733
NN	MM	ADASYN	NN__activation : tanh; NN__alpha : 2.6096146808538574; NN__hidden_layer_sizes : 8; NN__max_iter : 478; NN__random_state : 0; NN__solver : adam	0.708	0.728
RF	Not used	Not used	RF__class_weight : balanced; RF__criterion : log_loss; RF__max_depth : 2; RF__max_features : 1; RF__min_samples_leaf : 10; RF__n_estimators : 300; RF__random_state : 0	0.707	0.766
NN	SS	SMOTE	NN__activation : identity; NN__alpha : 0.00022236231662955448; NN__hidden_layer_sizes : 3; NN__max_iter : 138; NN__random_state : 0; NN__solver : adam	0.707	0.723
NN	SS	ROS	NN__activation : identity; NN__alpha : 0.2915359171992179; NN__hidden_layer_sizes : 3; NN__max_iter : 203; NN__random_state : 0; NN__solver : adam	0.707	0.733
NN	SS	SMOTE	NN__activation : identity; NN__alpha : 0.0001; NN__hidden_layer_sizes : 3; NN__max_iter : 134; NN__random_state : 0; NN__solver : adam	0.707	0.723
NN	MM	ROS	NN__activation : tanh; NN__alpha : 0.0001; NN__hidden_layer_sizes : 50; NN__max_iter : 50; NN__random_state : 0; NN__solver : adam	0.707	0.722
NN	Not used	Not used	NN__activation : identity; NN__alpha : 0.4938436618748837; NN__hidden_layer_sizes : 3;	0.707	0.735

			NN__max_iter : 414; NN__random_state : 0; NN__solver : lbfgs		
NN	Not used	Not used	NN__activation : identity; NN__alpha : 0.32676223258968823; NN__hidden_layer_sizes : 3; NN__max_iter : 184; NN__random_state : 0; NN__solver : lbfgs	0.707	0.735
NN	SS	ROS	NN__activation : identity; NN__alpha : 0.6175001932496609; NN__hidden_layer_sizes : 3; NN__max_iter : 500; NN__random_state : 0; NN__solver : adam	0.707	0.733
NN	MM	ROS	NN__activation : identity; NN__alpha : 0.2644589949352923; NN__hidden_layer_sizes : 50; NN__max_iter : 50; NN__random_state : 0; NN__solver : adam	0.707	0.722
NN	MM	SMOTE	NN__activation : tanh; NN__alpha : 2.6096146808538574; NN__hidden_layer_sizes : 8; NN__max_iter : 478; NN__random_state : 0; NN__solver : adam	0.707	0.727
RF	SS	SMOTE	RF__class_weight : None; RF__criterion : gini; RF__max_depth : 1; RF__max_features : 1; RF__min_samples_leaf : 1; RF__n_estimators : 526; RF__random_state : 81	0.707	0.737
NN	MM	ROS	NN__activation : identity; NN__alpha : 0.008603525977298382; NN__hidden_layer_sizes : 7; NN__max_iter : 500; NN__random_state : 0; NN__solver : adam	0.707	0.730
RF	Not used	Not used	RF__class_weight : None; RF__criterion : log_loss; RF__max_depth : 1; RF__max_features : 1; RF__min_samples_leaf : 20; RF__n_estimators : 1000; RF__random_state : 0	0.707	0.749

RF	Not used	Not used	RF__class_weight : None; RF__criterion : gini; RF__max_depth : 1; RF__max_features : 1; RF__min_samples_leaf : 2; RF__n_estimators : 1000; RF__random_state : 0	0.707	0.754
RF	SS	ADASYN	RF__class_weight : balanced; RF__criterion : log_loss; RF__max_depth : 1; RF__max_features : 1; RF__min_samples_leaf : 17; RF__n_estimators : 602; RF__random_state : 31	0.707	0.737
RF	SS	ROS	RF__class_weight : balanced; RF__criterion : log_loss; RF__max_depth : 1; RF__max_features : 1; RF__min_samples_leaf : 20; RF__n_estimators : 1000; RF__random_state : 29	0.707	0.754
RF	SS	SMOTE	RF__class_weight : None; RF__criterion : log_loss; RF__max_depth : 1; RF__max_features : 1; RF__min_samples_leaf : 12; RF__n_estimators : 997; RF__random_state : 47	0.707	0.740
NN	MM	ADASYN	NN__activation : tanh; NN__alpha : 0.0023278258548970043; NN__hidden_layer_sizes : 50; NN__max_iter : 50; NN__random_state : 0; NN__solver : adam	0.707	0.722
NN	MM	SMOTE	NN__activation : relu; NN__alpha : 10.0; NN__hidden_layer_sizes : 50; NN__max_iter : 144; NN__random_state : 0; NN__solver : adam	0.707	0.717
NN	Not used	Not used	NN__activation : identity; NN__alpha : 1.0433147962664788; NN__hidden_layer_sizes : 35; NN__max_iter : 478; NN__random_state : 0; NN__solver : lbfgs	0.707	0.735
NN	SS	SMOTE	NN__activation : identity; NN__alpha : 0.0001; NN__hidden_layer_sizes : 3; NN__max_iter : 157; NN__random_state : 0; NN__solver : adam	0.707	0.728

RF	MM	ROS	RF__class_weight : None; RF__criterion : gini; RF__max_depth : 1; RF__max_features : 1; RF__min_samples_leaf : 2; RF__n_estimators : 300; RF__random_state : 0	0.707	0.755
RF	SS	ROS	RF__class_weight : balanced; RF__criterion : gini; RF__max_depth : 1; RF__max_features : 1; RF__min_samples_leaf : 9; RF__n_estimators : 300; RF__random_state : 0	0.707	0.755
NN	MM	SMOTE	NN__activation : tanh; NN__alpha : 10.0; NN__hidden_layer_sizes : 3; NN__max_iter : 500; NN__random_state : 0; NN__solver : adam	0.707	0.713
RF	SS	SMOTE	RF__class_weight : balanced; RF__criterion : gini; RF__max_depth : 1; RF__max_features : 1; RF__min_samples_leaf : 20; RF__n_estimators : 300; RF__random_state : 63	0.707	0.735
RF	MM	SMOTE	RF__class_weight : balanced_subsample; RF__criterion : log_loss; RF__max_depth : 1; RF__max_features : 1; RF__min_samples_leaf : 20; RF__n_estimators : 347; RF__random_state : 86	0.707	0.740
NN	MM	ADASYN	NN__activation : tanh; NN__alpha : 10.0; NN__hidden_layer_sizes : 8; NN__max_iter : 392; NN__random_state : 0; NN__solver : adam	0.706	0.715
NN	Not used	Not used	NN__activation : identity; NN__alpha : 0.0001; NN__hidden_layer_sizes : 27; NN__max_iter : 50; NN__random_state : 0; NN__solver : lbfgs	0.706	0.735
NN	MM	ROS	NN__activation : identity; NN__alpha : 0.0011418420214747349; NN__hidden_layer_sizes : 50; NN__max_iter : 147; NN__random_state : 0; NN__solver : adam	0.706	0.732
XGB	Not used	Not used	XGB__alpha : 7.166397157050396; XGB__colsample_bytree : 1.0; XGB__gamma : 0.1;	0.706	0.809

			XGB__max_depth : 3; XGB__min_child_weight : 0.0; XGB__random_state : 0; XGB__scale_pos_weight : 1.6024735100883754; XGB__subsample : 0.5121135299157635		
RF	MM	ROS	RF__class_weight : balanced_subsample; RF__criterion : log_loss; RF__max_depth : 1; RF__max_features : 1; RF__min_samples_leaf : 3; RF__n_estimators : 974; RF__random_state : 95	0.706	0.757
NN	MM	ADASYN	NN__activation : identity; NN__alpha : 0.0001; NN__hidden_layer_sizes : 50; NN__max_iter : 50; NN__random_state : 0; NN__solver : adam	0.706	0.721
XGB	Not used	Not used	XGB__alpha : 9.371570362967208; XGB__colsample_bytree : 1.0; XGB__gamma : 0.1; XGB__max_depth : 3; XGB__min_child_weight : 0.0; XGB__random_state : 0; XGB__scale_pos_weight : 2.0; XGB__subsample : 0.6396824807776188	0.706	0.800
NN	MM	SMOTE	NN__activation : tanh; NN__alpha : 10.0; NN__hidden_layer_sizes : 50; NN__max_iter : 359; NN__random_state : 0; NN__solver : adam	0.706	0.716
RF	SS	SMOTE	RF__class_weight : None; RF__criterion : gini; RF__max_depth : 1; RF__max_features : 1; RF__min_samples_leaf : 20; RF__n_estimators : 637; RF__random_state : 64	0.706	0.736
NN	Not used	Not used	NN__activation : identity; NN__alpha : 0.7973871592855735; NN__hidden_layer_sizes : 35; NN__max_iter : 270; NN__random_state : 0; NN__solver : lbfgs	0.706	0.735
NN	SS	SMOTE	NN__activation : identity; NN__alpha : 10.0; NN__hidden_layer_sizes : 3; NN__max_iter : 258; NN__random_state : 0; NN__solver : adam	0.706	0.732

NN	MM	SMOTE	NN__activation : identity; NN__alpha : 0.6841591798153179; NN__hidden_layer_sizes : 3; NN__max_iter : 500; NN__random_state : 0; NN__solver : adam	0.706	0.723
NN	MM	SMOTE	NN__activation : identity; NN__alpha : 0.1883823891017267; NN__hidden_layer_sizes : 35; NN__max_iter : 50; NN__random_state : 0; NN__solver : adam	0.706	0.725
NN	MM	ROS	NN__activation : identity; NN__alpha : 0.0001; NN__hidden_layer_sizes : 35; NN__max_iter : 500; NN__random_state : 0; NN__solver : adam	0.706	0.733
NN	SS	ROS	NN__activation : logistic; NN__alpha : 1.6800063358888258; NN__hidden_layer_sizes : 50; NN__max_iter : 500; NN__random_state : 0; NN__solver : adam	0.706	0.734
RF	SS	ROS	RF__class_weight : balanced_subsample; RF__criterion : log_loss; RF__max_depth : 2; RF__max_features : 1; RF__min_samples_leaf : 1; RF__n_estimators : 390; RF__random_state : 96	0.706	0.771
RF	MM	SMOTE	RF__class_weight : None; RF__criterion : log_loss; RF__max_depth : 1; RF__max_features : 1; RF__min_samples_leaf : 20; RF__n_estimators : 626; RF__random_state : 0	0.706	0.738
NN	SS	ROS	NN__activation : identity; NN__alpha : 10.0; NN__hidden_layer_sizes : 50; NN__max_iter : 236; NN__random_state : 0; NN__solver : lbfgs	0.706	0.734
RF	MM	ADASYN	RF__class_weight : None; RF__criterion : log_loss; RF__max_depth : 1; RF__max_features : 1; RF__min_samples_leaf : 1; RF__n_estimators : 412; RF__random_state : 0	0.706	0.735

NN	MM	ROS	NN__activation : tanh; NN__alpha : 0.20237980080690054; NN__hidden_layer_sizes : 24; NN__max_iter : 53; NN__random_state : 0; NN__solver : adam	0.706	0.718
NN	SS	ROS	NN__activation : logistic; NN__alpha : 0.43513970791520495; NN__hidden_layer_sizes : 40; NN__max_iter : 192; NN__random_state : 0; NN__solver : adam	0.706	0.734
RF	SS	ROS	RF__class_weight : balanced_subsample; RF__criterion : log_loss; RF__max_depth : 2; RF__max_features : 1; RF__min_samples_leaf : 1; RF__n_estimators : 744; RF__random_state : 2	0.706	0.770
NN	MM	SMOTE	NN__activation : logistic; NN__alpha : 0.43513970791520495; NN__hidden_layer_sizes : 40; NN__max_iter : 192; NN__random_state : 0; NN__solver : adam	0.706	0.721
NN	SS	SMOTE	NN__activation : identity; NN__alpha : 0.0026839782809033263; NN__hidden_layer_sizes : 3; NN__max_iter : 173; NN__random_state : 0; NN__solver : adam	0.705	0.730
NN	MM	SMOTE	NN__activation : identity; NN__alpha : 10.0; NN__hidden_layer_sizes : 50; NN__max_iter : 149; NN__random_state : 0; NN__solver : adam	0.705	0.718
NN	SS	ROS	NN__activation : identity; NN__alpha : 10.0; NN__hidden_layer_sizes : 3; NN__max_iter : 123; NN__random_state : 0; NN__solver : lbfgs	0.705	0.734
NN	MM	ROS	NN__activation : identity; NN__alpha : 0.016538821315471873; NN__hidden_layer_sizes : 50; NN__max_iter : 50; NN__random_state : 0; NN__solver : adam	0.705	0.722

RF	MM	ADASYN	RF__class_weight : None; RF__criterion : log_loss; RF__max_depth : 1; RF__max_features : 1; RF__min_samples_leaf : 1; RF__n_estimators : 401; RF__random_state : 0	0.705	0.735
NN	MM	ADASYN	NN__activation : identity; NN__alpha : 0.0001; NN__hidden_layer_sizes : 40; NN__max_iter : 50; NN__random_state : 0; NN__solver : adam	0.705	0.723
NN	MM	ROS	NN__activation : identity; NN__alpha : 0.0001; NN__hidden_layer_sizes : 27; NN__max_iter : 268; NN__random_state : 0; NN__solver : adam	0.705	0.732
RF	MM	ROS	RF__class_weight : None; RF__criterion : log_loss; RF__max_depth : 2; RF__max_features : 1; RF__min_samples_leaf : 20; RF__n_estimators : 300; RF__random_state : 0	0.705	0.767
RF	SS	ROS	RF__class_weight : None; RF__criterion : log_loss; RF__max_depth : 2; RF__max_features : 1; RF__min_samples_leaf : 20; RF__n_estimators : 300; RF__random_state : 0	0.705	0.767
NN	MM	SMOTE	NN__activation : tanh; NN__alpha : 0.0011892310453067918; NN__hidden_layer_sizes : 50; NN__max_iter : 50; NN__random_state : 0; NN__solver : adam	0.705	0.721
RF	MM	ROS	RF__class_weight : balanced_subsample; RF__criterion : log_loss; RF__max_depth : 2; RF__max_features : 1; RF__min_samples_leaf : 7; RF__n_estimators : 890; RF__random_state : 16	0.705	0.771
RF	MM	ROS	RF__class_weight : balanced; RF__criterion : gini; RF__max_depth : 1; RF__max_features : 1; RF__min_samples_leaf : 6; RF__n_estimators : 1000; RF__random_state : 0	0.705	0.754

RF	Not used	Not used	RF__class_weight : None; RF__criterion : log_loss; RF__max_depth : 1; RF__max_features : 1; RF__min_samples_leaf : 2; RF__n_estimators : 1000; RF__random_state : 100	0.705	0.757
RF	SS	ROS	RF__class_weight : balanced; RF__criterion : log_loss; RF__max_depth : 2; RF__max_features : 1; RF__min_samples_leaf : 17; RF__n_estimators : 380; RF__random_state : 53	0.705	0.766
NN	MM	SMOTE	NN__activation : tanh; NN__alpha : 0.0001; NN__hidden_layer_sizes : 50; NN__max_iter : 50; NN__random_state : 0; NN__solver : adam	0.705	0.721
NN	MM	SMOTE	NN__activation : identity; NN__alpha : 0.021376539749443257; NN__hidden_layer_sizes : 50; NN__max_iter : 50; NN__random_state : 0; NN__solver : adam	0.705	0.720
NN	SS	SMOTE	NN__activation : identity; NN__alpha : 0.0001; NN__hidden_layer_sizes : 3; NN__max_iter : 132; NN__random_state : 0; NN__solver : adam	0.705	0.722
NN	SS	ROS	NN__activation : identity; NN__alpha : 0.3848820267958592; NN__hidden_layer_sizes : 3; NN__max_iter : 149; NN__random_state : 0; NN__solver : lbfgs	0.705	0.733
NN	SS	ROS	NN__activation : logistic; NN__alpha : 3.9229485139656597; NN__hidden_layer_sizes : 5; NN__max_iter : 245; NN__random_state : 0; NN__solver : adam	0.705	0.724
NN	MM	ROS	NN__activation : tanh; NN__alpha : 2.688786902631166; NN__hidden_layer_sizes : 30; NN__max_iter : 500; NN__random_state : 0; NN__solver : adam	0.705	0.727

NN	MM	ADASYN	NN__activation : identity; NN__alpha : 10.0; NN__hidden_layer_sizes : 9; NN__max_iter : 500; NN__random_state : 0; NN__solver : lbfgs	0.705	0.729
NN	MM	SMOTE	NN__activation : identity; NN__alpha : 0.3653960181115047; NN__hidden_layer_sizes : 50; NN__max_iter : 50; NN__random_state : 0; NN__solver : adam	0.705	0.720
RF	SS	ROS	RF__class_weight : balanced_subsample; RF__criterion : log_loss; RF__max_depth : 1; RF__max_features : 1; RF__min_samples_leaf : 1; RF__n_estimators : 712; RF__random_state : 98	0.705	0.751
RF	MM	ADASYN	RF__class_weight : balanced; RF__criterion : log_loss; RF__max_depth : 1; RF__max_features : 1; RF__min_samples_leaf : 5; RF__n_estimators : 418; RF__random_state : 0	0.705	0.736
NN	MM	ROS	NN__activation : identity; NN__alpha : 1.1355598656299941; NN__hidden_layer_sizes : 50; NN__max_iter : 500; NN__random_state : 0; NN__solver : adam	0.704	0.732
NN	MM	ROS	NN__activation : tanh; NN__alpha : 10.0; NN__hidden_layer_sizes : 22; NN__max_iter : 50; NN__random_state : 0; NN__solver : lbfgs	0.704	0.727
NN	MM	ROS	NN__activation : identity; NN__alpha : 0.0001; NN__hidden_layer_sizes : 50; NN__max_iter : 500; NN__random_state : 0; NN__solver : adam	0.704	0.732
NN	MM	ADASYN	NN__activation : identity; NN__alpha : 0.4224729525506053; NN__hidden_layer_sizes : 50; NN__max_iter : 50; NN__random_state : 0; NN__solver : adam	0.704	0.721

NN	MM	ADASYN	NN__activation : identity; NN__alpha : 10.0; NN__hidden_layer_sizes : 6; NN__max_iter : 50; NN__random_state : 0; NN__solver : lbfgs	0.704	0.729
NN	SS	SMOTE	NN__activation : identity; NN__alpha : 10.0; NN__hidden_layer_sizes : 3; NN__max_iter : 301; NN__random_state : 0; NN__solver : adam	0.704	0.733
RF	SS	ROS	RF__class_weight : None; RF__criterion : log_loss; RF__max_depth : 1; RF__max_features : 1; RF__min_samples_leaf : 3; RF__n_estimators : 300; RF__random_state : 29	0.704	0.753
RF	MM	ADASYN	RF__class_weight : None; RF__criterion : log_loss; RF__max_depth : 1; RF__max_features : 1; RF__min_samples_leaf : 5; RF__n_estimators : 370; RF__random_state : 0	0.704	0.735
NN	SS	ROS	NN__activation : identity; NN__alpha : 0.2516451633834142; NN__hidden_layer_sizes : 3; NN__max_iter : 50; NN__random_state : 0; NN__solver : lbfgs	0.704	0.733
RF	SS	SMOTE	RF__class_weight : None; RF__criterion : log_loss; RF__max_depth : 1; RF__max_features : 1; RF__min_samples_leaf : 14; RF__n_estimators : 821; RF__random_state : 4	0.704	0.739
RF	MM	ROS	RF__class_weight : balanced_subsample; RF__criterion : log_loss; RF__max_depth : 1; RF__max_features : 1; RF__min_samples_leaf : 20; RF__n_estimators : 1000; RF__random_state : 23	0.704	0.753
RF	MM	ADASYN	RF__class_weight : None; RF__criterion : log_loss; RF__max_depth : 1; RF__max_features : 1; RF__min_samples_leaf : 1; RF__n_estimators : 402; RF__random_state : 0	0.704	0.736

RF	SS	SMOTE	RF__class_weight : balanced; RF__criterion : log_loss; RF__max_depth : 1; RF__max_features : 1; RF__min_samples_leaf : 1; RF__n_estimators : 861; RF__random_state : 1	0.704	0.740
RF	MM	ROS	RF__class_weight : None; RF__criterion : log_loss; RF__max_depth : 1; RF__max_features : 1; RF__min_samples_leaf : 1; RF__n_estimators : 505; RF__random_state : 36	0.704	0.755
NN	SS	ROS	NN__activation : identity; NN__alpha : 0.0117356998807354; NN__hidden_layer_sizes : 50; NN__max_iter : 205; NN__random_state : 0; NN__solver : lbfgs	0.704	0.733
RF	Not used	Not used	RF__class_weight : balanced; RF__criterion : log_loss; RF__max_depth : 1; RF__max_features : 1; RF__min_samples_leaf : 10; RF__n_estimators : 307; RF__random_state : 100	0.704	0.752
RF	SS	ADASYN	RF__class_weight : balanced; RF__criterion : log_loss; RF__max_depth : 1; RF__max_features : 1; RF__min_samples_leaf : 1; RF__n_estimators : 1000; RF__random_state : 100	0.704	0.738
NN	MM	ADASYN	NN__activation : identity; NN__alpha : 10.0; NN__hidden_layer_sizes : 22; NN__max_iter : 183; NN__random_state : 0; NN__solver : lbfgs	0.704	0.729
NN	MM	SMOTE	NN__activation : identity; NN__alpha : 1.2278635248432284; NN__hidden_layer_sizes : 27; NN__max_iter : 165; NN__random_state : 0; NN__solver : adam	0.704	0.729
RF	MM	ROS	RF__class_weight : balanced; RF__criterion : log_loss; RF__max_depth : 1; RF__max_features : 1; RF__min_samples_leaf : 20; RF__n_estimators : 1000; RF__random_state : 47	0.704	0.756

NN	MM	SMOTE	NN__activation : logistic; NN__alpha : 10.0; NN__hidden_layer_sizes : 50; NN__max_iter : 343; NN__random_state : 0; NN__solver : adam	0.704	0.711
NN	MM	SMOTE	NN__activation : identity; NN__alpha : 10.0; NN__hidden_layer_sizes : 3; NN__max_iter : 500; NN__random_state : 0; NN__solver : adam	0.704	0.710
NN	SS	SMOTE	NN__activation : relu; NN__alpha : 10.0; NN__hidden_layer_sizes : 3; NN__max_iter : 311; NN__random_state : 0; NN__solver : adam	0.704	0.733
NN	MM	SMOTE	NN__activation : tanh; NN__alpha : 2.0451523048003124; NN__hidden_layer_sizes : 3; NN__max_iter : 500; NN__random_state : 0; NN__solver : adam	0.704	0.726
NN	MM	ADASYN	NN__activation : identity; NN__alpha : 10.0; NN__hidden_layer_sizes : 30; NN__max_iter : 500; NN__random_state : 0; NN__solver : lbfgs	0.703	0.729
NN	SS	ROS	NN__activation : identity; NN__alpha : 1.3069165088825327; NN__hidden_layer_sizes : 50; NN__max_iter : 500; NN__random_state : 0; NN__solver : adam	0.703	0.733
NN	SS	ROS	NN__activation : identity; NN__alpha : 1.2278635248432284; NN__hidden_layer_sizes : 27; NN__max_iter : 165; NN__random_state : 0; NN__solver : adam	0.703	0.733
NN	MM	ADASYN	NN__activation : identity; NN__alpha : 10.0; NN__hidden_layer_sizes : 50; NN__max_iter : 50; NN__random_state : 0; NN__solver : adam	0.703	0.713
RF	MM	ROS	RF__class_weight : balanced; RF__criterion : log_loss; RF__max_depth : 2; RF__max_features : 1; RF__min_samples_leaf : 1; RF__n_estimators : 878; RF__random_state : 0	0.703	0.771

RF	SS	SMOTE	RF__class_weight : balanced; RF__criterion : log_loss; RF__max_depth : 1; RF__max_features : 1; RF__min_samples_leaf : 20; RF__n_estimators : 386; RF__random_state : 98	0.703	0.737
NN	MM	SMOTE	NN__activation : tanh; NN__alpha : 10.0; NN__hidden_layer_sizes : 50; NN__max_iter : 500; NN__random_state : 0; NN__solver : lbfgs	0.703	0.726
RF	MM	ROS	RF__class_weight : balanced; RF__criterion : log_loss; RF__max_depth : 1; RF__max_features : 1; RF__min_samples_leaf : 20; RF__n_estimators : 1000; RF__random_state : 71	0.703	0.754
NN	MM	ADASYN	NN__activation : tanh; NN__alpha : 10.0; NN__hidden_layer_sizes : 22; NN__max_iter : 50; NN__random_state : 0; NN__solver : lbfgs	0.703	0.729
NN	MM	SMOTE	NN__activation : identity; NN__alpha : 8.954426783777631; NN__hidden_layer_sizes : 25; NN__max_iter : 429; NN__random_state : 0; NN__solver : lbfgs	0.703	0.727
NN	SS	SMOTE	NN__activation : identity; NN__alpha : 10.0; NN__hidden_layer_sizes : 3; NN__max_iter : 373; NN__random_state : 0; NN__solver : adam	0.703	0.733
NN	MM	ADASYN	NN__activation : tanh; NN__alpha : 1.0479599859326938; NN__hidden_layer_sizes : 7; NN__max_iter : 406; NN__random_state : 0; NN__solver : adam	0.703	0.731
NN	MM	SMOTE	NN__activation : identity; NN__alpha : 10.0; NN__hidden_layer_sizes : 27; NN__max_iter : 334; NN__random_state : 0; NN__solver : adam	0.703	0.715
NN	MM	ADASYN	NN__activation : tanh; NN__alpha : 0.0014153375045616748; NN__hidden_layer_sizes : 30;	0.703	0.725

			NN__max_iter : 50; NN__random_state : 0; NN__solver : adam		
NN	Not used	Not used	NN__activation : identity; NN__alpha : 1.4346575857584027; NN__hidden_layer_sizes : 35; NN__max_iter : 404; NN__random_state : 0; NN__solver : lbfgs	0.703	0.735
NN	SS	SMOTE	NN__activation : identity; NN__alpha : 0.0001; NN__hidden_layer_sizes : 3; NN__max_iter : 131; NN__random_state : 0; NN__solver : adam	0.703	0.721
NN	MM	ADASYN	NN__activation : tanh; NN__alpha : 10.0; NN__hidden_layer_sizes : 50; NN__max_iter : 61; NN__random_state : 0; NN__solver : adam	0.703	0.716
NN	MM	ADASYN	NN__activation : logistic; NN__alpha : 10.0; NN__hidden_layer_sizes : 50; NN__max_iter : 281; NN__random_state : 0; NN__solver : adam	0.703	0.712
XGB	Not used	Not used	XGB__alpha : 6.92724037969123; XGB__colsample_bytree : 1.0; XGB__gamma : 0.1; XGB__max_depth : 3; XGB__min_child_weight : 0.0; XGB__random_state : 0; XGB__scale_pos_weight : 1.5185948753904954; XGB__subsample : 0.5	0.703	0.809
NN	MM	ADASYN	NN__activation : identity; NN__alpha : 0.0001; NN__hidden_layer_sizes : 3; NN__max_iter : 500; NN__random_state : 0; NN__solver : adam	0.703	0.728
XGB	Not used	Not used	XGB__alpha : 8.171350453632432; XGB__colsample_bytree : 1.0; XGB__gamma : 0.1; XGB__max_depth : 3; XGB__min_child_weight : 0.0; XGB__random_state : 0; XGB__scale_pos_weight : 2.0; XGB__subsample : 0.5	0.703	0.812
RF	MM	ADASYN	RF__class_weight : None; RF__criterion : log_loss; RF__max_depth : 1; RF__max_features : 1;	0.703	0.736

			RF__min_samples_leaf : 1; RF__n_estimators : 542; RF__random_state : 0		
NN	MM	SMOTE	NN__activation : identity; NN__alpha : 1.6098539665726266; NN__hidden_layer_sizes : 9; NN__max_iter : 500; NN__random_state : 0; NN__solver : adam	0.703	0.730
NN	MM	SMOTE	NN__activation : relu; NN__alpha : 3.9814946105106603; NN__hidden_layer_sizes : 15; NN__max_iter : 427; NN__random_state : 0; NN__solver : adam	0.703	0.723
NN	MM	ADASYN	NN__activation : identity; NN__alpha : 10.0; NN__hidden_layer_sizes : 40; NN__max_iter : 500; NN__random_state : 0; NN__solver : lbfgs	0.703	0.729
NN	MM	SMOTE	NN__activation : identity; NN__alpha : 10.0; NN__hidden_layer_sizes : 27; NN__max_iter : 197; NN__random_state : 0; NN__solver : adam	0.703	0.715
NN	SS	SMOTE	NN__activation : identity; NN__alpha : 0.0001; NN__hidden_layer_sizes : 3; NN__max_iter : 196; NN__random_state : 0; NN__solver : adam	0.702	0.732
NN	MM	ADASYN	NN__activation : relu; NN__alpha : 10.0; NN__hidden_layer_sizes : 50; NN__max_iter : 163; NN__random_state : 0; NN__solver : adam	0.702	0.718
NN	SS	SMOTE	NN__activation : logistic; NN__alpha : 3.9229485139656597; NN__hidden_layer_sizes : 5; NN__max_iter : 245; NN__random_state : 0; NN__solver : adam	0.702	0.717
RF	MM	ADASYN	RF__class_weight : None; RF__criterion : log_loss; RF__max_depth : 1; RF__max_features : 1; RF__min_samples_leaf : 1; RF__n_estimators : 527; RF__random_state : 0	0.702	0.736

RF	MM	ADASYN	RF__class_weight : balanced; RF__criterion : log_loss; RF__max_depth : 1; RF__max_features : 1; RF__min_samples_leaf : 20; RF__n_estimators : 473; RF__random_state : 0	0.702	0.735
RF	MM	ADASYN	RF__class_weight : None; RF__criterion : log_loss; RF__max_depth : 1; RF__max_features : 1; RF__min_samples_leaf : 20; RF__n_estimators : 407; RF__random_state : 0	0.702	0.733
RF	MM	ROS	RF__class_weight : balanced; RF__criterion : log_loss; RF__max_depth : 2; RF__max_features : 1; RF__min_samples_leaf : 20; RF__n_estimators : 1000; RF__random_state : 0	0.702	0.768
RF	MM	ROS	RF__class_weight : balanced_subsample; RF__criterion : log_loss; RF__max_depth : 2; RF__max_features : 1; RF__min_samples_leaf : 2; RF__n_estimators : 956; RF__random_state : 99	0.702	0.770
NN	MM	ADASYN	NN__activation : tanh; NN__alpha : 10.0; NN__hidden_layer_sizes : 8; NN__max_iter : 211; NN__random_state : 0; NN__solver : lbfgs	0.702	0.729
NN	SS	SMOTE	NN__activation : identity; NN__alpha : 0.22138143217297065; NN__hidden_layer_sizes : 3; NN__max_iter : 247; NN__random_state : 0; NN__solver : adam	0.702	0.732
NN	MM	ADASYN	NN__activation : identity; NN__alpha : 0.5298558379337189; NN__hidden_layer_sizes : 40; NN__max_iter : 50; NN__random_state : 0; NN__solver : adam	0.702	0.722
XGB	Not used	Not used	XGB__alpha : 5.0; XGB__colsample_bytree : 0.8376896768834488; XGB__gamma : 0.1; XGB__max_depth : 1; XGB__min_child_weight : 0.0;	0.702	0.762

			XGB__random_state : 0; XGB__scale_pos_weight : 1.298280204682432; XGB__subsample : 0.5		
NN	SS	SMOTE	NN__activation : tanh; NN__alpha : 10.0; NN__hidden_layer_sizes : 3; NN__max_iter : 335; NN__random_state : 0; NN__solver : adam	0.702	0.734
NN	SS	ROS	NN__activation : relu; NN__alpha : 10.0; NN__hidden_layer_sizes : 3; NN__max_iter : 136; NN__random_state : 0; NN__solver : lbfgs	0.701	0.748
NN	SS	SMOTE	NN__activation : identity; NN__alpha : 0.03399605266487899; NN__hidden_layer_sizes : 3; NN__max_iter : 500; NN__random_state : 0; NN__solver : adam	0.701	0.731
NN	MM	ADASYN	NN__activation : relu; NN__alpha : 3.9814946105106603; NN__hidden_layer_sizes : 15; NN__max_iter : 427; NN__random_state : 0; NN__solver : adam	0.701	0.724
NN	MM	ADASYN	NN__activation : logistic; NN__alpha : 10.0; NN__hidden_layer_sizes : 27; NN__max_iter : 50; NN__random_state : 0; NN__solver : lbfgs	0.701	0.714
RF	SS	ADASYN	RF__class_weight : balanced; RF__criterion : gini; RF__max_depth : 1; RF__max_features : 1; RF__min_samples_leaf : 1; RF__n_estimators : 300; RF__random_state : 0	0.701	0.733
NN	MM	ADASYN	NN__activation : logistic; NN__alpha : 0.43513970791520495; NN__hidden_layer_sizes : 40; NN__max_iter : 192; NN__random_state : 0; NN__solver : adam	0.701	0.722
RF	MM	ROS	RF__class_weight : balanced; RF__criterion : log_loss; RF__max_depth : 1; RF__max_features : 1; RF__min_samples_leaf : 20; RF__n_estimators : 300; RF__random_state : 76	0.701	0.753

NN	SS	ROS	NN__activation : identity; NN__alpha : 0.0001; NN__hidden_layer_sizes : 50; NN__max_iter : 221; NN__random_state : 0; NN__solver : adam	0.701	0.733
NN	MM	ADASYN	NN__activation : tanh; NN__alpha : 10.0; NN__hidden_layer_sizes : 6; NN__max_iter : 395; NN__random_state : 0; NN__solver : lbfgs	0.701	0.729
XGB	Not used	Not used	XGB__alpha : 9.05087965932921; XGB__colsample_bytree : 1.0; XGB__gamma : 0.1; XGB__max_depth : 3; XGB__min_child_weight : 0.0; XGB__random_state : 0; XGB__scale_pos_weight : 2.0; XGB__subsample : 0.5199689455342374	0.701	0.798
XGB	Not used	Not used	XGB__alpha : 8.196217519929151; XGB__colsample_bytree : 1.0; XGB__gamma : 0.1; XGB__max_depth : 3; XGB__min_child_weight : 0.0; XGB__random_state : 0; XGB__scale_pos_weight : 2.0; XGB__subsample : 0.5208769421800412	0.701	0.810
NN	MM	ADASYN	NN__activation : logistic; NN__alpha : 10.0; NN__hidden_layer_sizes : 15; NN__max_iter : 50; NN__random_state : 0; NN__solver : lbfgs	0.701	0.714
RF	MM	ADASYN	RF__class_weight : None; RF__criterion : log_loss; RF__max_depth : 1; RF__max_features : 1; RF__min_samples_leaf : 1; RF__n_estimators : 515; RF__random_state : 0	0.700	0.736
RF	MM	ADASYN	RF__class_weight : None; RF__criterion : log_loss; RF__max_depth : 1; RF__max_features : 1; RF__min_samples_leaf : 1; RF__n_estimators : 502; RF__random_state : 0	0.700	0.736
NN	Not used	Not used	NN__activation : tanh; NN__alpha : 0.0001; NN__hidden_layer_sizes : 20; NN__max_iter : 500; NN__random_state : 0; NN__solver : adam	0.700	0.725

NN	MM	ADASYN	NN__activation : relu; NN__alpha : 10.0; NN__hidden_layer_sizes : 3; NN__max_iter : 500; NN__random_state : 0; NN__solver : adam	0.700	0.712
RF	Not used	Not used	RF__class_weight : balanced; RF__criterion : log_loss; RF__max_depth : 2; RF__max_features : 1; RF__min_samples_leaf : 1; RF__n_estimators : 1000; RF__random_state : 100	0.700	0.771
XGB	Not used	Not used	XGB__alpha : 7.1892679279518354; XGB__colsample_bytree : 1.0; XGB__gamma : 0.1; XGB__max_depth : 3; XGB__min_child_weight : 0.0; XGB__random_state : 0; XGB__scale_pos_weight : 1.5839827095911299; XGB__subsample : 0.6736377307490001	0.700	0.815
RF	MM	ROS	RF__class_weight : balanced_subsample; RF__criterion : log_loss; RF__max_depth : 2; RF__max_features : 1; RF__min_samples_leaf : 1; RF__n_estimators : 333; RF__random_state : 58	0.700	0.770
RF	MM	ADASYN	RF__class_weight : None; RF__criterion : log_loss; RF__max_depth : 1; RF__max_features : 1; RF__min_samples_leaf : 1; RF__n_estimators : 514; RF__random_state : 0	0.700	0.736
RF	MM	ADASYN	RF__class_weight : None; RF__criterion : log_loss; RF__max_depth : 1; RF__max_features : 1; RF__min_samples_leaf : 1; RF__n_estimators : 468; RF__random_state : 0	0.700	0.737
NN	SS	SMOTE	NN__activation : logistic; NN__alpha : 10.0; NN__hidden_layer_sizes : 9; NN__max_iter : 117; NN__random_state : 0; NN__solver : lbfgs	0.700	0.734
NN	SS	ADASYN	NN__activation : tanh; NN__alpha : 10.0; NN__hidden_layer_sizes : 50; NN__max_iter : 500; NN__random_state : 0; NN__solver : adam	0.700	0.733

XGB	Not used	Not used	XGB__alpha : 9.526170761453628; XGB__colsample_bytree : 1.0; XGB__gamma : 0.1; XGB__max_depth : 3; XGB__min_child_weight : 0.0; XGB__random_state : 0; XGB__scale_pos_weight : 2.0; XGB__subsample : 0.7	0.700	0.800
NN	MM	ADASYN	NN__activation : tanh; NN__alpha : 10.0; NN__hidden_layer_sizes : 7; NN__max_iter : 500; NN__random_state : 0; NN__solver : adam	0.700	0.719
NN	SS	ADASYN	NN__activation : identity; NN__alpha : 10.0; NN__hidden_layer_sizes : 50; NN__max_iter : 500; NN__random_state : 0; NN__solver : adam	0.699	0.733
RF	Not used	Not used	RF__class_weight : None; RF__criterion : gini; RF__max_depth : 2; RF__max_features : 1; RF__min_samples_leaf : 1; RF__n_estimators : 300; RF__random_state : 0	0.699	0.770
RF	SS	ADASYN	RF__class_weight : balanced_subsample; RF__criterion : gini; RF__max_depth : 1; RF__max_features : 1; RF__min_samples_leaf : 1; RF__n_estimators : 484; RF__random_state : 85	0.699	0.734
RF	MM	ROS	RF__class_weight : balanced; RF__criterion : log_loss; RF__max_depth : 2; RF__max_features : 1; RF__min_samples_leaf : 20; RF__n_estimators : 300; RF__random_state : 100	0.699	0.764
RF	SS	ROS	RF__class_weight : balanced; RF__criterion : log_loss; RF__max_depth : 2; RF__max_features : 1; RF__min_samples_leaf : 20; RF__n_estimators : 300; RF__random_state : 100	0.699	0.764
RF	SS	ROS	RF__class_weight : None; RF__criterion : gini; RF__max_depth : 2; RF__max_features : 1; RF__min_samples_leaf : 1; RF__n_estimators : 504; RF__random_state : 14	0.699	0.772

NN	SS	SMOTE	NN__activation : identity; NN__alpha : 10.0; NN__hidden_layer_sizes : 10; NN__max_iter : 447; NN__random_state : 0; NN__solver : adam	0.699	0.734
RF	MM	ROS	RF__class_weight : balanced; RF__criterion : log_loss; RF__max_depth : 3; RF__max_features : 1; RF__min_samples_leaf : 2; RF__n_estimators : 300; RF__random_state : 72	0.699	0.790
RF	MM	ADASYN	RF__class_weight : balanced_subsample; RF__criterion : log_loss; RF__max_depth : 1; RF__max_features : 1; RF__min_samples_leaf : 1; RF__n_estimators : 535; RF__random_state : 0	0.699	0.736
RF	MM	ADASYN	RF__class_weight : None; RF__criterion : log_loss; RF__max_depth : 1; RF__max_features : 1; RF__min_samples_leaf : 5; RF__n_estimators : 450; RF__random_state : 0	0.699	0.737
RF	MM	SMOTE	RF__class_weight : balanced_subsample; RF__criterion : log_loss; RF__max_depth : 1; RF__max_features : 1; RF__min_samples_leaf : 20; RF__n_estimators : 1000; RF__random_state : 0	0.698	0.738
RF	SS	SMOTE	RF__class_weight : None; RF__criterion : gini; RF__max_depth : 1; RF__max_features : 1; RF__min_samples_leaf : 9; RF__n_estimators : 999; RF__random_state : 4	0.698	0.735
RF	MM	ADASYN	RF__class_weight : None; RF__criterion : log_loss; RF__max_depth : 1; RF__max_features : 1; RF__min_samples_leaf : 20; RF__n_estimators : 533; RF__random_state : 0	0.698	0.734
RF	SS	ROS	RF__class_weight : balanced_subsample; RF__criterion : gini; RF__max_depth : 1; RF__max_features : 1; RF__min_samples_leaf : 20; RF__n_estimators : 300; RF__random_state : 26	0.698	0.754

NN	Not used	Not used	NN__activation : tanh; NN__alpha : 0.18681865077814802; NN__hidden_layer_sizes : 27; NN__max_iter : 349; NN__random_state : 0; NN__solver : adam	0.698	0.756
RF	SS	SMOTE	RF__class_weight : None; RF__criterion : gini; RF__max_depth : 1; RF__max_features : 1; RF__min_samples_leaf : 15; RF__n_estimators : 775; RF__random_state : 9	0.698	0.735
XGB	MM	ROS	XGB__alpha : 5.0; XGB__colsample_bytree : 1.0; XGB__gamma : 0.1; XGB__max_depth : 1; XGB__min_child_weight : 2.796923680739347; XGB__random_state : 0; XGB__scale_pos_weight : 1.8812267303489647; XGB__subsample : 0.7	0.698	0.801
XGB	Not used	Not used	XGB__alpha : 6.4802030805053175; XGB__colsample_bytree : 1.0; XGB__gamma : 0.1; XGB__max_depth : 3; XGB__min_child_weight : 0.0; XGB__random_state : 0; XGB__scale_pos_weight : 1.5154649125645612; XGB__subsample : 0.5	0.698	0.817
RF	Not used	Not used	RF__class_weight : balanced; RF__criterion : gini; RF__max_depth : 1; RF__max_features : 1; RF__min_samples_leaf : 1; RF__n_estimators : 1000; RF__random_state : 100	0.698	0.756
RF	MM	ADASYN	RF__class_weight : None; RF__criterion : log_loss; RF__max_depth : 1; RF__max_features : 1; RF__min_samples_leaf : 20; RF__n_estimators : 519; RF__random_state : 0	0.697	0.734
RF	MM	ADASYN	RF__class_weight : None; RF__criterion : log_loss; RF__max_depth : 1; RF__max_features : 1; RF__min_samples_leaf : 1; RF__n_estimators : 300; RF__random_state : 0	0.697	0.734

RF	SS	ADASYN	RF__class_weight : None; RF__criterion : gini; RF__max_depth : 1; RF__max_features : 1; RF__min_samples_leaf : 20; RF__n_estimators : 650; RF__random_state : 0	0.697	0.730
NN	SS	ADASYN	NN__activation : logistic; NN__alpha : 0.43513970791520495; NN__hidden_layer_sizes : 40; NN__max_iter : 192; NN__random_state : 0; NN__solver : adam	0.697	0.732
NN	Not used	Not used	NN__activation : identity; NN__alpha : 3.97855097535385; NN__hidden_layer_sizes : 4; NN__max_iter : 62; NN__random_state : 0; NN__solver : lbfgs	0.697	0.731
XGB	MM	ROS	XGB__alpha : 5.0; XGB__colsample_bytree : 1.0; XGB__gamma : 0.1; XGB__max_depth : 1; XGB__min_child_weight : 0.0; XGB__random_state : 0; XGB__scale_pos_weight : 2.0; XGB__subsample : 0.7	0.697	0.801
XGB	Not used	Not used	XGB__alpha : 8.443102490629965; XGB__colsample_bytree : 1.0; XGB__gamma : 0.1; XGB__max_depth : 3; XGB__min_child_weight : 0.0; XGB__random_state : 0; XGB__scale_pos_weight : 2.0; XGB__subsample : 0.5	0.697	0.808
RF	MM	ADASYN	RF__class_weight : balanced_subsample; RF__criterion : log_loss; RF__max_depth : 1; RF__max_features : 1; RF__min_samples_leaf : 15; RF__n_estimators : 552; RF__random_state : 0	0.697	0.736
RF	SS	ROS	RF__class_weight : None; RF__criterion : log_loss; RF__max_depth : 1; RF__max_features : 1; RF__min_samples_leaf : 1; RF__n_estimators : 302; RF__random_state : 53	0.697	0.756

XGB	Not used	Not used	XGB__alpha : 5.0; XGB__colsample_bytree : 1.0; XGB__gamma : 0.1; XGB__max_depth : 3; XGB__min_child_weight : 0.0; XGB__random_state : 0; XGB__scale_pos_weight : 1.4571087307953596; XGB__subsample : 0.5274138585267871	0.697	0.850
RF	MM	ADASYN	RF__class_weight : None; RF__criterion : log_loss; RF__max_depth : 1; RF__max_features : 1; RF__min_samples_leaf : 1; RF__n_estimators : 1000; RF__random_state : 0	0.697	0.734
RF	SS	SMOTE	RF__class_weight : None; RF__criterion : gini; RF__max_depth : 1; RF__max_features : 1; RF__min_samples_leaf : 1; RF__n_estimators : 378; RF__random_state : 35	0.697	0.733
RF	SS	SMOTE	RF__class_weight : balanced_subsample; RF__criterion : log_loss; RF__max_depth : 1; RF__max_features : 1; RF__min_samples_leaf : 1; RF__n_estimators : 1000; RF__random_state : 100	0.697	0.739
NN	Not used	Not used	NN__activation : tanh; NN__alpha : 0.13140976907192184; NN__hidden_layer_sizes : 30; NN__max_iter : 500; NN__random_state : 0; NN__solver : adam	0.697	0.761
RF	MM	ADASYN	RF__class_weight : balanced; RF__criterion : log_loss; RF__max_depth : 1; RF__max_features : 1; RF__min_samples_leaf : 3; RF__n_estimators : 364; RF__random_state : 0	0.697	0.735
NN	Not used	Not used	NN__activation : relu; NN__alpha : 0.003366028940110959; NN__hidden_layer_sizes : 50; NN__max_iter : 50; NN__random_state : 0; NN__solver : lbfgs	0.697	0.743
RF	SS	ADASYN	RF__class_weight : balanced; RF__criterion : log_loss; RF__max_depth : 2; RF__max_features : 1;	0.696	0.749

			RF__min_samples_leaf : 3; RF__n_estimators : 880; RF__random_state : 95		
XGB	Not used	Not used	XGB__alpha : 8.280818556474479; XGB__colsample_bytree : 1.0; XGB__gamma : 0.1; XGB__max_depth : 2; XGB__min_child_weight : 0.0; XGB__random_state : 0; XGB__scale_pos_weight : 1.3954288288051107; XGB__subsample : 0.5	0.696	0.764
NN	Not used	Not used	NN__activation : tanh; NN__alpha : 0.3184983766348907; NN__hidden_layer_sizes : 35; NN__max_iter : 237; NN__random_state : 0; NN__solver : adam	0.696	0.753
RF	SS	SMOTE	RF__class_weight : None; RF__criterion : gini; RF__max_depth : 1; RF__max_features : 1; RF__min_samples_leaf : 1; RF__n_estimators : 833; RF__random_state : 35	0.696	0.735
RF	Not used	Not used	RF__class_weight : balanced; RF__criterion : gini; RF__max_depth : 1; RF__max_features : 1; RF__min_samples_leaf : 20; RF__n_estimators : 1000; RF__random_state : 100	0.696	0.752
RF	MM	SMOTE	RF__class_weight : None; RF__criterion : gini; RF__max_depth : 1; RF__max_features : 1; RF__min_samples_leaf : 20; RF__n_estimators : 1000; RF__random_state : 85	0.696	0.736
RF	MM	ADASYN	RF__class_weight : None; RF__criterion : log_loss; RF__max_depth : 1; RF__max_features : 1; RF__min_samples_leaf : 1; RF__n_estimators : 693; RF__random_state : 0	0.696	0.734
XGB	Not used	Not used	XGB__alpha : 11.655829440952115; XGB__colsample_bytree : 0.9744062933741346; XGB__gamma : 0.1; XGB__max_depth : 3; XGB__min_child_weight : 0.0; XGB__random_state :	0.696	0.776

			0; XGB__scale_pos_weight : 2.0; XGB__subsample : 0.7		
RF	SS	SMOTE	RF__class_weight : balanced; RF__criterion : log_loss; RF__max_depth : 1; RF__max_features : 1; RF__min_samples_leaf : 20; RF__n_estimators : 300; RF__random_state : 32	0.696	0.736
NN	SS	ADASYN	NN__activation : identity; NN__alpha : 10.0; NN__hidden_layer_sizes : 8; NN__max_iter : 427; NN__random_state : 0; NN__solver : adam	0.695	0.732
RF	SS	ROS	RF__class_weight : None; RF__criterion : gini; RF__max_depth : 1; RF__max_features : 1; RF__min_samples_leaf : 13; RF__n_estimators : 996; RF__random_state : 95	0.695	0.757
RF	MM	SMOTE	RF__class_weight : None; RF__criterion : log_loss; RF__max_depth : 1; RF__max_features : 1; RF__min_samples_leaf : 1; RF__n_estimators : 1000; RF__random_state : 0	0.695	0.739
XGB	Not used	Not used	XGB__alpha : 7.243938265936928; XGB__colsample_bytree : 1.0; XGB__gamma : 0.1; XGB__max_depth : 3; XGB__min_child_weight : 0.0; XGB__random_state : 0; XGB__scale_pos_weight : 1.5328972807761156; XGB__subsample : 0.7	0.695	0.809
XGB	Not used	Not used	XGB__alpha : 5.0; XGB__colsample_bytree : 1.0; XGB__gamma : 0.1; XGB__max_depth : 3; XGB__min_child_weight : 0.0; XGB__random_state : 0; XGB__scale_pos_weight : 1.4000978172593856; XGB__subsample : 0.5	0.695	0.847
XGB	Not used	Not used	XGB__alpha : 9.099041682064584; XGB__colsample_bytree : 1.0; XGB__gamma : 0.1; XGB__max_depth : 3; XGB__min_child_weight : 0.0;	0.695	0.797

			XGB__random_state : 0; XGB__scale_pos_weight : 2.0; XGB__subsample : 0.5		
RF	SS	SMOTE	RF__class_weight : None; RF__criterion : log_loss; RF__max_depth : 1; RF__max_features : 1; RF__min_samples_leaf : 1; RF__n_estimators : 300; RF__random_state : 100	0.695	0.738
RF	SS	SMOTE	RF__class_weight : balanced_subsample; RF__criterion : log_loss; RF__max_depth : 1; RF__max_features : 1; RF__min_samples_leaf : 1; RF__n_estimators : 639; RF__random_state : 76	0.695	0.739
XGB	Not used	Not used	XGB__alpha : 5.0; XGB__colsample_bytree : 0.9151521394428219; XGB__gamma : 0.9460595040693235; XGB__max_depth : 1; XGB__min_child_weight : 0.0; XGB__random_state : 0; XGB__scale_pos_weight : 2.0; XGB__subsample : 0.637481203375762	0.695	0.763
RF	MM	ADASYN	RF__class_weight : balanced; RF__criterion : log_loss; RF__max_depth : 1; RF__max_features : 1; RF__min_samples_leaf : 1; RF__n_estimators : 1000; RF__random_state : 0	0.695	0.735
XGB	Not used	Not used	XGB__alpha : 8.957078766175758; XGB__colsample_bytree : 1.0; XGB__gamma : 0.1; XGB__max_depth : 3; XGB__min_child_weight : 0.0; XGB__random_state : 0; XGB__scale_pos_weight : 2.0; XGB__subsample : 0.5179020598467076	0.695	0.801
XGB	Not used	Not used	XGB__alpha : 5.0; XGB__colsample_bytree : 1.0; XGB__gamma : 0.1; XGB__max_depth : 1; XGB__min_child_weight : 0.0; XGB__random_state : 0; XGB__scale_pos_weight : 1.2784614768851763; XGB__subsample : 0.5	0.695	0.763

XGB	Not used	Not used	XGB__alpha : 9.010919349065759; XGB__colsample_bytree : 1.0; XGB__gamma : 0.1; XGB__max_depth : 2; XGB__min_child_weight : 0.0; XGB__random_state : 0; XGB__scale_pos_weight : 2.0; XGB__subsample : 0.5	0.694	0.783
RF	MM	ADASYN	RF__class_weight : balanced; RF__criterion : log_loss; RF__max_depth : 1; RF__max_features : 1; RF__min_samples_leaf : 1; RF__n_estimators : 300; RF__random_state : 0	0.694	0.734
XGB	Not used	Not used	XGB__alpha : 8.092701485401358; XGB__colsample_bytree : 1.0; XGB__gamma : 0.1; XGB__max_depth : 2; XGB__min_child_weight : 0.0; XGB__random_state : 0; XGB__scale_pos_weight : 2.0; XGB__subsample : 0.6844148110857339	0.694	0.800
NN	SS	ADASYN	NN__activation : identity; NN__alpha : 10.0; NN__hidden_layer_sizes : 50; NN__max_iter : 50; NN__random_state : 0; NN__solver : adam	0.694	0.731
RF	Not used	Not used	RF__class_weight : balanced; RF__criterion : gini; RF__max_depth : 2; RF__max_features : 1; RF__min_samples_leaf : 6; RF__n_estimators : 1000; RF__random_state : 100	0.694	0.771
XGB	MM	ROS	XGB__alpha : 5.0; XGB__colsample_bytree : 0.5; XGB__gamma : 0.1; XGB__max_depth : 1; XGB__min_child_weight : 4.880214389032; XGB__random_state : 0; XGB__scale_pos_weight : 1.586542707126661; XGB__subsample : 0.7	0.694	0.788
RF	SS	ROS	RF__class_weight : None; RF__criterion : gini; RF__max_depth : 2; RF__max_features : 1; RF__min_samples_leaf : 7; RF__n_estimators : 1000; RF__random_state : 100	0.694	0.771

XGB	Not used	Not used	XGB__alpha : 6.912497227354138; XGB__colsample_bytree : 1.0; XGB__gamma : 0.1; XGB__max_depth : 1; XGB__min_child_weight : 0.0; XGB__random_state : 0; XGB__scale_pos_weight : 1.402302773432067; XGB__subsample : 0.5	0.694	0.750
RF	SS	SMOTE	RF__class_weight : balanced_subsample; RF__criterion : log_loss; RF__max_depth : 2; RF__max_features : 1; RF__min_samples_leaf : 1; RF__n_estimators : 1000; RF__random_state : 65	0.694	0.755
RF	MM	ADASYN	RF__class_weight : balanced; RF__criterion : log_loss; RF__max_depth : 1; RF__max_features : 1; RF__min_samples_leaf : 1; RF__n_estimators : 665; RF__random_state : 0	0.694	0.734
XGB	Not used	Not used	XGB__alpha : 8.679351706688038; XGB__colsample_bytree : 1.0; XGB__gamma : 0.1; XGB__max_depth : 3; XGB__min_child_weight : 0.0; XGB__random_state : 0; XGB__scale_pos_weight : 2.0; XGB__subsample : 0.7	0.694	0.811
RF	MM	ADASYN	RF__class_weight : None; RF__criterion : log_loss; RF__max_depth : 1; RF__max_features : 1; RF__min_samples_leaf : 20; RF__n_estimators : 300; RF__random_state : 0	0.694	0.733
XGB	MM	ROS	XGB__alpha : 5.0; XGB__colsample_bytree : 0.5; XGB__gamma : 0.1; XGB__max_depth : 1; XGB__min_child_weight : 0.0; XGB__random_state : 0; XGB__scale_pos_weight : 2.0; XGB__subsample : 0.7	0.694	0.793
XGB	SS	ROS	XGB__alpha : 5.0; XGB__colsample_bytree : 0.5; XGB__gamma : 0.1; XGB__max_depth : 1; XGB__min_child_weight : 0.0; XGB__random_state :	0.694	0.793

			0; XGB__scale_pos_weight : 2.0; XGB__subsample : 0.7		
NN	SS	ADASYN	NN__activation : logistic; NN__alpha : 0.0001; NN__hidden_layer_sizes : 50; NN__max_iter : 500; NN__random_state : 0; NN__solver : adam	0.694	0.731
RF	MM	SMOTE	RF__class_weight : None; RF__criterion : gini; RF__max_depth : 1; RF__max_features : 1; RF__min_samples_leaf : 1; RF__n_estimators : 300; RF__random_state : 0	0.694	0.736
XGB	Not used	Not used	XGB__alpha : 8.495224571374575; XGB__colsample_bytree : 1.0; XGB__gamma : 0.1; XGB__max_depth : 3; XGB__min_child_weight : 0.0; XGB__random_state : 0; XGB__scale_pos_weight : 2.0; XGB__subsample : 0.6255803647450987	0.693	0.812
NN	SS	ADASYN	NN__activation : logistic; NN__alpha : 3.9229485139656597; NN__hidden_layer_sizes : 5; NN__max_iter : 245; NN__random_state : 0; NN__solver : adam	0.693	0.711
RF	MM	ADASYN	RF__class_weight : balanced; RF__criterion : log_loss; RF__max_depth : 1; RF__max_features : 1; RF__min_samples_leaf : 1; RF__n_estimators : 507; RF__random_state : 16	0.693	0.736
XGB	Not used	Not used	XGB__alpha : 7.272337795761228; XGB__colsample_bytree : 1.0; XGB__gamma : 0.1; XGB__max_depth : 3; XGB__min_child_weight : 0.0; XGB__random_state : 0; XGB__scale_pos_weight : 1.6314346406422557; XGB__subsample : 0.7	0.693	0.816
XGB	Not used	Not used	XGB__alpha : 5.0; XGB__colsample_bytree : 0.9916484327040396; XGB__gamma : 0.1; XGB__max_depth : 1; XGB__min_child_weight : 0.0;	0.693	0.760

			XGB__random_state : 0; XGB__scale_pos_weight : 1.1125583133014976; XGB__subsample : 0.7		
XGB	Not used	Not used	XGB__alpha : 7.207669982860329; XGB__colsample_bytree : 1.0; XGB__gamma : 0.1; XGB__max_depth : 3; XGB__min_child_weight : 0.0; XGB__random_state : 0; XGB__scale_pos_weight : 0.9427346130779876; XGB__subsample : 0.5	0.693	0.751
XGB	MM	ROS	XGB__alpha : 5.0; XGB__colsample_bytree : 0.8235208189215857; XGB__gamma : 0.1; XGB__max_depth : 1; XGB__min_child_weight : 0.0; XGB__random_state : 0; XGB__scale_pos_weight : 2.0; XGB__subsample : 0.5475153456705577	0.692	0.793
XGB	SS	ROS	XGB__alpha : 5.0; XGB__colsample_bytree : 0.8235208189215857; XGB__gamma : 0.1; XGB__max_depth : 1; XGB__min_child_weight : 0.0; XGB__random_state : 0; XGB__scale_pos_weight : 2.0; XGB__subsample : 0.5475153456705577	0.692	0.793
RF	SS	ROS	RF__class_weight : balanced; RF__criterion : log_loss; RF__max_depth : 3; RF__max_features : 1; RF__min_samples_leaf : 11; RF__n_estimators : 526; RF__random_state : 96	0.692	0.790
NN	SS	ADASYN	NN__activation : logistic; NN__alpha : 0.8035852444950408; NN__hidden_layer_sizes : 50; NN__max_iter : 500; NN__random_state : 0; NN__solver : adam	0.692	0.732
XGB	MM	ROS	XGB__alpha : 5.0; XGB__colsample_bytree : 1.0; XGB__gamma : 0.1; XGB__max_depth : 1; XGB__min_child_weight : 0.0; XGB__random_state : 0; XGB__scale_pos_weight : 1.625882542094309; XGB__subsample : 0.5	0.692	0.794

RF	SS	ROS	RF__class_weight : balanced_subsample; RF__criterion : log_loss; RF__max_depth : 3; RF__max_features : 1; RF__min_samples_leaf : 1; RF__n_estimators : 444; RF__random_state : 49	0.692	0.798
NN	SS	ADASYN	NN__activation : tanh; NN__alpha : 2.6096146808538574; NN__hidden_layer_sizes : 8; NN__max_iter : 478; NN__random_state : 0; NN__solver : adam	0.691	0.741
RF	MM	ADASYN	RF__class_weight : balanced; RF__criterion : log_loss; RF__max_depth : 1; RF__max_features : 1; RF__min_samples_leaf : 20; RF__n_estimators : 1000; RF__random_state : 0	0.691	0.733
RF	MM	ADASYN	RF__class_weight : balanced; RF__criterion : log_loss; RF__max_depth : 1; RF__max_features : 1; RF__min_samples_leaf : 20; RF__n_estimators : 1000; RF__random_state : 66	0.691	0.734
RF	SS	ADASYN	RF__class_weight : None; RF__criterion : log_loss; RF__max_depth : 2; RF__max_features : 1; RF__min_samples_leaf : 1; RF__n_estimators : 948; RF__random_state : 19	0.691	0.751
RF	SS	ADASYN	RF__class_weight : balanced; RF__criterion : log_loss; RF__max_depth : 2; RF__max_features : 1; RF__min_samples_leaf : 1; RF__n_estimators : 318; RF__random_state : 4	0.691	0.749
RF	MM	ADASYN	RF__class_weight : None; RF__criterion : log_loss; RF__max_depth : 1; RF__max_features : 1; RF__min_samples_leaf : 20; RF__n_estimators : 1000; RF__random_state : 0	0.691	0.733
XGB	MM	ROS	XGB__alpha : 6.736731598955543; XGB__colsample_bytree : 1.0; XGB__gamma : 0.1; XGB__max_depth : 1; XGB__min_child_weight : 0.0;	0.690	0.784

			XGB__random_state : 0; XGB__scale_pos_weight : 2.0; XGB__subsample : 0.5683523934852948		
RF	MM	SMOTE	RF__class_weight : balanced; RF__criterion : log_loss; RF__max_depth : 2; RF__max_features : 1; RF__min_samples_leaf : 20; RF__n_estimators : 300; RF__random_state : 0	0.690	0.747
XGB	MM	ROS	XGB__alpha : 5.0; XGB__colsample_bytree : 1.0; XGB__gamma : 0.1; XGB__max_depth : 1; XGB__min_child_weight : 0.0; XGB__random_state : 0; XGB__scale_pos_weight : 1.2466252209930042; XGB__subsample : 0.6572627607827732	0.689	0.793
RF	SS	ADASYN	RF__class_weight : balanced; RF__criterion : log_loss; RF__max_depth : 2; RF__max_features : 1; RF__min_samples_leaf : 20; RF__n_estimators : 786; RF__random_state : 96	0.688	0.745
RF	Not used	Not used	RF__class_weight : balanced_subsample; RF__criterion : log_loss; RF__max_depth : 5; RF__max_features : 1; RF__min_samples_leaf : 19; RF__n_estimators : 989; RF__random_state : 51	0.688	0.778
XGB	SS	ROS	XGB__alpha : 5.0; XGB__colsample_bytree : 1.0; XGB__gamma : 0.1; XGB__max_depth : 1; XGB__min_child_weight : 2.6508430705940436; XGB__random_state : 0; XGB__scale_pos_weight : 1.7600297028732244; XGB__subsample : 0.6639870852284843	0.688	0.801
XGB	SS	ROS	XGB__alpha : 5.0; XGB__colsample_bytree : 1.0; XGB__gamma : 1.7708138324740335; XGB__max_depth : 1; XGB__min_child_weight : 6.129595945684825; XGB__random_state : 0; XGB__scale_pos_weight : 2.0; XGB__subsample : 0.6861739509061591	0.688	0.782

NN	SS	ADASYN	NN__activation : relu; NN__alpha : 3.9814946105106603; NN__hidden_layer_sizes : 15; NN__max_iter : 427; NN__random_state : 0; NN__solver : adam	0.687	0.759
RF	Not used	Not used	RF__class_weight : None; RF__criterion : log_loss; RF__max_depth : 3; RF__max_features : 1; RF__min_samples_leaf : 20; RF__n_estimators : 498; RF__random_state : 59	0.687	0.768
NN	SS	ADASYN	NN__activation : identity; NN__alpha : 10.0; NN__hidden_layer_sizes : 50; NN__max_iter : 500; NN__random_state : 0; NN__solver : lbfgs	0.687	0.731
NN	SS	ADASYN	NN__activation : tanh; NN__alpha : 10.0; NN__hidden_layer_sizes : 3; NN__max_iter : 500; NN__random_state : 0; NN__solver : lbfgs	0.687	0.750
NN	SS	ADASYN	NN__activation : identity; NN__alpha : 10.0; NN__hidden_layer_sizes : 3; NN__max_iter : 50; NN__random_state : 0; NN__solver : lbfgs	0.687	0.731
XGB	MM	ROS	XGB__alpha : 10.196467921170566; XGB__colsample_bytree : 0.9886694173809806; XGB__gamma : 0.1; XGB__max_depth : 1; XGB__min_child_weight : 2.1234978311208788; XGB__random_state : 0; XGB__scale_pos_weight : 1.51260621923905; XGB__subsample : 0.5764970291109225	0.686	0.752
XGB	SS	ROS	XGB__alpha : 10.196467921170566; XGB__colsample_bytree : 0.9886694173809806; XGB__gamma : 0.1; XGB__max_depth : 1; XGB__min_child_weight : 2.1234978311208788; XGB__random_state : 0; XGB__scale_pos_weight : 1.51260621923905; XGB__subsample : 0.5764970291109225	0.686	0.752

XGB	SS	ROS	XGB__alpha : 5.0; XGB__colsample_bytree : 0.5; XGB__gamma : 1.8126757958675883; XGB__max_depth : 3; XGB__min_child_weight : 33.456795387334914; XGB__random_state : 0; XGB__scale_pos_weight : 2.0; XGB__subsample : 0.7	0.686	0.735
XGB	SS	ROS	XGB__alpha : 5.0; XGB__colsample_bytree : 0.5; XGB__gamma : 0.1; XGB__max_depth : 1; XGB__min_child_weight : 0.0; XGB__random_state : 0; XGB__scale_pos_weight : 1.357126240220297; XGB__subsample : 0.5	0.685	0.779
RF	SS	ROS	RF__class_weight : None; RF__criterion : gini; RF__max_depth : 3; RF__max_features : 1; RF__min_samples_leaf : 15; RF__n_estimators : 884; RF__random_state : 3	0.685	0.788
XGB	SS	ROS	XGB__alpha : 5.0; XGB__colsample_bytree : 1.0; XGB__gamma : 2.172424617631541; XGB__max_depth : 1; XGB__min_child_weight : 0.0; XGB__random_state : 0; XGB__scale_pos_weight : 2.0; XGB__subsample : 0.5	0.684	0.775
XGB	MM	ROS	XGB__alpha : 5.0; XGB__colsample_bytree : 0.506144547889209; XGB__gamma : 0.1; XGB__max_depth : 1; XGB__min_child_weight : 5.489585305776787; XGB__random_state : 0; XGB__scale_pos_weight : 0.9252207496952428; XGB__subsample : 0.6790194588564726	0.684	0.773
NN	SS	ADASYN	NN__activation : identity; NN__alpha : 0.007217166401724121; NN__hidden_layer_sizes : 50; NN__max_iter : 500; NN__random_state : 0; NN__solver : adam	0.683	0.729
XGB	MM	ROS	XGB__alpha : 5.0; XGB__colsample_bytree : 1.0; XGB__gamma : 0.1; XGB__max_depth : 2;	0.683	0.811

			XGB__min_child_weight : 0.0; XGB__random_state : 0; XGB__scale_pos_weight : 0.5; XGB__subsample : 0.5		
NN	SS	ADASYN	NN__activation : identity; NN__alpha : 0.0001; NN__hidden_layer_sizes : 50; NN__max_iter : 500; NN__random_state : 0; NN__solver : adam	0.683	0.729
XGB	MM	ROS	XGB__alpha : 5.0; XGB__colsample_bytree : 0.6137575243734916; XGB__gamma : 1.0480605164386632; XGB__max_depth : 1; XGB__min_child_weight : 0.0; XGB__random_state : 0; XGB__scale_pos_weight : 1.8795444638284153; XGB__subsample : 0.6574296775410922	0.682	0.782
XGB	SS	ROS	XGB__alpha : 5.0; XGB__colsample_bytree : 0.6137575243734916; XGB__gamma : 1.0480605164386632; XGB__max_depth : 1; XGB__min_child_weight : 0.0; XGB__random_state : 0; XGB__scale_pos_weight : 1.8795444638284153; XGB__subsample : 0.6574296775410922	0.682	0.782
NN	SS	ADASYN	NN__activation : identity; NN__alpha : 1.2278635248432284; NN__hidden_layer_sizes : 27; NN__max_iter : 165; NN__random_state : 0; NN__solver : adam	0.682	0.730
XGB	SS	ROS	XGB__alpha : 5.0; XGB__colsample_bytree : 0.5264851861390412; XGB__gamma : 0.1; XGB__max_depth : 3; XGB__min_child_weight : 0.0; XGB__random_state : 0; XGB__scale_pos_weight : 2.0; XGB__subsample : 0.548684309541424	0.682	0.883
NN	SS	ADASYN	NN__activation : identity; NN__alpha : 0.018123696732686986; NN__hidden_layer_sizes : 50; NN__max_iter : 500; NN__random_state : 0; NN__solver : lbfgs	0.682	0.730

XGB	MM	ROS	XGB__alpha : 5.0; XGB__colsample_bytree : 0.5; XGB__gamma : 0.1; XGB__max_depth : 1; XGB__min_child_weight : 19.290799090180425; XGB__random_state : 0; XGB__scale_pos_weight : 2.0; XGB__subsample : 0.7	0.681	0.766
XGB	MM	ROS	XGB__alpha : 5.0; XGB__colsample_bytree : 1.0; XGB__gamma : 0.1; XGB__max_depth : 1; XGB__min_child_weight : 0.0; XGB__random_state : 0; XGB__scale_pos_weight : 0.5; XGB__subsample : 0.5	0.681	0.757
XGB	SS	ROS	XGB__alpha : 5.0; XGB__colsample_bytree : 1.0; XGB__gamma : 0.1; XGB__max_depth : 1; XGB__min_child_weight : 0.0; XGB__random_state : 0; XGB__scale_pos_weight : 0.5; XGB__subsample : 0.5	0.681	0.757
XGB	MM	ROS	XGB__alpha : 5.0; XGB__colsample_bytree : 1.0; XGB__gamma : 1.8175464993510824; XGB__max_depth : 1; XGB__min_child_weight : 6.730562755006236; XGB__random_state : 0; XGB__scale_pos_weight : 2.0; XGB__subsample : 0.685323541161032	0.681	0.779
XGB	MM	ROS	XGB__alpha : 5.0; XGB__colsample_bytree : 1.0; XGB__gamma : 0.1; XGB__max_depth : 1; XGB__min_child_weight : 16.959304006574122; XGB__random_state : 0; XGB__scale_pos_weight : 1.3722225232484302; XGB__subsample : 0.5	0.681	0.757
RF	SS	ROS	RF__class_weight : balanced_subsample; RF__criterion : log_loss; RF__max_depth : 5; RF__max_features : 1; RF__min_samples_leaf : 13; RF__n_estimators : 816; RF__random_state : 0	0.681	0.821

XGB	MM	ROS	XGB__alpha : 5.0; XGB__colsample_bytree : 1.0; XGB__gamma : 0.1; XGB__max_depth : 1; XGB__min_child_weight : 12.346475001805546; XGB__random_state : 0; XGB__scale_pos_weight : 0.5; XGB__subsample : 0.5	0.681	0.736
RF	SS	ROS	RF__class_weight : None; RF__criterion : log_loss; RF__max_depth : 3; RF__max_features : 2; RF__min_samples_leaf : 18; RF__n_estimators : 301; RF__random_state : 8	0.680	0.792
XGB	SS	ROS	XGB__alpha : 5.0; XGB__colsample_bytree : 0.5; XGB__gamma : 1.0666493712918015; XGB__max_depth : 3; XGB__min_child_weight : 31.63515870320992; XGB__random_state : 0; XGB__scale_pos_weight : 2.0; XGB__subsample : 0.6598505460869761	0.680	0.737
XGB	MM	ROS	XGB__alpha : 5.0; XGB__colsample_bytree : 0.5264851861390412; XGB__gamma : 0.1; XGB__max_depth : 3; XGB__min_child_weight : 0.0; XGB__random_state : 0; XGB__scale_pos_weight : 2.0; XGB__subsample : 0.548684309541424	0.680	0.883
XGB	MM	ROS	XGB__alpha : 5.0; XGB__colsample_bytree : 0.5; XGB__gamma : 0.1; XGB__max_depth : 2; XGB__min_child_weight : 0.0; XGB__random_state : 0; XGB__scale_pos_weight : 0.5; XGB__subsample : 0.7	0.680	0.802
XGB	SS	ROS	XGB__alpha : 5.0; XGB__colsample_bytree : 0.5; XGB__gamma : 0.1; XGB__max_depth : 2; XGB__min_child_weight : 0.0; XGB__random_state : 0; XGB__scale_pos_weight : 0.5; XGB__subsample : 0.7	0.680	0.802

XGB	SS	ROS	XGB__alpha : 7.49790102877007; XGB__colsample_bytree : 0.5; XGB__gamma : 0.1; XGB__max_depth : 3; XGB__min_child_weight : 34.148554160929955; XGB__random_state : 0; XGB__scale_pos_weight : 2.0; XGB__subsample : 0.7	0.680	0.737
RF	SS	ROS	RF__class_weight : balanced_subsample; RF__criterion : gini; RF__max_depth : 5; RF__max_features : 1; RF__min_samples_leaf : 14; RF__n_estimators : 631; RF__random_state : 35	0.678	0.818
NN	SS	ADASYN	NN__activation : relu; NN__alpha : 4.975636992427353; NN__hidden_layer_sizes : 6; NN__max_iter : 135; NN__random_state : 0; NN__solver : adam	0.678	0.721
RF	SS	ADASYN	RF__class_weight : None; RF__criterion : gini; RF__max_depth : 2; RF__max_features : 1; RF__min_samples_leaf : 1; RF__n_estimators : 556; RF__random_state : 91	0.678	0.748
RF	SS	ROS	RF__class_weight : balanced_subsample; RF__criterion : log_loss; RF__max_depth : 1; RF__max_features : 2; RF__min_samples_leaf : 16; RF__n_estimators : 388; RF__random_state : 0	0.677	0.751
XGB	SS	ROS	XGB__alpha : 5.0; XGB__colsample_bytree : 0.5; XGB__gamma : 0.1; XGB__max_depth : 2; XGB__min_child_weight : 31.393426240369433; XGB__random_state : 0; XGB__scale_pos_weight : 2.0; XGB__subsample : 0.7	0.677	0.752
XGB	SS	ROS	XGB__alpha : 5.0; XGB__colsample_bytree : 0.5761956329403548; XGB__gamma : 2.5886922507410484; XGB__max_depth : 1; XGB__min_child_weight : 4.903091367772631;	0.676	0.767

			XGB__random_state : 0; XGB__scale_pos_weight : 2.0; XGB__subsample : 0.7		
RF	SS	ROS	RF__class_weight : None; RF__criterion : log_loss; RF__max_depth : 3; RF__max_features : 2; RF__min_samples_leaf : 19; RF__n_estimators : 316; RF__random_state : 99	0.676	0.793
XGB	MM	ROS	XGB__alpha : 5.0; XGB__colsample_bytree : 1.0; XGB__gamma : 0.1; XGB__max_depth : 3; XGB__min_child_weight : 0.0; XGB__random_state : 0; XGB__scale_pos_weight : 0.5; XGB__subsample : 0.7	0.675	0.854
XGB	SS	ROS	XGB__alpha : 5.0; XGB__colsample_bytree : 0.5; XGB__gamma : 2.141520841440125; XGB__max_depth : 3; XGB__min_child_weight : 28.081509474612222; XGB__random_state : 0; XGB__scale_pos_weight : 1.439334349509644; XGB__subsample : 0.7	0.674	0.736
RF	SS	ROS	RF__class_weight : balanced_subsample; RF__criterion : log_loss; RF__max_depth : 3; RF__max_features : 8; RF__min_samples_leaf : 3; RF__n_estimators : 319; RF__random_state : 57	0.673	0.803
XGB	SS	ROS	XGB__alpha : 5.0; XGB__colsample_bytree : 0.5; XGB__gamma : 1.7921422540006477; XGB__max_depth : 1; XGB__min_child_weight : 24.999870613602848; XGB__random_state : 0; XGB__scale_pos_weight : 1.3750951624475025; XGB__subsample : 0.7	0.673	0.736
XGB	MM	ROS	XGB__alpha : 5.0; XGB__colsample_bytree : 0.5; XGB__gamma : 0.1; XGB__max_depth : 1; XGB__min_child_weight : 11.636445258318036;	0.673	0.743

			XGB__random_state : 0; XGB__scale_pos_weight : 0.7371732942300812; XGB__subsample : 0.5		
RF	SS	ADASYN	RF__class_weight : balanced; RF__criterion : log_loss; RF__max_depth : 3; RF__max_features : 1; RF__min_samples_leaf : 20; RF__n_estimators : 785; RF__random_state : 3	0.672	0.756
RF	SS	ROS	RF__class_weight : balanced_subsample; RF__criterion : log_loss; RF__max_depth : 5; RF__max_features : 1; RF__min_samples_leaf : 10; RF__n_estimators : 419; RF__random_state : 95	0.672	0.834
XGB	SS	ROS	XGB__alpha : 5.0; XGB__colsample_bytree : 1.0; XGB__gamma : 0.1; XGB__max_depth : 1; XGB__min_child_weight : 6.017884701612123; XGB__random_state : 0; XGB__scale_pos_weight : 0.8526671439629193; XGB__subsample : 0.6652042584846335	0.672	0.774
XGB	MM	ROS	XGB__alpha : 5.0; XGB__colsample_bytree : 1.0; XGB__gamma : 0.1; XGB__max_depth : 2; XGB__min_child_weight : 14.511089713118544; XGB__random_state : 0; XGB__scale_pos_weight : 0.5; XGB__subsample : 0.5	0.672	0.729
XGB	MM	ROS	XGB__alpha : 5.0; XGB__colsample_bytree : 1.0; XGB__gamma : 0.1; XGB__max_depth : 1; XGB__min_child_weight : 22.67687517959803; XGB__random_state : 0; XGB__scale_pos_weight : 1.898323576719728; XGB__subsample : 0.6050331960216304	0.670	0.764
XGB	MM	ROS	XGB__alpha : 12.332686906518637; XGB__colsample_bytree : 1.0; XGB__gamma : 0.1; XGB__max_depth : 1; XGB__min_child_weight : 0.0;	0.670	0.751

			XGB__random_state : 0; XGB__scale_pos_weight : 2.0; XGB__subsample : 0.7		
XGB	MM	ROS	XGB__alpha : 5.0; XGB__colsample_bytree : 0.6538029761263292; XGB__gamma : 2.8505742334704736; XGB__max_depth : 2; XGB__min_child_weight : 1.2646515564067446; XGB__random_state : 0; XGB__scale_pos_weight : 2.0; XGB__subsample : 0.5678974858815471	0.670	0.803
XGB	SS	ROS	XGB__alpha : 5.0; XGB__colsample_bytree : 0.6538029761263292; XGB__gamma : 2.8505742334704736; XGB__max_depth : 2; XGB__min_child_weight : 1.2646515564067446; XGB__random_state : 0; XGB__scale_pos_weight : 2.0; XGB__subsample : 0.5678974858815471	0.670	0.803
XGB	MM	ROS	XGB__alpha : 5.0; XGB__colsample_bytree : 0.5; XGB__gamma : 0.1; XGB__max_depth : 1; XGB__min_child_weight : 23.0607397237513; XGB__random_state : 0; XGB__scale_pos_weight : 1.5561233626845743; XGB__subsample : 0.7	0.670	0.755
RF	SS	ROS	RF__class_weight : balanced; RF__criterion : log_loss; RF__max_depth : 1; RF__max_features : 2; RF__min_samples_leaf : 3; RF__n_estimators : 967; RF__random_state : 100	0.670	0.750
XGB	MM	ROS	XGB__alpha : 5.0; XGB__colsample_bytree : 0.9119313619721247; XGB__gamma : 5.0; XGB__max_depth : 1; XGB__min_child_weight : 0.0; XGB__random_state : 0; XGB__scale_pos_weight : 1.5857510275143625; XGB__subsample : 0.524570237808505	0.670	0.745
XGB	SS	ROS	XGB__alpha : 5.0; XGB__colsample_bytree : 0.9119313619721247; XGB__gamma : 5.0;	0.670	0.745

			XGB__max_depth : 1; XGB__min_child_weight : 0.0; XGB__random_state : 0; XGB__scale_pos_weight : 1.5857510275143625; XGB__subsample : 0.524570237808505		
XGB	MM	ROS	XGB__alpha : 5.0; XGB__colsample_bytree : 1.0; XGB__gamma : 0.4369554525516144; XGB__max_depth : 1; XGB__min_child_weight : 7.926369719218964; XGB__random_state : 0; XGB__scale_pos_weight : 2.0; XGB__subsample : 0.5639962547360536	0.670	0.784
XGB	SS	ROS	XGB__alpha : 5.0; XGB__colsample_bytree : 1.0; XGB__gamma : 0.4369554525516144; XGB__max_depth : 1; XGB__min_child_weight : 7.926369719218964; XGB__random_state : 0; XGB__scale_pos_weight : 2.0; XGB__subsample : 0.5639962547360536	0.670	0.784
RF	MM	SMOTE	RF__class_weight : balanced_subsample; RF__criterion : log_loss; RF__max_depth : 3; RF__max_features : 1; RF__min_samples_leaf : 3; RF__n_estimators : 996; RF__random_state : 70	0.670	0.773
XGB	SS	ROS	XGB__alpha : 5.0; XGB__colsample_bytree : 0.5; XGB__gamma : 1.226685331044141; XGB__max_depth : 3; XGB__min_child_weight : 22.058917704386314; XGB__random_state : 0; XGB__scale_pos_weight : 0.911982562205536; XGB__subsample : 0.7	0.669	0.734
XGB	SS	ROS	XGB__alpha : 5.0; XGB__colsample_bytree : 1.0; XGB__gamma : 3.92704707848348; XGB__max_depth : 1; XGB__min_child_weight : 0.0; XGB__random_state : 0; XGB__scale_pos_weight : 2.0; XGB__subsample : 0.602559348046943	0.669	0.758

XGB	MM	ROS	XGB__alpha : 5.0; XGB__colsample_bytree : 1.0; XGB__gamma : 1.1973981523302588; XGB__max_depth : 1; XGB__min_child_weight : 9.694469483369145; XGB__random_state : 0; XGB__scale_pos_weight : 1.1589710443262158; XGB__subsample : 0.658457542891933	0.669	0.759
XGB	SS	ROS	XGB__alpha : 5.0; XGB__colsample_bytree : 0.5; XGB__gamma : 0.1; XGB__max_depth : 3; XGB__min_child_weight : 11.923206352594525; XGB__random_state : 0; XGB__scale_pos_weight : 0.5; XGB__subsample : 0.5	0.669	0.736
XGB	SS	ROS	XGB__alpha : 5.0; XGB__colsample_bytree : 1.0; XGB__gamma : 1.2188283259997827; XGB__max_depth : 1; XGB__min_child_weight : 0.0; XGB__random_state : 0; XGB__scale_pos_weight : 0.6629510777554158; XGB__subsample : 0.691576002874868	0.668	0.752
XGB	MM	ROS	XGB__alpha : 5.0; XGB__colsample_bytree : 0.5; XGB__gamma : 0.1; XGB__max_depth : 3; XGB__min_child_weight : 20.278432134750943; XGB__random_state : 0; XGB__scale_pos_weight : 1.3289463000278767; XGB__subsample : 0.6968010272900145	0.668	0.779
XGB	SS	ROS	XGB__alpha : 13.346965365279308; XGB__colsample_bytree : 0.6968270208547013; XGB__gamma : 0.1; XGB__max_depth : 2; XGB__min_child_weight : 1.7044619025655332; XGB__random_state : 0; XGB__scale_pos_weight : 1.4121959210449408; XGB__subsample : 0.7	0.667	0.776
XGB	SS	ROS	XGB__alpha : 9.647185441645753; XGB__colsample_bytree : 0.7465821334427631;	0.667	0.798

			XGB__gamma : 0.4601545702046056; XGB__max_depth : 2; XGB__min_child_weight : 0.0; XGB__random_state : 0; XGB__scale_pos_weight : 1.6683458493066796; XGB__subsample : 0.5570264201746168		
XGB	SS	ROS	XGB__alpha : 5.0; XGB__colsample_bytree : 0.5; XGB__gamma : 1.509591831962534; XGB__max_depth : 1; XGB__min_child_weight : 23.544745552216703; XGB__random_state : 0; XGB__scale_pos_weight : 2.0; XGB__subsample : 0.6730909602391604	0.667	0.749
XGB	SS	ROS	XGB__alpha : 10.452985867505967; XGB__colsample_bytree : 0.8855336186336551; XGB__gamma : 0.1; XGB__max_depth : 1; XGB__min_child_weight : 11.750196922419978; XGB__random_state : 0; XGB__scale_pos_weight : 1.8323867141985335; XGB__subsample : 0.7	0.667	0.748
RF	SS	ADASYN	RF__class_weight : balanced_subsample; RF__criterion : log_loss; RF__max_depth : 3; RF__max_features : 1; RF__min_samples_leaf : 1; RF__n_estimators : 940; RF__random_state : 100	0.667	0.773
RF	MM	SMOTE	RF__class_weight : balanced_subsample; RF__criterion : gini; RF__max_depth : 2; RF__max_features : 1; RF__min_samples_leaf : 16; RF__n_estimators : 301; RF__random_state : 64	0.664	0.747
XGB	SS	SMOTE	XGB__alpha : 5.0; XGB__colsample_bytree : 0.5; XGB__gamma : 0.1; XGB__max_depth : 3; XGB__min_child_weight : 35.003002308269586; XGB__random_state : 0; XGB__scale_pos_weight : 1.8013071864082288; XGB__subsample : 0.5	0.661	0.700

XGB	MM	ADASYN	XGB__alpha : 5.0; XGB__colsample_bytree : 0.5104339708965451; XGB__gamma : 0.1; XGB__max_depth : 1; XGB__min_child_weight : 27.817712445384885; XGB__random_state : 0; XGB__scale_pos_weight : 1.504939050328973; XGB__subsample : 0.5	0.661	0.712
RF	MM	SMOTE	RF__class_weight : balanced_subsample; RF__criterion : log_loss; RF__max_depth : 1; RF__max_features : 2; RF__min_samples_leaf : 18; RF__n_estimators : 1000; RF__random_state : 9	0.660	0.727
XGB	MM	SMOTE	XGB__alpha : 5.0; XGB__colsample_bytree : 0.6762765744583707; XGB__gamma : 0.1; XGB__max_depth : 1; XGB__min_child_weight : 44.87118231447129; XGB__random_state : 0; XGB__scale_pos_weight : 2.0; XGB__subsample : 0.5	0.659	0.668
XGB	SS	ADASYN	XGB__alpha : 5.0; XGB__colsample_bytree : 0.5649231703666495; XGB__gamma : 0.1; XGB__max_depth : 1; XGB__min_child_weight : 44.073078378318876; XGB__random_state : 0; XGB__scale_pos_weight : 2.0; XGB__subsample : 0.5	0.659	0.676
RF	SS	ADASYN	RF__class_weight : balanced_subsample; RF__criterion : log_loss; RF__max_depth : 2; RF__max_features : 3; RF__min_samples_leaf : 11; RF__n_estimators : 470; RF__random_state : 46	0.659	0.731
XGB	SS	SMOTE	XGB__alpha : 5.0; XGB__colsample_bytree : 0.9789335516015518; XGB__gamma : 0.1; XGB__max_depth : 3; XGB__min_child_weight : 39.859630761960524; XGB__random_state : 0; XGB__scale_pos_weight : 2.0; XGB__subsample : 0.545659146018476	0.658	0.701

XGB	MM	ADASYN	XGB__alpha : 5.0; XGB__colsample_bytree : 0.9734046446248774; XGB__gamma : 0.1; XGB__max_depth : 3; XGB__min_child_weight : 23.547388620693035; XGB__random_state : 0; XGB__scale_pos_weight : 1.085450149082817; XGB__subsample : 0.5	0.657	0.713
XGB	SS	SMOTE	XGB__alpha : 5.0; XGB__colsample_bytree : 0.9341088008697684; XGB__gamma : 0.1; XGB__max_depth : 3; XGB__min_child_weight : 36.152884553633484; XGB__random_state : 0; XGB__scale_pos_weight : 1.6713008400273912; XGB__subsample : 0.5668252749427178	0.657	0.708
XGB	SS	SMOTE	XGB__alpha : 5.0; XGB__colsample_bytree : 1.0; XGB__gamma : 0.1; XGB__max_depth : 3; XGB__min_child_weight : 32.21546743766196; XGB__random_state : 0; XGB__scale_pos_weight : 2.0; XGB__subsample : 0.5	0.657	0.714
XGB	MM	ADASYN	XGB__alpha : 5.0; XGB__colsample_bytree : 0.9990891826903692; XGB__gamma : 0.1; XGB__max_depth : 1; XGB__min_child_weight : 28.246617231301364; XGB__random_state : 0; XGB__scale_pos_weight : 1.500494338720833; XGB__subsample : 0.5	0.657	0.712
XGB	SS	ADASYN	XGB__alpha : 5.0; XGB__colsample_bytree : 0.5; XGB__gamma : 0.1; XGB__max_depth : 3; XGB__min_child_weight : 42.128635004400024; XGB__random_state : 0; XGB__scale_pos_weight : 2.0; XGB__subsample : 0.5503763311789271	0.656	0.692
XGB	MM	SMOTE	XGB__alpha : 5.0; XGB__colsample_bytree : 0.6601994598267135; XGB__gamma : 0.1; XGB__max_depth : 2; XGB__min_child_weight :	0.656	0.721

			35.92187611818295; XGB__random_state : 0; XGB__scale_pos_weight : 2.0; XGB__subsample : 0.646670269530334		
XGB	SS	ADASYN	XGB__alpha : 5.0; XGB__colsample_bytree : 0.5; XGB__gamma : 3.8043174298483264; XGB__max_depth : 2; XGB__min_child_weight : 42.07988040296253; XGB__random_state : 0; XGB__scale_pos_weight : 2.0; XGB__subsample : 0.5590780031877133	0.656	0.693
XGB	SS	SMOTE	XGB__alpha : 10.256439322046969; XGB__colsample_bytree : 0.6222933045266974; XGB__gamma : 0.1; XGB__max_depth : 3; XGB__min_child_weight : 34.64134775717527; XGB__random_state : 0; XGB__scale_pos_weight : 2.0; XGB__subsample : 0.7	0.656	0.723
XGB	SS	SMOTE	XGB__alpha : 5.0; XGB__colsample_bytree : 0.5341509310357867; XGB__gamma : 0.1; XGB__max_depth : 2; XGB__min_child_weight : 35.85043621647163; XGB__random_state : 0; XGB__scale_pos_weight : 1.7891482660132831; XGB__subsample : 0.5	0.656	0.696
XGB	SS	SMOTE	XGB__alpha : 5.0; XGB__colsample_bytree : 0.8146986602557749; XGB__gamma : 0.1; XGB__max_depth : 2; XGB__min_child_weight : 34.78255126582806; XGB__random_state : 0; XGB__scale_pos_weight : 1.6000016569355706; XGB__subsample : 0.5	0.655	0.692
XGB	MM	ADASYN	XGB__alpha : 5.0; XGB__colsample_bytree : 0.947204554519848; XGB__gamma : 0.1; XGB__max_depth : 2; XGB__min_child_weight : 32.51610515339778; XGB__random_state : 0;	0.655	0.712

			XGB__scale_pos_weight : 1.8711145177438429; XGB__subsample : 0.5202948901555388		
XGB	MM	SMOTE	XGB__alpha : 5.0; XGB__colsample_bytree : 1.0; XGB__gamma : 0.1; XGB__max_depth : 3; XGB__min_child_weight : 28.108455796840826; XGB__random_state : 0; XGB__scale_pos_weight : 2.0; XGB__subsample : 0.6385273222839287	0.654	0.738
XGB	SS	SMOTE	XGB__alpha : 5.0; XGB__colsample_bytree : 1.0; XGB__gamma : 0.1; XGB__max_depth : 2; XGB__min_child_weight : 34.87601703810017; XGB__random_state : 0; XGB__scale_pos_weight : 2.0; XGB__subsample : 0.5	0.654	0.708
XGB	SS	ADASYN	XGB__alpha : 5.0; XGB__colsample_bytree : 0.5; XGB__gamma : 0.1; XGB__max_depth : 1; XGB__min_child_weight : 42.07697318443594; XGB__random_state : 0; XGB__scale_pos_weight : 2.0; XGB__subsample : 0.5185451502535441	0.654	0.690
RF	MM	SMOTE	RF__class_weight : None; RF__criterion : log_loss; RF__max_depth : 5; RF__max_features : 1; RF__min_samples_leaf : 20; RF__n_estimators : 709; RF__random_state : 33	0.654	0.775
XGB	SS	ADASYN	XGB__alpha : 5.0; XGB__colsample_bytree : 0.5; XGB__gamma : 5.0; XGB__max_depth : 3; XGB__min_child_weight : 36.53564295051981; XGB__random_state : 0; XGB__scale_pos_weight : 2.0; XGB__subsample : 0.5	0.653	0.697
XGB	MM	SMOTE	XGB__alpha : 5.0; XGB__colsample_bytree : 0.839380365173769; XGB__gamma : 0.1; XGB__max_depth : 3; XGB__min_child_weight : 38.58841595099147; XGB__random_state : 0;	0.653	0.719

			XGB__scale_pos_weight : 2.0; XGB__subsample : 0.6460343647575388		
XGB	MM	ADASYN	XGB__alpha : 5.0; XGB__colsample_bytree : 0.5; XGB__gamma : 2.270655376949256; XGB__max_depth : 1; XGB__min_child_weight : 32.08731823757594; XGB__random_state : 0; XGB__scale_pos_weight : 1.542050586715464; XGB__subsample : 0.5	0.653	0.695
XGB	SS	ADASYN	XGB__alpha : 5.0; XGB__colsample_bytree : 0.5; XGB__gamma : 0.1; XGB__max_depth : 2; XGB__min_child_weight : 50.0; XGB__random_state : 0; XGB__scale_pos_weight : 1.674723178234668; XGB__subsample : 0.7	0.652	0.691
XGB	MM	SMOTE	XGB__alpha : 5.0; XGB__colsample_bytree : 0.5; XGB__gamma : 0.1; XGB__max_depth : 3; XGB__min_child_weight : 36.63597927402169; XGB__random_state : 0; XGB__scale_pos_weight : 2.0; XGB__subsample : 0.6353302095361173	0.652	0.717
XGB	SS	SMOTE	XGB__alpha : 5.0; XGB__colsample_bytree : 0.5; XGB__gamma : 5.0; XGB__max_depth : 3; XGB__min_child_weight : 33.288951101423834; XGB__random_state : 0; XGB__scale_pos_weight : 2.0; XGB__subsample : 0.5	0.652	0.704
XGB	MM	SMOTE	XGB__alpha : 5.0; XGB__colsample_bytree : 1.0; XGB__gamma : 0.1; XGB__max_depth : 1; XGB__min_child_weight : 36.6278511511537; XGB__random_state : 0; XGB__scale_pos_weight : 2.0; XGB__subsample : 0.6562608319892366	0.652	0.723
RF	MM	SMOTE	RF__class_weight : balanced; RF__criterion : gini; RF__max_depth : 5; RF__max_features : 1;	0.652	0.773

			RF__min_samples_leaf : 20; RF__n_estimators : 1000; RF__random_state : 0		
RF	SS	ADASYN	RF__class_weight : balanced; RF__criterion : log_loss; RF__max_depth : 5; RF__max_features : 1; RF__min_samples_leaf : 17; RF__n_estimators : 746; RF__random_state : 1	0.652	0.779
XGB	SS	SMOTE	XGB__alpha : 5.0; XGB__colsample_bytree : 0.5; XGB__gamma : 0.1; XGB__max_depth : 3; XGB__min_child_weight : 36.20978223446801; XGB__random_state : 0; XGB__scale_pos_weight : 2.0; XGB__subsample : 0.5252290291005896	0.652	0.707
XGB	MM	ADASYN	XGB__alpha : 5.0; XGB__colsample_bytree : 1.0; XGB__gamma : 0.1; XGB__max_depth : 1; XGB__min_child_weight : 30.723529105039383; XGB__random_state : 0; XGB__scale_pos_weight : 1.3588467096218786; XGB__subsample : 0.5	0.652	0.702
XGB	MM	SMOTE	XGB__alpha : 8.535645016770244; XGB__colsample_bytree : 0.564658961611182; XGB__gamma : 0.1; XGB__max_depth : 3; XGB__min_child_weight : 49.27294225655442; XGB__random_state : 0; XGB__scale_pos_weight : 2.0; XGB__subsample : 0.6120357168191886	0.651	0.686
XGB	MM	SMOTE	XGB__alpha : 5.0; XGB__colsample_bytree : 0.5; XGB__gamma : 0.1; XGB__max_depth : 3; XGB__min_child_weight : 38.31684706500907; XGB__random_state : 0; XGB__scale_pos_weight : 2.0; XGB__subsample : 0.66195014280539	0.651	0.717
XGB	SS	SMOTE	XGB__alpha : 5.0; XGB__colsample_bytree : 1.0; XGB__gamma : 0.1; XGB__max_depth : 2; XGB__min_child_weight : 35.66649193306906;	0.651	0.714

			XGB__random_state : 0; XGB__scale_pos_weight : 2.0; XGB__subsample : 0.5644738656710776		
XGB	SS	SMOTE	XGB__alpha : 5.0; XGB__colsample_bytree : 0.92301881636697; XGB__gamma : 0.1; XGB__max_depth : 2; XGB__min_child_weight : 31.461190726467965; XGB__random_state : 0; XGB__scale_pos_weight : 1.798097337999943; XGB__subsample : 0.5724442082071851	0.651	0.718
XGB	SS	SMOTE	XGB__alpha : 9.947967265873556; XGB__colsample_bytree : 0.5; XGB__gamma : 0.1; XGB__max_depth : 1; XGB__min_child_weight : 32.005004962451814; XGB__random_state : 0; XGB__scale_pos_weight : 1.6051579466102508; XGB__subsample : 0.7	0.651	0.720
XGB	SS	ADASYN	XGB__alpha : 5.0; XGB__colsample_bytree : 0.5; XGB__gamma : 0.1; XGB__max_depth : 1; XGB__min_child_weight : 25.489236973082285; XGB__random_state : 0; XGB__scale_pos_weight : 1.1558347277987266; XGB__subsample : 0.5	0.650	0.712
XGB	SS	SMOTE	XGB__alpha : 5.0; XGB__colsample_bytree : 0.5; XGB__gamma : 0.1; XGB__max_depth : 3; XGB__min_child_weight : 33.955566325242835; XGB__random_state : 0; XGB__scale_pos_weight : 2.0; XGB__subsample : 0.7	0.650	0.730
XGB	MM	ADASYN	XGB__alpha : 5.0; XGB__colsample_bytree : 0.8111507512716805; XGB__gamma : 0.1; XGB__max_depth : 1; XGB__min_child_weight : 33.55974256592578; XGB__random_state : 0; XGB__scale_pos_weight : 1.4106261852786681; XGB__subsample : 0.6136452727589473	0.650	0.713

XGB	SS	SMOTE	XGB__alpha : 17.535957647485876; XGB__colsample_bytree : 0.805407502146489; XGB__gamma : 5.0; XGB__max_depth : 1; XGB__min_child_weight : 0.0; XGB__random_state : 0; XGB__scale_pos_weight : 2.0; XGB__subsample : 0.5303018670951418	0.650	0.703
XGB	MM	SMOTE	XGB__alpha : 8.856391040374422; XGB__colsample_bytree : 0.9483816792602746; XGB__gamma : 0.1; XGB__max_depth : 2; XGB__min_child_weight : 38.47234786836239; XGB__random_state : 0; XGB__scale_pos_weight : 2.0; XGB__subsample : 0.7	0.650	0.715
XGB	MM	SMOTE	XGB__alpha : 5.0; XGB__colsample_bytree : 0.5; XGB__gamma : 0.1; XGB__max_depth : 3; XGB__min_child_weight : 36.3648510105573; XGB__random_state : 0; XGB__scale_pos_weight : 2.0; XGB__subsample : 0.7	0.650	0.723
XGB	MM	ADASYN	XGB__alpha : 5.0; XGB__colsample_bytree : 0.5760919210120137; XGB__gamma : 0.1; XGB__max_depth : 3; XGB__min_child_weight : 32.41209755099559; XGB__random_state : 0; XGB__scale_pos_weight : 1.4474858421928154; XGB__subsample : 0.5815396095303651	0.650	0.712
XGB	MM	SMOTE	XGB__alpha : 5.0847870719083; XGB__colsample_bytree : 0.8560730276902576; XGB__gamma : 0.1; XGB__max_depth : 2; XGB__min_child_weight : 34.054533340356926; XGB__random_state : 0; XGB__scale_pos_weight : 1.9940777058431203; XGB__subsample : 0.6894420372017718	0.650	0.727

XGB	SS	ADASYN	XGB__alpha : 5.0; XGB__colsample_bytree : 0.5; XGB__gamma : 3.4470211671040367; XGB__max_depth : 1; XGB__min_child_weight : 27.79290671493711; XGB__random_state : 0; XGB__scale_pos_weight : 1.6634364151573753; XGB__subsample : 0.5	0.650	0.711
XGB	SS	SMOTE	XGB__alpha : 14.129280314425921; XGB__colsample_bytree : 0.5; XGB__gamma : 0.1; XGB__max_depth : 1; XGB__min_child_weight : 11.0257950614135; XGB__random_state : 0; XGB__scale_pos_weight : 2.0; XGB__subsample : 0.6962739793443856	0.649	0.723
XGB	MM	ADASYN	XGB__alpha : 6.505364620795106; XGB__colsample_bytree : 0.5475735878288952; XGB__gamma : 0.1; XGB__max_depth : 1; XGB__min_child_weight : 30.09956364610306; XGB__random_state : 0; XGB__scale_pos_weight : 1.93596789582916; XGB__subsample : 0.524927908391981	0.649	0.714
XGB	SS	ADASYN	XGB__alpha : 11.57333581097403; XGB__colsample_bytree : 0.5; XGB__gamma : 0.1; XGB__max_depth : 1; XGB__min_child_weight : 21.601295021607672; XGB__random_state : 0; XGB__scale_pos_weight : 1.8126354812894725; XGB__subsample : 0.5	0.649	0.719
XGB	SS	ADASYN	XGB__alpha : 5.0; XGB__colsample_bytree : 0.5; XGB__gamma : 5.0; XGB__max_depth : 1; XGB__min_child_weight : 50.0; XGB__random_state : 0; XGB__scale_pos_weight : 1.9238957247966377; XGB__subsample : 0.7	0.649	0.697

XGB	SS	SMOTE	XGB__alpha : 11.045265475242694; XGB__colsample_bytree : 1.0; XGB__gamma : 0.1; XGB__max_depth : 3; XGB__min_child_weight : 27.96743388776755; XGB__random_state : 0; XGB__scale_pos_weight : 2.0; XGB__subsample : 0.5	0.649	0.712
XGB	SS	ADASYN	XGB__alpha : 15.223943442166723; XGB__colsample_bytree : 0.5; XGB__gamma : 2.924043937926691; XGB__max_depth : 1; XGB__min_child_weight : 50.0; XGB__random_state : 0; XGB__scale_pos_weight : 1.8042221840506216; XGB__subsample : 0.7	0.648	0.692
XGB	SS	SMOTE	XGB__alpha : 5.0; XGB__colsample_bytree : 0.6212652676144056; XGB__gamma : 0.1; XGB__max_depth : 1; XGB__min_child_weight : 0.0; XGB__random_state : 0; XGB__scale_pos_weight : 0.7295402541781564; XGB__subsample : 0.6531270273933774	0.648	0.739
XGB	MM	SMOTE	XGB__alpha : 5.0; XGB__colsample_bytree : 0.8813803688090882; XGB__gamma : 3.5611985886706155; XGB__max_depth : 1; XGB__min_child_weight : 23.46878992849821; XGB__random_state : 0; XGB__scale_pos_weight : 1.189168496118092; XGB__subsample : 0.5685061370387613	0.648	0.711
XGB	SS	ADASYN	XGB__alpha : 6.389490064848099; XGB__colsample_bytree : 0.802981376600967; XGB__gamma : 0.3766716068908169; XGB__max_depth : 1; XGB__min_child_weight : 39.933748974672426; XGB__random_state : 0; XGB__scale_pos_weight : 1.4926037913994126; XGB__subsample : 0.6666624265289163	0.648	0.705

XGB	SS	ADASYN	XGB__alpha : 5.0; XGB__colsample_bytree : 0.5; XGB__gamma : 0.1; XGB__max_depth : 2; XGB__min_child_weight : 35.39911968331004; XGB__random_state : 0; XGB__scale_pos_weight : 1.4037005558005748; XGB__subsample : 0.7	0.648	0.719
XGB	SS	SMOTE	XGB__alpha : 8.704207077809748; XGB__colsample_bytree : 0.7818468782095859; XGB__gamma : 0.1; XGB__max_depth : 1; XGB__min_child_weight : 0.0; XGB__random_state : 0; XGB__scale_pos_weight : 1.9253213856810714; XGB__subsample : 0.6992016675833875	0.647	0.735
XGB	MM	ADASYN	XGB__alpha : 5.0; XGB__colsample_bytree : 0.9230566291195174; XGB__gamma : 2.806356147424415; XGB__max_depth : 2; XGB__min_child_weight : 24.916203072630342; XGB__random_state : 0; XGB__scale_pos_weight : 2.0; XGB__subsample : 0.5	0.647	0.713
XGB	SS	ADASYN	XGB__alpha : 14.654664456390485; XGB__colsample_bytree : 0.5; XGB__gamma : 0.1; XGB__max_depth : 1; XGB__min_child_weight : 29.57314269098548; XGB__random_state : 0; XGB__scale_pos_weight : 2.0; XGB__subsample : 0.5	0.647	0.705
XGB	MM	SMOTE	XGB__alpha : 5.0; XGB__colsample_bytree : 0.8092164695967409; XGB__gamma : 0.1; XGB__max_depth : 3; XGB__min_child_weight : 32.587360267774585; XGB__random_state : 0; XGB__scale_pos_weight : 2.0; XGB__subsample : 0.6239175328569142	0.647	0.725
XGB	SS	ADASYN	XGB__alpha : 5.0; XGB__colsample_bytree : 0.5; XGB__gamma : 0.1; XGB__max_depth : 1; XGB__min_child_weight : 34.332309181152986;	0.647	0.706

			XGB__random_state : 0; XGB__scale_pos_weight : 2.0; XGB__subsample : 0.5		
XGB	MM	SMOTE	XGB__alpha : 5.0; XGB__colsample_bytree : 0.5; XGB__gamma : 0.1; XGB__max_depth : 3; XGB__min_child_weight : 29.901913023975606; XGB__random_state : 0; XGB__scale_pos_weight : 2.0; XGB__subsample : 0.6378923259219701	0.647	0.731
XGB	MM	SMOTE	XGB__alpha : 5.0; XGB__colsample_bytree : 0.9892466705096656; XGB__gamma : 0.1; XGB__max_depth : 2; XGB__min_child_weight : 50.0; XGB__random_state : 0; XGB__scale_pos_weight : 2.0; XGB__subsample : 0.7	0.647	0.701
XGB	SS	ADASYN	XGB__alpha : 5.0; XGB__colsample_bytree : 0.654429478054703; XGB__gamma : 3.464379574024734; XGB__max_depth : 3; XGB__min_child_weight : 44.97059425227498; XGB__random_state : 0; XGB__scale_pos_weight : 2.0; XGB__subsample : 0.5943308183959563	0.647	0.693
XGB	SS	SMOTE	XGB__alpha : 5.0; XGB__colsample_bytree : 0.7863163139160485; XGB__gamma : 5.0; XGB__max_depth : 2; XGB__min_child_weight : 24.910126845176602; XGB__random_state : 0; XGB__scale_pos_weight : 1.2113785147779215; XGB__subsample : 0.7	0.647	0.721
XGB	SS	ADASYN	XGB__alpha : 15.868096749009974; XGB__colsample_bytree : 0.5; XGB__gamma : 0.1; XGB__max_depth : 2; XGB__min_child_weight : 50.0; XGB__random_state : 0; XGB__scale_pos_weight : 2.0; XGB__subsample : 0.7	0.647	0.699
RF	SS	ADASYN	RF__class_weight : balanced_subsample; RF__criterion : log_loss; RF__max_depth : 2;	0.647	0.718

			RF__max_features : 8; RF__min_samples_leaf : 6; RF__n_estimators : 614; RF__random_state : 65		
NN	SS	ADASYN	NN__activation : tanh; NN__alpha : 0.3184983766348907; NN__hidden_layer_sizes : 35; NN__max_iter : 237; NN__random_state : 0; NN__solver : adam	0.647	0.769
XGB	SS	SMOTE	XGB__alpha : 5.0; XGB__colsample_bytree : 1.0; XGB__gamma : 5.0; XGB__max_depth : 3; XGB__min_child_weight : 37.92246615752253; XGB__random_state : 0; XGB__scale_pos_weight : 2.0; XGB__subsample : 0.5379780671937486	0.646	0.702
XGB	SS	SMOTE	XGB__alpha : 5.0; XGB__colsample_bytree : 0.5; XGB__gamma : 2.4106379477706272; XGB__max_depth : 1; XGB__min_child_weight : 31.647899528708272; XGB__random_state : 0; XGB__scale_pos_weight : 1.990591567894769; XGB__subsample : 0.5	0.646	0.706
XGB	SS	ADASYN	XGB__alpha : 5.0; XGB__colsample_bytree : 0.5; XGB__gamma : 2.6242282899365024; XGB__max_depth : 1; XGB__min_child_weight : 17.076385017139156; XGB__random_state : 0; XGB__scale_pos_weight : 2.0; XGB__subsample : 0.5	0.646	0.722
XGB	MM	SMOTE	XGB__alpha : 5.555014824989462; XGB__colsample_bytree : 0.8136608731786787; XGB__gamma : 3.87949850561411; XGB__max_depth : 3; XGB__min_child_weight : 46.02881863076087; XGB__random_state : 0; XGB__scale_pos_weight : 2.0; XGB__subsample : 0.6665827585293885	0.646	0.700
XGB	SS	SMOTE	XGB__alpha : 16.349830223666636; XGB__colsample_bytree : 1.0; XGB__gamma : 0.1; XGB__max_depth : 1; XGB__min_child_weight : 0.0;	0.646	0.721

			XGB__random_state : 0; XGB__scale_pos_weight : 2.0; XGB__subsample : 0.7		
XGB	SS	ADASYN	XGB__alpha : 5.0; XGB__colsample_bytree : 1.0; XGB__gamma : 0.1; XGB__max_depth : 1; XGB__min_child_weight : 0.0; XGB__random_state : 0; XGB__scale_pos_weight : 0.5; XGB__subsample : 0.5	0.646	0.733
XGB	SS	SMOTE	XGB__alpha : 12.94016724446356; XGB__colsample_bytree : 0.7555692587730702; XGB__gamma : 0.1; XGB__max_depth : 1; XGB__min_child_weight : 4.604059493461397; XGB__random_state : 0; XGB__scale_pos_weight : 1.2113843128301593; XGB__subsample : 0.6817208353559527	0.646	0.722
XGB	SS	SMOTE	XGB__alpha : 5.0; XGB__colsample_bytree : 0.5; XGB__gamma : 5.0; XGB__max_depth : 3; XGB__min_child_weight : 33.61525605779739; XGB__random_state : 0; XGB__scale_pos_weight : 1.5943270502761795; XGB__subsample : 0.5	0.646	0.695
XGB	SS	ADASYN	XGB__alpha : 12.690541405047558; XGB__colsample_bytree : 0.5; XGB__gamma : 0.1; XGB__max_depth : 1; XGB__min_child_weight : 44.80020323319016; XGB__random_state : 0; XGB__scale_pos_weight : 1.79223470045007; XGB__subsample : 0.7	0.646	0.702
XGB	SS	ADASYN	XGB__alpha : 5.090757681368581; XGB__colsample_bytree : 0.9089150098318758; XGB__gamma : 3.7322854474033345; XGB__max_depth : 2; XGB__min_child_weight : 28.933216811419243; XGB__random_state : 0;	0.646	0.690

			XGB__scale_pos_weight : 0.9692589593519422; XGB__subsample : 0.5305189173676889		
XGB	SS	SMOTE	XGB__alpha : 5.0; XGB__colsample_bytree : 1.0; XGB__gamma : 0.1; XGB__max_depth : 3; XGB__min_child_weight : 35.76861192557142; XGB__random_state : 0; XGB__scale_pos_weight : 1.790707162660277; XGB__subsample : 0.5	0.646	0.699
XGB	MM	ADASYN	XGB__alpha : 6.828335713782861; XGB__colsample_bytree : 0.9589504168060757; XGB__gamma : 0.1; XGB__max_depth : 2; XGB__min_child_weight : 23.574481243074658; XGB__random_state : 0; XGB__scale_pos_weight : 0.8636797845630078; XGB__subsample : 0.5	0.646	0.699
XGB	MM	ADASYN	XGB__alpha : 5.0; XGB__colsample_bytree : 0.5500971989007697; XGB__gamma : 0.1; XGB__max_depth : 1; XGB__min_child_weight : 15.98180567778082; XGB__random_state : 0; XGB__scale_pos_weight : 0.9838311167256905; XGB__subsample : 0.5	0.646	0.724
XGB	SS	ADASYN	XGB__alpha : 5.0; XGB__colsample_bytree : 1.0; XGB__gamma : 0.1; XGB__max_depth : 1; XGB__min_child_weight : 35.29496259915357; XGB__random_state : 0; XGB__scale_pos_weight : 1.2443730515262763; XGB__subsample : 0.6106578643145091	0.645	0.702
XGB	SS	SMOTE	XGB__alpha : 5.0; XGB__colsample_bytree : 1.0; XGB__gamma : 0.1; XGB__max_depth : 3; XGB__min_child_weight : 35.371382982503256; XGB__random_state : 0; XGB__scale_pos_weight : 2.0; XGB__subsample : 0.7	0.645	0.728

XGB	MM	SMOTE	XGB__alpha : 5.0; XGB__colsample_bytree : 0.5; XGB__gamma : 0.1; XGB__max_depth : 3; XGB__min_child_weight : 50.0; XGB__random_state : 0; XGB__scale_pos_weight : 2.0; XGB__subsample : 0.7	0.645	0.697
XGB	SS	ADASYN	XGB__alpha : 5.0; XGB__colsample_bytree : 0.5; XGB__gamma : 0.6104411377671863; XGB__max_depth : 1; XGB__min_child_weight : 16.777980329250923; XGB__random_state : 0; XGB__scale_pos_weight : 1.5848982688405036; XGB__subsample : 0.5	0.645	0.730
XGB	SS	ADASYN	XGB__alpha : 11.014604677326979; XGB__colsample_bytree : 0.5434445849323879; XGB__gamma : 0.7772434129833954; XGB__max_depth : 1; XGB__min_child_weight : 37.85681623386644; XGB__random_state : 0; XGB__scale_pos_weight : 2.0; XGB__subsample : 0.6169110159099915	0.645	0.703
XGB	SS	ADASYN	XGB__alpha : 5.0; XGB__colsample_bytree : 0.9312464311386109; XGB__gamma : 0.1; XGB__max_depth : 1; XGB__min_child_weight : 29.85742369792121; XGB__random_state : 0; XGB__scale_pos_weight : 1.6008295671355113; XGB__subsample : 0.7	0.645	0.731
XGB	SS	SMOTE	XGB__alpha : 13.106405421775447; XGB__colsample_bytree : 0.5; XGB__gamma : 0.1; XGB__max_depth : 1; XGB__min_child_weight : 0.0; XGB__random_state : 0; XGB__scale_pos_weight : 1.2741479514907657; XGB__subsample : 0.7	0.645	0.721
XGB	MM	ADASYN	XGB__alpha : 5.0; XGB__colsample_bytree : 0.9458187950051224; XGB__gamma :	0.645	0.703

			4.544020954420247; XGB__max_depth : 1; XGB__min_child_weight : 31.47265565229504; XGB__random_state : 0; XGB__scale_pos_weight : 1.7112467395285698; XGB__subsample : 0.5674608928385733		
XGB	SS	ADASYN	XGB__alpha : 5.0; XGB__colsample_bytree : 0.5; XGB__gamma : 0.5210830103832461; XGB__max_depth : 3; XGB__min_child_weight : 38.1278119678781; XGB__random_state : 0; XGB__scale_pos_weight : 1.5698201304799229; XGB__subsample : 0.620291502842846	0.645	0.705
XGB	MM	SMOTE	XGB__alpha : 5.0; XGB__colsample_bytree : 0.8186004870289096; XGB__gamma : 3.9478774665581104; XGB__max_depth : 1; XGB__min_child_weight : 29.263828204867657; XGB__random_state : 0; XGB__scale_pos_weight : 1.290975669685075; XGB__subsample : 0.5783144139861306	0.645	0.705
XGB	SS	ADASYN	XGB__alpha : 5.0; XGB__colsample_bytree : 1.0; XGB__gamma : 3.3038875903320295; XGB__max_depth : 1; XGB__min_child_weight : 23.93592616950563; XGB__random_state : 0; XGB__scale_pos_weight : 2.0; XGB__subsample : 0.5	0.645	0.722
XGB	MM	ADASYN	XGB__alpha : 5.0; XGB__colsample_bytree : 1.0; XGB__gamma : 0.1; XGB__max_depth : 2; XGB__min_child_weight : 26.174477010078856; XGB__random_state : 0; XGB__scale_pos_weight : 0.8473506930391306; XGB__subsample : 0.5	0.644	0.692
RF	SS	ADASYN	RF__class_weight : None; RF__criterion : log_loss; RF__max_depth : 3; RF__max_features : 5;	0.644	0.753

			RF__min_samples_leaf : 1; RF__n_estimators : 445; RF__random_state : 100		
RF	MM	SMOTE	RF__class_weight : balanced_subsample; RF__criterion : log_loss; RF__max_depth : 2; RF__max_features : 3; RF__min_samples_leaf : 11; RF__n_estimators : 470; RF__random_state : 46	0.644	0.737
XGB	SS	SMOTE	XGB__alpha : 5.0; XGB__colsample_bytree : 1.0; XGB__gamma : 0.1; XGB__max_depth : 1; XGB__min_child_weight : 34.91505732296905; XGB__random_state : 0; XGB__scale_pos_weight : 2.0; XGB__subsample : 0.7	0.644	0.728
XGB	MM	SMOTE	XGB__alpha : 5.0; XGB__colsample_bytree : 0.5; XGB__gamma : 0.1; XGB__max_depth : 3; XGB__min_child_weight : 49.32918662998627; XGB__random_state : 0; XGB__scale_pos_weight : 1.9199855167561757; XGB__subsample : 0.6808018192896189	0.644	0.696
XGB	MM	SMOTE	XGB__alpha : 5.0; XGB__colsample_bytree : 0.5; XGB__gamma : 2.4789201044073765; XGB__max_depth : 2; XGB__min_child_weight : 21.317318502348016; XGB__random_state : 0; XGB__scale_pos_weight : 1.6932796247991477; XGB__subsample : 0.5	0.644	0.722
XGB	SS	SMOTE	XGB__alpha : 5.0; XGB__colsample_bytree : 0.5; XGB__gamma : 0.1; XGB__max_depth : 1; XGB__min_child_weight : 6.069270285354885; XGB__random_state : 0; XGB__scale_pos_weight : 0.9142335532025312; XGB__subsample : 0.7	0.644	0.739
XGB	SS	ADASYN	XGB__alpha : 5.0; XGB__colsample_bytree : 1.0; XGB__gamma : 0.1; XGB__max_depth : 1; XGB__min_child_weight : 35.92902767599311;	0.643	0.686

			XGB__random_state : 0; XGB__scale_pos_weight : 1.4560979758247516; XGB__subsample : 0.5		
XGB	SS	ADASYN	XGB__alpha : 5.0; XGB__colsample_bytree : 1.0; XGB__gamma : 5.0; XGB__max_depth : 1; XGB__min_child_weight : 50.0; XGB__random_state : 0; XGB__scale_pos_weight : 2.0; XGB__subsample : 0.7	0.643	0.699
XGB	SS	ADASYN	XGB__alpha : 5.0; XGB__colsample_bytree : 0.5; XGB__gamma : 1.593725263078724; XGB__max_depth : 3; XGB__min_child_weight : 22.96770129500678; XGB__random_state : 0; XGB__scale_pos_weight : 1.3234221598079956; XGB__subsample : 0.5	0.643	0.718
XGB	MM	SMOTE	XGB__alpha : 5.0; XGB__colsample_bytree : 0.5793871354638163; XGB__gamma : 2.135175777238684; XGB__max_depth : 3; XGB__min_child_weight : 24.97523848885872; XGB__random_state : 0; XGB__scale_pos_weight : 2.0; XGB__subsample : 0.532107668200475	0.643	0.721
XGB	MM	ADASYN	XGB__alpha : 5.0; XGB__colsample_bytree : 1.0; XGB__gamma : 0.1; XGB__max_depth : 1; XGB__min_child_weight : 24.822233736774376; XGB__random_state : 0; XGB__scale_pos_weight : 1.0560941798795729; XGB__subsample : 0.5601657872007464	0.643	0.716
XGB	MM	ADASYN	XGB__alpha : 6.682648842233059; XGB__colsample_bytree : 0.7959821257042177; XGB__gamma : 0.1; XGB__max_depth : 1; XGB__min_child_weight : 38.26825930773817; XGB__random_state : 0; XGB__scale_pos_weight : 1.31135133887779; XGB__subsample : 0.7	0.643	0.708

XGB	MM	ADASYN	XGB__alpha : 5.0; XGB__colsample_bytree : 0.5077765683712948; XGB__gamma : 3.4982070396120037; XGB__max_depth : 2; XGB__min_child_weight : 24.48101772134476; XGB__random_state : 0; XGB__scale_pos_weight : 2.0; XGB__subsample : 0.5	0.642	0.714
XGB	MM	SMOTE	XGB__alpha : 5.090757681368581; XGB__colsample_bytree : 0.9089150098318758; XGB__gamma : 3.7322854474033345; XGB__max_depth : 2; XGB__min_child_weight : 28.933216811419243; XGB__random_state : 0; XGB__scale_pos_weight : 0.9692589593519422; XGB__subsample : 0.5305189173676889	0.642	0.686
XGB	MM	ADASYN	XGB__alpha : 5.0; XGB__colsample_bytree : 0.7632963420740408; XGB__gamma : 2.4487777466835854; XGB__max_depth : 2; XGB__min_child_weight : 25.664108006195203; XGB__random_state : 0; XGB__scale_pos_weight : 1.2170515560952246; XGB__subsample : 0.5	0.642	0.703
XGB	MM	ADASYN	XGB__alpha : 5.0; XGB__colsample_bytree : 0.9557350378947429; XGB__gamma : 1.8809283149686082; XGB__max_depth : 2; XGB__min_child_weight : 25.769073125312154; XGB__random_state : 0; XGB__scale_pos_weight : 2.0; XGB__subsample : 0.5	0.642	0.716
XGB	MM	SMOTE	XGB__alpha : 9.610813214696833; XGB__colsample_bytree : 0.5; XGB__gamma : 2.496083933674486; XGB__max_depth : 1; XGB__min_child_weight : 34.030263952063486; XGB__random_state : 0; XGB__scale_pos_weight :	0.642	0.710

			1.899584803291475; XGB__subsample : 0.6796461564528673		
XGB	MM	ADASYN	XGB__alpha : 5.0; XGB__colsample_bytree : 0.9118031856681843; XGB__gamma : 3.283060487896752; XGB__max_depth : 3; XGB__min_child_weight : 24.968263168116444; XGB__random_state : 0; XGB__scale_pos_weight : 1.9067286778397454; XGB__subsample : 0.5	0.642	0.713
RF	SS	ADASYN	RF__class_weight : balanced_subsample; RF__criterion : log_loss; RF__max_depth : 1; RF__max_features : 3; RF__min_samples_leaf : 15; RF__n_estimators : 897; RF__random_state : 90	0.642	0.713
RF	SS	ADASYN	RF__class_weight : None; RF__criterion : log_loss; RF__max_depth : 2; RF__max_features : 9; RF__min_samples_leaf : 13; RF__n_estimators : 323; RF__random_state : 14	0.641	0.716
RF	SS	ADASYN	RF__class_weight : None; RF__criterion : log_loss; RF__max_depth : 1; RF__max_features : 3; RF__min_samples_leaf : 7; RF__n_estimators : 487; RF__random_state : 51	0.641	0.713
RF	MM	SMOTE	RF__class_weight : balanced; RF__criterion : gini; RF__max_depth : 1; RF__max_features : 2; RF__min_samples_leaf : 20; RF__n_estimators : 300; RF__random_state : 0	0.641	0.721
RF	SS	ADASYN	RF__class_weight : balanced; RF__criterion : log_loss; RF__max_depth : 4; RF__max_features : 3; RF__min_samples_leaf : 6; RF__n_estimators : 418; RF__random_state : 31	0.641	0.779
XGB	MM	ADASYN	XGB__alpha : 10.331149362834475; XGB__colsample_bytree : 0.757383253596286; XGB__gamma : 0.1; XGB__max_depth : 1;	0.641	0.712

			XGB__min_child_weight : 28.244738519918737; XGB__random_state : 0; XGB__scale_pos_weight : 1.6352415369130944; XGB__subsample : 0.6003339919157272		
XGB	MM	ADASYN	XGB__alpha : 9.138688430626786; XGB__colsample_bytree : 0.9816150455877549; XGB__gamma : 2.3410678419602227; XGB__max_depth : 1; XGB__min_child_weight : 25.424861302988226; XGB__random_state : 0; XGB__scale_pos_weight : 2.0; XGB__subsample : 0.5	0.640	0.705
RF	SS	ADASYN	RF__class_weight : balanced_subsample; RF__criterion : log_loss; RF__max_depth : 3; RF__max_features : 8; RF__min_samples_leaf : 3; RF__n_estimators : 319; RF__random_state : 57	0.640	0.747
XGB	MM	ADASYN	XGB__alpha : 6.53173065258426; XGB__colsample_bytree : 1.0; XGB__gamma : 0.1; XGB__max_depth : 3; XGB__min_child_weight : 23.58071968410965; XGB__random_state : 0; XGB__scale_pos_weight : 1.2485626334583546; XGB__subsample : 0.5076127783456571	0.640	0.713
XGB	MM	ADASYN	XGB__alpha : 5.024826632028878; XGB__colsample_bytree : 0.607507595086666; XGB__gamma : 2.5114251347540666; XGB__max_depth : 2; XGB__min_child_weight : 21.975148208105896; XGB__random_state : 0; XGB__scale_pos_weight : 0.8004227893896005; XGB__subsample : 0.5374979839310523	0.640	0.700
XGB	MM	SMOTE	XGB__alpha : 5.0; XGB__colsample_bytree : 0.5; XGB__gamma : 1.5303206552835271; XGB__max_depth : 2; XGB__min_child_weight : 28.29673162640361; XGB__random_state : 0;	0.640	0.709

			XGB__scale_pos_weight : 1.6048062657022475; XGB__subsample : 0.5		
RF	SS	ADASYN	RF__class_weight : balanced_subsample; RF__criterion : gini; RF__max_depth : 2; RF__max_features : 3; RF__min_samples_leaf : 20; RF__n_estimators : 895; RF__random_state : 73	0.640	0.726
XGB	MM	ADASYN	XGB__alpha : 5.0; XGB__colsample_bytree : 0.7830652058141597; XGB__gamma : 0.1; XGB__max_depth : 2; XGB__min_child_weight : 20.902230624047338; XGB__random_state : 0; XGB__scale_pos_weight : 0.9487905507204323; XGB__subsample : 0.5251617649513477	0.640	0.718
XGB	MM	ADASYN	XGB__alpha : 5.0; XGB__colsample_bytree : 0.904476155681954; XGB__gamma : 2.6525593861219265; XGB__max_depth : 2; XGB__min_child_weight : 22.86105851841431; XGB__random_state : 0; XGB__scale_pos_weight : 1.0055481801673023; XGB__subsample : 0.5	0.639	0.703
XGB	MM	SMOTE	XGB__alpha : 30.0; XGB__colsample_bytree : 0.5; XGB__gamma : 0.1; XGB__max_depth : 1; XGB__min_child_weight : 0.0; XGB__random_state : 0; XGB__scale_pos_weight : 1.5194070724266835; XGB__subsample : 0.5	0.639	0.688
XGB	MM	SMOTE	XGB__alpha : 5.0; XGB__colsample_bytree : 0.5; XGB__gamma : 0.5560886450468363; XGB__max_depth : 3; XGB__min_child_weight : 22.06792380347624; XGB__random_state : 0; XGB__scale_pos_weight : 1.9836774131711135; XGB__subsample : 0.513029158504781	0.639	0.731
XGB	MM	SMOTE	XGB__alpha : 5.0; XGB__colsample_bytree : 1.0; XGB__gamma : 3.3428174387647167;	0.638	0.719

			XGB__max_depth : 1; XGB__min_child_weight : 29.0094485985584; XGB__random_state : 0; XGB__scale_pos_weight : 1.6433391737264327; XGB__subsample : 0.7		
XGB	MM	ADASYN	XGB__alpha : 5.0; XGB__colsample_bytree : 0.8464017889354936; XGB__gamma : 2.789425688176876; XGB__max_depth : 3; XGB__min_child_weight : 24.05925237186569; XGB__random_state : 0; XGB__scale_pos_weight : 2.0; XGB__subsample : 0.5	0.638	0.714
XGB	MM	SMOTE	XGB__alpha : 5.0; XGB__colsample_bytree : 1.0; XGB__gamma : 4.0241424936389345; XGB__max_depth : 1; XGB__min_child_weight : 31.949573776380756; XGB__random_state : 0; XGB__scale_pos_weight : 2.0; XGB__subsample : 0.7	0.638	0.716
XGB	MM	SMOTE	XGB__alpha : 5.0; XGB__colsample_bytree : 0.5466719428805302; XGB__gamma : 0.1; XGB__max_depth : 1; XGB__min_child_weight : 0.0; XGB__random_state : 0; XGB__scale_pos_weight : 1.1419602419721246; XGB__subsample : 0.6005673810243899	0.637	0.739
XGB	MM	SMOTE	XGB__alpha : 12.899472874131337; XGB__colsample_bytree : 1.0; XGB__gamma : 0.1; XGB__max_depth : 3; XGB__min_child_weight : 28.592127912125463; XGB__random_state : 0; XGB__scale_pos_weight : 1.7869053519864724; XGB__subsample : 0.5	0.637	0.704
XGB	MM	ADASYN	XGB__alpha : 5.0; XGB__colsample_bytree : 0.9716377416872342; XGB__gamma : 3.1389689669089993; XGB__max_depth : 2; XGB__min_child_weight : 22.454326191301643;	0.637	0.690

			XGB__random_state : 0; XGB__scale_pos_weight : 0.7976241387808932; XGB__subsample : 0.5		
XGB	MM	ADASYN	XGB__alpha : 8.495652245314343; XGB__colsample_bytree : 0.6641387807088202; XGB__gamma : 0.8831447638334863; XGB__max_depth : 1; XGB__min_child_weight : 26.513980700965362; XGB__random_state : 0; XGB__scale_pos_weight : 1.361350461624014; XGB__subsample : 0.5013293298143793	0.637	0.702
XGB	MM	ADASYN	XGB__alpha : 14.516125569122034; XGB__colsample_bytree : 0.7160312574064115; XGB__gamma : 0.1; XGB__max_depth : 1; XGB__min_child_weight : 31.958393761023522; XGB__random_state : 0; XGB__scale_pos_weight : 2.0; XGB__subsample : 0.5	0.636	0.694
RF	SS	ADASYN	RF__class_weight : balanced_subsample; RF__criterion : gini; RF__max_depth : 4; RF__max_features : 1; RF__min_samples_leaf : 1; RF__n_estimators : 518; RF__random_state : 1	0.636	0.803
RF	SS	ADASYN	RF__class_weight : balanced_subsample; RF__criterion : log_loss; RF__max_depth : 1; RF__max_features : 4; RF__min_samples_leaf : 2; RF__n_estimators : 518; RF__random_state : 16	0.631	0.706
RF	SS	ADASYN	RF__class_weight : balanced_subsample; RF__criterion : gini; RF__max_depth : 3; RF__max_features : 9; RF__min_samples_leaf : 1; RF__n_estimators : 841; RF__random_state : 1	0.631	0.764
NN	SS	ADASYN	NN__activation : tanh; NN__alpha : 0.0001; NN__hidden_layer_sizes : 3; NN__max_iter : 500; NN__random_state : 0; NN__solver : lbfgs	0.624	0.764

NN	SS	ADASYN	NN__activation : relu; NN__alpha : 0.0007233655252968397; NN__hidden_layer_sizes : 22; NN__max_iter : 411; NN__random_state : 0; NN__solver : adam	0.593	0.810
NN	SS	ADASYN	NN__activation : logistic; NN__alpha : 0.0001; NN__hidden_layer_sizes : 50; NN__max_iter : 50; NN__random_state : 0; NN__solver : lbfgs	0.569	0.834
NN	SS	ADASYN	NN__activation : relu; NN__alpha : 0.7530466730222446; NN__hidden_layer_sizes : 9; NN__max_iter : 443; NN__random_state : 0; NN__solver : lbfgs	0.548	0.855
NN	SS	ADASYN	NN__activation : relu; NN__alpha : 0.01549337141764052; NN__hidden_layer_sizes : 20; NN__max_iter : 372; NN__random_state : 0; NN__solver : lbfgs	0.522	0.963

ii. 1-year VT recurrence

Model	Preprocessing	Oversampling	Parameters	Mean AUC (test)	Mean AUC (train)
RF	SS	SMOTE	RF__class_weight : balanced_subsample; RF__criterion : gini; RF__max_depth : 2; RF__max_features : 7; RF__min_samples_leaf : 2; RF__n_estimators : 320; RF__random_state : 100	0.713114	0.751557
RF	MM	SMOTE	RF__class_weight : balanced_subsample; RF__criterion : gini; RF__max_depth : 2; RF__max_features : 9; RF__min_samples_leaf : 1; RF__n_estimators : 1000; RF__random_state : 0	0.712971	0.751631
RF	SS	SMOTE	RF__class_weight : balanced; RF__criterion : gini; RF__max_depth : 2; RF__max_features : 6; RF__min_samples_leaf : 1; RF__n_estimators : 300; RF__random_state : 100	0.712873	0.752107
RF	SS	SMOTE	RF__class_weight : balanced; RF__criterion : gini; RF__max_depth : 2; RF__max_features : 6; RF__min_samples_leaf : 2; RF__n_estimators : 322; RF__random_state : 100	0.712873	0.751591
RF	SS	SMOTE	RF__class_weight : balanced_subsample; RF__criterion : gini; RF__max_depth : 2; RF__max_features : 6; RF__min_samples_leaf : 1; RF__n_estimators : 300; RF__random_state : 100	0.712683	0.751863
RF	Not used	Not used	RF__class_weight : None; RF__criterion : gini; RF__max_depth : 3; RF__max_features : 1; RF__min_samples_leaf : 2; RF__n_estimators : 1000; RF__random_state : 58	0.712572	0.802466
RF	MM	SMOTE	RF__class_weight : balanced_subsample; RF__criterion : gini; RF__max_depth : 2;	0.711336	0.751717

			RF__max_features : 9; RF__min_samples_leaf : 1; RF__n_estimators : 765; RF__random_state : 0		
RF	MM	SMOTE	RF__class_weight : balanced_subsample; RF__criterion : gini; RF__max_depth : 2; RF__max_features : 7; RF__min_samples_leaf : 1; RF__n_estimators : 602; RF__random_state : 100	0.711164	0.749136
XGB	Not used	Not used	XGB__colsample_bytree : 1.0; XGB__gamma : 9.791519809878324; XGB__max_depth : 3; XGB__min_child_weight : 0.0; XGB__random_state : 0; XGB__scale_pos_weight : 3.135366643716628; XGB__subsample : 0.8976962358465848	0.711151	0.810438
RF	SS	SMOTE	RF__class_weight : None; RF__criterion : gini; RF__max_depth : 2; RF__max_features : 6; RF__min_samples_leaf : 1; RF__n_estimators : 446; RF__random_state : 100	0.711082	0.752145
RF	SS	SMOTE	RF__class_weight : balanced_subsample; RF__criterion : log_loss; RF__max_depth : 2; RF__max_features : 6; RF__min_samples_leaf : 2; RF__n_estimators : 435; RF__random_state : 100	0.711037	0.747612
RF	MM	SMOTE	RF__class_weight : None; RF__criterion : gini; RF__max_depth : 2; RF__max_features : 7; RF__min_samples_leaf : 1; RF__n_estimators : 1000; RF__random_state : 0	0.710973	0.751843
RF	MM	SMOTE	RF__class_weight : balanced; RF__criterion : gini; RF__max_depth : 2; RF__max_features : 5; RF__min_samples_leaf : 1; RF__n_estimators : 924; RF__random_state : 100	0.710785	0.750287
RF	MM	SMOTE	RF__class_weight : None; RF__criterion : gini; RF__max_depth : 2; RF__max_features : 7; RF__min_samples_leaf : 1; RF__n_estimators : 1000; RF__random_state : 100	0.71075	0.750887

RF	MM	SMOTE	RF__class_weight : balanced; RF__criterion : gini; RF__max_depth : 2; RF__max_features : 9; RF__min_samples_leaf : 1; RF__n_estimators : 360; RF__random_state : 9	0.710472	0.752736
RF	SS	SMOTE	RF__class_weight : balanced_subsample; RF__criterion : gini; RF__max_depth : 2; RF__max_features : 7; RF__min_samples_leaf : 2; RF__n_estimators : 720; RF__random_state : 87	0.710248	0.753066
RF	Not used	Not used	RF__class_weight : None; RF__criterion : gini; RF__max_depth : 3; RF__max_features : 1; RF__min_samples_leaf : 2; RF__n_estimators : 1000; RF__random_state : 100	0.709988	0.801734
RF	Not used	Not used	RF__class_weight : None; RF__criterion : gini; RF__max_depth : 3; RF__max_features : 1; RF__min_samples_leaf : 2; RF__n_estimators : 1000; RF__random_state : 67	0.709783	0.802199
NN	SS	SMOTE	NN__activation : logistic; NN__alpha : 0.00019205456188436704; NN__hidden_layer_sizes : 50; NN__max_iter : 50; NN__random_state : 0; NN__solver : adam	0.709466	0.701448
RF	MM	SMOTE	RF__class_weight : balanced; RF__criterion : gini; RF__max_depth : 2; RF__max_features : 5; RF__min_samples_leaf : 1; RF__n_estimators : 327; RF__random_state : 99	0.709324	0.750896
RF	MM	ADASYN	RF__class_weight : balanced; RF__criterion : gini; RF__max_depth : 3; RF__max_features : 6; RF__min_samples_leaf : 2; RF__n_estimators : 955; RF__random_state : 30	0.709171	0.785111
NN	SS	SMOTE	NN__activation : logistic; NN__alpha : 0.04830937680960537; NN__hidden_layer_sizes : 50;	0.709053	0.701448

			NN__max_iter : 50; NN__random_state : 0; NN__solver : adam		
NN	SS	ROS	NN__activation : logistic; NN__alpha : 0.0001; NN__hidden_layer_sizes : 50; NN__max_iter : 50; NN__random_state : 0; NN__solver : adam	0.709035	0.700632
RF	Not used	Not used	RF__class_weight : None; RF__criterion : gini; RF__max_depth : 3; RF__max_features : 1; RF__min_samples_leaf : 3; RF__n_estimators : 1000; RF__random_state : 100	0.708817	0.799239
RF	Not used	Not used	RF__class_weight : None; RF__criterion : log_loss; RF__max_depth : 2; RF__max_features : 1; RF__min_samples_leaf : 1; RF__n_estimators : 401; RF__random_state : 58	0.708755	0.775912
RF	Not used	Not used	RF__class_weight : None; RF__criterion : gini; RF__max_depth : 3; RF__max_features : 1; RF__min_samples_leaf : 3; RF__n_estimators : 1000; RF__random_state : 67	0.70856	0.799681
RF	Not used	Not used	RF__class_weight : None; RF__criterion : log_loss; RF__max_depth : 3; RF__max_features : 1; RF__min_samples_leaf : 1; RF__n_estimators : 414; RF__random_state : 45	0.708492	0.805885
RF	Not used	Not used	RF__class_weight : None; RF__criterion : gini; RF__max_depth : 3; RF__max_features : 1; RF__min_samples_leaf : 2; RF__n_estimators : 1000; RF__random_state : 24	0.708438	0.802004
RF	MM	SMOTE	RF__class_weight : None; RF__criterion : gini; RF__max_depth : 2; RF__max_features : 5; RF__min_samples_leaf : 1; RF__n_estimators : 395; RF__random_state : 95	0.708374	0.751916
RF	MM	SMOTE	RF__class_weight : None; RF__criterion : gini; RF__max_depth : 2; RF__max_features : 5;	0.70834	0.74744

			RF__min_samples_leaf : 3; RF__n_estimators : 529; RF__random_state : 100		
RF	Not used	Not used	RF__class_weight : None; RF__criterion : gini; RF__max_depth : 3; RF__max_features : 1; RF__min_samples_leaf : 2; RF__n_estimators : 300; RF__random_state : 53	0.708302	0.801074
RF	MM	SMOTE	RF__class_weight : None; RF__criterion : gini; RF__max_depth : 2; RF__max_features : 6; RF__min_samples_leaf : 1; RF__n_estimators : 377; RF__random_state : 6	0.708289	0.752328
NN	SS	ROS	NN__activation : logistic; NN__alpha : 0.0001; NN__hidden_layer_sizes : 50; NN__max_iter : 59; NN__random_state : 0; NN__solver : adam	0.70826	0.701019
RF	MM	SMOTE	RF__class_weight : None; RF__criterion : gini; RF__max_depth : 2; RF__max_features : 9; RF__min_samples_leaf : 1; RF__n_estimators : 540; RF__random_state : 0	0.708235	0.75109
RF	Not used	Not used	RF__class_weight : None; RF__criterion : log_loss; RF__max_depth : 3; RF__max_features : 1; RF__min_samples_leaf : 2; RF__n_estimators : 1000; RF__random_state : 41	0.707699	0.802826
XGB	SS	SMOTE	XGB__colsample_bytree : 1.0; XGB__gamma : 3.7851564085172176; XGB__max_depth : 1; XGB__min_child_weight : 6.240836183879521; XGB__random_state : 0; XGB__scale_pos_weight : 3.773237969910909; XGB__subsample : 0.9	0.707403	0.766053
RF	Not used	Not used	RF__class_weight : None; RF__criterion : gini; RF__max_depth : 2; RF__max_features : 1; RF__min_samples_leaf : 2; RF__n_estimators : 433; RF__random_state : 72	0.707289	0.772737

RF	MM	SMOTE	RF__class_weight : None; RF__criterion : gini; RF__max_depth : 2; RF__max_features : 7; RF__min_samples_leaf : 1; RF__n_estimators : 1000; RF__random_state : 2	0.707221	0.751897
RF	SS	ADASYN	RF__class_weight : None; RF__criterion : gini; RF__max_depth : 3; RF__max_features : 1; RF__min_samples_leaf : 1; RF__n_estimators : 500; RF__random_state : 5	0.707154	0.792279
RF	MM	ADASYN	RF__class_weight : None; RF__criterion : gini; RF__max_depth : 3; RF__max_features : 9; RF__min_samples_leaf : 1; RF__n_estimators : 558; RF__random_state : 59	0.706985	0.785991
RF	MM	ADASYN	RF__class_weight : None; RF__criterion : gini; RF__max_depth : 3; RF__max_features : 7; RF__min_samples_leaf : 2; RF__n_estimators : 1000; RF__random_state : 0	0.706915	0.784632
RF	SS	ADASYN	RF__class_weight : None; RF__criterion : gini; RF__max_depth : 3; RF__max_features : 1; RF__min_samples_leaf : 2; RF__n_estimators : 305; RF__random_state : 60	0.706758	0.789179
RF	Not used	Not used	RF__class_weight : None; RF__criterion : gini; RF__max_depth : 3; RF__max_features : 1; RF__min_samples_leaf : 2; RF__n_estimators : 1000; RF__random_state : 0	0.706649	0.801521
RF	Not used	Not used	RF__class_weight : None; RF__criterion : log_loss; RF__max_depth : 3; RF__max_features : 1; RF__min_samples_leaf : 2; RF__n_estimators : 1000; RF__random_state : 100	0.706546	0.802347
RF	SS	ADASYN	RF__class_weight : None; RF__criterion : log_loss; RF__max_depth : 3; RF__max_features : 1;	0.706462	0.791803

			RF__min_samples_leaf : 1; RF__n_estimators : 967; RF__random_state : 11		
RF	SS	SMOTE	RF__class_weight : balanced_subsample; RF__criterion : log_loss; RF__max_depth : 3; RF__max_features : 1; RF__min_samples_leaf : 2; RF__n_estimators : 329; RF__random_state : 98	0.706257	0.787812
RF	Not used	Not used	RF__class_weight : None; RF__criterion : log_loss; RF__max_depth : 3; RF__max_features : 1; RF__min_samples_leaf : 2; RF__n_estimators : 499; RF__random_state : 100	0.706166	0.802518
RF	MM	ADASYN	RF__class_weight : balanced; RF__criterion : gini; RF__max_depth : 3; RF__max_features : 6; RF__min_samples_leaf : 4; RF__n_estimators : 300; RF__random_state : 84	0.706139	0.780628
RF	MM	SMOTE	RF__class_weight : balanced_subsample; RF__criterion : gini; RF__max_depth : 2; RF__max_features : 9; RF__min_samples_leaf : 1; RF__n_estimators : 991; RF__random_state : 21	0.706136	0.75292
RF	SS	ADASYN	RF__class_weight : None; RF__criterion : gini; RF__max_depth : 3; RF__max_features : 1; RF__min_samples_leaf : 3; RF__n_estimators : 341; RF__random_state : 33	0.706015	0.789281
RF	SS	SMOTE	RF__class_weight : balanced_subsample; RF__criterion : gini; RF__max_depth : 2; RF__max_features : 8; RF__min_samples_leaf : 1; RF__n_estimators : 713; RF__random_state : 90	0.705944	0.753496
RF	Not used	Not used	RF__class_weight : balanced; RF__criterion : log_loss; RF__max_depth : 3; RF__max_features : 1; RF__min_samples_leaf : 1; RF__n_estimators : 1000; RF__random_state : 43	0.705893	0.804836

RF	SS	ADASYN	RF__class_weight : None; RF__criterion : gini; RF__max_depth : 3; RF__max_features : 1; RF__min_samples_leaf : 1; RF__n_estimators : 1000; RF__random_state : 100	0.705706	0.791993
RF	Not used	Not used	RF__class_weight : None; RF__criterion : gini; RF__max_depth : 3; RF__max_features : 1; RF__min_samples_leaf : 2; RF__n_estimators : 392; RF__random_state : 100	0.70558	0.801651
RF	Not used	Not used	RF__class_weight : None; RF__criterion : log_loss; RF__max_depth : 3; RF__max_features : 1; RF__min_samples_leaf : 1; RF__n_estimators : 643; RF__random_state : 6	0.705547	0.80434
RF	Not used	Not used	RF__class_weight : None; RF__criterion : log_loss; RF__max_depth : 3; RF__max_features : 1; RF__min_samples_leaf : 3; RF__n_estimators : 1000; RF__random_state : 100	0.70546	0.800107
RF	SS	ADASYN	RF__class_weight : None; RF__criterion : gini; RF__max_depth : 3; RF__max_features : 1; RF__min_samples_leaf : 1; RF__n_estimators : 1000; RF__random_state : 95	0.705447	0.791467
RF	MM	ADASYN	RF__class_weight : balanced; RF__criterion : gini; RF__max_depth : 3; RF__max_features : 7; RF__min_samples_leaf : 1; RF__n_estimators : 1000; RF__random_state : 27	0.705434	0.787929
RF	MM	SMOTE	RF__class_weight : None; RF__criterion : gini; RF__max_depth : 2; RF__max_features : 5; RF__min_samples_leaf : 3; RF__n_estimators : 875; RF__random_state : 17	0.705413	0.750186
RF	SS	SMOTE	RF__class_weight : balanced_subsample; RF__criterion : gini; RF__max_depth : 2;	0.705325	0.752564

			RF__max_features : 9; RF__min_samples_leaf : 2; RF__n_estimators : 328; RF__random_state : 94		
RF	Not used	Not used	RF__class_weight : None; RF__criterion : gini; RF__max_depth : 4; RF__max_features : 1; RF__min_samples_leaf : 1; RF__n_estimators : 1000; RF__random_state : 47	0.70529	0.840891
XGB	MM	ROS	XGB__colsample_bytree : 1.0; XGB__gamma : 16.163930617339958; XGB__max_depth : 1; XGB__min_child_weight : 12.12990798989308; XGB__random_state : 0; XGB__scale_pos_weight : 4.457452531197775; XGB__subsample : 0.7710463290923624	0.705242	0.735828
RF	MM	SMOTE	RF__class_weight : balanced; RF__criterion : gini; RF__max_depth : 2; RF__max_features : 9; RF__min_samples_leaf : 1; RF__n_estimators : 368; RF__random_state : 88	0.705086	0.75332
RF	Not used	Not used	RF__class_weight : None; RF__criterion : gini; RF__max_depth : 3; RF__max_features : 1; RF__min_samples_leaf : 3; RF__n_estimators : 1000; RF__random_state : 0	0.705065	0.798511
RF	Not used	Not used	RF__class_weight : None; RF__criterion : log_loss; RF__max_depth : 3; RF__max_features : 1; RF__min_samples_leaf : 1; RF__n_estimators : 1000; RF__random_state : 0	0.704995	0.804406
RF	MM	ADASYN	RF__class_weight : balanced; RF__criterion : gini; RF__max_depth : 3; RF__max_features : 9; RF__min_samples_leaf : 1; RF__n_estimators : 552; RF__random_state : 31	0.704969	0.788347
RF	SS	ADASYN	RF__class_weight : None; RF__criterion : gini; RF__max_depth : 3; RF__max_features : 1;	0.704829	0.792973

			RF__min_samples_leaf : 1; RF__n_estimators : 1000; RF__random_state : 53		
RF	MM	SMOTE	RF__class_weight : None; RF__criterion : gini; RF__max_depth : 2; RF__max_features : 4; RF__min_samples_leaf : 1; RF__n_estimators : 961; RF__random_state : 10	0.704726	0.75211
RF	SS	ADASYN	RF__class_weight : None; RF__criterion : log_loss; RF__max_depth : 2; RF__max_features : 1; RF__min_samples_leaf : 1; RF__n_estimators : 861; RF__random_state : 61	0.704726	0.763041
RF	MM	ADASYN	RF__class_weight : balanced_subsample; RF__criterion : gini; RF__max_depth : 3; RF__max_features : 4; RF__min_samples_leaf : 1; RF__n_estimators : 300; RF__random_state : 100	0.704691	0.788642
RF	MM	ADASYN	RF__class_weight : balanced; RF__criterion : log_loss; RF__max_depth : 2; RF__max_features : 1; RF__min_samples_leaf : 1; RF__n_estimators : 300; RF__random_state : 85	0.704677	0.759364
RF	MM	ADASYN	RF__class_weight : balanced; RF__criterion : gini; RF__max_depth : 3; RF__max_features : 5; RF__min_samples_leaf : 2; RF__n_estimators : 1000; RF__random_state : 0	0.704642	0.785165
RF	MM	SMOTE	RF__class_weight : balanced; RF__criterion : gini; RF__max_depth : 2; RF__max_features : 4; RF__min_samples_leaf : 1; RF__n_estimators : 949; RF__random_state : 83	0.704552	0.750599
RF	MM	SMOTE	RF__class_weight : None; RF__criterion : gini; RF__max_depth : 2; RF__max_features : 7; RF__min_samples_leaf : 1; RF__n_estimators : 861; RF__random_state : 92	0.704465	0.751463

RF	MM	ADASYN	RF__class_weight : balanced; RF__criterion : gini; RF__max_depth : 3; RF__max_features : 9; RF__min_samples_leaf : 1; RF__n_estimators : 1000; RF__random_state : 0	0.704332	0.788509
RF	MM	ADASYN	RF__class_weight : None; RF__criterion : gini; RF__max_depth : 3; RF__max_features : 2; RF__min_samples_leaf : 3; RF__n_estimators : 300; RF__random_state : 95	0.704257	0.785451
RF	MM	ADASYN	RF__class_weight : None; RF__criterion : gini; RF__max_depth : 3; RF__max_features : 9; RF__min_samples_leaf : 1; RF__n_estimators : 1000; RF__random_state : 0	0.704247	0.788107
RF	SS	ADASYN	RF__class_weight : None; RF__criterion : gini; RF__max_depth : 3; RF__max_features : 1; RF__min_samples_leaf : 1; RF__n_estimators : 1000; RF__random_state : 37	0.704244	0.79328
RF	SS	ADASYN	RF__class_weight : None; RF__criterion : log_loss; RF__max_depth : 3; RF__max_features : 1; RF__min_samples_leaf : 1; RF__n_estimators : 1000; RF__random_state : 0	0.704242	0.790797
RF	SS	ADASYN	RF__class_weight : balanced; RF__criterion : log_loss; RF__max_depth : 3; RF__max_features : 1; RF__min_samples_leaf : 2; RF__n_estimators : 1000; RF__random_state : 0	0.70424	0.789084
RF	SS	ADASYN	RF__class_weight : balanced; RF__criterion : log_loss; RF__max_depth : 2; RF__max_features : 1; RF__min_samples_leaf : 5; RF__n_estimators : 633; RF__random_state : 62	0.704194	0.760584
RF	SS	ADASYN	RF__class_weight : balanced; RF__criterion : log_loss; RF__max_depth : 4; RF__max_features : 9;	0.704096	0.826472

			RF__min_samples_leaf : 1; RF__n_estimators : 709; RF__random_state : 100		
RF	MM	ADASYN	RF__class_weight : None; RF__criterion : gini; RF__max_depth : 2; RF__max_features : 1; RF__min_samples_leaf : 5; RF__n_estimators : 743; RF__random_state : 24	0.704057	0.760253
RF	SS	ADASYN	RF__class_weight : None; RF__criterion : gini; RF__max_depth : 3; RF__max_features : 1; RF__min_samples_leaf : 1; RF__n_estimators : 795; RF__random_state : 99	0.703988	0.793396
XGB	SS	SMOTE	XGB__colsample_bytree : 1.0; XGB__gamma : 4.254911587555754; XGB__max_depth : 1; XGB__min_child_weight : 9.338139007486001; XGB__random_state : 0; XGB__scale_pos_weight : 4.158272306235727; XGB__subsample : 0.9	0.703949	0.754206
RF	MM	ADASYN	RF__class_weight : balanced; RF__criterion : gini; RF__max_depth : 3; RF__max_features : 5; RF__min_samples_leaf : 2; RF__n_estimators : 934; RF__random_state : 0	0.703885	0.785167
RF	MM	SMOTE	RF__class_weight : balanced; RF__criterion : gini; RF__max_depth : 2; RF__max_features : 9; RF__min_samples_leaf : 1; RF__n_estimators : 875; RF__random_state : 21	0.70388	0.752373
RF	MM	SMOTE	RF__class_weight : balanced; RF__criterion : gini; RF__max_depth : 2; RF__max_features : 9; RF__min_samples_leaf : 1; RF__n_estimators : 744; RF__random_state : 7	0.703742	0.75174
RF	SS	ADASYN	RF__class_weight : None; RF__criterion : gini; RF__max_depth : 3; RF__max_features : 1; RF__min_samples_leaf : 1; RF__n_estimators : 541; RF__random_state : 4	0.703709	0.791167

RF	SS	ADASYN	RF__class_weight : None; RF__criterion : log_loss; RF__max_depth : 3; RF__max_features : 1; RF__min_samples_leaf : 1; RF__n_estimators : 329; RF__random_state : 47	0.703689	0.79016
RF	SS	ADASYN	RF__class_weight : balanced; RF__criterion : gini; RF__max_depth : 2; RF__max_features : 1; RF__min_samples_leaf : 1; RF__n_estimators : 943; RF__random_state : 87	0.703689	0.761277
RF	Not used	Not used	RF__class_weight : balanced_subsample; RF__criterion : log_loss; RF__max_depth : 3; RF__max_features : 1; RF__min_samples_leaf : 1; RF__n_estimators : 599; RF__random_state : 97	0.703688	0.803944
RF	MM	ADASYN	RF__class_weight : balanced_subsample; RF__criterion : gini; RF__max_depth : 3; RF__max_features : 4; RF__min_samples_leaf : 3; RF__n_estimators : 319; RF__random_state : 6	0.703675	0.784502
RF	SS	ADASYN	RF__class_weight : None; RF__criterion : gini; RF__max_depth : 3; RF__max_features : 1; RF__min_samples_leaf : 1; RF__n_estimators : 382; RF__random_state : 100	0.703673	0.791778
RF	SS	ADASYN	RF__class_weight : None; RF__criterion : log_loss; RF__max_depth : 3; RF__max_features : 1; RF__min_samples_leaf : 3; RF__n_estimators : 312; RF__random_state : 28	0.703622	0.787261
RF	Not used	Not used	RF__class_weight : None; RF__criterion : log_loss; RF__max_depth : 3; RF__max_features : 2; RF__min_samples_leaf : 1; RF__n_estimators : 753; RF__random_state : 81	0.703617	0.807143
RF	MM	ADASYN	RF__class_weight : balanced; RF__criterion : gini; RF__max_depth : 3; RF__max_features : 5;	0.703575	0.784301

			RF__min_samples_leaf : 2; RF__n_estimators : 906; RF__random_state : 100		
RF	SS	ADASYN	RF__class_weight : None; RF__criterion : log_loss; RF__max_depth : 4; RF__max_features : 6; RF__min_samples_leaf : 1; RF__n_estimators : 972; RF__random_state : 22	0.703545	0.828357
RF	MM	ADASYN	RF__class_weight : balanced; RF__criterion : gini; RF__max_depth : 3; RF__max_features : 7; RF__min_samples_leaf : 1; RF__n_estimators : 308; RF__random_state : 38	0.703539	0.788739
XGB	SS	SMOTE	XGB__colsample_bytree : 1.0; XGB__gamma : 4.918964170968306; XGB__max_depth : 1; XGB__min_child_weight : 6.140280281648867; XGB__random_state : 0; XGB__scale_pos_weight : 5.0; XGB__subsample : 0.9	0.703538	0.763194
RF	SS	ADASYN	RF__class_weight : None; RF__criterion : gini; RF__max_depth : 2; RF__max_features : 1; RF__min_samples_leaf : 2; RF__n_estimators : 319; RF__random_state : 23	0.703418	0.764297
XGB	SS	ADASYN	XGB__colsample_bytree : 0.1; XGB__gamma : 1.594651861223503; XGB__max_depth : 1; XGB__min_child_weight : 0.0; XGB__random_state : 0; XGB__scale_pos_weight : 0.5; XGB__subsample : 0.5	0.703389	0.762422
RF	MM	ADASYN	RF__class_weight : None; RF__criterion : gini; RF__max_depth : 2; RF__max_features : 1; RF__min_samples_leaf : 2; RF__n_estimators : 1000; RF__random_state : 96	0.703265	0.762079
RF	MM	SMOTE	RF__class_weight : None; RF__criterion : gini; RF__max_depth : 2; RF__max_features : 9;	0.70326	0.750593

			RF__min_samples_leaf : 3; RF__n_estimators : 948; RF__random_state : 48		
RF	MM	ADASYN	RF__class_weight : balanced; RF__criterion : log_loss; RF__max_depth : 3; RF__max_features : 7; RF__min_samples_leaf : 1; RF__n_estimators : 825; RF__random_state : 34	0.703211	0.780729
RF	Not used	Not used	RF__class_weight : None; RF__criterion : gini; RF__max_depth : 3; RF__max_features : 1; RF__min_samples_leaf : 2; RF__n_estimators : 702; RF__random_state : 52	0.703204	0.801771
RF	MM	ROS	RF__class_weight : None; RF__criterion : gini; RF__max_depth : 4; RF__max_features : 1; RF__min_samples_leaf : 8; RF__n_estimators : 461; RF__random_state : 37	0.703101	0.802869
RF	Not used	Not used	RF__class_weight : None; RF__criterion : log_loss; RF__max_depth : 3; RF__max_features : 1; RF__min_samples_leaf : 3; RF__n_estimators : 300; RF__random_state : 100	0.703052	0.799255
RF	MM	SMOTE	RF__class_weight : None; RF__criterion : gini; RF__max_depth : 2; RF__max_features : 7; RF__min_samples_leaf : 1; RF__n_estimators : 1000; RF__random_state : 98	0.703002	0.753186
RF	MM	ADASYN	RF__class_weight : balanced; RF__criterion : gini; RF__max_depth : 3; RF__max_features : 6; RF__min_samples_leaf : 2; RF__n_estimators : 614; RF__random_state : 3	0.702955	0.784903
RF	MM	SMOTE	RF__class_weight : balanced; RF__criterion : log_loss; RF__max_depth : 2; RF__max_features : 6; RF__min_samples_leaf : 1; RF__n_estimators : 770; RF__random_state : 4	0.702951	0.748434

RF	Not used	Not used	RF__class_weight : balanced; RF__criterion : log_loss; RF__max_depth : 4; RF__max_features : 1; RF__min_samples_leaf : 2; RF__n_estimators : 519; RF__random_state : 67	0.702893	0.833733
RF	MM	SMOTE	RF__class_weight : None; RF__criterion : gini; RF__max_depth : 2; RF__max_features : 7; RF__min_samples_leaf : 1; RF__n_estimators : 1000; RF__random_state : 57	0.702812	0.753995
RF	SS	ADASYN	RF__class_weight : None; RF__criterion : log_loss; RF__max_depth : 3; RF__max_features : 1; RF__min_samples_leaf : 1; RF__n_estimators : 951; RF__random_state : 95	0.702794	0.790368
RF	Not used	Not used	RF__class_weight : None; RF__criterion : gini; RF__max_depth : 4; RF__max_features : 1; RF__min_samples_leaf : 2; RF__n_estimators : 300; RF__random_state : 100	0.702793	0.833302
RF	Not used	Not used	RF__class_weight : None; RF__criterion : log_loss; RF__max_depth : 2; RF__max_features : 1; RF__min_samples_leaf : 2; RF__n_estimators : 478; RF__random_state : 100	0.702724	0.774631
RF	MM	ADASYN	RF__class_weight : balanced_subsample; RF__criterion : gini; RF__max_depth : 3; RF__max_features : 6; RF__min_samples_leaf : 1; RF__n_estimators : 300; RF__random_state : 77	0.702571	0.788948
RF	Not used	Not used	RF__class_weight : balanced_subsample; RF__criterion : log_loss; RF__max_depth : 3; RF__max_features : 1; RF__min_samples_leaf : 3; RF__n_estimators : 580; RF__random_state : 100	0.702484	0.797556
RF	MM	ROS	RF__class_weight : None; RF__criterion : gini; RF__max_depth : 4; RF__max_features : 1;	0.702292	0.814823

			RF__min_samples_leaf : 5; RF__n_estimators : 1000; RF__random_state : 100		
RF	SS	ADASYN	RF__class_weight : None; RF__criterion : gini; RF__max_depth : 3; RF__max_features : 1; RF__min_samples_leaf : 2; RF__n_estimators : 300; RF__random_state : 0	0.702178	0.790969
RF	SS	ADASYN	RF__class_weight : balanced; RF__criterion : gini; RF__max_depth : 3; RF__max_features : 1; RF__min_samples_leaf : 1; RF__n_estimators : 322; RF__random_state : 2	0.70216	0.790411
XGB	SS	SMOTE	XGB__colsample_bytree : 1.0; XGB__gamma : 3.75538505665694; XGB__max_depth : 1; XGB__min_child_weight : 6.034763897106964; XGB__random_state : 0; XGB__scale_pos_weight : 3.500199914633356; XGB__subsample : 0.9	0.702126	0.765963
RF	MM	ADASYN	RF__class_weight : None; RF__criterion : gini; RF__max_depth : 3; RF__max_features : 1; RF__min_samples_leaf : 1; RF__n_estimators : 1000; RF__random_state : 99	0.702038	0.795098
RF	SS	ADASYN	RF__class_weight : None; RF__criterion : log_loss; RF__max_depth : 2; RF__max_features : 1; RF__min_samples_leaf : 2; RF__n_estimators : 1000; RF__random_state : 0	0.702023	0.761531
RF	MM	SMOTE	RF__class_weight : balanced_subsample; RF__criterion : gini; RF__max_depth : 2; RF__max_features : 9; RF__min_samples_leaf : 1; RF__n_estimators : 300; RF__random_state : 58	0.701952	0.750641
RF	SS	ADASYN	RF__class_weight : balanced; RF__criterion : log_loss; RF__max_depth : 4; RF__max_features : 6; RF__min_samples_leaf : 2; RF__n_estimators : 456; RF__random_state : 29	0.70192	0.819471

RF	MM	SMOTE	RF__class_weight : None; RF__criterion : gini; RF__max_depth : 2; RF__max_features : 7; RF__min_samples_leaf : 1; RF__n_estimators : 861; RF__random_state : 7	0.701832	0.751883
XGB	SS	SMOTE	XGB__colsample_bytree : 1.0; XGB__gamma : 4.296914398874771; XGB__max_depth : 1; XGB__min_child_weight : 8.913657735308098; XGB__random_state : 0; XGB__scale_pos_weight : 4.126980830609001; XGB__subsample : 0.9	0.701796	0.755303
RF	MM	SMOTE	RF__class_weight : balanced_subsample; RF__criterion : gini; RF__max_depth : 2; RF__max_features : 7; RF__min_samples_leaf : 1; RF__n_estimators : 1000; RF__random_state : 18	0.70171	0.752147
RF	SS	SMOTE	RF__class_weight : balanced; RF__criterion : log_loss; RF__max_depth : 2; RF__max_features : 1; RF__min_samples_leaf : 1; RF__n_estimators : 344; RF__random_state : 98	0.701708	0.762454
RF	SS	ADASYN	RF__class_weight : balanced_subsample; RF__criterion : log_loss; RF__max_depth : 4; RF__max_features : 7; RF__min_samples_leaf : 1; RF__n_estimators : 300; RF__random_state : 100	0.701699	0.8264
XGB	SS	ADASYN	XGB__colsample_bytree : 1.0; XGB__gamma : 4.241222837982958; XGB__max_depth : 1; XGB__min_child_weight : 0.0; XGB__random_state : 0; XGB__scale_pos_weight : 0.5; XGB__subsample : 0.5	0.701681	0.754004
RF	Not used	Not used	RF__class_weight : None; RF__criterion : log_loss; RF__max_depth : 4; RF__max_features : 1; RF__min_samples_leaf : 3; RF__n_estimators : 300; RF__random_state : 100	0.701484	0.826848

RF	MM	SMOTE	RF__class_weight : None; RF__criterion : gini; RF__max_depth : 2; RF__max_features : 3; RF__min_samples_leaf : 1; RF__n_estimators : 300; RF__random_state : 15	0.701471	0.75359
XGB	SS	SMOTE	XGB__colsample_bytree : 1.0; XGB__gamma : 4.678879243752123; XGB__max_depth : 1; XGB__min_child_weight : 8.899688149760228; XGB__random_state : 0; XGB__scale_pos_weight : 4.129583662828905; XGB__subsample : 0.9	0.70145	0.754521
RF	SS	ADASYN	RF__class_weight : None; RF__criterion : log_loss; RF__max_depth : 3; RF__max_features : 9; RF__min_samples_leaf : 1; RF__n_estimators : 365; RF__random_state : 98	0.701436	0.785229
RF	SS	ADASYN	RF__class_weight : None; RF__criterion : gini; RF__max_depth : 3; RF__max_features : 1; RF__min_samples_leaf : 1; RF__n_estimators : 1000; RF__random_state : 0	0.70135	0.791876
NN	SS	ROS	NN__activation : logistic; NN__alpha : 0.00010041313053825191; NN__hidden_layer_sizes : 50; NN__max_iter : 500; NN__random_state : 0; NN__solver : adam	0.701282	0.702622
RF	SS	ADASYN	RF__class_weight : balanced; RF__criterion : gini; RF__max_depth : 3; RF__max_features : 1; RF__min_samples_leaf : 2; RF__n_estimators : 934; RF__random_state : 82	0.70111	0.789951
RF	MM	ADASYN	RF__class_weight : balanced; RF__criterion : gini; RF__max_depth : 3; RF__max_features : 6; RF__min_samples_leaf : 4; RF__n_estimators : 760; RF__random_state : 1	0.701092	0.781822
XGB	SS	SMOTE	XGB__colsample_bytree : 1.0; XGB__gamma : 4.764505060102767; XGB__max_depth : 1;	0.701007	0.762859

			XGB__min_child_weight : 6.4262652449332105; XGB__random_state : 0; XGB__scale_pos_weight : 5.0; XGB__subsample : 0.9		
RF	MM	ADASYN	RF__class_weight : balanced; RF__criterion : gini; RF__max_depth : 3; RF__max_features : 7; RF__min_samples_leaf : 1; RF__n_estimators : 1000; RF__random_state : 43	0.700994	0.78866
XGB	SS	SMOTE	XGB__colsample_bytree : 1.0; XGB__gamma : 3.872504674892467; XGB__max_depth : 1; XGB__min_child_weight : 9.855428640015464; XGB__random_state : 0; XGB__scale_pos_weight : 5.0; XGB__subsample : 0.9	0.700986	0.75538
XGB	SS	ADASYN	XGB__colsample_bytree : 1.0; XGB__gamma : 4.7626566805308155; XGB__max_depth : 1; XGB__min_child_weight : 0.0; XGB__random_state : 0; XGB__scale_pos_weight : 0.5; XGB__subsample : 0.5	0.700892	0.742186
XGB	Not used	Not used	XGB__colsample_bytree : 0.4915354427214047; XGB__gamma : 5.265304807725755; XGB__max_depth : 2; XGB__min_child_weight : 0.0; XGB__random_state : 0; XGB__scale_pos_weight : 2.7840629929455236; XGB__subsample : 0.899342467100523	0.700844	0.806561
XGB	MM	SMOTE	XGB__colsample_bytree : 0.7551049404234403; XGB__gamma : 4.751256572397854; XGB__max_depth : 1; XGB__min_child_weight : 0.0; XGB__random_state : 0; XGB__scale_pos_weight : 3.827959148451575; XGB__subsample : 0.8414529617618633	0.700812	0.768316
RF	MM	ROS	RF__class_weight : balanced; RF__criterion : gini; RF__max_depth : 3; RF__max_features : 1;	0.700762	0.795797

			RF__min_samples_leaf : 2; RF__n_estimators : 896; RF__random_state : 5		
XGB	Not used	Not used	XGB__colsample_bytree : 1.0; XGB__gamma : 8.135617307096926; XGB__max_depth : 1; XGB__min_child_weight : 0.0; XGB__random_state : 0; XGB__scale_pos_weight : 3.1186247273806336; XGB__subsample : 0.9	0.700717	0.753496
RF	MM	ADASYN	RF__class_weight : None; RF__criterion : log_loss; RF__max_depth : 2; RF__max_features : 1; RF__min_samples_leaf : 1; RF__n_estimators : 820; RF__random_state : 28	0.700699	0.764065
RF	SS	SMOTE	RF__class_weight : None; RF__criterion : log_loss; RF__max_depth : 3; RF__max_features : 4; RF__min_samples_leaf : 1; RF__n_estimators : 304; RF__random_state : 17	0.700638	0.792976
RF	SS	ROS	RF__class_weight : balanced; RF__criterion : log_loss; RF__max_depth : 5; RF__max_features : 1; RF__min_samples_leaf : 4; RF__n_estimators : 558; RF__random_state : 100	0.700485	0.842837
XGB	SS	SMOTE	XGB__colsample_bytree : 1.0; XGB__gamma : 4.167097834109587; XGB__max_depth : 1; XGB__min_child_weight : 5.641797887723928; XGB__random_state : 0; XGB__scale_pos_weight : 3.6383028480816413; XGB__subsample : 0.9	0.700456	0.763653
RF	MM	ADASYN	RF__class_weight : balanced; RF__criterion : gini; RF__max_depth : 3; RF__max_features : 1; RF__min_samples_leaf : 2; RF__n_estimators : 310; RF__random_state : 81	0.700379	0.793482
XGB	SS	ADASYN	XGB__colsample_bytree : 1.0; XGB__gamma : 4.276909823793428; XGB__max_depth : 1; XGB__min_child_weight : 0.0; XGB__random_state :	0.700308	0.75425

			0; XGB__scale_pos_weight : 0.5; XGB__subsample : 0.5		
NN	SS	ROS	NN__activation : logistic; NN__alpha : 10.0; NN__hidden_layer_sizes : 50; NN__max_iter : 438; NN__random_state : 0; NN__solver : adam	0.700284	0.701622
XGB	SS	SMOTE	XGB__colsample_bytree : 1.0; XGB__gamma : 5.297030762745017; XGB__max_depth : 1; XGB__min_child_weight : 4.878728139125758; XGB__random_state : 0; XGB__scale_pos_weight : 3.5496428352971146; XGB__subsample : 0.9	0.700266	0.759412
NN	SS	ADASYN	NN__activation : logistic; NN__alpha : 0.0001; NN__hidden_layer_sizes : 50; NN__max_iter : 50; NN__random_state : 0; NN__solver : adam	0.700254	0.702179
RF	MM	ADASYN	RF__class_weight : balanced_subsample; RF__criterion : gini; RF__max_depth : 3; RF__max_features : 4; RF__min_samples_leaf : 3; RF__n_estimators : 300; RF__random_state : 100	0.700248	0.783931
RF	MM	SMOTE	RF__class_weight : None; RF__criterion : log_loss; RF__max_depth : 2; RF__max_features : 5; RF__min_samples_leaf : 1; RF__n_estimators : 811; RF__random_state : 99	0.700231	0.748211
XGB	Not used	Not used	XGB__colsample_bytree : 1.0; XGB__gamma : 5.495324270671143; XGB__max_depth : 1; XGB__min_child_weight : 0.0; XGB__random_state : 0; XGB__scale_pos_weight : 2.9836216372515407; XGB__subsample : 0.6275159750982203	0.700056	0.779497
RF	MM	ROS	RF__class_weight : None; RF__criterion : gini; RF__max_depth : 3; RF__max_features : 1; RF__min_samples_leaf : 3; RF__n_estimators : 947; RF__random_state : 100	0.699749	0.794401

RF	MM	ROS	RF__class_weight : None; RF__criterion : gini; RF__max_depth : 3; RF__max_features : 1; RF__min_samples_leaf : 8; RF__n_estimators : 932; RF__random_state : 63	0.699746	0.785585
XGB	SS	SMOTE	XGB__colsample_bytree : 1.0; XGB__gamma : 4.754108910429519; XGB__max_depth : 1; XGB__min_child_weight : 7.676454933519953; XGB__random_state : 0; XGB__scale_pos_weight : 4.140827317710019; XGB__subsample : 0.9	0.699702	0.757717
RF	MM	ROS	RF__class_weight : balanced; RF__criterion : gini; RF__max_depth : 4; RF__max_features : 1; RF__min_samples_leaf : 6; RF__n_estimators : 1000; RF__random_state : 100	0.699657	0.810428
RF	MM	ROS	RF__class_weight : balanced; RF__criterion : gini; RF__max_depth : 2; RF__max_features : 1; RF__min_samples_leaf : 1; RF__n_estimators : 1000; RF__random_state : 100	0.699611	0.766318
RF	MM	ADASYN	RF__class_weight : balanced; RF__criterion : gini; RF__max_depth : 3; RF__max_features : 1; RF__min_samples_leaf : 5; RF__n_estimators : 1000; RF__random_state : 26	0.699608	0.787001
RF	MM	ADASYN	RF__class_weight : balanced; RF__criterion : log_loss; RF__max_depth : 3; RF__max_features : 4; RF__min_samples_leaf : 4; RF__n_estimators : 300; RF__random_state : 100	0.699574	0.778067
XGB	SS	SMOTE	XGB__colsample_bytree : 1.0; XGB__gamma : 4.140677302376605; XGB__max_depth : 1; XGB__min_child_weight : 10.50933690138394; XGB__random_state : 0; XGB__scale_pos_weight : 5.0; XGB__subsample : 0.9	0.699451	0.754241

RF	MM	ADASYN	RF__class_weight : None; RF__criterion : log_loss; RF__max_depth : 3; RF__max_features : 9; RF__min_samples_leaf : 1; RF__n_estimators : 318; RF__random_state : 76	0.699403	0.781129
RF	MM	ROS	RF__class_weight : None; RF__criterion : gini; RF__max_depth : 4; RF__max_features : 1; RF__min_samples_leaf : 5; RF__n_estimators : 368; RF__random_state : 100	0.699246	0.814645
RF	SS	ROS	RF__class_weight : balanced; RF__criterion : gini; RF__max_depth : 2; RF__max_features : 1; RF__min_samples_leaf : 11; RF__n_estimators : 705; RF__random_state : 100	0.699218	0.75913
XGB	SS	SMOTE	XGB__colsample_bytree : 1.0; XGB__gamma : 5.128228593675832; XGB__max_depth : 1; XGB__min_child_weight : 5.871496692006498; XGB__random_state : 0; XGB__scale_pos_weight : 5.0; XGB__subsample : 0.9	0.699044	0.761583
RF	SS	ROS	RF__class_weight : None; RF__criterion : gini; RF__max_depth : 4; RF__max_features : 1; RF__min_samples_leaf : 5; RF__n_estimators : 361; RF__random_state : 88	0.698901	0.816177
XGB	SS	ADASYN	XGB__colsample_bytree : 0.1; XGB__gamma : 2.631648171634598; XGB__max_depth : 1; XGB__min_child_weight : 0.0; XGB__random_state : 0; XGB__scale_pos_weight : 0.5; XGB__subsample : 0.5	0.698784	0.753103
RF	SS	SMOTE	RF__class_weight : balanced_subsample; RF__criterion : log_loss; RF__max_depth : 3; RF__max_features : 1; RF__min_samples_leaf : 1; RF__n_estimators : 898; RF__random_state : 97	0.698764	0.788998

RF	SS	ROS	RF__class_weight : balanced_subsample; RF__criterion : gini; RF__max_depth : 5; RF__max_features : 1; RF__min_samples_leaf : 3; RF__n_estimators : 332; RF__random_state : 100	0.698693	0.851723
XGB	Not used	Not used	XGB__colsample_bytree : 1.0; XGB__gamma : 12.522484455388685; XGB__max_depth : 2; XGB__min_child_weight : 0.0; XGB__random_state : 0; XGB__scale_pos_weight : 3.7022442003311538; XGB__subsample : 0.9	0.698424	0.777833
RF	SS	ROS	RF__class_weight : balanced; RF__criterion : gini; RF__max_depth : 4; RF__max_features : 1; RF__min_samples_leaf : 1; RF__n_estimators : 300; RF__random_state : 100	0.698214	0.835166
RF	SS	SMOTE	RF__class_weight : None; RF__criterion : log_loss; RF__max_depth : 4; RF__max_features : 1; RF__min_samples_leaf : 3; RF__n_estimators : 708; RF__random_state : 98	0.698193	0.813255
RF	SS	ROS	RF__class_weight : balanced; RF__criterion : log_loss; RF__max_depth : 2; RF__max_features : 1; RF__min_samples_leaf : 8; RF__n_estimators : 581; RF__random_state : 100	0.698149	0.762047
XGB	SS	SMOTE	XGB__colsample_bytree : 1.0; XGB__gamma : 4.302195515165045; XGB__max_depth : 1; XGB__min_child_weight : 8.371699143821461; XGB__random_state : 0; XGB__scale_pos_weight : 5.0; XGB__subsample : 0.9	0.69805	0.759473
RF	MM	ROS	RF__class_weight : balanced; RF__criterion : gini; RF__max_depth : 2; RF__max_features : 1; RF__min_samples_leaf : 2; RF__n_estimators : 574; RF__random_state : 41	0.698045	0.765719

RF	SS	ROS	RF__class_weight : None; RF__criterion : gini; RF__max_depth : 5; RF__max_features : 1; RF__min_samples_leaf : 1; RF__n_estimators : 546; RF__random_state : 100	0.697955	0.879275
XGB	MM	SMOTE	XGB__colsample_bytree : 0.6747884605318322; XGB__gamma : 6.977039154012745; XGB__max_depth : 1; XGB__min_child_weight : 0.0; XGB__random_state : 0; XGB__scale_pos_weight : 3.9410278120128917; XGB__subsample : 0.8408644785816788	0.697857	0.757732
RF	MM	ROS	RF__class_weight : None; RF__criterion : gini; RF__max_depth : 4; RF__max_features : 1; RF__min_samples_leaf : 5; RF__n_estimators : 300; RF__random_state : 100	0.697817	0.814001
XGB	MM	SMOTE	XGB__colsample_bytree : 0.630740395791118; XGB__gamma : 6.006071201245516; XGB__max_depth : 1; XGB__min_child_weight : 0.0; XGB__random_state : 0; XGB__scale_pos_weight : 3.9981378323146455; XGB__subsample : 0.9	0.69774	0.762282
RF	SS	ROS	RF__class_weight : balanced; RF__criterion : gini; RF__max_depth : 4; RF__max_features : 1; RF__min_samples_leaf : 2; RF__n_estimators : 536; RF__random_state : 100	0.69768	0.830106
NN	SS	SMOTE	NN__activation : logistic; NN__alpha : 0.007306211783268143; NN__hidden_layer_sizes : 30; NN__max_iter : 50; NN__random_state : 0; NN__solver : adam	0.697553	0.69465
XGB	SS	ADASYN	XGB__colsample_bytree : 0.1; XGB__gamma : 0.01; XGB__max_depth : 1; XGB__min_child_weight : 0.0; XGB__random_state : 0; XGB__scale_pos_weight : 2.4862644924154425; XGB__subsample : 0.9	0.697392	0.777013

RF	MM	ROS	RF__class_weight : balanced; RF__criterion : gini; RF__max_depth : 3; RF__max_features : 1; RF__min_samples_leaf : 2; RF__n_estimators : 300; RF__random_state : 0	0.697371	0.796431
XGB	SS	SMOTE	XGB__colsample_bytree : 0.9520694817560885; XGB__gamma : 4.731161553420115; XGB__max_depth : 1; XGB__min_child_weight : 7.880539662378007; XGB__random_state : 0; XGB__scale_pos_weight : 3.4185982274891034; XGB__subsample : 0.9	0.697336	0.75301
NN	SS	ROS	NN__activation : logistic; NN__alpha : 0.0001; NN__hidden_layer_sizes : 3; NN__max_iter : 455; NN__random_state : 0; NN__solver : adam	0.697306	0.697452
RF	MM	ROS	RF__class_weight : balanced; RF__criterion : gini; RF__max_depth : 4; RF__max_features : 1; RF__min_samples_leaf : 6; RF__n_estimators : 1000; RF__random_state : 72	0.697301	0.810728
RF	SS	ROS	RF__class_weight : balanced; RF__criterion : gini; RF__max_depth : 2; RF__max_features : 1; RF__min_samples_leaf : 3; RF__n_estimators : 300; RF__random_state : 100	0.697286	0.764093
RF	SS	SMOTE	RF__class_weight : balanced_subsample; RF__criterion : gini; RF__max_depth : 1; RF__max_features : 1; RF__min_samples_leaf : 5; RF__n_estimators : 455; RF__random_state : 100	0.697177	0.733618
RF	MM	ROS	RF__class_weight : balanced; RF__criterion : gini; RF__max_depth : 4; RF__max_features : 1; RF__min_samples_leaf : 4; RF__n_estimators : 398; RF__random_state : 94	0.697175	0.818449
RF	MM	ROS	RF__class_weight : None; RF__criterion : log_loss; RF__max_depth : 5; RF__max_features : 1;	0.697129	0.827762

			RF__min_samples_leaf : 6; RF__n_estimators : 311; RF__random_state : 62		
XGB	SS	SMOTE	XGB__colsample_bytree : 0.1; XGB__gamma : 4.164451665860661; XGB__max_depth : 1; XGB__min_child_weight : 8.996899106010986; XGB__random_state : 0; XGB__scale_pos_weight : 5.0; XGB__subsample : 0.9	0.697093	0.75016
RF	MM	ROS	RF__class_weight : None; RF__criterion : gini; RF__max_depth : 4; RF__max_features : 1; RF__min_samples_leaf : 5; RF__n_estimators : 1000; RF__random_state : 0	0.696955	0.814484
NN	SS	ADASYN	NN__activation : tanh; NN__alpha : 0.004562925820006588; NN__hidden_layer_sizes : 5; NN__max_iter : 500; NN__random_state : 0; NN__solver : adam	0.696909	0.701705
RF	MM	ROS	RF__class_weight : None; RF__criterion : log_loss; RF__max_depth : 4; RF__max_features : 1; RF__min_samples_leaf : 3; RF__n_estimators : 875; RF__random_state : 55	0.696906	0.823291
RF	SS	SMOTE	RF__class_weight : None; RF__criterion : log_loss; RF__max_depth : 2; RF__max_features : 3; RF__min_samples_leaf : 1; RF__n_estimators : 984; RF__random_state : 4	0.696837	0.751912
XGB	MM	SMOTE	XGB__colsample_bytree : 0.7029459141756554; XGB__gamma : 7.604446730553565; XGB__max_depth : 1; XGB__min_child_weight : 0.0; XGB__random_state : 0; XGB__scale_pos_weight : 3.990282070957032; XGB__subsample : 0.8357335463942575	0.696805	0.754824
RF	SS	ROS	RF__class_weight : balanced; RF__criterion : gini; RF__max_depth : 5; RF__max_features : 1;	0.696783	0.83496

			RF__min_samples_leaf : 5; RF__n_estimators : 803; RF__random_state : 49		
RF	SS	SMOTE	RF__class_weight : balanced_subsample; RF__criterion : log_loss; RF__max_depth : 3; RF__max_features : 1; RF__min_samples_leaf : 5; RF__n_estimators : 336; RF__random_state : 99	0.696732	0.781132
RF	MM	ROS	RF__class_weight : balanced; RF__criterion : gini; RF__max_depth : 5; RF__max_features : 1; RF__min_samples_leaf : 5; RF__n_estimators : 887; RF__random_state : 82	0.696697	0.834033
RF	SS	ROS	RF__class_weight : balanced; RF__criterion : gini; RF__max_depth : 5; RF__max_features : 1; RF__min_samples_leaf : 4; RF__n_estimators : 822; RF__random_state : 100	0.696665	0.842927
RF	MM	ROS	RF__class_weight : None; RF__criterion : gini; RF__max_depth : 4; RF__max_features : 1; RF__min_samples_leaf : 1; RF__n_estimators : 985; RF__random_state : 95	0.696663	0.836398
XGB	MM	ROS	XGB__colsample_bytree : 1.0; XGB__gamma : 12.983876280799059; XGB__max_depth : 1; XGB__min_child_weight : 6.613493800394396; XGB__random_state : 0; XGB__scale_pos_weight : 4.2934687732688985; XGB__subsample : 0.7672320783527744	0.696504	0.739695
RF	SS	ROS	RF__class_weight : None; RF__criterion : gini; RF__max_depth : 5; RF__max_features : 1; RF__min_samples_leaf : 1; RF__n_estimators : 400; RF__random_state : 100	0.696371	0.879646
RF	MM	ROS	RF__class_weight : None; RF__criterion : gini; RF__max_depth : 3; RF__max_features : 1;	0.696251	0.795744

			RF__min_samples_leaf : 1; RF__n_estimators : 331; RF__random_state : 96		
RF	SS	ROS	RF__class_weight : balanced; RF__criterion : log_loss; RF__max_depth : 3; RF__max_features : 1; RF__min_samples_leaf : 3; RF__n_estimators : 1000; RF__random_state : 100	0.696235	0.794066
RF	SS	ROS	RF__class_weight : balanced; RF__criterion : log_loss; RF__max_depth : 2; RF__max_features : 6; RF__min_samples_leaf : 1; RF__n_estimators : 1000; RF__random_state : 100	0.696233	0.767553
RF	SS	ROS	RF__class_weight : balanced; RF__criterion : log_loss; RF__max_depth : 3; RF__max_features : 1; RF__min_samples_leaf : 1; RF__n_estimators : 1000; RF__random_state : 100	0.696166	0.7978
RF	MM	ROS	RF__class_weight : None; RF__criterion : gini; RF__max_depth : 4; RF__max_features : 1; RF__min_samples_leaf : 5; RF__n_estimators : 1000; RF__random_state : 79	0.696165	0.81422
RF	MM	ROS	RF__class_weight : None; RF__criterion : gini; RF__max_depth : 4; RF__max_features : 1; RF__min_samples_leaf : 6; RF__n_estimators : 300; RF__random_state : 0	0.696112	0.809856
RF	SS	ROS	RF__class_weight : balanced_subsample; RF__criterion : log_loss; RF__max_depth : 5; RF__max_features : 1; RF__min_samples_leaf : 3; RF__n_estimators : 632; RF__random_state : 0	0.696078	0.851923
RF	SS	SMOTE	RF__class_weight : None; RF__criterion : log_loss; RF__max_depth : 3; RF__max_features : 4; RF__min_samples_leaf : 2; RF__n_estimators : 991; RF__random_state : 14	0.696061	0.79166

NN	SS	ROS	NN__activation : logistic; NN__alpha : 0.43513970791520495; NN__hidden_layer_sizes : 40; NN__max_iter : 192; NN__random_state : 0; NN__solver : adam	0.696048	0.701765
XGB	Not used	Not used	XGB__colsample_bytree : 1.0; XGB__gamma : 13.047166214482912; XGB__max_depth : 1; XGB__min_child_weight : 0.0; XGB__random_state : 0; XGB__scale_pos_weight : 3.159704766400389; XGB__subsample : 0.6305878164966068	0.695992	0.738306
XGB	SS	SMOTE	XGB__colsample_bytree : 0.5739866723148325; XGB__gamma : 3.679309787330441; XGB__max_depth : 1; XGB__min_child_weight : 7.933127779169759; XGB__random_state : 0; XGB__scale_pos_weight : 5.0; XGB__subsample : 0.9	0.695985	0.76025
XGB	SS	ROS	XGB__colsample_bytree : 0.1; XGB__gamma : 0.01; XGB__max_depth : 1; XGB__min_child_weight : 0.0; XGB__random_state : 0; XGB__scale_pos_weight : 2.760551823960115; XGB__subsample : 0.5	0.695968	0.779879
XGB	SS	SMOTE	XGB__colsample_bytree : 1.0; XGB__gamma : 3.8635825153568053; XGB__max_depth : 1; XGB__min_child_weight : 6.047204046742893; XGB__random_state : 0; XGB__scale_pos_weight : 3.5030253516598058; XGB__subsample : 0.9	0.695928	0.765597
RF	SS	ROS	RF__class_weight : balanced_subsample; RF__criterion : log_loss; RF__max_depth : 2; RF__max_features : 6; RF__min_samples_leaf : 2; RF__n_estimators : 300; RF__random_state : 100	0.695846	0.766436
XGB	Not used	Not used	XGB__colsample_bytree : 1.0; XGB__gamma : 7.901615368030613; XGB__max_depth : 1; XGB__min_child_weight : 0.0; XGB__random_state :	0.695793	0.755961

			0; XGB__scale_pos_weight : 3.9680884627856092; XGB__subsample : 0.9		
RF	SS	ROS	RF__class_weight : None; RF__criterion : log_loss; RF__max_depth : 2; RF__max_features : 1; RF__min_samples_leaf : 1; RF__n_estimators : 321; RF__random_state : 100	0.69572	0.765099
RF	SS	ROS	RF__class_weight : balanced; RF__criterion : log_loss; RF__max_depth : 5; RF__max_features : 1; RF__min_samples_leaf : 2; RF__n_estimators : 443; RF__random_state : 100	0.695714	0.863717
XGB	MM	SMOTE	XGB__colsample_bytree : 0.6504103845092375; XGB__gamma : 5.976130305505102; XGB__max_depth : 1; XGB__min_child_weight : 0.0; XGB__random_state : 0; XGB__scale_pos_weight : 3.915482202159969; XGB__subsample : 0.9	0.695673	0.761826
RF	SS	ROS	RF__class_weight : balanced; RF__criterion : log_loss; RF__max_depth : 5; RF__max_features : 1; RF__min_samples_leaf : 1; RF__n_estimators : 1000; RF__random_state : 100	0.695596	0.877031
RF	SS	SMOTE	RF__class_weight : None; RF__criterion : log_loss; RF__max_depth : 2; RF__max_features : 1; RF__min_samples_leaf : 2; RF__n_estimators : 927; RF__random_state : 51	0.695563	0.758526
XGB	SS	SMOTE	XGB__colsample_bytree : 1.0; XGB__gamma : 6.10047413137733; XGB__max_depth : 1; XGB__min_child_weight : 0.0; XGB__random_state : 0; XGB__scale_pos_weight : 3.6875534884544467; XGB__subsample : 0.9	0.695558	0.76123
RF	MM	ROS	RF__class_weight : balanced_subsample; RF__criterion : gini; RF__max_depth : 3;	0.695441	0.787449

			RF__max_features : 1; RF__min_samples_leaf : 6; RF__n_estimators : 1000; RF__random_state : 0		
RF	SS	ROS	RF__class_weight : balanced; RF__criterion : log_loss; RF__max_depth : 1; RF__max_features : 1; RF__min_samples_leaf : 5; RF__n_estimators : 300; RF__random_state : 90	0.695317	0.737899
XGB	SS	SMOTE	XGB__colsample_bytree : 1.0; XGB__gamma : 10.134857613880865; XGB__max_depth : 1; XGB__min_child_weight : 0.0; XGB__random_state : 0; XGB__scale_pos_weight : 4.067226597671307; XGB__subsample : 0.9	0.695216	0.742971
XGB	SS	SMOTE	XGB__colsample_bytree : 1.0; XGB__gamma : 5.131335783616919; XGB__max_depth : 1; XGB__min_child_weight : 6.199819563667402; XGB__random_state : 0; XGB__scale_pos_weight : 5.0; XGB__subsample : 0.9	0.695187	0.761365
RF	SS	ROS	RF__class_weight : balanced; RF__criterion : gini; RF__max_depth : 1; RF__max_features : 1; RF__min_samples_leaf : 14; RF__n_estimators : 308; RF__random_state : 100	0.695147	0.734235
XGB	MM	SMOTE	XGB__colsample_bytree : 0.5757431420985275; XGB__gamma : 5.950034688430638; XGB__max_depth : 1; XGB__min_child_weight : 0.0; XGB__random_state : 0; XGB__scale_pos_weight : 3.978507500949876; XGB__subsample : 0.8377619703535413	0.695141	0.761319
RF	MM	ROS	RF__class_weight : balanced_subsample; RF__criterion : gini; RF__max_depth : 2; RF__max_features : 7; RF__min_samples_leaf : 2; RF__n_estimators : 789; RF__random_state : 100	0.69513	0.767552

RF	SS	ROS	RF__class_weight : None; RF__criterion : log_loss; RF__max_depth : 2; RF__max_features : 1; RF__min_samples_leaf : 4; RF__n_estimators : 849; RF__random_state : 100	0.6951	0.765936
XGB	MM	SMOTE	XGB__colsample_bytree : 0.6336730229580582; XGB__gamma : 5.773927742955594; XGB__max_depth : 1; XGB__min_child_weight : 0.0; XGB__random_state : 0; XGB__scale_pos_weight : 3.909451949160928; XGB__subsample : 0.9	0.69505	0.763197
XGB	SS	ROS	XGB__colsample_bytree : 0.1; XGB__gamma : 0.01; XGB__max_depth : 1; XGB__min_child_weight : 0.0; XGB__random_state : 0; XGB__scale_pos_weight : 2.054272846361842; XGB__subsample : 0.5	0.694987	0.78078
RF	SS	ROS	RF__class_weight : balanced; RF__criterion : log_loss; RF__max_depth : 1; RF__max_features : 1; RF__min_samples_leaf : 10; RF__n_estimators : 309; RF__random_state : 100	0.694958	0.73476
NN	SS	ROS	NN__activation : tanh; NN__alpha : 2.6096146808538574; NN__hidden_layer_sizes : 8; NN__max_iter : 478; NN__random_state : 0; NN__solver : adam	0.694927	0.709437
RF	SS	SMOTE	RF__class_weight : balanced; RF__criterion : gini; RF__max_depth : 3; RF__max_features : 1; RF__min_samples_leaf : 1; RF__n_estimators : 300; RF__random_state : 0	0.694907	0.789781
XGB	MM	ROS	XGB__colsample_bytree : 1.0; XGB__gamma : 13.967662174349467; XGB__max_depth : 1; XGB__min_child_weight : 10.854940935747983; XGB__random_state : 0; XGB__scale_pos_weight : 4.957277013256746; XGB__subsample : 0.7470605098509118	0.694751	0.735803

RF	SS	ROS	RF__class_weight : None; RF__criterion : gini; RF__max_depth : 5; RF__max_features : 1; RF__min_samples_leaf : 1; RF__n_estimators : 620; RF__random_state : 73	0.694733	0.879299
RF	SS	ROS	RF__class_weight : balanced; RF__criterion : log_loss; RF__max_depth : 5; RF__max_features : 1; RF__min_samples_leaf : 3; RF__n_estimators : 300; RF__random_state : 100	0.694717	0.852378
RF	SS	ROS	RF__class_weight : None; RF__criterion : gini; RF__max_depth : 5; RF__max_features : 1; RF__min_samples_leaf : 6; RF__n_estimators : 456; RF__random_state : 2	0.694699	0.826845
RF	SS	ROS	RF__class_weight : balanced; RF__criterion : log_loss; RF__max_depth : 1; RF__max_features : 1; RF__min_samples_leaf : 2; RF__n_estimators : 300; RF__random_state : 100	0.694613	0.734183
XGB	SS	ROS	XGB__colsample_bytree : 0.1; XGB__gamma : 0.01; XGB__max_depth : 1; XGB__min_child_weight : 0.0; XGB__random_state : 0; XGB__scale_pos_weight : 2.0744553052420187; XGB__subsample : 0.5	0.694608	0.780848
XGB	Not used	Not used	XGB__colsample_bytree : 1.0; XGB__gamma : 8.952798715710603; XGB__max_depth : 1; XGB__min_child_weight : 0.0; XGB__random_state : 0; XGB__scale_pos_weight : 4.002287695828253; XGB__subsample : 0.9	0.694604	0.753177
XGB	Not used	Not used	XGB__colsample_bytree : 1.0; XGB__gamma : 9.05955711809137; XGB__max_depth : 1; XGB__min_child_weight : 0.0; XGB__random_state : 0; XGB__scale_pos_weight : 4.053478835955055; XGB__subsample : 0.9	0.694585	0.753032

RF	MM	ROS	RF__class_weight : balanced_subsample; RF__criterion : log_loss; RF__max_depth : 1; RF__max_features : 1; RF__min_samples_leaf : 20; RF__n_estimators : 481; RF__random_state : 100	0.694547	0.733328
RF	MM	ROS	RF__class_weight : balanced_subsample; RF__criterion : log_loss; RF__max_depth : 2; RF__max_features : 1; RF__min_samples_leaf : 3; RF__n_estimators : 596; RF__random_state : 99	0.69453	0.763649
XGB	SS	ADASYN	XGB__colsample_bytree : 0.1; XGB__gamma : 4.061834030884504; XGB__max_depth : 1; XGB__min_child_weight : 0.0; XGB__random_state : 0; XGB__scale_pos_weight : 3.3414294102558997; XGB__subsample : 0.9	0.694389	0.766401
NN	SS	ADASYN	NN__activation : tanh; NN__alpha : 0.0014850323682057333; NN__hidden_layer_sizes : 20; NN__max_iter : 50; NN__random_state : 0; NN__solver : adam	0.694382	0.702941
XGB	SS	SMOTE	XGB__colsample_bytree : 1.0; XGB__gamma : 3.7499656581046463; XGB__max_depth : 1; XGB__min_child_weight : 7.73628773092814; XGB__random_state : 0; XGB__scale_pos_weight : 5.0; XGB__subsample : 0.9	0.694341	0.763244
RF	SS	ROS	RF__class_weight : None; RF__criterion : gini; RF__max_depth : 3; RF__max_features : 1; RF__min_samples_leaf : 10; RF__n_estimators : 614; RF__random_state : 100	0.694339	0.779725
RF	SS	ROS	RF__class_weight : balanced; RF__criterion : log_loss; RF__max_depth : 5; RF__max_features : 2; RF__min_samples_leaf : 1; RF__n_estimators : 336; RF__random_state : 100	0.694218	0.880265

XGB	SS	ROS	XGB__colsample_bytree : 0.1; XGB__gamma : 0.01; XGB__max_depth : 1; XGB__min_child_weight : 0.0; XGB__random_state : 0; XGB__scale_pos_weight : 1.7841648265068442; XGB__subsample : 0.5	0.694177	0.78034
XGB	MM	SMOTE	XGB__colsample_bytree : 0.7024895240402017; XGB__gamma : 7.474466176373593; XGB__max_depth : 1; XGB__min_child_weight : 0.0; XGB__random_state : 0; XGB__scale_pos_weight : 3.995593708708675; XGB__subsample : 0.8346605838640351	0.694153	0.754659
RF	SS	SMOTE	RF__class_weight : None; RF__criterion : log_loss; RF__max_depth : 2; RF__max_features : 1; RF__min_samples_leaf : 7; RF__n_estimators : 318; RF__random_state : 99	0.694131	0.755936
XGB	SS	ADASYN	XGB__colsample_bytree : 0.1; XGB__gamma : 0.01; XGB__max_depth : 1; XGB__min_child_weight : 0.0; XGB__random_state : 0; XGB__scale_pos_weight : 3.0546364663464303; XGB__subsample : 0.9	0.694112	0.776887
RF	SS	SMOTE	RF__class_weight : balanced; RF__criterion : gini; RF__max_depth : 2; RF__max_features : 2; RF__min_samples_leaf : 3; RF__n_estimators : 1000; RF__random_state : 30	0.694046	0.755992
NN	SS	ROS	NN__activation : tanh; NN__alpha : 0.3184983766348907; NN__hidden_layer_sizes : 35; NN__max_iter : 237; NN__random_state : 0; NN__solver : adam	0.694046	0.723394
RF	MM	ROS	RF__class_weight : balanced; RF__criterion : log_loss; RF__max_depth : 3; RF__max_features : 1; RF__min_samples_leaf : 1; RF__n_estimators : 867; RF__random_state : 64	0.693997	0.797754

NN	SS	ROS	NN__activation : identity; NN__alpha : 10.0; NN__hidden_layer_sizes : 15; NN__max_iter : 500; NN__random_state : 0; NN__solver : adam	0.693962	0.702487
RF	MM	ROS	RF__class_weight : balanced; RF__criterion : gini; RF__max_depth : 4; RF__max_features : 1; RF__min_samples_leaf : 2; RF__n_estimators : 776; RF__random_state : 0	0.693961	0.829423
RF	MM	ROS	RF__class_weight : balanced; RF__criterion : gini; RF__max_depth : 2; RF__max_features : 5; RF__min_samples_leaf : 1; RF__n_estimators : 361; RF__random_state : 61	0.693883	0.769026
XGB	MM	SMOTE	XGB__colsample_bytree : 0.7984402541532296; XGB__gamma : 7.360158568012926; XGB__max_depth : 1; XGB__min_child_weight : 0.0; XGB__random_state : 0; XGB__scale_pos_weight : 4.080490693638323; XGB__subsample : 0.8525057658872475	0.693845	0.755605
NN	SS	SMOTE	NN__activation : tanh; NN__alpha : 0.14833592925889552; NN__hidden_layer_sizes : 3; NN__max_iter : 500; NN__random_state : 0; NN__solver : adam	0.693639	0.720996
NN	SS	ROS	NN__activation : logistic; NN__alpha : 10.0; NN__hidden_layer_sizes : 50; NN__max_iter : 496; NN__random_state : 0; NN__solver : lbfgs	0.693636	0.70179
RF	MM	ROS	RF__class_weight : None; RF__criterion : gini; RF__max_depth : 2; RF__max_features : 6; RF__min_samples_leaf : 2; RF__n_estimators : 338; RF__random_state : 5	0.693557	0.769197
XGB	Not used	Not used	XGB__colsample_bytree : 0.5238431776133157; XGB__gamma : 3.792195455030537; XGB__max_depth : 2; XGB__min_child_weight : 0.0;	0.693451	0.828518

			XGB__random_state : 0; XGB__scale_pos_weight : 3.6232357072738735; XGB__subsample : 0.9		
XGB	MM	SMOTE	XGB__colsample_bytree : 0.590382816088513; XGB__gamma : 4.834587218228065; XGB__max_depth : 1; XGB__min_child_weight : 0.0; XGB__random_state : 0; XGB__scale_pos_weight : 3.7581709378652435; XGB__subsample : 0.9	0.693324	0.766447
XGB	MM	ADASYN	XGB__colsample_bytree : 1.0; XGB__gamma : 5.249346010787132; XGB__max_depth : 1; XGB__min_child_weight : 0.0; XGB__random_state : 0; XGB__scale_pos_weight : 0.5; XGB__subsample : 0.6674005885827915	0.693301	0.742103
XGB	SS	ROS	XGB__colsample_bytree : 0.125993416307355; XGB__gamma : 0.01; XGB__max_depth : 1; XGB__min_child_weight : 5.811187786708272; XGB__random_state : 0; XGB__scale_pos_weight : 2.386588510611061; XGB__subsample : 0.9	0.693263	0.778056
RF	MM	ROS	RF__class_weight : balanced; RF__criterion : gini; RF__max_depth : 5; RF__max_features : 1; RF__min_samples_leaf : 2; RF__n_estimators : 552; RF__random_state : 96	0.69322	0.862738
NN	SS	ADASYN	NN__activation : logistic; NN__alpha : 0.0041311729337456336; NN__hidden_layer_sizes : 40; NN__max_iter : 486; NN__random_state : 0; NN__solver : adam	0.693087	0.703006
XGB	MM	SMOTE	XGB__colsample_bytree : 0.7294082371469425; XGB__gamma : 7.992507664600049; XGB__max_depth : 1; XGB__min_child_weight : 0.0; XGB__random_state : 0; XGB__scale_pos_weight : 4.026275056798889; XGB__subsample : 0.8322597911320617	0.693038	0.750942

XGB	MM	SMOTE	XGB__colsample_bytree : 0.5376547986680752; XGB__gamma : 5.484601988755668; XGB__max_depth : 1; XGB__min_child_weight : 0.0; XGB__random_state : 0; XGB__scale_pos_weight : 3.784428584534968; XGB__subsample : 0.832856094938742	0.69296	0.764919
XGB	SS	ADASYN	XGB__colsample_bytree : 0.1; XGB__gamma : 0.01; XGB__max_depth : 1; XGB__min_child_weight : 0.0; XGB__random_state : 0; XGB__scale_pos_weight : 1.8950650474636528; XGB__subsample : 0.9	0.692951	0.777582
XGB	Not used	Not used	XGB__colsample_bytree : 0.9506538846584873; XGB__gamma : 12.831511260940486; XGB__max_depth : 1; XGB__min_child_weight : 0.0; XGB__random_state : 0; XGB__scale_pos_weight : 5.0; XGB__subsample : 0.7348957497769235	0.692951	0.7499
XGB	SS	ADASYN	XGB__colsample_bytree : 1.0; XGB__gamma : 5.759911271557475; XGB__max_depth : 1; XGB__min_child_weight : 0.0; XGB__random_state : 0; XGB__scale_pos_weight : 0.5; XGB__subsample : 0.9	0.692945	0.733264
XGB	MM	SMOTE	XGB__colsample_bytree : 1.0; XGB__gamma : 2.9716748876704724; XGB__max_depth : 1; XGB__min_child_weight : 0.0; XGB__random_state : 0; XGB__scale_pos_weight : 3.6655622002485915; XGB__subsample : 0.9	0.692914	0.776671
NN	SS	SMOTE	NN__activation : logistic; NN__alpha : 0.00021228965539871224; NN__hidden_layer_sizes : 27; NN__max_iter : 50; NN__random_state : 0; NN__solver : adam	0.692911	0.696633
XGB	MM	SMOTE	XGB__colsample_bytree : 0.5292839702664169; XGB__gamma : 5.389372251327775;	0.692881	0.765498

			XGB__max_depth : 1; XGB__min_child_weight : 0.0; XGB__random_state : 0; XGB__scale_pos_weight : 3.7862116145489155; XGB__subsample : 0.8325823249082597		
NN	SS	SMOTE	NN__activation : tanh; NN__alpha : 0.07125080367906376; NN__hidden_layer_sizes : 3; NN__max_iter : 500; NN__random_state : 0; NN__solver : adam	0.692864	0.721826
NN	SS	ROS	NN__activation : tanh; NN__alpha : 10.0; NN__hidden_layer_sizes : 50; NN__max_iter : 50; NN__random_state : 0; NN__solver : adam	0.692757	0.704562
XGB	MM	SMOTE	XGB__colsample_bytree : 0.5866829103915807; XGB__gamma : 4.49373707241089; XGB__max_depth : 1; XGB__min_child_weight : 0.0; XGB__random_state : 0; XGB__scale_pos_weight : 3.6703470418497735; XGB__subsample : 0.9	0.692685	0.766804
NN	SS	ADASYN	NN__activation : logistic; NN__alpha : 0.0001; NN__hidden_layer_sizes : 40; NN__max_iter : 171; NN__random_state : 0; NN__solver : adam	0.692673	0.703172
RF	SS	SMOTE	RF__class_weight : balanced; RF__criterion : log_loss; RF__max_depth : 1; RF__max_features : 2; RF__min_samples_leaf : 12; RF__n_estimators : 343; RF__random_state : 25	0.692364	0.724846
XGB	SS	ADASYN	XGB__colsample_bytree : 0.1; XGB__gamma : 3.021437347663065; XGB__max_depth : 1; XGB__min_child_weight : 0.0; XGB__random_state : 0; XGB__scale_pos_weight : 1.7124216154620462; XGB__subsample : 0.9	0.692358	0.768877
RF	SS	SMOTE	RF__class_weight : balanced; RF__criterion : gini; RF__max_depth : 3; RF__max_features : 9;	0.692154	0.794638

			RF__min_samples_leaf : 1; RF__n_estimators : 614; RF__random_state : 96		
RF	SS	SMOTE	RF__class_weight : balanced_subsample; RF__criterion : log_loss; RF__max_depth : 3; RF__max_features : 8; RF__min_samples_leaf : 3; RF__n_estimators : 319; RF__random_state : 57	0.692137	0.789923
XGB	MM	SMOTE	XGB__colsample_bytree : 0.5437700723328859; XGB__gamma : 5.707424877893317; XGB__max_depth : 1; XGB__min_child_weight : 0.0; XGB__random_state : 0; XGB__scale_pos_weight : 3.7621912919751628; XGB__subsample : 0.8270266378914082	0.692124	0.761118
XGB	MM	SMOTE	XGB__colsample_bytree : 0.6966393683050776; XGB__gamma : 6.457676393022992; XGB__max_depth : 1; XGB__min_child_weight : 0.0; XGB__random_state : 0; XGB__scale_pos_weight : 3.9466483912053016; XGB__subsample : 0.9	0.691853	0.759928
NN	SS	SMOTE	NN__activation : logistic; NN__alpha : 0.10062227690897685; NN__hidden_layer_sizes : 50; NN__max_iter : 500; NN__random_state : 0; NN__solver : adam	0.691572	0.703884
NN	SS	ROS	NN__activation : tanh; NN__alpha : 10.0; NN__hidden_layer_sizes : 3; NN__max_iter : 500; NN__random_state : 0; NN__solver : adam	0.691558	0.701091
NN	SS	ROS	NN__activation : identity; NN__alpha : 10.0; NN__hidden_layer_sizes : 50; NN__max_iter : 54; NN__random_state : 0; NN__solver : lbfgs	0.691501	0.702256
XGB	SS	ROS	XGB__colsample_bytree : 0.1; XGB__gamma : 0.01; XGB__max_depth : 1; XGB__min_child_weight : 0.0; XGB__random_state : 0; XGB__scale_pos_weight :	0.691362	0.783744

			1.6825471777346699; XGB__subsample : 0.666098393693635		
XGB	SS	ROS	XGB__colsample_bytree : 0.1; XGB__gamma : 0.01; XGB__max_depth : 1; XGB__min_child_weight : 4.349577253151341; XGB__random_state : 0; XGB__scale_pos_weight : 2.097687129430841; XGB__subsample : 0.5	0.691097	0.768453
XGB	SS	ADASYN	XGB__colsample_bytree : 0.1; XGB__gamma : 3.4516403509060947; XGB__max_depth : 1; XGB__min_child_weight : 0.0; XGB__random_state : 0; XGB__scale_pos_weight : 1.8031768252910492; XGB__subsample : 0.5	0.691081	0.760445
NN	SS	ADASYN	NN__activation : logistic; NN__alpha : 0.3663928960626971; NN__hidden_layer_sizes : 30; NN__max_iter : 500; NN__random_state : 0; NN__solver : adam	0.691073	0.701667
XGB	SS	ADASYN	XGB__colsample_bytree : 0.1; XGB__gamma : 1.3404544702071914; XGB__max_depth : 1; XGB__min_child_weight : 0.0; XGB__random_state : 0; XGB__scale_pos_weight : 0.5; XGB__subsample : 0.5	0.691051	0.763946
NN	SS	SMOTE	NN__activation : logistic; NN__alpha : 0.0019011746609606456; NN__hidden_layer_sizes : 50; NN__max_iter : 500; NN__random_state : 0; NN__solver : adam	0.69078	0.703937
XGB	MM	ADASYN	XGB__colsample_bytree : 1.0; XGB__gamma : 4.364606566317022; XGB__max_depth : 1; XGB__min_child_weight : 0.0; XGB__random_state : 0; XGB__scale_pos_weight : 0.5; XGB__subsample : 0.5	0.690725	0.758276

NN	SS	ROS	NN__activation : identity; NN__alpha : 0.0001; NN__hidden_layer_sizes : 50; NN__max_iter : 50; NN__random_state : 0; NN__solver : adam	0.690709	0.702271
RF	SS	SMOTE	RF__class_weight : None; RF__criterion : gini; RF__max_depth : 1; RF__max_features : 1; RF__min_samples_leaf : 1; RF__n_estimators : 318; RF__random_state : 85	0.690623	0.732294
XGB	SS	ADASYN	XGB__colsample_bytree : 0.1; XGB__gamma : 4.930722956029769; XGB__max_depth : 1; XGB__min_child_weight : 0.0; XGB__random_state : 0; XGB__scale_pos_weight : 0.5; XGB__subsample : 0.5	0.690613	0.726211
XGB	SS	ADASYN	XGB__colsample_bytree : 0.1; XGB__gamma : 3.0300554993150377; XGB__max_depth : 1; XGB__min_child_weight : 0.0; XGB__random_state : 0; XGB__scale_pos_weight : 3.474607422256215; XGB__subsample : 0.9	0.69061	0.770336
XGB	MM	SMOTE	XGB__colsample_bytree : 0.5329561500821663; XGB__gamma : 6.193894008943266; XGB__max_depth : 1; XGB__min_child_weight : 0.0; XGB__random_state : 0; XGB__scale_pos_weight : 4.0172360533660765; XGB__subsample : 0.9	0.690484	0.758186
XGB	Not used	Not used	XGB__colsample_bytree : 0.969105573687723; XGB__gamma : 3.9229310692710517; XGB__max_depth : 2; XGB__min_child_weight : 0.0; XGB__random_state : 0; XGB__scale_pos_weight : 0.5; XGB__subsample : 0.866403240900494	0.690482	0.764026
XGB	SS	ROS	XGB__colsample_bytree : 0.22627644728878518; XGB__gamma : 0.01; XGB__max_depth : 1; XGB__min_child_weight : 0.0; XGB__random_state :	0.690373	0.779275

			0; XGB__scale_pos_weight : 5.0; XGB__subsample : 0.5096725459468833		
XGB	MM	SMOTE	XGB__colsample_bytree : 0.7991353418231801; XGB__gamma : 6.842031677841393; XGB__max_depth : 1; XGB__min_child_weight : 0.0; XGB__random_state : 0; XGB__scale_pos_weight : 3.9724298487997705; XGB__subsample : 0.9	0.690337	0.758813
NN	SS	ROS	NN__activation : identity; NN__alpha : 0.007282087011117253; NN__hidden_layer_sizes : 50; NN__max_iter : 500; NN__random_state : 0; NN__solver : adam	0.69033	0.702196
RF	SS	SMOTE	RF__class_weight : balanced_subsample; RF__criterion : log_loss; RF__max_depth : 2; RF__max_features : 3; RF__min_samples_leaf : 11; RF__n_estimators : 470; RF__random_state : 46	0.690312	0.741356
XGB	Not used	Not used	XGB__colsample_bytree : 1.0; XGB__gamma : 7.91156646020262; XGB__max_depth : 3; XGB__min_child_weight : 0.0; XGB__random_state : 0; XGB__scale_pos_weight : 3.291022952759355; XGB__subsample : 0.8029335000309636	0.690308	0.826784
RF	SS	SMOTE	RF__class_weight : None; RF__criterion : gini; RF__max_depth : 5; RF__max_features : 1; RF__min_samples_leaf : 2; RF__n_estimators : 1000; RF__random_state : 0	0.690163	0.849565
XGB	MM	ADASYN	XGB__colsample_bytree : 1.0; XGB__gamma : 11.621325063724372; XGB__max_depth : 6; XGB__min_child_weight : 0.0; XGB__random_state : 0; XGB__scale_pos_weight : 2.4121603268482694; XGB__subsample : 0.8815630067016531	0.690047	0.811156

NN	SS	ADASYN	NN__activation : logistic; NN__alpha : 0.0001; NN__hidden_layer_sizes : 3; NN__max_iter : 463; NN__random_state : 0; NN__solver : adam	0.690043	0.692972
XGB	Not used	Not used	XGB__colsample_bytree : 0.1; XGB__gamma : 8.186133302415978; XGB__max_depth : 1; XGB__min_child_weight : 0.0; XGB__random_state : 0; XGB__scale_pos_weight : 3.097005067296185; XGB__subsample : 0.9	0.690024	0.746615
XGB	Not used	Not used	XGB__colsample_bytree : 0.8173650468662265; XGB__gamma : 2.9204370293898365; XGB__max_depth : 1; XGB__min_child_weight : 0.0; XGB__random_state : 0; XGB__scale_pos_weight : 2.2160160154091493; XGB__subsample : 0.9	0.690023	0.778346
XGB	SS	ROS	XGB__colsample_bytree : 0.1; XGB__gamma : 0.01; XGB__max_depth : 1; XGB__min_child_weight : 13.10393335588331; XGB__random_state : 0; XGB__scale_pos_weight : 2.9003621236310666; XGB__subsample : 0.9	0.690011	0.752979
XGB	Not used	Not used	XGB__colsample_bytree : 1.0; XGB__gamma : 3.9662383677555546; XGB__max_depth : 1; XGB__min_child_weight : 0.0; XGB__random_state : 0; XGB__scale_pos_weight : 3.4031107291041716; XGB__subsample : 0.9	0.689972	0.779066
RF	SS	SMOTE	RF__class_weight : balanced; RF__criterion : log_loss; RF__max_depth : 4; RF__max_features : 8; RF__min_samples_leaf : 1; RF__n_estimators : 391; RF__random_state : 0	0.689866	0.830763
XGB	SS	SMOTE	XGB__colsample_bytree : 1.0; XGB__gamma : 5.131091349001754; XGB__max_depth : 1; XGB__min_child_weight : 7.36376132291421;	0.689865	0.756343

			XGB__random_state : 0; XGB__scale_pos_weight : 4.175844606564892; XGB__subsample : 0.5		
NN	Not used	Not used	NN__activation : tanh; NN__alpha : 10.0; NN__hidden_layer_sizes : 3; NN__max_iter : 408; NN__random_state : 0; NN__solver : lbfgs	0.689812	0.713764
XGB	SS	ADASYN	XGB__colsample_bytree : 1.0; XGB__gamma : 7.20050984483231; XGB__max_depth : 1; XGB__min_child_weight : 0.0; XGB__random_state : 0; XGB__scale_pos_weight : 2.7612210590268162; XGB__subsample : 0.9	0.689694	0.754667
NN	SS	ROS	NN__activation : tanh; NN__alpha : 10.0; NN__hidden_layer_sizes : 50; NN__max_iter : 500; NN__random_state : 0; NN__solver : lbfgs	0.689676	0.70531
NN	SS	SMOTE	NN__activation : tanh; NN__alpha : 0.003489939724014517; NN__hidden_layer_sizes : 3; NN__max_iter : 500; NN__random_state : 0; NN__solver : adam	0.68966	0.722528
RF	SS	SMOTE	RF__class_weight : None; RF__criterion : log_loss; RF__max_depth : 2; RF__max_features : 9; RF__min_samples_leaf : 13; RF__n_estimators : 323; RF__random_state : 14	0.689628	0.73444
XGB	SS	ROS	XGB__colsample_bytree : 0.1; XGB__gamma : 0.01; XGB__max_depth : 1; XGB__min_child_weight : 0.0; XGB__random_state : 0; XGB__scale_pos_weight : 2.5563713240556116; XGB__subsample : 0.580083322605866	0.689546	0.782886
NN	SS	ADASYN	NN__activation : logistic; NN__alpha : 10.0; NN__hidden_layer_sizes : 50; NN__max_iter : 500; NN__random_state : 0; NN__solver : lbfgs	0.68954	0.704045

NN	SS	ADASYN	NN__activation : logistic; NN__alpha : 10.0; NN__hidden_layer_sizes : 4; NN__max_iter : 59; NN__random_state : 0; NN__solver : lbfgs	0.689523	0.704365
NN	Not used	Not used	NN__activation : tanh; NN__alpha : 10.0; NN__hidden_layer_sizes : 3; NN__max_iter : 486; NN__random_state : 0; NN__solver : lbfgs	0.689433	0.714083
NN	SS	ADASYN	NN__activation : identity; NN__alpha : 0.0001; NN__hidden_layer_sizes : 40; NN__max_iter : 497; NN__random_state : 0; NN__solver : adam	0.689419	0.701636
XGB	SS	ADASYN	XGB__colsample_bytree : 0.5798993919824169; XGB__gamma : 3.594635122330068; XGB__max_depth : 1; XGB__min_child_weight : 0.0; XGB__random_state : 0; XGB__scale_pos_weight : 0.5; XGB__subsample : 0.6711929722506166	0.689362	0.754136
XGB	MM	SMOTE	XGB__colsample_bytree : 0.67424325634792; XGB__gamma : 5.457191084340767; XGB__max_depth : 1; XGB__min_child_weight : 0.0; XGB__random_state : 0; XGB__scale_pos_weight : 3.6228448439232492; XGB__subsample : 0.9	0.689215	0.76386
NN	MM	ROS	NN__activation : identity; NN__alpha : 0.0001; NN__hidden_layer_sizes : 25; NN__max_iter : 50; NN__random_state : 0; NN__solver : lbfgs	0.689194	0.70215
XGB	Not used	Not used	XGB__colsample_bytree : 0.306395007148906; XGB__gamma : 0.9265721861190738; XGB__max_depth : 1; XGB__min_child_weight : 1.0154839677303173; XGB__random_state : 0; XGB__scale_pos_weight : 0.5; XGB__subsample : 0.9	0.689132	0.765692
XGB	MM	SMOTE	XGB__colsample_bytree : 0.5728842704861302; XGB__gamma : 6.021795747638219; XGB__max_depth : 1; XGB__min_child_weight : 0.0; XGB__random_state : 0; XGB__scale_pos_weight :	0.689127	0.76203

			3.98740617698512; XGB__subsample : 0.8330998515021423		
XGB	SS	ROS	XGB__colsample_bytree : 0.1; XGB__gamma : 0.01; XGB__max_depth : 1; XGB__min_child_weight : 0.0; XGB__random_state : 0; XGB__scale_pos_weight : 4.129769645270979; XGB__subsample : 0.5	0.689097	0.778562
NN	SS	SMOTE	NN__activation : logistic; NN__alpha : 6.07913840947257; NN__hidden_layer_sizes : 50; NN__max_iter : 50; NN__random_state : 0; NN__solver : adam	0.689023	0.703601
NN	SS	ROS	NN__activation : tanh; NN__alpha : 0.0001; NN__hidden_layer_sizes : 50; NN__max_iter : 50; NN__random_state : 0; NN__solver : adam	0.68902	0.706847
NN	MM	ROS	NN__activation : identity; NN__alpha : 1.0905857484072239; NN__hidden_layer_sizes : 10; NN__max_iter : 500; NN__random_state : 0; NN__solver : lbfgs	0.688987	0.700411
XGB	MM	ADASYN	XGB__colsample_bytree : 1.0; XGB__gamma : 5.438958966584836; XGB__max_depth : 1; XGB__min_child_weight : 0.0; XGB__random_state : 0; XGB__scale_pos_weight : 1.444798323656828; XGB__subsample : 0.5	0.688986	0.765577
XGB	Not used	Not used	XGB__colsample_bytree : 1.0; XGB__gamma : 8.03926030885034; XGB__max_depth : 1; XGB__min_child_weight : 0.0; XGB__random_state : 0; XGB__scale_pos_weight : 3.7621340300083714; XGB__subsample : 0.781695368008678	0.688984	0.761487
XGB	MM	ADASYN	XGB__colsample_bytree : 1.0; XGB__gamma : 7.515115034838921; XGB__max_depth : 1; XGB__min_child_weight : 0.0; XGB__random_state :	0.68898	0.716864

			0; XGB__scale_pos_weight : 0.5; XGB__subsample : 0.5		
XGB	MM	ADASYN	XGB__colsample_bytree : 1.0; XGB__gamma : 11.057024575219641; XGB__max_depth : 5; XGB__min_child_weight : 0.0; XGB__random_state : 0; XGB__scale_pos_weight : 1.571408444289567; XGB__subsample : 0.5	0.68898	0.773865
NN	MM	ROS	NN__activation : identity; NN__alpha : 1.3543790179385211; NN__hidden_layer_sizes : 3; NN__max_iter : 357; NN__random_state : 0; NN__solver : lbfgs	0.688902	0.700021
NN	SS	ROS	NN__activation : relu; NN__alpha : 10.0; NN__hidden_layer_sizes : 50; NN__max_iter : 500; NN__random_state : 0; NN__solver : adam	0.688902	0.705962
NN	MM	ROS	NN__activation : identity; NN__alpha : 0.0001; NN__hidden_layer_sizes : 50; NN__max_iter : 50; NN__random_state : 0; NN__solver : lbfgs	0.688815	0.702133
NN	SS	ROS	NN__activation : identity; NN__alpha : 0.0001; NN__hidden_layer_sizes : 40; NN__max_iter : 122; NN__random_state : 0; NN__solver : lbfgs	0.688815	0.702133
XGB	SS	SMOTE	XGB__colsample_bytree : 0.181320495951649; XGB__gamma : 2.0410812235423212; XGB__max_depth : 1; XGB__min_child_weight : 7.86328897006377; XGB__random_state : 0; XGB__scale_pos_weight : 3.8569717111789372; XGB__subsample : 0.9	0.688812	0.761366
XGB	Not used	Not used	XGB__colsample_bytree : 1.0; XGB__gamma : 10.878489531937504; XGB__max_depth : 4; XGB__min_child_weight : 0.0; XGB__random_state : 0; XGB__scale_pos_weight : 3.1389986492654187; XGB__subsample : 0.9	0.688793	0.807072

XGB	SS	ADASYN	XGB__colsample_bytree : 0.1; XGB__gamma : 0.01; XGB__max_depth : 1; XGB__min_child_weight : 0.0; XGB__random_state : 0; XGB__scale_pos_weight : 0.5; XGB__subsample : 0.9	0.688768	0.773357
NN	SS	ADASYN	NN__activation : logistic; NN__alpha : 10.0; NN__hidden_layer_sizes : 3; NN__max_iter : 50; NN__random_state : 0; NN__solver : lbfgs	0.688766	0.70438
NN	Not used	Not used	NN__activation : identity; NN__alpha : 0.00960372168030384; NN__hidden_layer_sizes : 4; NN__max_iter : 500; NN__random_state : 0; NN__solver : lbfgs	0.688763	0.703187
NN	Not used	Not used	NN__activation : identity; NN__alpha : 0.0001; NN__hidden_layer_sizes : 4; NN__max_iter : 146; NN__random_state : 0; NN__solver : lbfgs	0.688453	0.702909
NN	SS	ADASYN	NN__activation : identity; NN__alpha : 10.0; NN__hidden_layer_sizes : 20; NN__max_iter : 50; NN__random_state : 0; NN__solver : lbfgs	0.688438	0.704514
XGB	MM	ADASYN	XGB__colsample_bytree : 1.0; XGB__gamma : 4.821937101666894; XGB__max_depth : 1; XGB__min_child_weight : 0.0; XGB__random_state : 0; XGB__scale_pos_weight : 0.5; XGB__subsample : 0.5	0.688435	0.752236
NN	SS	ADASYN	NN__activation : identity; NN__alpha : 5.439673928658996; NN__hidden_layer_sizes : 50; NN__max_iter : 50; NN__random_state : 0; NN__solver : lbfgs	0.68842	0.704765
NN	Not used	Not used	NN__activation : identity; NN__alpha : 0.0417544842586434; NN__hidden_layer_sizes : 6; NN__max_iter : 500; NN__random_state : 0; NN__solver : lbfgs	0.688384	0.7031

XGB	MM	ADASYN	XGB__colsample_bytree : 1.0; XGB__gamma : 5.20375706309181; XGB__max_depth : 1; XGB__min_child_weight : 0.0; XGB__random_state : 0; XGB__scale_pos_weight : 1.464186120029798; XGB__subsample : 0.5	0.688378	0.76575
NN	SS	ROS	NN__activation : identity; NN__alpha : 1.2278635248432284; NN__hidden_layer_sizes : 27; NN__max_iter : 165; NN__random_state : 0; NN__solver : adam	0.688367	0.701942
XGB	SS	ADASYN	XGB__colsample_bytree : 1.0; XGB__gamma : 11.255443825347797; XGB__max_depth : 4; XGB__min_child_weight : 0.0; XGB__random_state : 0; XGB__scale_pos_weight : 5.0; XGB__subsample : 0.9	0.688327	0.831469
NN	SS	SMOTE	NN__activation : logistic; NN__alpha : 0.43513970791520495; NN__hidden_layer_sizes : 40; NN__max_iter : 192; NN__random_state : 0; NN__solver : adam	0.688264	0.703614
XGB	MM	ROS	XGB__colsample_bytree : 1.0; XGB__gamma : 13.034380619531959; XGB__max_depth : 1; XGB__min_child_weight : 7.3565562570639775; XGB__random_state : 0; XGB__scale_pos_weight : 4.553474636855185; XGB__subsample : 0.7294030327236785	0.688149	0.738611
XGB	SS	SMOTE	XGB__colsample_bytree : 0.1707401115876679; XGB__gamma : 6.298649339001223; XGB__max_depth : 1; XGB__min_child_weight : 9.56810194026891; XGB__random_state : 0; XGB__scale_pos_weight : 5.0; XGB__subsample : 0.9	0.688134	0.744455

NN	SS	ADASYN	NN__activation : identity; NN__alpha : 10.0; NN__hidden_layer_sizes : 50; NN__max_iter : 500; NN__random_state : 0; NN__solver : lbfgs	0.688059	0.704678
NN	SS	ADASYN	NN__activation : identity; NN__alpha : 10.0; NN__hidden_layer_sizes : 3; NN__max_iter : 500; NN__random_state : 0; NN__solver : lbfgs	0.688025	0.704688
NN	Not used	Not used	NN__activation : identity; NN__alpha : 0.009473292575666816; NN__hidden_layer_sizes : 4; NN__max_iter : 500; NN__random_state : 0; NN__solver : lbfgs	0.688005	0.703148
NN	SS	SMOTE	NN__activation : logistic; NN__alpha : 5.451335018692285; NN__hidden_layer_sizes : 50; NN__max_iter : 50; NN__random_state : 0; NN__solver : adam	0.687853	0.703625
XGB	MM	SMOTE	XGB__colsample_bytree : 0.6842842891710897; XGB__gamma : 7.813493954838153; XGB__max_depth : 1; XGB__min_child_weight : 0.0; XGB__random_state : 0; XGB__scale_pos_weight : 3.9791548904893723; XGB__subsample : 0.8325149428493588	0.687682	0.752031
XGB	MM	ADASYN	XGB__colsample_bytree : 1.0; XGB__gamma : 4.284700058509442; XGB__max_depth : 1; XGB__min_child_weight : 0.0; XGB__random_state : 0; XGB__scale_pos_weight : 0.5; XGB__subsample : 0.5	0.687677	0.759733
NN	SS	ADASYN	NN__activation : identity; NN__alpha : 5.057387294024656; NN__hidden_layer_sizes : 4; NN__max_iter : 153; NN__random_state : 0; NN__solver : lbfgs	0.687662	0.704673
NN	Not used	Not used	NN__activation : identity; NN__alpha : 0.0301999644723022; NN__hidden_layer_sizes : 10;	0.687626	0.703085

			NN__max_iter : 500; NN__random_state : 0; NN__solver : lbfgs		
NN	Not used	Not used	NN__activation : identity; NN__alpha : 0.03922808864950279; NN__hidden_layer_sizes : 6; NN__max_iter : 500; NN__random_state : 0; NN__solver : lbfgs	0.687592	0.703018
NN	MM	ROS	NN__activation : identity; NN__alpha : 0.31318205551038464; NN__hidden_layer_sizes : 6; NN__max_iter : 50; NN__random_state : 0; NN__solver : lbfgs	0.687559	0.70175
XGB	SS	ADASYN	XGB__colsample_bytree : 0.1; XGB__gamma : 0.01; XGB__max_depth : 1; XGB__min_child_weight : 0.0; XGB__random_state : 0; XGB__scale_pos_weight : 4.999999999999999; XGB__subsample : 0.9	0.687475	0.774739
NN	SS	ADASYN	NN__activation : tanh; NN__alpha : 0.00046250380792257244; NN__hidden_layer_sizes : 40; NN__max_iter : 83; NN__random_state : 0; NN__solver : adam	0.687454	0.702916
NN	SS	SMOTE	NN__activation : logistic; NN__alpha : 10.0; NN__hidden_layer_sizes : 50; NN__max_iter : 50; NN__random_state : 0; NN__solver : adam	0.687439	0.703554
NN	SS	ADASYN	NN__activation : logistic; NN__alpha : 0.0001; NN__hidden_layer_sizes : 50; NN__max_iter : 321; NN__random_state : 0; NN__solver : adam	0.687423	0.704161
NN	SS	ROS	NN__activation : tanh; NN__alpha : 10.0; NN__hidden_layer_sizes : 5; NN__max_iter : 429; NN__random_state : 0; NN__solver : lbfgs	0.687421	0.706508
XGB	MM	SMOTE	XGB__colsample_bytree : 0.6416547901500855; XGB__gamma : 5.379018527502745; XGB__max_depth : 1; XGB__min_child_weight : 0.0;	0.687339	0.76513

			XGB__random_state : 0; XGB__scale_pos_weight : 3.82219914081332; XGB__subsample : 0.9		
NN	SS	ADASYN	NN__activation : identity; NN__alpha : 10.0; NN__hidden_layer_sizes : 40; NN__max_iter : 197; NN__random_state : 0; NN__solver : lbfgs	0.687284	0.704746
NN	Not used	Not used	NN__activation : identity; NN__alpha : 0.0375186019885839; NN__hidden_layer_sizes : 6; NN__max_iter : 500; NN__random_state : 0; NN__solver : lbfgs	0.687264	0.703139
NN	Not used	Not used	NN__activation : identity; NN__alpha : 0.0001; NN__hidden_layer_sizes : 3; NN__max_iter : 130; NN__random_state : 0; NN__solver : lbfgs	0.687247	0.70309
NN	Not used	Not used	NN__activation : identity; NN__alpha : 0.04508448195578453; NN__hidden_layer_sizes : 6; NN__max_iter : 500; NN__random_state : 0; NN__solver : lbfgs	0.687231	0.702937
NN	Not used	Not used	NN__activation : identity; NN__alpha : 0.015649016009266582; NN__hidden_layer_sizes : 10; NN__max_iter : 500; NN__random_state : 0; NN__solver : lbfgs	0.687229	0.703018
XGB	Not used	Not used	XGB__colsample_bytree : 0.1; XGB__gamma : 0.01; XGB__max_depth : 1; XGB__min_child_weight : 0.0; XGB__random_state : 0; XGB__scale_pos_weight : 2.710211421062956; XGB__subsample : 0.9	0.687223	0.789563
NN	SS	ADASYN	NN__activation : identity; NN__alpha : 3.3117909977544056; NN__hidden_layer_sizes : 8; NN__max_iter : 473; NN__random_state : 0; NN__solver : lbfgs	0.687215	0.704852
NN	Not used	Not used	NN__activation : identity; NN__alpha : 0.013864640789074593; NN__hidden_layer_sizes : 10;	0.687213	0.703109

			NN__max_iter : 500; NN__random_state : 0; NN__solver : lbfgs		
NN	Not used	Not used	NN__activation : identity; NN__alpha : 0.0428487733335792; NN__hidden_layer_sizes : 6; NN__max_iter : 500; NN__random_state : 0; NN__solver : lbfgs	0.687197	0.702912
NN	MM	ROS	NN__activation : identity; NN__alpha : 0.24445194577764492; NN__hidden_layer_sizes : 35; NN__max_iter : 405; NN__random_state : 0; NN__solver : lbfgs	0.687162	0.701967
NN	SS	ADASYN	NN__activation : logistic; NN__alpha : 0.04305231117279188; NN__hidden_layer_sizes : 50; NN__max_iter : 499; NN__random_state : 0; NN__solver : adam	0.687044	0.704195
NN	Not used	Not used	NN__activation : identity; NN__alpha : 0.04570853433831053; NN__hidden_layer_sizes : 6; NN__max_iter : 500; NN__random_state : 0; NN__solver : lbfgs	0.686834	0.703003
XGB	Not used	Not used	XGB__colsample_bytree : 1.0; XGB__gamma : 0.01; XGB__max_depth : 1; XGB__min_child_weight : 0.0; XGB__random_state : 0; XGB__scale_pos_weight : 2.908258551955626; XGB__subsample : 0.9	0.686754	0.802656
NN	MM	ADASYN	NN__activation : relu; NN__alpha : 3.9814946105106603; NN__hidden_layer_sizes : 15; NN__max_iter : 427; NN__random_state : 0; NN__solver : adam	0.686685	0.697165
XGB	SS	SMOTE	XGB__colsample_bytree : 1.0; XGB__gamma : 1.9017948765351307; XGB__max_depth : 1; XGB__min_child_weight : 8.149836108001697; XGB__random_state : 0; XGB__scale_pos_weight : 5.0; XGB__subsample : 0.9	0.686682	0.768215

XGB	MM	SMOTE	XGB__colsample_bytree : 0.665070071816777; XGB__gamma : 6.333018425892856; XGB__max_depth : 1; XGB__min_child_weight : 0.0; XGB__random_state : 0; XGB__scale_pos_weight : 3.641847917547219; XGB__subsample : 0.8508568816277423	0.686657	0.759008
XGB	Not used	Not used	XGB__colsample_bytree : 0.7632618764416226; XGB__gamma : 11.800954452418459; XGB__max_depth : 1; XGB__min_child_weight : 0.0; XGB__random_state : 0; XGB__scale_pos_weight : 5.0; XGB__subsample : 0.5997315910352279	0.686639	0.750917
XGB	MM	SMOTE	XGB__colsample_bytree : 0.7865896772579317; XGB__gamma : 4.080624907867269; XGB__max_depth : 1; XGB__min_child_weight : 0.0; XGB__random_state : 0; XGB__scale_pos_weight : 4.0280090908703805; XGB__subsample : 0.9	0.686634	0.772403
NN	SS	ADASYN	NN__activation : logistic; NN__alpha : 0.43513970791520495; NN__hidden_layer_sizes : 40; NN__max_iter : 192; NN__random_state : 0; NN__solver : adam	0.686544	0.699169
NN	Not used	Not used	NN__activation : identity; NN__alpha : 0.043914740925410284; NN__hidden_layer_sizes : 6; NN__max_iter : 500; NN__random_state : 0; NN__solver : lbfgs	0.68649	0.702932
NN	Not used	Not used	NN__activation : identity; NN__alpha : 0.05358638384164173; NN__hidden_layer_sizes : 6; NN__max_iter : 500; NN__random_state : 0; NN__solver : lbfgs	0.686455	0.702999
NN	Not used	Not used	NN__activation : identity; NN__alpha : 0.01122128879270935; NN__hidden_layer_sizes : 10;	0.686455	0.703095

			NN__max_iter : 500; NN__random_state : 0; NN__solver : lbfgs		
NN	SS	ADASYN	NN__activation : logistic; NN__alpha : 0.00044991471422135625; NN__hidden_layer_sizes : 27; NN__max_iter : 452; NN__random_state : 0; NN__solver : adam	0.686421	0.703298
NN	SS	ADASYN	NN__activation : identity; NN__alpha : 0.0009214284259312227; NN__hidden_layer_sizes : 30; NN__max_iter : 50; NN__random_state : 0; NN__solver : adam	0.686408	0.700694
NN	Not used	Not used	NN__activation : identity; NN__alpha : 0.01397339208400228; NN__hidden_layer_sizes : 10; NN__max_iter : 500; NN__random_state : 0; NN__solver : lbfgs	0.686405	0.703095
XGB	SS	ROS	XGB__colsample_bytree : 0.1; XGB__gamma : 0.01; XGB__max_depth : 1; XGB__min_child_weight : 13.080933416539409; XGB__random_state : 0; XGB__scale_pos_weight : 3.995624674375618; XGB__subsample : 0.9	0.686377	0.759347
XGB	MM	ROS	XGB__colsample_bytree : 1.0; XGB__gamma : 23.97177848430441; XGB__max_depth : 8; XGB__min_child_weight : 0.0; XGB__random_state : 0; XGB__scale_pos_weight : 5.0; XGB__subsample : 0.6691298081725635	0.686317	0.798055
XGB	SS	ROS	XGB__colsample_bytree : 1.0; XGB__gamma : 0.01; XGB__max_depth : 1; XGB__min_child_weight : 16.523149769879616; XGB__random_state : 0; XGB__scale_pos_weight : 5.0; XGB__subsample : 0.9	0.686273	0.758686
NN	Not used	Not used	NN__activation : identity; NN__alpha : 0.41662566665749423; NN__hidden_layer_sizes : 3;	0.686129	0.70296

			NN__max_iter : 500; NN__random_state : 0; NN__solver : lbfgs		
NN	Not used	Not used	NN__activation : identity; NN__alpha : 0.040440309036605705; NN__hidden_layer_sizes : 6; NN__max_iter : 500; NN__random_state : 0; NN__solver : lbfgs	0.686093	0.703096
NN	Not used	Not used	NN__activation : identity; NN__alpha : 0.043636434202618694; NN__hidden_layer_sizes : 6; NN__max_iter : 500; NN__random_state : 0; NN__solver : lbfgs	0.686077	0.7031
NN	Not used	Not used	NN__activation : identity; NN__alpha : 0.03795004970390232; NN__hidden_layer_sizes : 6; NN__max_iter : 500; NN__random_state : 0; NN__solver : lbfgs	0.686059	0.703003
NN	Not used	Not used	NN__activation : identity; NN__alpha : 0.03781777508749155; NN__hidden_layer_sizes : 6; NN__max_iter : 500; NN__random_state : 0; NN__solver : lbfgs	0.686042	0.703134
NN	Not used	Not used	NN__activation : identity; NN__alpha : 0.025670769542096534; NN__hidden_layer_sizes : 22; NN__max_iter : 500; NN__random_state : 0; NN__solver : lbfgs	0.686024	0.702975
XGB	Not used	Not used	XGB__colsample_bytree : 1.0; XGB__gamma : 6.62183984453924; XGB__max_depth : 1; XGB__min_child_weight : 0.0; XGB__random_state : 0; XGB__scale_pos_weight : 3.8091146094219717; XGB__subsample : 0.8968131174212	0.686005	0.763068
XGB	SS	ADASYN	XGB__colsample_bytree : 1.0; XGB__gamma : 5.19326877350158; XGB__max_depth : 1; XGB__min_child_weight : 0.0; XGB__random_state :	0.685894	0.764627

			0; XGB__scale_pos_weight : 1.1792763822325183; XGB__subsample : 0.5		
XGB	MM	ADASYN	XGB__colsample_bytree : 0.1; XGB__gamma : 0.01; XGB__max_depth : 1; XGB__min_child_weight : 0.0; XGB__random_state : 0; XGB__scale_pos_weight : 3.2919024155881385; XGB__subsample : 0.9	0.685827	0.776299
NN	SS	ADASYN	NN__activation : logistic; NN__alpha : 0.22837885717498688; NN__hidden_layer_sizes : 50; NN__max_iter : 500; NN__random_state : 0; NN__solver : adam	0.685804	0.703748
XGB	SS	SMOTE	XGB__colsample_bytree : 1.0; XGB__gamma : 4.328942673249065; XGB__max_depth : 1; XGB__min_child_weight : 1.4814464697611438; XGB__random_state : 0; XGB__scale_pos_weight : 5.0; XGB__subsample : 0.9	0.685777	0.767624
NN	Not used	Not used	NN__activation : identity; NN__alpha : 10.0; NN__hidden_layer_sizes : 50; NN__max_iter : 500; NN__random_state : 0; NN__solver : lbfgs	0.685773	0.701371
NN	Not used	Not used	NN__activation : identity; NN__alpha : 0.04095316977413132; NN__hidden_layer_sizes : 6; NN__max_iter : 500; NN__random_state : 0; NN__solver : lbfgs	0.685732	0.702994
XGB	SS	SMOTE	XGB__colsample_bytree : 1.0; XGB__gamma : 0.01; XGB__max_depth : 1; XGB__min_child_weight : 14.260508560698518; XGB__random_state : 0; XGB__scale_pos_weight : 5.0; XGB__subsample : 0.9	0.685671	0.755818
NN	SS	ADASYN	NN__activation : identity; NN__alpha : 1.6524873646335299; NN__hidden_layer_sizes : 3; NN__max_iter : 50; NN__random_state : 0; NN__solver : lbfgs	0.685665	0.704775

NN	Not used	Not used	NN__activation : identity; NN__alpha : 0.042433570795289574; NN__hidden_layer_sizes : 6; NN__max_iter : 500; NN__random_state : 0; NN__solver : lbfgs	0.685663	0.703009
NN	Not used	Not used	NN__activation : identity; NN__alpha : 10.0; NN__hidden_layer_sizes : 3; NN__max_iter : 500; NN__random_state : 0; NN__solver : lbfgs	0.685395	0.701432
XGB	MM	ROS	XGB__colsample_bytree : 1.0; XGB__gamma : 14.506826759274759; XGB__max_depth : 1; XGB__min_child_weight : 10.702249995013977; XGB__random_state : 0; XGB__scale_pos_weight : 3.7398854666430434; XGB__subsample : 0.7295681381996688	0.685354	0.735675
XGB	SS	ROS	XGB__colsample_bytree : 0.1; XGB__gamma : 0.01; XGB__max_depth : 1; XGB__min_child_weight : 0.0; XGB__random_state : 0; XGB__scale_pos_weight : 3.3935025100209266; XGB__subsample : 0.5590918819212974	0.685275	0.781511
NN	SS	ADASYN	NN__activation : identity; NN__alpha : 0.013930278588117039; NN__hidden_layer_sizes : 3; NN__max_iter : 500; NN__random_state : 0; NN__solver : lbfgs	0.685252	0.704823
NN	MM	ADASYN	NN__activation : relu; NN__alpha : 10.0; NN__hidden_layer_sizes : 15; NN__max_iter : 431; NN__random_state : 0; NN__solver : adam	0.685237	0.693502
XGB	SS	ADASYN	XGB__colsample_bytree : 1.0; XGB__gamma : 13.762364455948857; XGB__max_depth : 6; XGB__min_child_weight : 0.0; XGB__random_state : 0; XGB__scale_pos_weight : 5.0; XGB__subsample : 0.9	0.685213	0.836589

XGB	SS	ROS	XGB__colsample_bytree : 0.9571430619381621; XGB__gamma : 0.01; XGB__max_depth : 1; XGB__min_child_weight : 0.0; XGB__random_state : 0; XGB__scale_pos_weight : 1.7918028137222497; XGB__subsample : 0.5351811764646127	0.685131	0.792058
NN	SS	ROS	NN__activation : relu; NN__alpha : 3.9814946105106603; NN__hidden_layer_sizes : 15; NN__max_iter : 427; NN__random_state : 0; NN__solver : adam	0.685093	0.721917
NN	MM	ADASYN	NN__activation : relu; NN__alpha : 4.623397721291552; NN__hidden_layer_sizes : 15; NN__max_iter : 391; NN__random_state : 0; NN__solver : adam	0.684965	0.695928
XGB	Not used	Not used	XGB__colsample_bytree : 1.0; XGB__gamma : 9.889675159430956; XGB__max_depth : 1; XGB__min_child_weight : 0.0; XGB__random_state : 0; XGB__scale_pos_weight : 3.772849164955461; XGB__subsample : 0.7190708844277698	0.684951	0.755873
NN	Not used	Not used	NN__activation : identity; NN__alpha : 0.24450918748319259; NN__hidden_layer_sizes : 10; NN__max_iter : 242; NN__random_state : 0; NN__solver : lbfgs	0.684942	0.703014
NN	SS	ADASYN	NN__activation : identity; NN__alpha : 0.5411600543905455; NN__hidden_layer_sizes : 50; NN__max_iter : 50; NN__random_state : 0; NN__solver : lbfgs	0.684873	0.704598
XGB	MM	SMOTE	XGB__colsample_bytree : 0.1; XGB__gamma : 0.01; XGB__max_depth : 1; XGB__min_child_weight : 0.0; XGB__random_state : 0; XGB__scale_pos_weight : 0.5; XGB__subsample : 0.6361967644248446	0.684866	0.769664

NN	SS	SMOTE	NN__activation : tanh; NN__alpha : 2.6096146808538574; NN__hidden_layer_sizes : 8; NN__max_iter : 478; NN__random_state : 0; NN__solver : adam	0.684801	0.712205
XGB	Not used	Not used	XGB__colsample_bytree : 0.42030240747014935; XGB__gamma : 0.01; XGB__max_depth : 1; XGB__min_child_weight : 0.12070101753670352; XGB__random_state : 0; XGB__scale_pos_weight : 0.5; XGB__subsample : 0.9	0.684653	0.789845
NN	SS	ADASYN	NN__activation : identity; NN__alpha : 0.0001; NN__hidden_layer_sizes : 50; NN__max_iter : 500; NN__random_state : 0; NN__solver : lbfgs	0.684494	0.704842
NN	SS	ADASYN	NN__activation : identity; NN__alpha : 0.0001; NN__hidden_layer_sizes : 7; NN__max_iter : 500; NN__random_state : 0; NN__solver : adam	0.684491	0.701181
XGB	MM	ADASYN	XGB__colsample_bytree : 0.1; XGB__gamma : 0.01; XGB__max_depth : 1; XGB__min_child_weight : 0.0; XGB__random_state : 0; XGB__scale_pos_weight : 2.3561711330511446; XGB__subsample : 0.9	0.684465	0.776834
XGB	MM	ROS	XGB__colsample_bytree : 1.0; XGB__gamma : 12.403338344335701; XGB__max_depth : 1; XGB__min_child_weight : 12.947512177353046; XGB__random_state : 0; XGB__scale_pos_weight : 3.6619985925099057; XGB__subsample : 0.8003847597690481	0.684395	0.73623
XGB	MM	ADASYN	XGB__colsample_bytree : 0.1; XGB__gamma : 0.01; XGB__max_depth : 1; XGB__min_child_weight : 0.0; XGB__random_state : 0; XGB__scale_pos_weight : 2.750867312591029; XGB__subsample : 0.9	0.684103	0.776915
XGB	MM	ADASYN	XGB__colsample_bytree : 0.9161986022010945; XGB__gamma : 14.708965317754027;	0.684093	0.8001

			XGB__max_depth : 5; XGB__min_child_weight : 0.0; XGB__random_state : 0; XGB__scale_pos_weight : 3.405378827602404; XGB__subsample : 0.9		
XGB	MM	ADASYN	XGB__colsample_bytree : 0.1; XGB__gamma : 0.01; XGB__max_depth : 1; XGB__min_child_weight : 0.0; XGB__random_state : 0; XGB__scale_pos_weight : 2.3036705290606543; XGB__subsample : 0.9	0.684086	0.777034
XGB	Not used	Not used	XGB__colsample_bytree : 0.22631499503043714; XGB__gamma : 2.37161859052575; XGB__max_depth : 1; XGB__min_child_weight : 0.0; XGB__random_state : 0; XGB__scale_pos_weight : 4.491758075821906; XGB__subsample : 0.8296146597514118	0.684071	0.784008
XGB	SS	ADASYN	XGB__colsample_bytree : 0.1; XGB__gamma : 4.209438230604554; XGB__max_depth : 1; XGB__min_child_weight : 0.0; XGB__random_state : 0; XGB__scale_pos_weight : 5.0; XGB__subsample : 0.9	0.683986	0.766442
NN	MM	ROS	NN__activation : identity; NN__alpha : 0.04805857396436818; NN__hidden_layer_sizes : 50; NN__max_iter : 500; NN__random_state : 0; NN__solver : adam	0.68394	0.698878
XGB	Not used	Not used	XGB__colsample_bytree : 1.0; XGB__gamma : 8.039658846222679; XGB__max_depth : 1; XGB__min_child_weight : 0.0; XGB__random_state : 0; XGB__scale_pos_weight : 3.367356397977191; XGB__subsample : 0.748378773480607	0.683835	0.763822
NN	MM	ADASYN	NN__activation : identity; NN__alpha : 0.015191029773769228; NN__hidden_layer_sizes : 35; NN__max_iter : 500; NN__random_state : 0; NN__solver : lbfgs	0.683789	0.704498

NN	MM	ADASYN	NN__activation : identity; NN__alpha : 0.4367664749354218; NN__hidden_layer_sizes : 30; NN__max_iter : 50; NN__random_state : 0; NN__solver : lbfgs	0.683771	0.704279
XGB	Not used	Not used	XGB__colsample_bytree : 1.0; XGB__gamma : 7.9045090750170335; XGB__max_depth : 1; XGB__min_child_weight : 0.0; XGB__random_state : 0; XGB__scale_pos_weight : 3.6597051214002723; XGB__subsample : 0.782868036600278	0.683715	0.762757
NN	SS	ROS	NN__activation : identity; NN__alpha : 0.0001; NN__hidden_layer_sizes : 3; NN__max_iter : 500; NN__random_state : 0; NN__solver : adam	0.683702	0.69708
NN	MM	ROS	NN__activation : relu; NN__alpha : 0.25133758836084097; NN__hidden_layer_sizes : 3; NN__max_iter : 50; NN__random_state : 0; NN__solver : lbfgs	0.683668	0.710854
XGB	SS	ADASYN	XGB__colsample_bytree : 1.0; XGB__gamma : 5.764439719589001; XGB__max_depth : 2; XGB__min_child_weight : 0.0; XGB__random_state : 0; XGB__scale_pos_weight : 0.9009281752791984; XGB__subsample : 0.9	0.683607	0.781639
NN	SS	SMOTE	NN__activation : logistic; NN__alpha : 10.0; NN__hidden_layer_sizes : 3; NN__max_iter : 50; NN__random_state : 0; NN__solver : lbfgs	0.683425	0.703687
NN	MM	ADASYN	NN__activation : identity; NN__alpha : 0.07632752579983847; NN__hidden_layer_sizes : 40; NN__max_iter : 50; NN__random_state : 0; NN__solver : lbfgs	0.68341	0.704611
NN	MM	ADASYN	NN__activation : identity; NN__alpha : 0.0005233146462783461; NN__hidden_layer_sizes : 35;	0.683392	0.704476

			NN__max_iter : 215; NN__random_state : 0; NN__solver : lbfgs		
NN	MM	ADASYN	NN__activation : identity; NN__alpha : 0.46260455085651814; NN__hidden_layer_sizes : 50; NN__max_iter : 50; NN__random_state : 0; NN__solver : lbfgs	0.683358	0.704315
NN	MM	ADASYN	NN__activation : identity; NN__alpha : 1.1910302863117617; NN__hidden_layer_sizes : 50; NN__max_iter : 50; NN__random_state : 0; NN__solver : lbfgs	0.68334	0.703931
NN	MM	ADASYN	NN__activation : relu; NN__alpha : 10.0; NN__hidden_layer_sizes : 15; NN__max_iter : 273; NN__random_state : 0; NN__solver : adam	0.683258	0.694635
NN	MM	ADASYN	NN__activation : identity; NN__alpha : 0.0001; NN__hidden_layer_sizes : 25; NN__max_iter : 251; NN__random_state : 0; NN__solver : lbfgs	0.683014	0.704491
NN	SS	SMOTE	NN__activation : logistic; NN__alpha : 10.0; NN__hidden_layer_sizes : 50; NN__max_iter : 50; NN__random_state : 0; NN__solver : lbfgs	0.683012	0.703736
NN	MM	ADASYN	NN__activation : identity; NN__alpha : 0.006108717856811489; NN__hidden_layer_sizes : 30; NN__max_iter : 169; NN__random_state : 0; NN__solver : lbfgs	0.682997	0.704472
NN	MM	ADASYN	NN__activation : identity; NN__alpha : 0.003515280422858324; NN__hidden_layer_sizes : 50; NN__max_iter : 313; NN__random_state : 0; NN__solver : lbfgs	0.682979	0.7045
NN	MM	ADASYN	NN__activation : identity; NN__alpha : 0.047220733687036824; NN__hidden_layer_sizes : 30; NN__max_iter : 268; NN__random_state : 0; NN__solver : lbfgs	0.682961	0.704428

NN	MM	ADASYN	NN__activation : identity; NN__alpha : 0.7101916142921896; NN__hidden_layer_sizes : 50; NN__max_iter : 50; NN__random_state : 0; NN__solver : lbfgs	0.682945	0.7041
XGB	MM	SMOTE	XGB__colsample_bytree : 0.6423885696122095; XGB__gamma : 5.416358342759316; XGB__max_depth : 1; XGB__min_child_weight : 0.0; XGB__random_state : 0; XGB__scale_pos_weight : 4.1828896028689755; XGB__subsample : 0.9	0.682913	0.764909
NN	MM	ROS	NN__activation : identity; NN__alpha : 0.009832373675930922; NN__hidden_layer_sizes : 50; NN__max_iter : 500; NN__random_state : 0; NN__solver : adam	0.682769	0.698948
NN	MM	ROS	NN__activation : logistic; NN__alpha : 0.470830948133204; NN__hidden_layer_sizes : 3; NN__max_iter : 500; NN__random_state : 0; NN__solver : lbfgs	0.68272	0.702675
NN	MM	ADASYN	NN__activation : identity; NN__alpha : 0.0001; NN__hidden_layer_sizes : 27; NN__max_iter : 213; NN__random_state : 0; NN__solver : lbfgs	0.682635	0.704515
NN	MM	ADASYN	NN__activation : identity; NN__alpha : 0.2077497530138646; NN__hidden_layer_sizes : 50; NN__max_iter : 50; NN__random_state : 0; NN__solver : lbfgs	0.682617	0.704387
NN	MM	ADASYN	NN__activation : identity; NN__alpha : 1.1411043710947752; NN__hidden_layer_sizes : 50; NN__max_iter : 50; NN__random_state : 0; NN__solver : lbfgs	0.682548	0.704071
XGB	Not used	Not used	XGB__colsample_bytree : 1.0; XGB__gamma : 10.94345897801498; XGB__max_depth : 1; XGB__min_child_weight : 0.0; XGB__random_state :	0.682527	0.741475

			0; XGB__scale_pos_weight : 3.133561678682152; XGB__subsample : 0.9		
XGB	SS	ADASYN	XGB__colsample_bytree : 1.0; XGB__gamma : 9.102724944750237; XGB__max_depth : 1; XGB__min_child_weight : 0.0; XGB__random_state : 0; XGB__scale_pos_weight : 0.5; XGB__subsample : 0.5	0.682503	0.699383
XGB	Not used	Not used	XGB__colsample_bytree : 0.4307331304755524; XGB__gamma : 13.863043802398858; XGB__max_depth : 7; XGB__min_child_weight : 2.3115453865193447; XGB__random_state : 0; XGB__scale_pos_weight : 3.335600082637225; XGB__subsample : 0.7814117169683723	0.682349	0.757209
NN	MM	ADASYN	NN__activation : relu; NN__alpha : 5.498044012529747; NN__hidden_layer_sizes : 15; NN__max_iter : 500; NN__random_state : 0; NN__solver : adam	0.682054	0.695197
NN	SS	ROS	NN__activation : logistic; NN__alpha : 3.9229485139656597; NN__hidden_layer_sizes : 5; NN__max_iter : 245; NN__random_state : 0; NN__solver : adam	0.682031	0.697034
XGB	SS	SMOTE	XGB__colsample_bytree : 1.0; XGB__gamma : 7.393987926889745; XGB__max_depth : 1; XGB__min_child_weight : 6.3651285508379045; XGB__random_state : 0; XGB__scale_pos_weight : 5.0; XGB__subsample : 0.9	0.681965	0.752082
XGB	SS	ADASYN	XGB__colsample_bytree : 0.6346538594327273; XGB__gamma : 0.01; XGB__max_depth : 1; XGB__min_child_weight : 0.0; XGB__random_state : 0; XGB__scale_pos_weight : 0.5; XGB__subsample : 0.9	0.681964	0.778492

XGB	SS	SMOTE	XGB__colsample_bytree : 0.1; XGB__gamma : 0.01; XGB__max_depth : 1; XGB__min_child_weight : 8.649141882223175; XGB__random_state : 0; XGB__scale_pos_weight : 5.0; XGB__subsample : 0.9	0.681866	0.763403
XGB	SS	ADASYN	XGB__colsample_bytree : 1.0; XGB__gamma : 5.179712432626178; XGB__max_depth : 1; XGB__min_child_weight : 0.0; XGB__random_state : 0; XGB__scale_pos_weight : 1.4529652701728988; XGB__subsample : 0.9	0.681865	0.762017
XGB	MM	SMOTE	XGB__colsample_bytree : 0.5063398125315677; XGB__gamma : 9.711199250149726; XGB__max_depth : 1; XGB__min_child_weight : 0.0; XGB__random_state : 0; XGB__scale_pos_weight : 5.0; XGB__subsample : 0.9	0.681785	0.743323
XGB	MM	ROS	XGB__colsample_bytree : 1.0; XGB__gamma : 20.288359564977508; XGB__max_depth : 4; XGB__min_child_weight : 8.587909765136308; XGB__random_state : 0; XGB__scale_pos_weight : 4.748599083219661; XGB__subsample : 0.6779029474061055	0.681703	0.758022
XGB	MM	SMOTE	XGB__colsample_bytree : 1.0; XGB__gamma : 8.536029719846876; XGB__max_depth : 1; XGB__min_child_weight : 0.0; XGB__random_state : 0; XGB__scale_pos_weight : 5.0; XGB__subsample : 0.9	0.681694	0.752827
XGB	MM	SMOTE	XGB__colsample_bytree : 0.20863147515778596; XGB__gamma : 0.01; XGB__max_depth : 1; XGB__min_child_weight : 0.0; XGB__random_state : 0; XGB__scale_pos_weight : 4.400072765013536; XGB__subsample : 0.9	0.681225	0.777622

NN	SS	ROS	NN__activation : relu; NN__alpha : 4.975636992427353; NN__hidden_layer_sizes : 6; NN__max_iter : 135; NN__random_state : 0; NN__solver : adam	0.681115	0.686752
NN	MM	ROS	NN__activation : tanh; NN__alpha : 0.0001; NN__hidden_layer_sizes : 50; NN__max_iter : 500; NN__random_state : 0; NN__solver : adam	0.680911	0.69967
NN	SS	ROS	NN__activation : identity; NN__alpha : 0.04506632524476747; NN__hidden_layer_sizes : 8; NN__max_iter : 389; NN__random_state : 0; NN__solver : adam	0.680792	0.695909
XGB	MM	ROS	XGB__colsample_bytree : 1.0; XGB__gamma : 18.676681527877843; XGB__max_depth : 2; XGB__min_child_weight : 0.0; XGB__random_state : 0; XGB__scale_pos_weight : 4.048327124084032; XGB__subsample : 0.696019224850727	0.680786	0.761447
NN	MM	ROS	NN__activation : tanh; NN__alpha : 0.6395547662686945; NN__hidden_layer_sizes : 22; NN__max_iter : 500; NN__random_state : 0; NN__solver : adam	0.6806	0.696224
NN	SS	SMOTE	NN__activation : tanh; NN__alpha : 0.3184983766348907; NN__hidden_layer_sizes : 35; NN__max_iter : 237; NN__random_state : 0; NN__solver : adam	0.680529	0.726873
XGB	SS	ADASYN	XGB__colsample_bytree : 1.0; XGB__gamma : 7.144083521934234; XGB__max_depth : 1; XGB__min_child_weight : 0.0; XGB__random_state : 0; XGB__scale_pos_weight : 3.208751930190358; XGB__subsample : 0.5	0.680509	0.761548
NN	MM	ROS	NN__activation : tanh; NN__alpha : 0.00037911622348291683; NN__hidden_layer_sizes :	0.680497	0.698334

			27; NN__max_iter : 500; NN__random_state : 0; NN__solver : adam		
NN	MM	ADASYN	NN__activation : logistic; NN__alpha : 0.0001; NN__hidden_layer_sizes : 15; NN__max_iter : 335; NN__random_state : 0; NN__solver : adam	0.68048	0.694317
XGB	MM	ADASYN	XGB__colsample_bytree : 0.1; XGB__gamma : 0.01; XGB__max_depth : 1; XGB__min_child_weight : 2.2277798404256646; XGB__random_state : 0; XGB__scale_pos_weight : 5.0; XGB__subsample : 0.9	0.680474	0.770889
XGB	SS	ROS	XGB__colsample_bytree : 0.1; XGB__gamma : 9.249654634328682; XGB__max_depth : 1; XGB__min_child_weight : 0.0; XGB__random_state : 0; XGB__scale_pos_weight : 5.0; XGB__subsample : 0.5	0.680474	0.746708
XGB	SS	ADASYN	XGB__colsample_bytree : 1.0; XGB__gamma : 10.22900649487368; XGB__max_depth : 1; XGB__min_child_weight : 0.0; XGB__random_state : 0; XGB__scale_pos_weight : 5.0; XGB__subsample : 0.9	0.680367	0.745488
NN	SS	SMOTE	NN__activation : tanh; NN__alpha : 10.0; NN__hidden_layer_sizes : 50; NN__max_iter : 500; NN__random_state : 0; NN__solver : adam	0.680326	0.703895
NN	SS	SMOTE	NN__activation : identity; NN__alpha : 10.0; NN__hidden_layer_sizes : 50; NN__max_iter : 467; NN__random_state : 0; NN__solver : adam	0.680223	0.703722
XGB	SS	ADASYN	XGB__colsample_bytree : 0.1; XGB__gamma : 0.01; XGB__max_depth : 1; XGB__min_child_weight : 0.0; XGB__random_state : 0; XGB__scale_pos_weight : 5.0; XGB__subsample : 0.5356068644759493	0.680122	0.762193

NN	MM	ROS	NN__activation : relu; NN__alpha : 5.00909433339747; NN__hidden_layer_sizes : 3; NN__max_iter : 304; NN__random_state : 0; NN__solver : lbfgs	0.680052	0.693821
XGB	SS	ROS	XGB__colsample_bytree : 1.0; XGB__gamma : 0.01; XGB__max_depth : 1; XGB__min_child_weight : 0.0; XGB__random_state : 0; XGB__scale_pos_weight : 2.273230091187787; XGB__subsample : 0.9	0.679928	0.795125
NN	MM	ROS	NN__activation : tanh; NN__alpha : 0.0020259931451460577; NN__hidden_layer_sizes : 24; NN__max_iter : 434; NN__random_state : 0; NN__solver : adam	0.679793	0.700111
NN	MM	ROS	NN__activation : identity; NN__alpha : 0.009117434544910765; NN__hidden_layer_sizes : 30; NN__max_iter : 500; NN__random_state : 0; NN__solver : adam	0.679773	0.698129
NN	SS	SMOTE	NN__activation : relu; NN__alpha : 10.0; NN__hidden_layer_sizes : 3; NN__max_iter : 50; NN__random_state : 0; NN__solver : lbfgs	0.679766	0.704621
NN	MM	ADASYN	NN__activation : logistic; NN__alpha : 0.000126477357080222; NN__hidden_layer_sizes : 15; NN__max_iter : 500; NN__random_state : 0; NN__solver : adam	0.679344	0.694501
XGB	SS	ROS	XGB__colsample_bytree : 0.1; XGB__gamma : 0.01; XGB__max_depth : 1; XGB__min_child_weight : 0.0; XGB__random_state : 0; XGB__scale_pos_weight : 1.5754883765595538; XGB__subsample : 0.9	0.679319	0.784787
XGB	SS	ROS	XGB__colsample_bytree : 0.1; XGB__gamma : 0.01; XGB__max_depth : 1; XGB__min_child_weight : 0.0; XGB__random_state : 0; XGB__scale_pos_weight : 5.0; XGB__subsample : 0.9	0.679254	0.785042

XGB	MM	ADASYN	XGB__colsample_bytree : 1.0; XGB__gamma : 2.9461063871502726; XGB__max_depth : 2; XGB__min_child_weight : 0.0; XGB__random_state : 0; XGB__scale_pos_weight : 0.5; XGB__subsample : 0.9	0.679135	0.792225
NN	SS	SMOTE	NN__activation : identity; NN__alpha : 10.0; NN__hidden_layer_sizes : 3; NN__max_iter : 500; NN__random_state : 0; NN__solver : lbfgs	0.679052	0.703677
NN	MM	ROS	NN__activation : logistic; NN__alpha : 0.0001; NN__hidden_layer_sizes : 40; NN__max_iter : 500; NN__random_state : 0; NN__solver : adam	0.67895	0.692553
NN	MM	ROS	NN__activation : relu; NN__alpha : 0.07874106728803539; NN__hidden_layer_sizes : 24; NN__max_iter : 500; NN__random_state : 0; NN__solver : adam	0.678942	0.7182
XGB	MM	ADASYN	XGB__colsample_bytree : 1.0; XGB__gamma : 4.115447640089052; XGB__max_depth : 1; XGB__min_child_weight : 0.0; XGB__random_state : 0; XGB__scale_pos_weight : 0.5; XGB__subsample : 0.9	0.678862	0.748522
NN	SS	SMOTE	NN__activation : tanh; NN__alpha : 0.0008219253949873161; NN__hidden_layer_sizes : 50; NN__max_iter : 50; NN__random_state : 0; NN__solver : adam	0.678568	0.708027
XGB	SS	ROS	XGB__colsample_bytree : 0.1; XGB__gamma : 0.01; XGB__max_depth : 1; XGB__min_child_weight : 0.0; XGB__random_state : 0; XGB__scale_pos_weight : 4.267710064533322; XGB__subsample : 0.6793920884966658	0.678484	0.782205

NN	SS	SMOTE	NN__activation : identity; NN__alpha : 10.0; NN__hidden_layer_sizes : 50; NN__max_iter : 50; NN__random_state : 0; NN__solver : lbfgs	0.678294	0.703668
XGB	MM	ROS	XGB__colsample_bytree : 0.1; XGB__gamma : 11.78700499760513; XGB__max_depth : 1; XGB__min_child_weight : 10.423163741144396; XGB__random_state : 0; XGB__scale_pos_weight : 4.343460292055702; XGB__subsample : 0.7840051242138955	0.67824	0.735535
NN	MM	ADASYN	NN__activation : logistic; NN__alpha : 0.36185798550473247; NN__hidden_layer_sizes : 30; NN__max_iter : 500; NN__random_state : 0; NN__solver : lbfgs	0.678088	0.707209
XGB	MM	ROS	XGB__colsample_bytree : 1.0; XGB__gamma : 18.571241977413063; XGB__max_depth : 1; XGB__min_child_weight : 10.863276176061525; XGB__random_state : 0; XGB__scale_pos_weight : 4.07032376883234; XGB__subsample : 0.9	0.678063	0.72776
XGB	MM	ADASYN	XGB__colsample_bytree : 0.1; XGB__gamma : 0.01; XGB__max_depth : 1; XGB__min_child_weight : 0.0; XGB__random_state : 0; XGB__scale_pos_weight : 2.146007328437145; XGB__subsample : 0.5	0.678002	0.767306
NN	MM	ADASYN	NN__activation : identity; NN__alpha : 4.548287284206085; NN__hidden_layer_sizes : 30; NN__max_iter : 500; NN__random_state : 0; NN__solver : lbfgs	0.677968	0.70068
NN	SS	SMOTE	NN__activation : tanh; NN__alpha : 10.0; NN__hidden_layer_sizes : 3; NN__max_iter : 500; NN__random_state : 0; NN__solver : adam	0.677901	0.706392
XGB	SS	ROS	XGB__colsample_bytree : 0.1; XGB__gamma : 0.01; XGB__max_depth : 1; XGB__min_child_weight : 0.0;	0.677732	0.779141

			XGB__random_state : 0; XGB__scale_pos_weight : 0.5; XGB__subsample : 0.7087165941778301		
NN	MM	ROS	NN__activation : tanh; NN__alpha : 0.17129999616900296; NN__hidden_layer_sizes : 25; NN__max_iter : 500; NN__random_state : 0; NN__solver : adam	0.67762	0.698219
NN	SS	SMOTE	NN__activation : identity; NN__alpha : 0.28534423108597284; NN__hidden_layer_sizes : 3; NN__max_iter : 50; NN__random_state : 0; NN__solver : lbfgs	0.677468	0.703823
NN	MM	ROS	NN__activation : tanh; NN__alpha : 0.0001; NN__hidden_layer_sizes : 22; NN__max_iter : 500; NN__random_state : 0; NN__solver : adam	0.677328	0.698248
NN	MM	ADASYN	NN__activation : tanh; NN__alpha : 3.800332467979972; NN__hidden_layer_sizes : 50; NN__max_iter : 470; NN__random_state : 0; NN__solver : lbfgs	0.677312	0.702124
XGB	MM	ROS	XGB__colsample_bytree : 1.0; XGB__gamma : 0.01; XGB__max_depth : 8; XGB__min_child_weight : 50.0; XGB__random_state : 0; XGB__scale_pos_weight : 5.0; XGB__subsample : 0.8336733407808952	0.677255	0.71603
XGB	MM	ROS	XGB__colsample_bytree : 1.0; XGB__gamma : 12.798674701129748; XGB__max_depth : 1; XGB__min_child_weight : 1.9168720632159062; XGB__random_state : 0; XGB__scale_pos_weight : 4.5293176600106415; XGB__subsample : 0.8279458585317834	0.676837	0.744884
NN	SS	SMOTE	NN__activation : identity; NN__alpha : 1.2278635248432284; NN__hidden_layer_sizes : 27; NN__max_iter : 165; NN__random_state : 0; NN__solver : adam	0.67671	0.703396

NN	SS	SMOTE	NN__activation : identity; NN__alpha : 0.42310533834131386; NN__hidden_layer_sizes : 50; NN__max_iter : 50; NN__random_state : 0; NN__solver : lbfgs	0.676676	0.70379
NN	SS	SMOTE	NN__activation : identity; NN__alpha : 0.00011158207754410737; NN__hidden_layer_sizes : 3; NN__max_iter : 165; NN__random_state : 0; NN__solver : adam	0.676641	0.693495
XGB	MM	ROS	XGB__colsample_bytree : 1.0; XGB__gamma : 0.01; XGB__max_depth : 1; XGB__min_child_weight : 50.0; XGB__random_state : 0; XGB__scale_pos_weight : 3.3767277508006277; XGB__subsample : 0.9	0.676483	0.70437
NN	SS	SMOTE	NN__activation : identity; NN__alpha : 0.0025231362554514265; NN__hidden_layer_sizes : 3; NN__max_iter : 50; NN__random_state : 0; NN__solver : lbfgs	0.676297	0.703886
NN	MM	ROS	NN__activation : tanh; NN__alpha : 0.3184983766348907; NN__hidden_layer_sizes : 35; NN__max_iter : 237; NN__random_state : 0; NN__solver : adam	0.676292	0.698286
NN	MM	ROS	NN__activation : tanh; NN__alpha : 0.009079456243786288; NN__hidden_layer_sizes : 30; NN__max_iter : 500; NN__random_state : 0; NN__solver : adam	0.676276	0.698584
XGB	SS	ROS	XGB__colsample_bytree : 0.1; XGB__gamma : 6.693041360995059; XGB__max_depth : 1; XGB__min_child_weight : 0.0; XGB__random_state : 0; XGB__scale_pos_weight : 2.7080598523485593; XGB__subsample : 0.5	0.67618	0.755519
XGB	SS	ROS	XGB__colsample_bytree : 1.0; XGB__gamma : 16.224461980171178; XGB__max_depth : 1;	0.676097	0.736449

			XGB__min_child_weight : 0.0; XGB__random_state : 0; XGB__scale_pos_weight : 4.452545569234384; XGB__subsample : 0.5		
XGB	MM	ADASYN	XGB__colsample_bytree : 1.0; XGB__gamma : 3.944544252994107; XGB__max_depth : 1; XGB__min_child_weight : 0.0; XGB__random_state : 0; XGB__scale_pos_weight : 0.5; XGB__subsample : 0.5	0.676006	0.760928
NN	MM	SMOTE	NN__activation : identity; NN__alpha : 0.0001; NN__hidden_layer_sizes : 35; NN__max_iter : 253; NN__random_state : 0; NN__solver : lbfgs	0.675953	0.703532
XGB	SS	ROS	XGB__colsample_bytree : 0.1; XGB__gamma : 0.01; XGB__max_depth : 2; XGB__min_child_weight : 32.99293216572262; XGB__random_state : 0; XGB__scale_pos_weight : 5.0; XGB__subsample : 0.5	0.675891	0.69789
XGB	MM	ROS	XGB__colsample_bytree : 1.0; XGB__gamma : 14.16520226699194; XGB__max_depth : 5; XGB__min_child_weight : 0.0; XGB__random_state : 0; XGB__scale_pos_weight : 5.0; XGB__subsample : 0.7083605219364747	0.675755	0.837673
NN	MM	ROS	NN__activation : tanh; NN__alpha : 9.681467416121485; NN__hidden_layer_sizes : 25; NN__max_iter : 304; NN__random_state : 0; NN__solver : lbfgs	0.675595	0.683046
NN	MM	ROS	NN__activation : tanh; NN__alpha : 9.860917988825792; NN__hidden_layer_sizes : 50; NN__max_iter : 311; NN__random_state : 0; NN__solver : lbfgs	0.675579	0.682809
XGB	SS	ROS	XGB__colsample_bytree : 1.0; XGB__gamma : 12.37581754675186; XGB__max_depth : 1; XGB__min_child_weight : 0.0; XGB__random_state :	0.675289	0.747802

			0; XGB__scale_pos_weight : 5.0; XGB__subsample : 0.9		
NN	MM	ROS	NN__activation : relu; NN__alpha : 10.0; NN__hidden_layer_sizes : 50; NN__max_iter : 133; NN__random_state : 0; NN__solver : lbfgs	0.675182	0.682593
NN	MM	SMOTE	NN__activation : identity; NN__alpha : 0.0001; NN__hidden_layer_sizes : 15; NN__max_iter : 192; NN__random_state : 0; NN__solver : lbfgs	0.675161	0.703525
NN	MM	ROS	NN__activation : relu; NN__alpha : 10.0; NN__hidden_layer_sizes : 30; NN__max_iter : 229; NN__random_state : 0; NN__solver : lbfgs	0.675113	0.683062
NN	SS	SMOTE	NN__activation : tanh; NN__alpha : 0.0001; NN__hidden_layer_sizes : 7; NN__max_iter : 463; NN__random_state : 0; NN__solver : adam	0.675108	0.709278
XGB	MM	ROS	XGB__colsample_bytree : 1.0; XGB__gamma : 26.227304370768316; XGB__max_depth : 8; XGB__min_child_weight : 0.0; XGB__random_state : 0; XGB__scale_pos_weight : 4.184661234690365; XGB__subsample : 0.6286807682625302	0.674934	0.766198
XGB	MM	ADASYN	XGB__colsample_bytree : 0.1; XGB__gamma : 0.01; XGB__max_depth : 1; XGB__min_child_weight : 0.0; XGB__random_state : 0; XGB__scale_pos_weight : 0.5; XGB__subsample : 0.5	0.674908	0.766469
NN	MM	ROS	NN__activation : identity; NN__alpha : 1.2278635248432284; NN__hidden_layer_sizes : 27; NN__max_iter : 165; NN__random_state : 0; NN__solver : adam	0.674884	0.690522
NN	MM	SMOTE	NN__activation : identity; NN__alpha : 0.0010098554274638714; NN__hidden_layer_sizes : 3; NN__max_iter : 50; NN__random_state : 0; NN__solver : lbfgs	0.674782	0.70351

NN	MM	ROS	NN__activation : tanh; NN__alpha : 10.0; NN__hidden_layer_sizes : 9; NN__max_iter : 322; NN__random_state : 0; NN__solver : lbfgs	0.674769	0.68255
NN	MM	SMOTE	NN__activation : identity; NN__alpha : 0.24292043584111792; NN__hidden_layer_sizes : 50; NN__max_iter : 417; NN__random_state : 0; NN__solver : lbfgs	0.674747	0.703432
NN	MM	SMOTE	NN__activation : identity; NN__alpha : 0.0001; NN__hidden_layer_sizes : 50; NN__max_iter : 363; NN__random_state : 0; NN__solver : lbfgs	0.674403	0.703587
NN	MM	SMOTE	NN__activation : identity; NN__alpha : 0.6926685392029206; NN__hidden_layer_sizes : 50; NN__max_iter : 310; NN__random_state : 0; NN__solver : lbfgs	0.674403	0.70353
NN	MM	ROS	NN__activation : identity; NN__alpha : 10.0; NN__hidden_layer_sizes : 50; NN__max_iter : 50; NN__random_state : 0; NN__solver : lbfgs	0.67439	0.682385
NN	MM	SMOTE	NN__activation : identity; NN__alpha : 0.08251180234141221; NN__hidden_layer_sizes : 8; NN__max_iter : 349; NN__random_state : 0; NN__solver : lbfgs	0.674369	0.703544
NN	MM	ROS	NN__activation : tanh; NN__alpha : 10.0; NN__hidden_layer_sizes : 35; NN__max_iter : 50; NN__random_state : 0; NN__solver : lbfgs	0.674356	0.682472
XGB	MM	ADASYN	XGB__colsample_bytree : 0.1; XGB__gamma : 0.01; XGB__max_depth : 1; XGB__min_child_weight : 0.0; XGB__random_state : 0; XGB__scale_pos_weight : 0.5; XGB__subsample : 0.9	0.674064	0.773944
XGB	MM	ROS	XGB__colsample_bytree : 0.977542367912643; XGB__gamma : 0.01; XGB__max_depth : 5; XGB__min_child_weight : 42.84753342717848;	0.674018	0.719112

			XGB__random_state : 0; XGB__scale_pos_weight : 4.246001826810197; XGB__subsample : 0.7759245107451025		
NN	MM	SMOTE	NN__activation : identity; NN__alpha : 0.0008112873099284723; NN__hidden_layer_sizes : 30; NN__max_iter : 270; NN__random_state : 0; NN__solver : lbfgs	0.67399	0.703625
XGB	MM	ADASYN	XGB__colsample_bytree : 0.656043020699853; XGB__gamma : 4.422609650361867; XGB__max_depth : 1; XGB__min_child_weight : 0.0; XGB__random_state : 0; XGB__scale_pos_weight : 1.25910817089834; XGB__subsample : 0.9	0.673682	0.760474
XGB	MM	ROS	XGB__colsample_bytree : 0.19586410089367562; XGB__gamma : 0.01; XGB__max_depth : 8; XGB__min_child_weight : 50.0; XGB__random_state : 0; XGB__scale_pos_weight : 2.9567241112383504; XGB__subsample : 0.9	0.673517	0.696277
XGB	MM	ADASYN	XGB__colsample_bytree : 0.5502867522567378; XGB__gamma : 0.01; XGB__max_depth : 1; XGB__min_child_weight : 0.0; XGB__random_state : 0; XGB__scale_pos_weight : 0.5; XGB__subsample : 0.9	0.673444	0.776363
XGB	SS	ROS	XGB__colsample_bytree : 0.2528104392554187; XGB__gamma : 0.01; XGB__max_depth : 2; XGB__min_child_weight : 0.0; XGB__random_state : 0; XGB__scale_pos_weight : 2.660506520436399; XGB__subsample : 0.5	0.673193	0.801765
NN	MM	SMOTE	NN__activation : identity; NN__alpha : 1.7914584450333455; NN__hidden_layer_sizes : 5; NN__max_iter : 500; NN__random_state : 0; NN__solver : lbfgs	0.673077	0.703008

XGB	MM	ADASYN	XGB__colsample_bytree : 0.1; XGB__gamma : 0.01; XGB__max_depth : 1; XGB__min_child_weight : 0.0; XGB__random_state : 0; XGB__scale_pos_weight : 3.0451191007968426; XGB__subsample : 0.5	0.672903	0.767275
XGB	SS	ROS	XGB__colsample_bytree : 0.1; XGB__gamma : 0.01; XGB__max_depth : 8; XGB__min_child_weight : 24.595248366811877; XGB__random_state : 0; XGB__scale_pos_weight : 1.3841357752604413; XGB__subsample : 0.5	0.672884	0.674677
NN	MM	SMOTE	NN__activation : identity; NN__alpha : 1.765456809010971; NN__hidden_layer_sizes : 50; NN__max_iter : 254; NN__random_state : 0; NN__solver : lbfgs	0.672663	0.702987
XGB	MM	ROS	XGB__colsample_bytree : 0.1; XGB__gamma : 0.01; XGB__max_depth : 8; XGB__min_child_weight : 37.16045353144856; XGB__random_state : 0; XGB__scale_pos_weight : 5.0; XGB__subsample : 0.632693392817871	0.672627	0.705128
XGB	MM	ADASYN	XGB__colsample_bytree : 1.0; XGB__gamma : 4.412403807080667; XGB__max_depth : 1; XGB__min_child_weight : 0.0; XGB__random_state : 0; XGB__scale_pos_weight : 0.5; XGB__subsample : 0.9	0.672544	0.746808
NN	MM	ADASYN	NN__activation : relu; NN__alpha : 2.7354342694498306; NN__hidden_layer_sizes : 50; NN__max_iter : 229; NN__random_state : 0; NN__solver : lbfgs	0.672399	0.715191
XGB	MM	ROS	XGB__colsample_bytree : 0.1; XGB__gamma : 0.01; XGB__max_depth : 8; XGB__min_child_weight : 50.0; XGB__random_state : 0; XGB__scale_pos_weight : 3.448386126656192; XGB__subsample : 0.9	0.672285	0.702211

XGB	MM	ROS	XGB__colsample_bytree : 1.0; XGB__gamma : 0.01; XGB__max_depth : 8; XGB__min_child_weight : 50.0; XGB__random_state : 0; XGB__scale_pos_weight : 4.449305626418177; XGB__subsample : 0.9	0.671887	0.719422
XGB	MM	ROS	XGB__colsample_bytree : 1.0; XGB__gamma : 0.01; XGB__max_depth : 3; XGB__min_child_weight : 50.0; XGB__random_state : 0; XGB__scale_pos_weight : 4.542984599714905; XGB__subsample : 0.9	0.671818	0.719818
XGB	MM	ROS	XGB__colsample_bytree : 1.0; XGB__gamma : 0.01; XGB__max_depth : 8; XGB__min_child_weight : 50.0; XGB__random_state : 0; XGB__scale_pos_weight : 4.382539245399; XGB__subsample : 0.9	0.671508	0.718997
NN	MM	SMOTE	NN__activation : relu; NN__alpha : 2.0532223750546117; NN__hidden_layer_sizes : 3; NN__max_iter : 50; NN__random_state : 0; NN__solver : lbfgs	0.671474	0.703429
NN	MM	SMOTE	NN__activation : tanh; NN__alpha : 1.2688048350010857; NN__hidden_layer_sizes : 20; NN__max_iter : 232; NN__random_state : 0; NN__solver : lbfgs	0.671179	0.71356
XGB	MM	ADASYN	XGB__colsample_bytree : 0.5949006479896596; XGB__gamma : 0.01; XGB__max_depth : 1; XGB__min_child_weight : 0.0; XGB__random_state : 0; XGB__scale_pos_weight : 1.8166923233341714; XGB__subsample : 0.9	0.67112	0.781039
NN	MM	SMOTE	NN__activation : relu; NN__alpha : 0.001160161703428094; NN__hidden_layer_sizes : 27; NN__max_iter : 483; NN__random_state : 0; NN__solver : adam	0.671087	0.71523

NN	MM	SMOTE	NN__activation : logistic; NN__alpha : 0.0001; NN__hidden_layer_sizes : 50; NN__max_iter : 362; NN__random_state : 0; NN__solver : adam	0.671045	0.696511
XGB	MM	ADASYN	XGB__colsample_bytree : 0.6558242728770811; XGB__gamma : 0.01; XGB__max_depth : 1; XGB__min_child_weight : 0.0; XGB__random_state : 0; XGB__scale_pos_weight : 1.709460839167244; XGB__subsample : 0.5	0.671033	0.777726
XGB	MM	ROS	XGB__colsample_bytree : 1.0; XGB__gamma : 0.01; XGB__max_depth : 8; XGB__min_child_weight : 50.0; XGB__random_state : 0; XGB__scale_pos_weight : 5.0; XGB__subsample : 0.9	0.670905	0.72315
XGB	MM	ADASYN	XGB__colsample_bytree : 1.0; XGB__gamma : 0.01; XGB__max_depth : 1; XGB__min_child_weight : 0.0; XGB__random_state : 0; XGB__scale_pos_weight : 0.5; XGB__subsample : 0.9	0.670807	0.77777
XGB	MM	ROS	XGB__colsample_bytree : 1.0; XGB__gamma : 0.01; XGB__max_depth : 8; XGB__min_child_weight : 45.48985994195283; XGB__random_state : 0; XGB__scale_pos_weight : 3.2999249694761894; XGB__subsample : 0.8140835049941484	0.670732	0.706964
NN	MM	SMOTE	NN__activation : logistic; NN__alpha : 0.0001; NN__hidden_layer_sizes : 25; NN__max_iter : 500; NN__random_state : 0; NN__solver : adam	0.670702	0.693815
XGB	MM	ROS	XGB__colsample_bytree : 1.0; XGB__gamma : 15.671258767745782; XGB__max_depth : 1; XGB__min_child_weight : 0.0; XGB__random_state : 0; XGB__scale_pos_weight : 3.391506876320594; XGB__subsample : 0.6659907592909782	0.670603	0.739969
XGB	MM	ROS	XGB__colsample_bytree : 1.0; XGB__gamma : 0.01; XGB__max_depth : 8; XGB__min_child_weight : 50.0;	0.670422	0.717701

			XGB__random_state : 0; XGB__scale_pos_weight : 4.016696892589927; XGB__subsample : 0.9		
XGB	SS	ROS	XGB__colsample_bytree : 1.0; XGB__gamma : 0.01; XGB__max_depth : 8; XGB__min_child_weight : 50.0; XGB__random_state : 0; XGB__scale_pos_weight : 3.9880396450717157; XGB__subsample : 0.9	0.670355	0.717558
NN	MM	SMOTE	NN__activation : identity; NN__alpha : 7.08537176228385; NN__hidden_layer_sizes : 3; NN__max_iter : 50; NN__random_state : 0; NN__solver : lbfgs	0.670322	0.69514
XGB	MM	ROS	XGB__colsample_bytree : 0.1; XGB__gamma : 0.01; XGB__max_depth : 8; XGB__min_child_weight : 39.810852518617864; XGB__random_state : 0; XGB__scale_pos_weight : 3.6446632953934954; XGB__subsample : 0.6653847072456476	0.670273	0.700436
NN	MM	SMOTE	NN__activation : tanh; NN__alpha : 10.0; NN__hidden_layer_sizes : 15; NN__max_iter : 368; NN__random_state : 0; NN__solver : adam	0.670136	0.683722
XGB	MM	ROS	XGB__colsample_bytree : 0.1; XGB__gamma : 0.01; XGB__max_depth : 1; XGB__min_child_weight : 34.88040223779923; XGB__random_state : 0; XGB__scale_pos_weight : 4.140781589809216; XGB__subsample : 0.592974525787412	0.670063	0.703647
XGB	SS	ROS	XGB__colsample_bytree : 0.47248573380119663; XGB__gamma : 0.01; XGB__max_depth : 8; XGB__min_child_weight : 17.689509660846095; XGB__random_state : 0; XGB__scale_pos_weight : 0.5; XGB__subsample : 0.7887925376076751	0.670039	0.704974
XGB	MM	ROS	XGB__colsample_bytree : 0.1; XGB__gamma : 0.01; XGB__max_depth : 1; XGB__min_child_weight : 50.0; XGB__random_state : 0; XGB__scale_pos_weight :	0.669868	0.695193

			3.6354767291173093; XGB__subsample : 0.8084035061647417		
XGB	SS	ROS	XGB__colsample_bytree : 0.1; XGB__gamma : 0.01; XGB__max_depth : 8; XGB__min_child_weight : 24.87965371428194; XGB__random_state : 0; XGB__scale_pos_weight : 1.9394145340674889; XGB__subsample : 0.5001007667683355	0.669487	0.691502
NN	MM	SMOTE	NN__activation : identity; NN__alpha : 0.0001; NN__hidden_layer_sizes : 27; NN__max_iter : 289; NN__random_state : 0; NN__solver : adam	0.668615	0.701735
XGB	MM	ADASYN	XGB__colsample_bytree : 1.0; XGB__gamma : 5.637148713191554; XGB__max_depth : 1; XGB__min_child_weight : 0.0; XGB__random_state : 0; XGB__scale_pos_weight : 1.5251673265043093; XGB__subsample : 0.9	0.668577	0.760341
NN	MM	SMOTE	NN__activation : relu; NN__alpha : 0.11595947054071278; NN__hidden_layer_sizes : 25; NN__max_iter : 390; NN__random_state : 0; NN__solver : adam	0.66842	0.718557
XGB	MM	ADASYN	XGB__colsample_bytree : 1.0; XGB__gamma : 5.724755061328439; XGB__max_depth : 1; XGB__min_child_weight : 0.0; XGB__random_state : 0; XGB__scale_pos_weight : 1.5355620830927734; XGB__subsample : 0.9	0.668215	0.759417
NN	MM	SMOTE	NN__activation : tanh; NN__alpha : 0.938467758201178; NN__hidden_layer_sizes : 25; NN__max_iter : 357; NN__random_state : 0; NN__solver : adam	0.66796	0.701301
NN	MM	ADASYN	NN__activation : logistic; NN__alpha : 2.2135773244197514; NN__hidden_layer_sizes : 50;	0.66745	0.690171

			NN__max_iter : 452; NN__random_state : 0; NN__solver : lbfgs		
NN	MM	SMOTE	NN__activation : tanh; NN__alpha : 0.3184983766348907; NN__hidden_layer_sizes : 35; NN__max_iter : 237; NN__random_state : 0; NN__solver : adam	0.665548	0.702026
NN	MM	SMOTE	NN__activation : identity; NN__alpha : 0.024409645284325585; NN__hidden_layer_sizes : 35; NN__max_iter : 233; NN__random_state : 0; NN__solver : adam	0.664843	0.701853
NN	MM	SMOTE	NN__activation : tanh; NN__alpha : 0.03869204009250102; NN__hidden_layer_sizes : 25; NN__max_iter : 450; NN__random_state : 0; NN__solver : adam	0.664827	0.703958
NN	MM	SMOTE	NN__activation : identity; NN__alpha : 0.00017727138126991392; NN__hidden_layer_sizes : 25; NN__max_iter : 500; NN__random_state : 0; NN__solver : adam	0.664035	0.702059
NN	MM	ADASYN	NN__activation : relu; NN__alpha : 10.0; NN__hidden_layer_sizes : 50; NN__max_iter : 50; NN__random_state : 0; NN__solver : lbfgs	0.6638	0.687105
NN	MM	SMOTE	NN__activation : tanh; NN__alpha : 0.0001; NN__hidden_layer_sizes : 30; NN__max_iter : 500; NN__random_state : 0; NN__solver : adam	0.66362	0.702466
NN	MM	ADASYN	NN__activation : identity; NN__alpha : 10.0; NN__hidden_layer_sizes : 24; NN__max_iter : 500; NN__random_state : 0; NN__solver : lbfgs	0.663422	0.687001
NN	MM	SMOTE	NN__activation : tanh; NN__alpha : 0.35567522781433475; NN__hidden_layer_sizes : 20; NN__max_iter : 316; NN__random_state : 0; NN__solver : adam	0.663276	0.701488

NN	MM	ADASYN	NN__activation : relu; NN__alpha : 0.3471573597085078; NN__hidden_layer_sizes : 15; NN__max_iter : 500; NN__random_state : 0; NN__solver : adam	0.66322	0.711189
NN	MM	SMOTE	NN__activation : relu; NN__alpha : 3.9814946105106603; NN__hidden_layer_sizes : 15; NN__max_iter : 427; NN__random_state : 0; NN__solver : adam	0.66309	0.692602
NN	MM	ADASYN	NN__activation : identity; NN__alpha : 10.0; NN__hidden_layer_sizes : 8; NN__max_iter : 500; NN__random_state : 0; NN__solver : lbfgs	0.663043	0.686996
NN	MM	SMOTE	NN__activation : tanh; NN__alpha : 1.009315952517627; NN__hidden_layer_sizes : 15; NN__max_iter : 50; NN__random_state : 0; NN__solver : lbfgs	0.662944	0.714652
NN	MM	SMOTE	NN__activation : identity; NN__alpha : 0.23092602763971176; NN__hidden_layer_sizes : 25; NN__max_iter : 325; NN__random_state : 0; NN__solver : adam	0.66283	0.701805
NN	MM	SMOTE	NN__activation : tanh; NN__alpha : 0.15101413680232648; NN__hidden_layer_sizes : 30; NN__max_iter : 259; NN__random_state : 0; NN__solver : adam	0.662741	0.701843
NN	SS	ROS	NN__activation : relu; NN__alpha : 0.7530466730222446; NN__hidden_layer_sizes : 9; NN__max_iter : 443; NN__random_state : 0; NN__solver : lbfgs	0.646437	0.827539
NN	SS	ROS	NN__activation : relu; NN__alpha : 0.0007233655252968397; NN__hidden_layer_sizes : 22; NN__max_iter : 411; NN__random_state : 0; NN__solver : adam	0.638897	0.759154

NN	SS	ROS	NN__activation : relu; NN__alpha : 0.0001; NN__hidden_layer_sizes : 50; NN__max_iter : 50; NN__random_state : 0; NN__solver : lbfgs	0.629326	0.929607
NN	SS	ROS	NN__activation : relu; NN__alpha : 0.01549337141764052; NN__hidden_layer_sizes : 20; NN__max_iter : 372; NN__random_state : 0; NN__solver : lbfgs	0.608999	0.9688
